# Promoting data-driven decision-making in Jordan: strengthening national health information system and achieving consensus on core set of health system indicators

**DOI:** 10.1186/s12978-025-01988-1

**Published:** 2025-05-31

**Authors:** Fadi El-Jardali, Racha Fadlallah, Raeda Abu AlRub, Diana Jamal, Najla Daher

**Affiliations:** 1 Department of Health Management and Policy, American University of Beirut, Beirut, Lebanon; 2 Knowledge to Policy (K2P) Center, American University of Beirut, Beirut, Lebanon; 3 Faculty of Nursing, Jordan University of Science and Technology, Irbid, Jordan

**Keywords:** Health information system, Indicators, Core health systems, Maternal and child health indicators, Adolescent indicators, Refugee health indicators, Jordan, Data-driven decision-making, Consensus meetings

## Abstract

**Background:**

A well-functioning health information system (HIS) is foundational for strong health systems and the achievement of the Sustainable Development Goals. In Jordan, the national HIS, overseen by the Ministry of Health, faces challenges related to overlapping data collection, data availability gaps, and operational inefficiencies which compromise effective decision-making. This study aims to promote data-driven decision-making in Jordan by assessing the existing HIS and fostering consensus on a standardized set of indicators for core health system functions, maternal, child and adolescent health, and refugee health.

**Methods:**

A multifaceted stepwise approach was adopted, encompassing the following steps: baseline assessment of HIS, compilation of a comprehensive list of candidate indicators, consensus meetings to prioritize and validate the indicators, and development of procedure manual for standardizing the shortlisted indicators.

**Results:**

The baseline assessment of HIS identified areas for improvement at the following levels: governance and planning; infrastructure and resources; data management; and institutional capacity to support data-driven decision-making. Of 4,120 indicators reviewed from international sources and 215 from Jordan's indicators inventory, 415 candidate indicators were compiled and categorized into three priority thematic areas: core health and health systems indicators (n = 167), maternal, child and adolescent health indicators (n = 137), and refugee health indicators (n = 111). Fifteen stakeholders took part in the first consensus meeting, 14 in the second, and 10 in the third meeting. Utilizing a criterion-based ranking system, participants independently rated each candidate indicator against three criteria: Importance, Feasibility, and Actionability. The shortlisted indicators were subsequently validated against the criterion ‘retain’. This process resulted in a final validated list of indicators, comprising 55 core health systems indicators (33 of which are reported in Jordan); 40 maternal, child and adolescent health indicators (21 of which are reported in Jordan); and 26 refugee health indicators (none of which are reported in Jordan). Participants also suggested indicators to be added to each thematic areas. Three procedure manuals were developed and validated, corresponding to the three thematic areas.

**Conclusion:**

Findings from this study can contribute to the broader discourse on HIS reforms in Jordan, emphasizing the need for ongoing efforts to enhance data quality, stakeholder collaboration, and infrastructure.

**Supplementary Information:**

The online version contains supplementary material available at 10.1186/s12978-025-01988-1.

## Plain language summary

In this study, we seek to strengthen Jordan's national health information system (HIS), which is overseen by the Ministry of Health, by assessing its current state and establishing a consensus on a standardized set of national indicators. A multistep approach was employed, involving a baseline assessment of the HIS, compilation of potential indicators, consensus meetings, and the creation of procedure manuals for the standardized indicators.Results from the baseline assessment highlighted areas for improvement in governance, planning, infrastructure, resources, data management, and institutional capacity of the HIS. A total of 415 candidate indicators were compiled from Jordan and international sources, focusing on core health systems, maternal, child and adolescent health, as well as refugee health. Stakeholders participated in consensus meetings, using a criteria-based ranking system to independently assess the indicators based on importance, feasibility, and actionability. The final set of validated indicators included 55 core health systems indicators, 40 maternal, child and adolescent health indicators, and 26 refugee health indicators. Participants also suggested additional indicators for inclusion.The study's findings offer valuable insights for national discussions among key stakeholders in Jordan, emphasizing the importance of continuous efforts to enhance data quality, stakeholder collaboration, and infrastructure for strengthening HIS. The approach, tools and manuals can be adapted by other countries to strengthen their own HIS, facilitating data-driven health policymaking on a global scale.

**Supplementary Information:**

The online version contains supplementary material available at 10.1186/s12978-025-01988-1.

## Résumé

**Contexte:**

Un système d’information sanitaire (SIS) qui fonctionne bien est fondamental pour de solides systèmes de santé et la réalisation des objectifs de développement durable. En Jordanie, le SIS national, supervisé par le ministère de la Santé, est confronté à des défis liés au chevauchement des collectes de données, aux lacunes en matière de disponibilité des données et aux inefficacités opérationnelles qui compromettent l’efficacité des prises de décision. Cette étude vise à promouvoir la prise de décision basée sur les données probantes en Jordanie en évaluant le SIS existant et en favorisant un consensus sur un ensemble standardisé d’indicateurs pour les fonctions essentielles du système de santé, la santé maternelle, des enfants et des adolescent(e)s et la santé des réfugiés.

**Méthodes:**

Une approche multidimensionnelle progressive a été adoptée, englobant les étapes suivantes : évaluation de base du SIS, compilation d’une liste exhaustive des indicateurs potentiels, réunions de consensus pour établir l’ordre de priorité et valider les indicateurs, et élaboration d’un manuel de procédures pour la normalisation des indicateurs présélectionnés.

**Résultats:**

L’évaluation de base du SIS a identifié des domaines à améliorer aux niveaux suivants : gouvernance et planification ; infrastructure et ressources ; gestion des données ; et capacité institutionnelle à soutenir la prise de décision fondée sur les données. Sur les 4 120 indicateurs examinés à partir de sources internationales et 215 de l’inventaire des indicateurs de Jordanie, 415 indicateurs potentiels ont été compilés et classés en trois domaines thématiques prioritaires, soit les indicateurs de base de la santé et des systèmes de santé (n = 167), les indicateurs de santé maternelle, infantile et adolescente (n = 137) et les indicateurs de santé des réfugiés (n = 111). Quinze parties prenantes ont participé à la première réunion de consensus, 14 à la deuxième et 10 à la troisième réunion. À l’aide d’un système de classement basé sur des critères prédéfinis, les participants ont évalué indépendamment chaque indicateur potentiel en fonction de trois critères : Importance, Faisabilité et Capacité d’action. Les indicateurs présélectionnés ont ensuite été validés en fonction du critère « à conserver ». Ce processus a donné lieu à une liste définitive validée d’indicateurs, comprenant 55 indicateurs de base des systèmes de santé (dont 33 sont déclarés en Jordanie) ; 40 indicateurs de santé maternelle, infantile et adolescente (dont 21 sont déclarés en Jordanie) ; et 26 indicateurs de santé des réfugiés (dont aucun n’est déclaré en Jordanie). Les participants ont également suggéré d’ajouter des indicateurs à chaque domaine thématique. Trois manuels de procédures ont été élaborés et validés, correspondant aux trois domaines thématiques.

**Conclusion:**

Les résultats de cette étude peuvent contribuer au débat plus large sur les réformes du SIS en Jordanie, en soulignant la nécessité de poursuivre les efforts pour améliorer la qualité des données, la collaboration des parties prenantes et l’infrastructure.

## Background

Reliable and timely health information is the foundation of all health systems [1, 2]. It enables decision-makers at all levels of the health system to identify problems; make evidence-informed decisions on health policies and programs; and optimally allocate scarce resources, with the ultimate aim of achieving health improvements [3–7].

A well-functioning health information system (HIS) is critical for the attainment of the health-related Sustainable Development Goals (SDGs) [8], particularly 'SDG 3: Good Health and Well-being’, as it supports targeted health interventions and robust monitoring of health outcomes, notably in areas such as maternal, child, and adolescent health. Additionally, HIS plays a key role in advancing 'SDG 5: Gender Equality’ and ‘SDG 10: Reduced Inequalities’ by identifying disparities and facilitating efforts and equitable resource allocation to improve health outcomes across diverse populations, thereby enhancing health equity [9, 10]. A robust HIS enables the planning and implementation of actions that mitigate health inequities at all care levels, aligning with the SDG mandate to ‘Leave No One Behind' and ensuring equitable access to essential services like reproductive health [11]. Increasingly, the emphasis on the accuracy and reliability of health data collection has been recognized as pivotal in enhancing health systems and achieving these goals [12].

The COVID-19 pandemic further re-enforced the indispensable role of HIS and the need for leveraging the capabilities of existing HIS to collect, transmit and analyze health data in real-time to understand the epidemiological situation and craft appropriate data-driven strategies and responses [13–15]. During such crises, HIS offers the necessary evidence to make informed decisions, helping policymakers and health professionals act wisely and change their strategies when needed to improve health outcomes [10].

While there is a growing realization of the need for effective and sustained action to strengthen HIS, many countries in the Middle East and North Africa (MENA) region lack well-functioning HIS to support health system strengthening [4, 16–19]. Key challenges include: 1) production of poor-quality data (inaccessible, inaccurate, incomplete, or irrelevant data) that does not align with decision-makers’ needs; 2) duplication, fragmentation and unnecessary production of data that become a burden and a barrier to effective information use; 3) a critical shortage of skilled health personnel who are equipped with the knowledge and capacity to collect and report essential health indicators and 4) poor dissemination of data in decision-making and health systems strengthening. Without proper actions aimed at strengthening the existing HIS and improving the use of data produced by HIS, health systems may never fully be able to meet the needs of the populations they serve, and countries will fail to achieve the SDGs by 2030 [20].

This project seeks to strengthen the HIS in two low- and middle-income countries (LMICs) in the MENA region—specifically, Lebanon and Jordan. In this paper, we present the findings from the research conducted in Jordan.

### Jordanian context

Jordan is an upper middle-level income country with a total area of 89 342 square kilometers divided into 12 governorates. The population size reached 10.5 million in 2019 [21]. The country has a constitutional monarchy with a Prime Minister as head of Government appointed by His Majesty the King [21]. The Higher Health Council is the authorized entity to develop health policies and prepare national strategies for the health system.

Jordan’s health care system is a complex amalgam of three major sectors: public, private, and donors. It is relatively fragmented (between the public and private sectors and also within the public sector) and hospital-centric [21]. The main health care provider is the public sector, consisting of three providers: Ministry of Health (MOH), the Military Royal Medical Services (RMSs) and two university-affiliated hospitals (UHs) as well as the Centre for Diabetes, Endocrinology and Genetics. These organizations finance and deliver healthcare services to approximately 70% of the population, including civil service employees and members of the military as well as their dependents. The private sector includes private hospitals and diagnostic and therapeutic centers in addition to hundreds of private clinics. The international sector and charitable sectors provide services through UNRWA clinics for Palestinian refugees and the UNHCR and King Hussein Cancer Center and charity association clinic. [22–24].

The Health Information Centre, established in the MoH in 1992, is overseen by the Minister of Health. The directorate of Health Information, Studies and Research established within the MoH (later linked as ‘Department of Information Studies’ to the Institutional Development and Quality Control Directorate in 2019) collects basic health information from MoH facilities and other public and private hospitals. The Directorate produces the MoH annual report which is considered the main source of information about health services, including health human resources. Jordan’s HIS obtains data from different type of sources such as various institutions and population-based data sources, including [25]:

- MoH health centers and hospitals (all healthcare services).
- Royal Medical Services (RMS), university
- Hospitals, and private Hospitals.
- Department of Statistics information on health population indicators.
- Ministry of Health Directorates (communicable, non-communicable, occupational, school, environment, chest and migrant and organ plant directorates) through the annual periodic reports from central directorates in the Ministry of Health.
- Civil registration and vital statistics (births and deaths).
- Integrated Electronic Reporting System
- Jordan Cancer Registry Report (bi-annual reporting).
- Annual forensic report.
- Different activities of central directorates at the Ministry of Health (e.g. Licensing directorate).
- Financial data from the MoH (including supplies and medications).
- Annual Health Insurance financial reports.
- National Health accounts available from the Higher Health Council

While significant strides have been made in developing the HIS over the past decade, including the launching of Hakeem, a national e-health program, with the goal of revolutionizing Jordan's healthcare system [26], the need to strengthen the HIS has been reiterated in the National Strategy for Health Sector in Jordan [23]. Priorities identified by the MoH relate to reinforcing the HIS, including civil registration, risk factor and morbidity monitoring and health systems performance; and promoting evidence-based health policy development and effective utilization of knowledge management systems [27].

Existing studies within Jordan indicate that the HIS struggles with fragmentation and effective coordination and integration across different data sources. It has been noted that data from various sources are not well coordinated within the HIS framework, leading to overlapping data collection, gaps in available data, challenges in readiness, and resource wastage [28]. Additionally, the HIS lacks effective linkages and integration with routine information from various departments within the MoH and other ministries, such as the Civil Registration and Vital Statistics [28]. This complexity is compounded by outdated population survey tools, incomplete survey modules, and underutilized institutional data capabilities, which hinder efficient data utilization and impact the formulation of data-driven national health policies [28].

Within the realm of maternal, child and adolescent health, there is still a pressing need to integrate family planning services into primary health care, adopt preconception care within the public maternal and reproductive health services, and strengthen the maternal mortality surveillance and response system [8]. Additionally, Jordan faces challenges in adolescent health due to the lack of standardized data and indicators, as the HIS is not well-equipped to capture this information [27].

Furthermore, Jordan is among the countries most severely impacted by the Syrian refugee crisis, hosting the world's second-highest proportion of refugees relative to its population [29, 30]. However, the country's HIS is still not well-designed to support disaggregation by refugee status, thus making it difficult to ascertain coverage rates of key health indicators for refugees [31]. This need for data to be disaggregated by refugee status represents an important area for improvement of the country’s HIS.

A recent assessment highlighted that while Jordan’s HIS does support tracking of progress towards social policies and actions, it falls short in effectively addressing health inequities [32]. The system includes measures of some social determinants of health but lacks a comprehensive database and necessary integration to effectively measure and address health disparities [32]. Additional challenges faced by Jordan’s HIS include constrained financial resources, a lack of regulatory and policy support specifically tailored to health information systems, and inadequate standardization in the methods of data collection, processing, and analysis, which limits the utility of the data for research purposes [33, 34]. There is also a notable deficiency in skilled IT personnel capable of managing and advancing HIS capabilities [33, 34].

In light of these issues, there is a need to strengthen the existing HIS and promote the collection of relevant, reliable and comprehensive data and indicators to allow for improved health planning and decision-making in Jordan.

### Aim and objectives

The overall aim of this study is to promote data-informed decision-making in Jordan, by strengthening the existing HIS and ensuring the right sets of indicators are in place for generating relevant, reliable and timely data to effectively inform decisions and support health systems strengthening. In line with the SDG’s commitment to “leaving no one behind” and the articulation of the importance of addressing vulnerabilities on a global scale, specific efforts will be invested to optimize indicators related to maternal, child and adolescent health as well as refugee health-related indicators.

This study seeks to answer the following question: How can the current HIS in Jordan be strengthened to capture quality, relevant and standardized data with a focus on vulnerable populations?

The specific objectives are to:

- Conduct a baseline assessment of the existing national HIS structures and capacities, including those of staffs, directors and heads of departments within the MoH directorates
- Achieve consensus on a core set of health system indicators that are relevant and useful for health decision-making as well as optimize indicators for vulnerable populations including mothers, children, adolescents and refugees
- Standardize data collection process for the core set of selected HIS indicators

To the best of our knowledge, this is the first experience from the MENA region to report on the selection, development and validation of standardized health system indicators for informing health decision-making and strengthening health systems.

### Methods

A multifaceted stepwise approach was adopted, encompassing the following steps:

- Baseline assessment of the health information system
- Compilation of a comprehensive list of candidate indicators
- Consensus meetings to prioritize and validate indicators
- Development of procedure manual for standardizing the shortlisted indicators

A local liaison team was established in Jordan to coordinate stakeholder engagement and co-lead implementation of the project in the country. Data collection spanned from 2019 until 2022.

## Data collection

- Assessment of health information system

A tools manual was developed based on the Performance of Routine Information System Management conceptual framework. This framework adopts a multifaceted approach to evaluate routine health information system performance by examining the causal pathways between the organizational, technical and behavioral determinants of performance. The tools manual included the following sub-tools:

- Sub-tool 1: RHIS Mapping Tool
- Sub-tool 2: RHIS Profile Overview
- Sub-tool 3: RHIS Performance Diagnostic Tool
- Sub-tool 4: RHIS Organizational and Behavioral Assessment Tool
- Sub-tool 5: RHIS Management Assessment Tool

The tools manual was pilot-tested in collaboration with the local liaison team as part of a one-day workshop in December 2019. Following the pilot-testing, the local liaison team coordinated the administration of the tools manual with key selected stakeholders in Jordan. Data collection for the different sections of the tools manual involved a mix of interviews, focused group discussions and self-administered surveys. Where applicable, participants were asked to provide documentations to supplement and cross-reference their responses. Participants were selected based on their relevance to the different sections of the tools manual. An overview of the target audience and process of administration of the different sub-tools within the tools manual is presented in Table 1.

**Table 1:** Target audience and process of administration of the different sub-tools within the tools manual

Tools manual	Target audience	Process of administration
Sub-tool 1: RHIS Mapping Tool	Core team members of the Information Studies Department at the MoH (7 staff members)	Focused group discussions
Sub-tool 2: RHIS Profile Overview	Senior leads including the Head of the information technology department at the MOH	Structured interviews (participants were selected responded to different sections of the interview guide and responses were collated)
Sub-tool 3: RHIS Performance Diagnostic Tool	Core team members of the Information Studies Department at the MoH (7 staff members)	Focused group discussions
Sub-tool 4: RHIS Organizational and Behavioral Assessment Tool	Directors and heads of departments of the different MOH directorates (26 purposively selected individuals)	Self-administered surveys
Sub-tool 5: RHIS Management Assessment Tool	Senior leads including the Head of the Information Studies Department at the MOH	Structured interview (participants were selected responded to different sections of the interview guide and responses were collated)

b Compilation of a comprehensive list of candidate indicators (i.e. initial selection of indicators)

This step drew on a review of international health system indicators, and an inventory of health indicators reported in Jordan.

We conducted an extensive literature review of international health and health system indicators to select candidate dimensions and indicators for inclusion in the national HIS of Jordan. We ran broad and specific searches corresponding to core health systems; maternal, child and adolescent health; and refugee health-related indicators. We searched both published and grey literature. For the published literature, we searched PubMed and Google Scholar using the following keywords: (health system OR maternal OR child OR adolescent OR refugee) AND (indicator* OR data OR reporting system). For the grey literature, we searched intergovernmental websites and reports. A brief overview of the different sources is provided below, by thematic areas:

- *Core health and health systems indicators:* A comprehensive list of health and health systems indicators was compiled by reviewing existing indicator sets from the published literature and leading organizations such as the World Health Organization (WHO), Centers for Disease Prevention and Control (CDC), United Nations Statistics Division, United Nations Children's Fund (UNICEF), Pan American Health Organization (PAHO), Organization for Economic Co-operation and Development (OECD), and World Bank [27, 35–45]. The following two sources were used as starting points for indicator compilation: 1) the WHO Global Reference List of 100 Core Health Indicators (plus health-related SDGs) which includes all SDG 3 indicators [40] and; 2) the core indicators for monitoring health and health system performance in the Eastern Mediterranean Region [27]. These were then supplemented by additional indicators identified from the other organizations and the published literature.
- *Maternal, child and adolescent health indicators:* We adapted the Health Equity Assessment Toolkit (HEAT) developed by the WHO which contains the WHO Health Equity Monitor database. This database allows users to assess in-country inequalities against over 30 Reproductive, Maternal, Newborn and Child Health (RMNCH) indicators and six dimensions of inequality (economic status, education, place of residence, subnational region as well as age and sex (where applicable) [40]. This was supplemented by indicator lists from UNICEF [46], and two scoping reviews on maternal and child health [47, 48]. As for adolescent health, we utilized the list of 32 indicators generated during the WHO Technical consultation meeting on indicators of adolescent health as a starting point for prioritization efforts. These indicators have been chosen on the basis of four criteria: public health relevance, validity of constructs, feasibility of measurement and possibility of detecting change over time [49]*.* The indicators were complemented by additional indicators proposed by other sources.
- *Refugee health-related indicators:* Data from United Nations High Commissioner for Refugees (UNHCR) [50] health information system was supplemented by additional indicators from UNRWA and the published literature on refugee health.

For the Jordanian indicator inventory, we conducted a comprehensive mapping exercise to pinpoint the health system indicators presently captured by the national HIS in Jordan. Data sources included official reports from the MOH and Department of Statistics, annual statistical reports such as Population and Family Health Surveys, and national health accounts. Following this, the compiled indicator inventory underwent scrutiny and validation by representatives from the MoH.

We cross-checked, refined and consolidated the indicator lists derived from the international document review with the indicator inventory specific to Jordan. The consolidated candidate indicators were subsequently categorized under three priority thematic areas, namely: Core health and health system indicators; maternal, child, and adolescent health indicators; and refugee health indicators. Within each thematic area, we applied the WHO conceptual framework [27] to further stratify the indicators into the following domains: health status, risk factors, service coverage, and health system performance.

c Consensus meetings to prioritize and validate the indicators

Three consensus meetings were held with policymakers and stakeholders in Jordan between July 2019 and February 2022 to prioritize and shortlist the initially consolidated indicators and subsequently validate the shortlisted indicators within each thematic area (core health and health systems; maternal, child and adolescent health; and refugee health). In the first meeting, participants prioritized and shortlisted the “core health and health systems” and the “maternal, child and adolescent health” indicators. In the second meeting, participants validated the shortlisted indicators from the first meeting. In the third meeting, participants prioritized and shortlisted the “refugee health” indicators followed by subsequent validation of the shortlisted indicators.

A two-round modified Delphi technique was employed to derive a final set of indicators (within each thematic area) for inclusion in the national HIS for Jordan. For the prioritization and shortlisting of candidate indicators, participants individually ranked each candidate indicator against the following 3 criteria: Important; Feasible and Actionable (see Table 2). These criteria were adapted from a study by Al-Katheeri el at 2018 [51] and revised based on stakeholder consultations in Jordan. For the validation of the shortlisted indicators, participants reviewed each indicator and indicated whether it should be retained. For an indicator to be retained, it should meet a list of criteria, as depicted in Table 2. Following the validation of indicators, participants were asked to select a subset of those indicators for visualization and regular reporting (see Table 2). For both the prioritization and validation phases, participants had the opportunity to list any additional indicators or suggest modifications to the wordings of the existing indicators.

**Table 2:** Criteria used for prioritization and validation of indicators

Criteria	Definitions	Scoring
Prioritization/shortlisting criteria		
Important	● The indicator reflects an issue that is important to the general population and relevant stakeholders in the health system; or ● The indicator represents the most critical issues and priorities of the health systems; or ● The indicator is important for policy and regulation; or ● The indicator can help inform health system goals and strategies	Yes or No
Feasible	The indicator is easy to measure in terms of: ● Data availability; or ● Minimal burden of data collection; or ● Minimal costs of data collection	Yes or No
Actionable	● The indicator can help identify opportunities for health system improvement and galvanize action at the national, state, local or community level; or ● The indicator provides information that is appropriate and useful for guiding policies and programs as well as for decision-making; or ● The indicator measures an aspect that is subject to control by the health system and is actually used at a national level for policy making, monitoring or strategy development	High or Low
Validation criteria		
Retain indicator	Rate your agreement on whether the indicator should be retained in the prioritized list of indicators, based on the following: ● The indicator is important, relevant and acceptable to stakeholders ● The indicator assesses an important leverage point for improvement ● The indicator is worth measuring: it represents an important and salient aspect of the public's health ● The indicator is well defined and standardized ● The methodology of the indicator is well documented and readily available ● The indicator provokes change (for example in policies, services or lifestyles) ● The indicator can lead to set of targets or thresholds	Agree or Disagree
Require data visualization	● Benchmarking and visualization can support decision making on national priority areas ● Can provide tangible information needed for policymakers to develop national strategies and initiatives ● Can support departments in our ministry/department in responding to questions/objectives of ongoing projects ● Real time information is needed to support decision making related to this indicator	Yes or No

Each consensus meeting involved a diverse group of 12–15 individuals purposively selected based on the below sampling framework:

Senior and middle-level policymakers from the public sector such as officials from the MoH and related ministries

- Representatives from High Health Council in Jordan
- Representatives from professional associations
- Healthcare directors, managers and professionals
- Representatives of NGOs, UN agencies and other non-state sector who are involved in refugee work or collect data related to refugee
- Researchers and academics who are active in the realm of health systems research
- Other stakeholders who use data from the national health information system to inform their decisions

The sampling also took into consideration gender balance.

The meetings were informed and guided by three prioritization and validation tools, corresponding to the core health and health systems indicators; maternal, child and adolescent indicators; and refugee health indicators, respectively (see Supplementary file 1). Each tool was filled on an individual basis by participants.

d Development of procedure manuals

Procedure manuals enable the standardization of all aspects of data handling from collection, quality-assurance and flow, to processing, compilation and analysis. This can provide guidance on key aspects including individuals responsible for data collection, type and methods of data collection, timing of data reporting, and data interpretation and use. When utilized in a systematic and coordinated fashion by all health partners, these resources help to ensure that data and indicators are comparable across settings and achieve compatible degrees of aggregation [52].

Procedure manuals were developed for the short-listed indicators. The development of the content for the procedure manuals was guided by the WHO global metadata registry and the Standards and Best Practices for Data Sources [3, 53]. The procedure manuals were validated through pre-testing to enable assessment of the technical feasibility of data gathering. Specifically, a small working group encompassing representatives from the MoH hospitals and primary care was established to validate the feasibility and applicability of the procedure manuals within the Jordanian context, including the available and preferred data sources for each indicator.

## Data analysis

For the baseline assessment of HIS structures and capacities, data generated from the different sources were collated and analyzed in aggregate form and categorized according to the components of the tools manual. Data triangulation helped provide a more in-depth understanding of the issue and increase the reliability and validity of findings through cross-checking of information across different data sources. For the development of action plan for improvement, we made use of both thematic and framework analysis. Specifically, we adapted the framework by MEASURE Evaluation for rapid assessment of routine HIS to categorize the emerging themes into four strategic areas for improvement: Governance and planning; Infrastructure and resources; Data management;and Institutional capacity building [54]. In addition to coding the findings under predefined themes within each strategic area, we also allowed for new themes to emerge inductively.

For the indicators, separate scores were generated for each thematic area: core health system, maternal, child and adolescent health, and refugee health. Shortlisting of indicators was done against three criteria: Important, Feasible and Applicable. Participants rated each indicator on a binary scale (Yes/No) for each criterion. Average scores were calculated for each criterion per domain. Indicators that scored above average for all three criteria were shortlisted for validation. The short-listed indicators were validated based on the criteria ‘retain’. Indicators scoring above average for retention criterion were included in the final list for the HIS. Participants were also asked to identify those shortlisted indicators with potential for visualization.

## Results

### Assessment of health information system

Findings from the baseline assessments revealed lack of well-functioning national HIS that can support health system strengthening. Data sets were often fragmented, patchy, and compartmentalized. Institutional structures, systems and culture were not conducive for MoH to operate as proactive knowledge management organizations. Existing personnel lacked the capacity to manage and advance HIS capabilities and promote data-driven decision-making.

Actions for improvements encompass four strategic areas which interact with each other to strengthen the HIS and facilitate better-informed decisions (see Table 3 and Supplementary file 2 for the detailed action plan):

- Governance and planning
- Infrastructure and resources
- Data management
- Institutional capacity building

**Table 3:** Strategic areas for improvement

Strategic area	Actions for improvement
Strategic Area I: Governance and planning	● Update and implement health information system policies, laws and regulations ● Develop an explicit HIS strategy or plan and integrate it within the broader health sector strategy ● Improve national coordination & planning of health information management to have a harmonized data system
Strategic Area II: Infrastructure and resources	● Ensure adequate human resources are available for health information system ● Ensure adequate financial resources are dedicated for health information system ● Ensure availability of adequate information hardware and software ● Establish and update databases for health facilities’ infrastructure and resources
Strategic Area III: Data management	● Develop, revise, and update the list of indicators (health systems, performance, quality, etc.) reported on and used by each department at ministry of health (MoH) ● Establish a National Health Indicator Compendium for national level programs and priorities, harmonized with international standards and reporting requirements ● Develop procedures manuals to standardize data collection and reporting ● Strengthen existing data sources (population census, individual records, etc.) ● Optimize data flow within MoH as well as between MoH and health facilities and external stakeholders ● Improve data analysis at MoH and at level of districts and health facilities ● Implement improved data quality assurance and monitoring mechanisms at MoH and at level of districts and health facilities
Strategic Area IV: Institutional capacity to expand use of information to support evidence-informed decision-making	● Build health information capacity of MoH staff and of healthcare professionals ● Conduct periodic assessment of Information needs ● Develop an Information Products Plan to meet specific needs of different data and information users ● Establish mechanisms to increase access to health information and analysis tools ● Strengthen data and Information dissemination mechanisms ● Enhance accountability & transparency of decision-making processes

### Compilation of a comprehensive list of candidate indicators

As indicated earlier, this step drew on a review of international health system indicators, and an inventory of health indicators reported in Jordan.

**Fig. 1:**
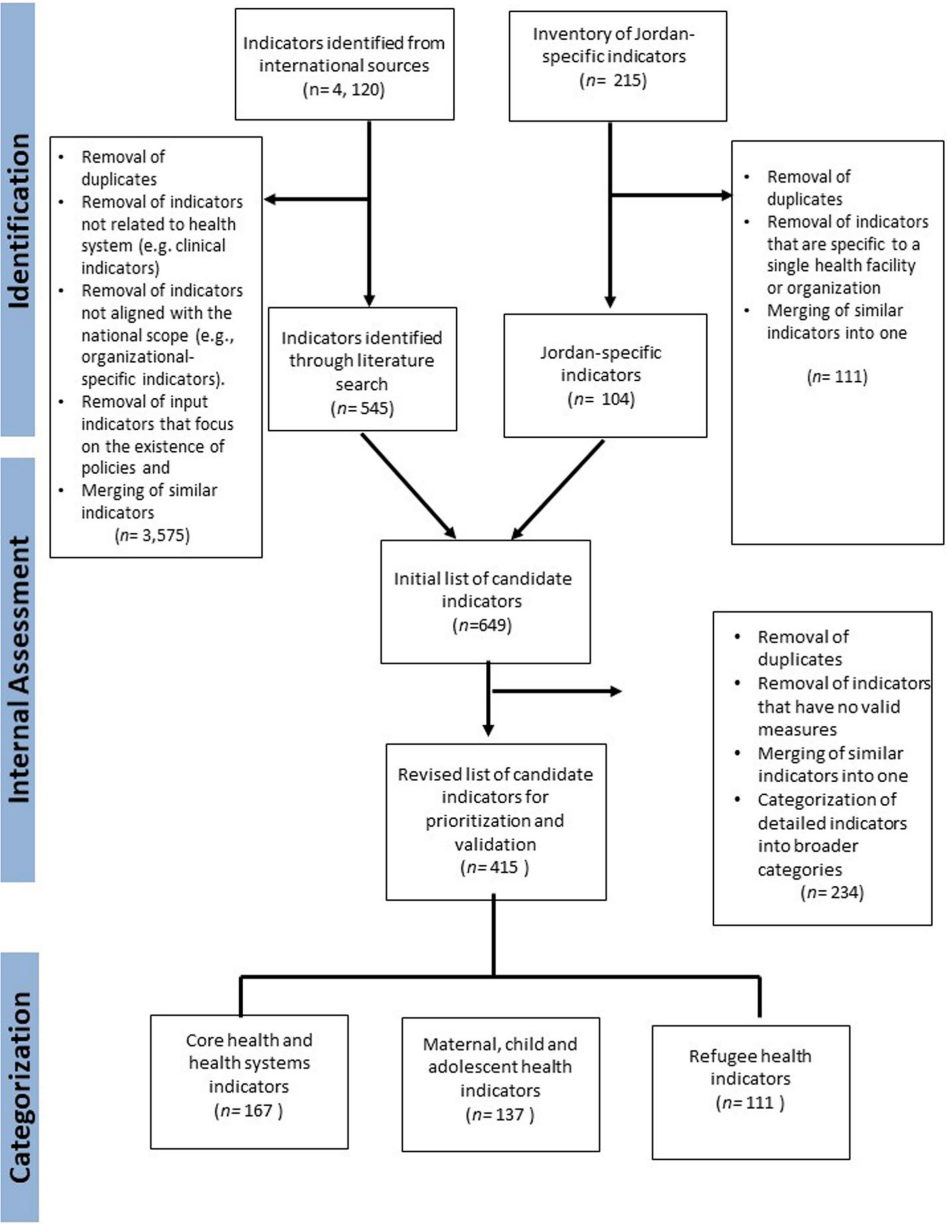
Flowchart detailing the process of indicator selection

The review of international sources retrieved an initial list of 4,120 indicators (Fig. 1). A first round of assessment by the research team refined the list by removing duplicates and eliminating indicators that are not related to health systems (e.g., clinical indicators) or are not aligned with the national scope (e.g., facility/organizational-specific indicators). Furthermore, we excluded input indicators that focus on the existence of policies and strategies (e.g., policies on breastfeeding) and merged similar indicators into one. This process resulted in 545 potentially relevant indicators from international sources (Fig. 1).

Additionally, 215 indicators were initially compiled from the different national sources within Jordan. Supplementary file 3 provides an overview and evolution of indicators from Jordan’s national HIS. The research team collaborated with the local liaison team in Jordan to review the list, removing duplicates (n = 4), filtering out indicators that are specific to a single health facility or organization (n = 28), and merging similar indicators into one (n = 79). This process resulted in a refined list of 104 indicators already reported in Jordan.

The refined lists of indicators from Jordan (n = 104) and international sources (n = 545) were combined to create the initial draft of the Priority Setting Tool. This draft tool contained 649 indicators. In a second round of assessment, we collaborated with the local liaison team in Jordan and an expert from the MoH to further remove duplicates, merge similar indicators into one, discard those without valid measurement methods, and categorize detailed indicators into broader categories. This resulted in the selection of 415 candidate indicators for subsequent prioritization and validation by key policymakers and stakeholders in Jordan. Of these, 84 were already being reported within Jordan’s HIS. In other words, 80.7% of the identified indicators for Jordan were integrated into the tool. The remaining 331 indicators comprising the tool were newly identified from international sources.

The 415 indicators were subsequently categorized under three thematic areas (corresponding to three priority setting tools), namely: core health and health systems indicators (n = 167); maternal, child and adolescent health indicators (n = 137); and refugee health-related indicators (n = 111). Within each thematic area, the indicators were further stratified into the following four domains: health status, risk factor indicators, service coverage indicators and health system indicators.

### Prioritization and validation of the indicators

Fifteen participants took part in the first, 14 in the second, and 10 participants in the third consensus meeting. Each meeting included a diverse mix of participants from the High Health Council, the Jordanian Ministry of Health, Jordanian Royal Medical Services (RMS), Syndicate of Physicians, Syndicate of Nurses, Private Hospitals Association, and the Association of Public Health, WHO-Jordan and UNHCR Jordan.

In the initial round of prioritization, participants independently assessed the indicators under each thematic area. Utilizing a criterion-based ranking system, they rated each indicator against 3 criteria: Importance, Feasibility, and Actionability. This led to the preliminary selection of 55 core health systems indicators; 57 maternal, child and adolescent health indicators; and 33 refugee health indicators. In the second round, these shortlisted indicators were validated against the criterion ‘retain’. This resulted in a final validated set of indicators, comprising of 55 core health and health systems indicators, of which 33 (60%) were already reported in Jordan; 40 maternal, child and adolescent health indicators, with 21 (52.5%) already reported in Jordan; and 26 refugee health indicators, none of which were being reported in Jordan. Participants also suggested indicators to be added to each of the three thematic areas.

Of the indicators initially being reported in Jordan and included in the prioritization tools, 35 (41.6%) were subsequently retained as part of this exercise (Supplementary file 3).

The detailed findings are presented below, by thematic area.

i Core health and health systems indicators

The initial list comprised 167 core health system indicators. At the time of data collection, 58 of these indicators, representing 33% of the total, were being reported within Jordan's national HIS. Respondents rated each indicator against three criteria: importance, feasibility, and applicability. Fifty-five indicators scored above the average for all criteria and were subsequently shortlisted for validation. Table 4 summarizes the average scores per criterion and per domain.

**Table 4:** Summary of average scores per criteria and domain (prioritization)

Criteria	Average Score
Applicability	81.43%
Feasibility	65.76%
Importance	89.53%
Included Indicators	*55*

The 55 shortlisted indicators were subsequently validated based on average agreement per domain on the ‘retention’ criterion (Table 5). This resulted in a final validated set of 55 core health systems indicators (10 in dimension 1, 9 in dimension 2, 3 in dimension 3, and 32 in dimension 4). Among these 55 retained indicators, 33 (60%) were already being reported within Jordan's national HIS. Moreover, 17 indicators were identified as suitable for visualization. Participants also suggested additional indicators for inclusion.

**Table 5:** Summary of average scores per criteria and domain (validation)

Domains	Average for Retain	Average for Visualization	Total included indicators	Indicators to be visualized	Indicators being reported as part of national HIS
Domain 1: Health status	81.7%	53.2%	10	6	7
Domain 2: Risk factors	85.1%	56.3%	9	5	9
Domain 3: Service coverage	89.6%	65.6%	4	3	2
Domain 4: Health system performance	91.5%	57.1%	32	3	15
Total	88.5%	56.9%	**55**	**17**	33

Table 6 provides an overview of the final shortlisted and validated set of core health and health system indicators, including those already being reported within Jordan's national HIS at the time of data collection and those identified as suitable for visualization. Participants individually suggested 17 additional indicators for inclusion in the final set. These indicators were subsequently refined by the research team, based on the literature (Table 7).

**Table 6:** Final shortlisted and validated set of core health and health systems indicators

Domains	Included Indicators	Visualization (Yes/No)	Reporting Status in Jordan’s National HIS (Yes/No)
Domain 1: Health Status	1. Adult mortality rate (15–60 years)	Yes	Yes
	2. Premature NCD mortality disaggregated by type and sex [SDG 3.4.1]	No	No
	3. TB mortality rate	No	Yes
	4. Mortality rate from road traffic injuries [SDG 3.6.1]	Yes	Yes
	5. New cases of vaccine-preventable disease	Yes	No
	6. HIV incidence rate [SDG 3.3.1]	No	Yes
	7. Cancer incidence by type of cancer	Yes	Yes
	8. Prevalence of diabetes in adults	Yes	Yes
	9. Prevalence of end-stage kidney disease in adults	Yes	Yes
	10. Prevalence of severe visual impairment and blindness (0 to 14 years, 15 to 49 years and 50 years and older, non-disaggregated by gender)	No	No
Domain 2: Risk Factors	11. Prevalence of hypertension among adults	Yes	Yes
	12. Prevalence of raised cholesterol among adults	Yes	Yes
	13. Prevalence of overweight and obesity in adults and adolescents disaggregated by age and sex [gender sensitive] [SDG 2.2.2]	Yes	Yes
	14. Population size	Yes	Yes
	15. Sex ratio at birth (male births per female births)	No	Yes
	16. Population growth rate	No	Yes
	17. Educational attainment by level, age and sex	Yes	Yes
	18. Human development index	No	Yes
	19. Rank in elderly health on Global Watch Index	No	Yes
Domain 3: Service Coverage	20. Immunization coverage rate by vaccine for each vaccine in the national schedule [SDG 3.b.1]	Yes	Yes
	21. Antiretroviral therapy (ART) coverage	No	Yes
	22. Coverage of essential health services [SDG 3.8.1]	Yes	No
	23. % of health centers providing immunization	Yes	No
Domain 4: Health System Performance	24. Number of healthcare institutions with accreditation by type of institution	Yes	Yes
	25. Number of hospitals that have ambulance and emergency specialist at their disposal	No	Yes
	26. Hospital-standardized mortality ratio	Yes	No
	27. Prevalence and incidence of hospital-acquired infections (% of patients hospitalized)	Yes	Yes
	28. Five-year survival rates for breast, cervix and colon cancer	No	Yes
	29. % diabetics aged 18–75 years who received hemoglobin A1c test in primary health care	No	No
	30. % hypertensive patients with BP 140/90 or less in primary healthcare	No	No
	31. Staff satisfaction rate	Yes	No
	32. Patient/customer experience/satisfaction with care	Yes	No
	33. Number of emergency department visit per year	No	No
	34. Hospital inpatient admissions per 100 000 population per year	No	No
	35. Number, type and distribution of health facilities per 10,000 population	No	No
	36. Hospital bed density and distribution per 10,000 population	No	Yes
	37. Bed occupancy rate, acute care hospitals only	No	No
	38. Access to acute care	No	No
	39. Proportion of health facilities that have a core set of relevant essential medicines available and affordable on a sustainable basis [SDG 3.b.3]	No	No
	40. Hospital day cases as a percentage of total patient population	Yes	No
	41. % of population served by 24-h ambulance services	No	No
	42. % of referrals from primary health care to hospitals	No	No
	43. Number of health workers per 10, 000 population by type of health worker [SDG 3.c.1]	Yes	Yes
	44. Distribution of health workers by occupation/specialization, region, level of care, place of work and sex [SDG 3.c.1]	Yes	No
	45. Registered recent graduates of health profession educational institutions per 100, 000 pop	Yes	No
	46. Birth registration [SDG 16.9.1]	Yes	Yes
	47. Death registration [SDG 17.19.2]	Yes	Yes
	48. GDP growth (annual %)	Yes	Yes
	49. National health expenditure as % of GDP	Yes	Yes
	50. Total current expenditure on health as % of gross domestic product	Yes	Yes
	51. Total capital expenditure on health as % of current + capital expenditure on health	No	No
	52. Public domestic sources of current spending on health as % of current health expenditure	No	Yes
	53. % of gov health spending allocated to PHC	Yes	Yes
	54. Total expenditure on pharmaceuticals (% total expenditure on health)	Yes	Yes
	55. Gov expenditure on pharmaceuticals (per capita average exchange rate)	No	Yes
Total (Yes)		N = 30 (54.5%)	N = 33 (60%)

**Table 7:** Additional suggested core health systems indicators and their refinement

Suggested indicators	Refined indicators	Included in the tools but not retained	Being reported in Jordan
1. Indicators related to sexually transmitted infections in general	Prevalence of specific sexually-transmitted infection (e.g., Chlamydia, Gonorrhea, Syphilis) in the population disaggregated by age group, gender, and risk factors	Yes	No
2. Suicide prevalence indicators	Age-standardized suicide rate per 100,000 population, including suicide attempts and methods used	Yes	No
3. Indicators for measuring triglycerides and cholesterol	Average serum levels of triglycerides and cholesterol disaggregated by age group, gender, and risk factors	No	No
4. Prevalence of smoking, alcohol consumption and drug usage	Prevalence of smoking, alcohol consumption, and drug use among individuals disaggregated by age group, gender, and type of substance	Yes	No
5. Treatment for substance abuse	Proportion of individuals with substance use disorders who receive treatment (e.g., medication-assisted therapy, behavioral therapy)	Yes	No
6. Antibiotic resistance (antibiogram)	Antibiogram data showing the prevalence of antibiotic resistance among common bacterial pathogens	No	No
7. Medication errors	Rate of medication errors reported in healthcare settings	Yes	No
8. Mortality from cardiovascular and pulmonary diseases	Age-standardized mortality rates from cardiovascular diseases (e.g., heart disease, stroke) and pulmonary diseases (e.g., pneumonia, chronic obstructive pulmonary disease)	No	No
9. Percentage of preventive complications of hypertension and Diabetes Mellitus	Proportion of individuals with hypertension and diabetes who experience complications related to lack of preventive care (e.g., retinopathy, nephropathy, amputations)	No	No
10. Dialysis cases (number and percentage)	Number and percentage of individuals undergoing dialysis treatment for kidney failure	No	No
11. Out of pocket expenditure (* being measured in Jordan)	Average out-of-pocket expenditure on healthcare per capita	Yes	Yes
12. Mismanagement cases (and resulting deaths)	Number and percentage of cases involving medical mismanagement and resulting deaths	No	No
13. Expenditure by scheme	Total health expenditure (public and private) as a percentage of Gross Domestic Product (GDP)	Yes	Yes
14. % of catastrophic health expenditure	Proportion of catastrophic health expenditure	No	No
15. Current health expenditure (public/private)	Current government expenditure on health (as % of total expenditure)	Yes	Yes
16. CHE per capita	Current Health Expenditure (CHE) per capita	No	No
17. % impoverishment (FRPI)	Percentage of impoverishment due to health expenditure (Financing Risk Protection Index—FRPI)	No	No

ii Maternal, child and adolescent health indicators

The initial list consisted of 137 indicators (100 maternal and child health, and 37 adolescent health indicators). At the time of data collection, 41 of these indicators (36 for maternal and child health, and 5 for adolescent health), collectively representing 30% of the total indicators considered for prioritization, were being reported within Jordan's national HIS.

Participants rated each indicator against three criteria: importance, feasibility, and applicability. Average scores were calculated for each criterion, stratified by indicator type (maternal and child vs. adolescent health) (see Table 7). Forty-one maternal and child health indicators (dimension 1 (n = 13), dimension 2 (n = 18), dimension 3 (n = 18) and dimension 4 (n = 9)) and 16 adolescent health indicators (dimension 1 (n = 11), dimension 2 (n = 3), dimension 3 (n = 2), and dimension 4 (n = 10)) scored above average for all of the criteria and were subsequently shortlisted for validation. Table 8 summarizes the results.

**Table 8:** Summary of average scores per criterion and domain (prioritization)

Criteria	Adolescents indicators	Maternal and child health indicators	Total
Applicability	62.89%	87.07%	
Feasibility	43.01%	73.47%	
Importance	78.12%	94.62%	
Not Applicable	9.20%	10.26%	
Included indicators	16	41	57

The shortlisted indicators were subsequently validated based on average agreement per domain on the ‘retention’ criterion (Table 9). The final set included 40 indicators: 32 maternal and child health indicators and 8 adolescent health indicators. Among these 40 retained indicators, 21 (52.5%) were being reported by Jordan's national HIS (19 for maternal and child health, and 2 for adolescent health) at the time of data collection. Moreover, 25 indicators were identified as having potential for visualization. Participants also suggested additional indicators for inclusion in the final set.

**Table 9:** Summary of average scores per criteria (validation)

Domains	Average for Retain	Average for Visualization	Included maternal and child health indicators	Included adolescent health indicators	Total included indicators	Indicators to be visualized	Indicators being reported as part of national HIS
Domain 1: Health status	77%	32%	13	7	20	8	10
Domain 2: Risk factors	87%	29%	1	1	2	2	1
Domain 3: Service coverage	83%	38%	12	0	12	9	7
Domain 4: Health system performance	74%	30%	6	0	6	6	3
Total	80.3%	32.3%	32	8	40	25	21

Table 10 provides an overview of the final set of shortlisted and validated maternal, child and adolescent health indicators, including those already being reported within Jordan's national HIS at the time of data collection and those identified as suitable for visualization. Participants also individually suggested 19 additional indicators for inclusion in the final list. These indicators were subsequently refined by the research team, based on the literature (Table 11).

**Table 10:** Final shortlisted and validated set of maternal, child and adolescent health indicators

Domains	Included Indicators	Visualization (Yes/No)	Reporting Status in Jordan’s National HIS (Yes/No)
Domain 1: Health status	1. Maternal mortality ratio (per 100 000 live births) [SDG 3.1.1] [gender-sensitive]	Yes	Yes
	2. Under-five mortality rate; boys and girls [SDG 3.2.1] [gender sensitive]	No	Yes
	3. Infant mortality rate; boys and girls [gender sensitive]	No	Yes
	4. Neonatal mortality rate (per 1000 live births) [SDG 3.2]	No	Yes
	5. Distribution of causes of death among children aged < 5 years (%)	Yes	Yes
	6. Incidence of low birth weight; boys and girls [gender sensitive]	Yes	Yes
	7. Children under 5 years who are stunted [SDG 2.2.1]	Yes	Yes
	8. Prevalence of anemia in children	Yes	Yes
	9. New cases of cancer per 100,000 children aged 0–14 years	Yes	No
	10. New cases of children aged 0–14 years receiving insulin on the National diabetes register as a rate per 100,000 children	No	No
	11. Proportion of children aged 0–14 years with disability	No	No
	12. Proportion of children with congenital anomalies (by type)	No	No
	13. Adolescent mortality rate	No	Yes
	14. Adolescent mortality rate from road traffic injuries	Yes	No
	15. Adolescent mortality rate from suicide	Yes	No
	16. Adolescent mortality rate from violence	No	No
	17. Adolescent maternal mortality ratio (per 100,000 live births)	No	No
	18. Percentage of births delivered by caesarean section	No	Yes
	19. Prevalence of suicide attempts among adolescents	No	No
	20. Prevalence of anemia among adolescents	No	No
Domain 2: Risk factors	21. Prevalence of anemia in women of reproductive age [gender sensitive]	Yes	Yes
	22. Prevalence of iron deficiency anemia in 10–24-year-old	Yes	No
Domain 3: Service coverage	23. Availability of basic essential obstetric and newborn care facilities per 500,000 population	No	No
	24. Availability of Emergency Obstetric and Newborn Care facilities per 500,000 population	No	No
	25. Percentage of postpartum clients receiving modern family planning method from health centers	No	Yes
	26. Referral rates for women with obstetric complications	Yes	Yes
	27. Demand for family planning satisfied with modern methods [SDG 3.7.1]	Yes	Yes
	28. Antenatal care (at least one visit) (%)	Yes	Yes
	29. Antenatal care (at least four visits) (%)	Yes	Yes
	30. Proportion of pregnant women receiving iron and folic acid supplements	Yes	No
	31. Percentage of newborns receiving essential newborn care	Yes	No
	32. Proportion of pregnant women with hypertension receiving antihypertensive drugs	Yes	No
	33. Percentage of mothers who received counselling, support or messages on optimal breastfeeding at least once in the last year	Yes	Yes
	34. Vitamin A supplementation coverage (Percent of children aged 6–59 months who received two age-appropriate doses of vitamin A in the past 12 months)	Yes	Yes
Domain 4: Health system performance	35. Proportion of women with severe pre-eclampsia or eclampsia treated with magnesium sulfate injection	Yes	No
	36. Proportion of women who developed severe post-partum hemorrhage (PPH)	Yes	Yes
	37. Facility neonatal mortality rate disaggregated by birth weight: > 4000 g, 2500–3999 g, 2000–2499 g, 1500–1999 g, < 1500 g	Yes	No
	38. Proportion of health facilities with safe, uninterrupted oxygen supply in childbirth, neonatal and pediatric wards	Yes	No
	39. Percentage and distribution of health workers trained to provide reproductive, maternal and child health	Yes	Yes
	40. Density of midwives, by district (by births)	Yes	Yes
Total (Yes)		N = 25 (62.5%)	N = 21(52.5%)

**Table 11:** Additional suggested maternal, child and adolescent health indicators and their refinement

Suggested indicators	Refined indicators	Included in the tools but not retained	Being reported in Jordan
1. % of maternal breastfeeding	Exclusive and continued breastfeeding rates for infants under six months and one year, respectively	Yes	Yes
2. Gestational Diabetes Mellitus	Prevalence of Gestational Diabetes Mellitus among pregnant women and proportion who receive appropriate management	No	No
3. Post-partum complication that need invasive procedures	Proportion of women experiencing post-partum complications requiring surgical interventions (e.g., caesarean section, hysterectomy)	No	No
4. Distribution of causes of adolescent mortality rate	Proportion of adolescent deaths aggregated by cause of death	Yes	No
5. Smoking among adolescents	Percentage of adolescents who report current smoking or drug use, disaggregated by age group and gender	Yes	No
6. School health	Availability and utilization of school health services, including access to healthcare professionals, vaccinations, and health education programs	No	No
7. Obesity among adolescents	Body Mass Index (BMI) for-age percentiles	No	No
8. Control of diabetes among adolescents	Proportion of adolescents diagnosed with diabetes receiving adequate treatment and achieving glycemic control	No	No
9. Vitamin D deficiency among adolescents	Prevalence of vitamin D and B12 deficiencies among children and adolescents	No	No
10. Drug use and addiction among adolescents by age and gender	Prevalence of drug use and addiction among individuals disaggregated by age group and gender	Yes	No
11. Age of giving birth (by gender)	Mean age and distribution of first birth by gender	No	No
12. % of abortion by age	Proportion of abortions performed among women disaggregated by age group	No	No
13. School drop-outs	Percentage of adolescents who drop out of school before completing secondary education	No	No
14. % of teens/adolescent’s pregnancies	Teenage birth rate per 1,000 females aged 15–19	Yes	Yes
15. Mental health status among adolescents	Percentage of adolescents reporting symptoms of depression or anxiety	No	No
16. Adolescents sexually transmitted diseases	Incidence rate of common sexually transmitted diseases (e.g., chlamydia, gonorrhea) among adolescents	No	No
17. Adolescents diabetes	Prevalence of diabetes among adolescents (Type 1 and Type 2)	No	No
18. Adolescents nutrition	Percentage of adolescents meeting recommended daily fruit and vegetable intake	No	No
19. Adolescent dental health	Percentage of adolescents with regular dental check-ups	No	No

iii Refugee health indicators

The initial list contained 111 refugee health indicators. It is important to note that, at the time of data collection, none of these indicators were reported by the national HIS. However, 60 indicators were tracked by International Non-Governmental Organizations (INGOs) including entities like the United Nations High Commissioner for Refugees (UNHCR). Respondents rated these indicators against three criteria: importance, feasibility, and applicability. Thirty-three indicators scored above average for all of the criteria and were subsequently shortlisted for validation (Table 12).

**Table 12:** Summary of average scores per criteria (prioritization)

Domains	Important	Feasible	Actionable	Total Included indicators
Domain 1: Health status	95.6%	82.2%	83.1%	8
Domain 2: Risk factors	89.6%	57.7%	72.4%	10
Domain 3: Service coverage	85.4%	73.4%	77.2%	12
Domain 4: Health system performance	88.9%	75.6%	74.1%	3
Total	89.4%	71.3%	76.8%	33

The 33 shortlisted indicators were subsequently validated based on average agreement per domain on the ‘retention’ criterion (Table 13). The final set included 26 refugee health indicators (domain 1 (n = 8), domain 2 (n = 8), domain 3 (n = 8), domain 4 (n = 2)). While none of these core indicators were reported by Jordan's national HIS, 16 were tracked by INGOs or other external agencies at the time of data collection. Additionally, 14 indicators were identified as having potential for visualization. Participants also suggested additional indicators for inclusion in the final set.

**Table 13:** Summary of average scores per criteria (validation)

Domains	Average for Retain	Average for Visualization	Total included indicators	Indicators to be visualized	Indicators being reported as part of national HIS
Domain 1: Health status	91%	58%	8	3	0
Domain 2: Risk factors	85%	54%	8	5	0
Domain 3: Service coverage	84%	54%	8	5	0
Domain 4: Health system performance	73%	44%	2	1	0
Total	83.3%	52.5%	26	14	0

Table 14 provides an overview of the final shortlisted and validated set of refugee health indicators, including those suitable for visualization and those currently reported by Jordan’s national HIS. Participants also individually suggested 4 additional indicators for inclusion in the final list (Table 15).

**Table 14:** Final shortlisted and validated set of refugee health indicators

Domains	Included Indicators	Visualization (Yes/No)	Reporting Status in Jordan’s National HIS (Yes/No)
Domain 1: Health Status	1. Maternal mortality ratio (per 100,000 live births)*	Yes	No
	2. Under-five mortality rate; boys and girls	No	No
	3. Distribution of causes of death among children aged < 5 years (%)*	No	No
	4. Infant mortality rate; boys and girls *	No	No
	5. Neonatal mortality rate (per 1000 live births)*	No	No
	6. Perinatal mortality rate*	No	No
	7. Prevalence of diabetes in adults*	Yes	No
	8. Crude Birth Rate	Yes	No
Domain 2: Risk Factors	9. Number of children fully vaccinated	Yes	No
	10. Prevalence of overweight and obesity in adults disaggregated by age and sex *	No	No
	11. Percentage of ever-married women and men aged 15–49 who smoke various tobacco products (cigarettes, water pipe and any type of tobacco) *	No	No
	12. Prevalence of moderate to severe acute malnutrition in children under 5 years of age	Yes	No
	13. Percentage of women who use contraceptives *	Yes	No
	14. Proportion of population using safely managed drinking water services*	No	No
	15. Percentage of refugees aged 6 to 25 currently enrolled in formal education*	Yes	No
	16. Percentage of children under 5 whose births are registered and who had a birth certificate	Yes	No
Domain 3: Service Coverage	17. Breast cancer exam *	Yes	No
	18. Antenatal care (at least four visits) (%)*	No	No
	19. Percentage of newborns receiving essential newborn care *	No	No
	20. Percentage of women receiving Postpartum Care	No	No
	21. Obstetric referral rates, defined as the number of documented obstetric referrals by camp health staff to a referral hospital per 100 000 live births	Yes	No
	22. Immunization coverage rate by vaccine for each vaccine in the national schedule*	Yes	No
	23. Percentage of children age 24–35 months who ever had a vaccination card	Yes	No
	24. Percentage of refugee outside camp who received care from public health system	Yes	No
Domain 4: Health system Performance	25. Proportion of births attended by skilled health personnel *	Yes	No
	26. Percentage of refugees who are satisfied with public health services	No	No
Total (Yes)		N = 14 (53.8%)	N = 0 (0%)

^*^Denotes being reported by an external agency or INGO (e.g., UNHCR) at the time of data collection

**Table 15:** Additional suggested indicators for refugee health

Suggested indicators	Included in the tools but not retained	Being reported in Jordan
1. Proportion of refugees who reported accessing health services within a specified timeframe (e.g., past month) disaggregated by source (private, government, NGO, UN)	No	No
2. Percentage of refugees receiving vaccination (children under 5)	Yes	No
3. Proportion of refugees living outside camps who reported accessing services at MOH facilities and receiving the full scope of services they needed	No	No
4. Percentage of refugees diagnosed with Hypertension (HTN)	No	No

### Development of procedure manuals

Three procedure manuals were developed, corresponding to the three prioritized thematic areas: core health system indicators; maternal child and adolescent indicators; and refugee health indicators, respectively.

**Fig. 2:**
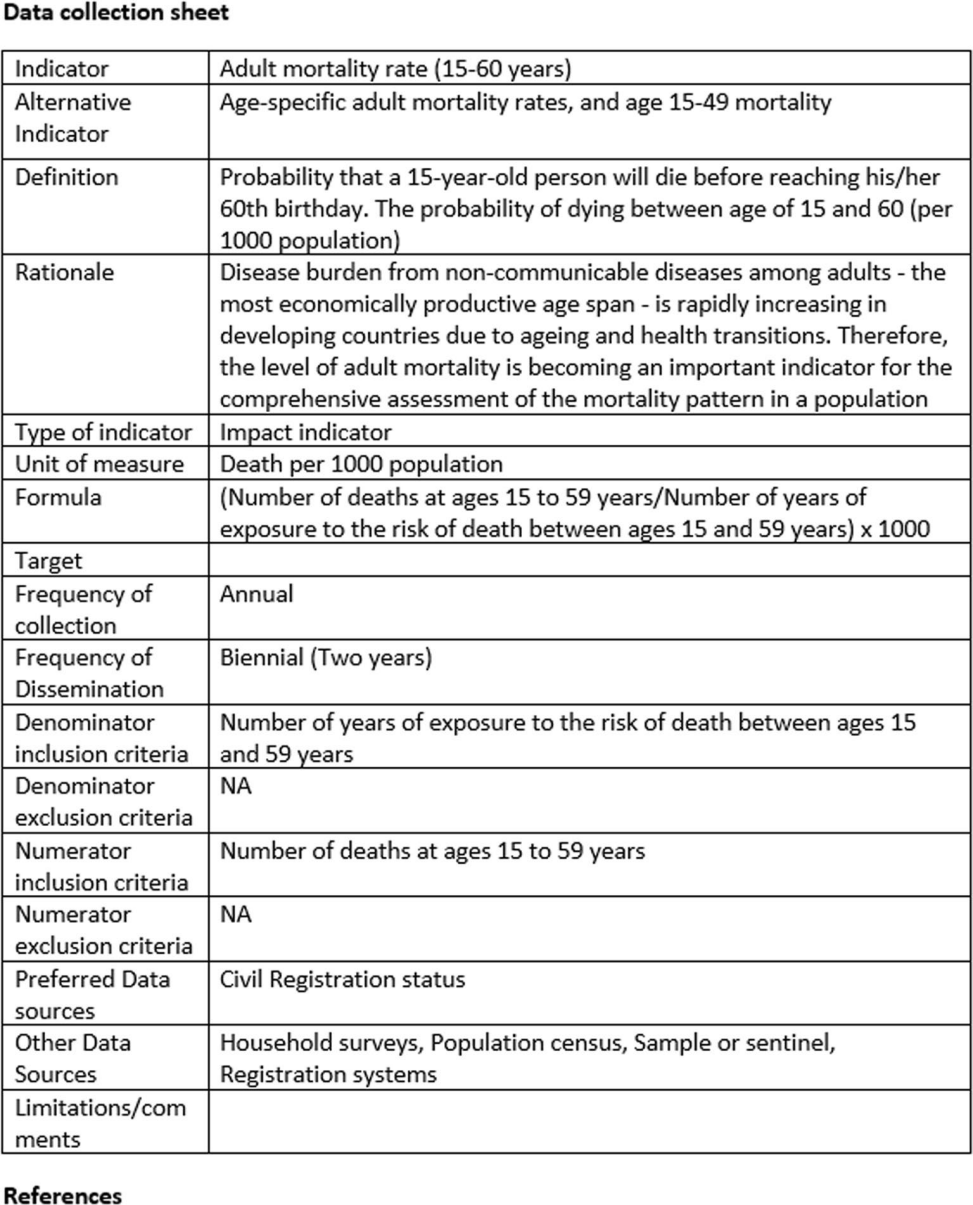
Example of data collection sheet for the indicator ‘mortality’

Each procedure manual provided the following information for each included indicator (see Fig. 2):

- Indicator
- Alternative indicator
- Definition
- Rationale
- Type of indicator
- Unit of measure
- Formula
- Target
- Frequency of collection
- Frequency of dissemination
- Denominator inclusion criteria
- Denominator exclusion criteria
- Numerator inclusion criteria
- Numerator exclusion criteria
- Preferred data sources
- Other data sources
- Limitations/comments
- References

The procedure manuals were validated through pre-testing to enable assessment of the technical feasibility of data gathering. The revised manuals were subsequently shared with key policymakers and stakeholders in Jordan. Supplementary file 4 provides a sample procedure manual for maternal, child and adolescent health.

## Discussion

To our knowledge, this is the first study from the MENA region to undertake a comprehensive assessment of the national HIS and achieve consensus on a core set of health and health systems indicators including for maternal, child adolescent and refugee health. The COVID-19 pandemic reaffirmed the criticality of a robust HIS with valid and reliable indicators for more effective and timely decision-making [14, 55]. Findings from this study can help foster a culture of sharing and using data by ensuring the right indicators, capacities and resources are in place to promote well-functioning HIS that can inform policymaking and practice.

The baseline assessments of the national HIS structure and capacities revealed gaps and highlighted areas for improvement at the following levels: governance and planning; infrastructure and resources; data management and institutional capacity. Strengthening these areas is crucial to support data-driven decision-making, which is vital for advancing public health outcomes and enhancing the efficiency of health systems in Jordan.

Importantly, through this study, we were able to achieve consensus on a standardized set of national health system indicators, which has significant implications for healthcare planning and resource allocation in Jordan. Of the 4,120 indicators reviewed from international sources and 215 from the indicators inventory in Jordan, 415 candidate indicators were compiled and categorized into three priority thematic areas: core health system indicators (n = 167), maternal, child and adolescent health indicators (n = 137), and refugee health indicators (n = 111). To ensure the selection of appropriate indicators and also increase the likelihood that indicators will have wider acceptance, we involved various stakeholders, including policymakers, health professionals, health researchers and representatives of non-governmental and intergovernmental organizations, in prioritizing, shortlisting and validating the indicators. This resulted in a final shortlisted and validated set of 121 indicators, comprising 55 core health systems indicators, of which 60% were already reported in Jordan; 40 maternal, child, and adolescent health indicators, with 52.5% reported in Jordan; and 26 refugee health indicators, none of which were reported in Jordan. A striking finding is that less than half (41.6%) of the indicators already reported in Jordan's national HIS and included in the priority setting tool were ultimately retained after the prioritization and validation exercises. This underscores the urgent need for revising and integrating new indicators into the national HIS to ensure it can provide a comprehensive and effective framework for ongoing health monitoring and data-driven decision-making. Participants also suggested indicators to be added to each of the three thematic areas. While several of these suggested indicators were included in the initial priority setting tools, they were not retained in the final selection. Consequently, these indicators are listed separately for future consideration, indicating potential areas for expanding the HIS to better meet the needs of the health system.

Another notable finding is the absence of refugee health indicators in the national HIS of Jordan, with most relevant data being managed by external agencies and INGOs such as UNHCR. The national system's current data stratification—categorized by nationality for some indicators (Jordanians and non-Jordanians)—provides limited insights specific to the substantial Syrian refugee population in Jordan, highlighting a gap in equity-related health data. As of January 2024, around 730,000 Syrian refugees are registered with the UNHCR in Jordan [56], with an estimated 80% living below the poverty line and 60% in extreme poverty [57]. The ongoing collection of data for key demographic groups such as mothers, children, adolescents, and refugees is essential. It allows for the monitoring of individual risk factors and the identification of both positive and negative health trends [58]. These data are invaluable for prompt health policy decisions and can significantly influence the overall health status of the population [58].

The indicators generated from this study comprise an initial core set of indicators that can be further revised and incrementally scaled up once the system is well-established and sufficiently mature to collect, measure, report and respond to the needs of policymakers and decision-makers. Policymakers can use the identified indicators to prioritize areas requiring intervention, allocate resources efficiently, and monitor the impact of health programs over time. Standardized data collection protocols and improved reporting mechanisms are also crucial for generating actionable insights and promoting informed decision-making at both the policy and healthcare delivery levels. As such, the procedures manuals generated from this study were subsequently shared with policymakers and practitioners to carry forward the task of liaising with different stakeholders to identify data sources, gaps, data analysis needs, and decision support systems that need to be scaled up to support this undertaking.

Effective governance of indicator development, monitoring, and periodic updates is essential to maintain a high-functioning health system. It is advisable to involve a diverse array of stakeholders, including policymakers, healthcare providers, patient representatives, and academic researchers in these processes to promote inclusive and more equitable decision-making [59]. Governance should involve clear protocols for regularly assessing the relevance and effectiveness of indicators, reflecting evolving health challenges and outcomes. By institutionalizing roles such as a steering committee, project management team, data quality advisory group, and expert panel within government structures, stakeholders can continuously evaluate and update national indicator sets as necessary. It is also vital to adopt the learning health systems approach which relies on a bidirectional flow of information between data sources and users to ultimately improve outcomes and enhance efficiency [60]. Incorporating feedback from end-users can glean insights into their experiences with the indicators and gather their recommendations on draft criteria for evaluating them [59]. This in turn, ensures that indicators remain relevant and aligned with the dynamic and evolving health needs and priorities of all community segments [61].

Moreover, the implementation cost considerations are critical. Detailed budgeting and securing sustainable funding are imperative to support the ongoing operations and expansion of health information systems. Anticipated costs include training personnel, technology updates, data management, and security measures [62]. These technical, human, social and organizational considerations should be accounted for in any national level plan for enhancing a HIS. This is particularly in low and middle-income countries and those in the early stages of establishing health information systems which often face challenges in obtaining the necessary resources for development, implementation, and maintenance of HIS [55].

Lastly, once the HIS is sufficiently mature and the capacity is built for collecting and reporting evidence-based performance indicators, considerations could be given to prioritize the selection of a subset of indicators that facilitate meaningful within-country and cross-country comparisons, thereby enhancing the relevance and utility of findings for decision-makers at both national and regional levels. Selecting such indicators not only enables the identification of variations and disparities within different regions of Jordan, but also the benchmarking of performance against regional peers in the MENA region. This comparative analysis is vital for policymakers and stakeholders to assess the strengths and weaknesses of the Jordanian health system, identify best practices, and implement targeted interventions to improve health outcomes and healthcare delivery. It also promotes regional benchmarks and fosters a competitive drive towards healthcare excellence, which is instrumental in driving systemic improvements [63].

Some of the challenges and potential limitations of this study are worth noting. Firstly, the data collection period coincided with the onset of the COVID-19 pandemic, resulting in numerous delays and the rescheduling of initially planned stakeholder meetings. This situation also impacted our ability to convene the same group of stakeholders for both prioritization and subsequent validation sessions. Despite these challenges, we endeavored to mitigate the disruptions by arranging targeted follow-up meetings and seamlessly transitioning to online interviews and group discussions when necessary. Additionally, to accommodate participants, tools and manuals were distributed via email for input in certain instances. Moreover, we engaged a diverse array of stakeholders, including policymakers, healthcare professionals, researchers, and representatives from non-governmental and intergovernmental organizations, to ensure the selection of relevant indicators and foster broader acceptance of the chosen metrics. Secondly, despite our attempt to develop and validate a core set of national indicators for regular reporting, the additional indicators suggested by participants warrant further review and validation with a diverse group of stakeholders in Jordan. Finally, while this study describes the process of selecting, developing, and validating core set of indicators, it does not discuss the actual implementation of indicators or the process for using results of indicators in policymaking or regulation. In future phases, we aim to examine the implementation and use of the indicators in healthcare quality improvement and policymaking in Jordan.

## Conclusion

In this study, we conducted a baseline assessment of the national HIS in Jordan and achieved consensus on a standardized set of national indicators for the core health system, maternal, child and adolescent health, and refugee health, respectively. The findings and gaps emerging from this study can act as a basis to inform national deliberations and dialogues among key stakeholders in Jordan. They can also contribute to the broader discourse on HIS, emphasizing the need for ongoing efforts to enhance data quality, stakeholder collaboration, and technological infrastructure. Furthermore, the study's approach, tools, and manuals can be adapted by other countries looking to strengthen their HIS and promote data-driven health policymaking and practice.

## Supplementary Information

Additional file 1. Prioritization and validation tools

Additional file 2. Jordan action plan

Additional file 3. Overview and evolution of indicators from Jordan’s national health information system

Additional file 4. Procedure manual for maternal, child and adolescent health.

## Data Availability

All data generated or analyzed during this study are included in this published article and its supplementary information files. Baseline assessment tools are available upon request from authors.

## Abbreviations

HIS: Health information system
INGOs: International non-governmental organizations
MENA: Middle East and North Africa region
MoH: Ministry of health
SDGs: Sustainable development goals
UNHCR: United nations high commissioner for refugees
UNICEF: United nations children's fund
WHO: World Health Organization
