# Supplementary material for: Promoting data-driven decision-making in Jordan: strengthening national health information system and achieving consensus on core set of health system indicators
Source: Reprod Health. 2025 May 31;22(Suppl 1):72. doi: 10.1186/s12978-025-01988-1 (PMC12125760; doi:10.1186/s12978-025-01988-1)
Supplement: Supplementary file 1 — Additional file 1. Prioritization and validation tools [file 12978_2025_1988_MOESM1_ESM.pdf]

Core Health Systems Indicators

© K2P Center (as part of IDRC-funded project to strengthen national health information systems)

Priority Setting Tool for Core Health System Indicators

أداة تحديد اولويات لمؤشرات النظام الصحي األساسية

Instructions

التعليمات

You are kindly invited to complete the priority setting tool to assess a set of 167 indicators compiled from global/regional reference list of core indicators in addition to Jordanian health system and National Health Strategy (NHS), for possible inclusion in the National Health Information System of Jordan.

The purpose of this exercise is to optimize the existing national health information system in Jordan by achieving consensus on a core set of indicators that draw on those that are globally recommended and standardized and that are relevant and useful for decision-makers at each level of the health system and that align indicators with health-related SDG targets.

Please note that what you will share with us will remain confidential and anonymous

يرجى من حضرتكم ملء أداة تحديد األولويات لتقييم مجموعة من المؤشرات)167(التي تم جمعها من مراجع اقليمية وعالمية ومن االستراتيجية الوطنية للنظام الصحي األردني وذلك بهدف دراسة إمكانية إدراجها في نظام المعلومات الصحية الوطني في األردن.

الهدف من هذا التمرين هو تحسين نظام المعلومات الصحية الوطني الحالي في األردن و ذلك من خالل التوصل إلى إجماع بشأن مجموعة من المؤشرات األساسية المبنية على التوصيات و المعايير العالمية التي تفيد صناع القرار في كافة مستويات
النظام الصحي األردني والتي تساهم في تحقيق أهداف التنمية المستدامة المتعلقة بالصحة في األردن.

يرجى مالحظة أن ما ستشاركونه معنا سيظل سريًا ومجهول الهوية

© K2P Center (as part of IDRC-funded project to strengthen national health information systems)

The indicators selected for inclusion encompass the following four **dimensions:**

1.	**Health Status**	Indicators include mortality by age, sex and cause and health related SDGs as well as core morbidity and fertility indicators
2.	**Risk factor indicators**	Indicators include those relating to nutrition, environmental, behavioral, injuries and violence as well as socioeconomic factors
3.	**Service coverage indicators**	indicators reflect priorities across the spectrum of health services including immunization, HIV, TB, non-communicable diseases, mental health and substance abuse
4.	**Health system indicators**	Indicators include indicators of health system inputs and outputs such as health facility density and distribution, health workforce, health information and quality and safety of care

تشمل المؤشرات المختارة ألابعاد ألاربعة التالية:

.1 **الحالة الصحية**	تشمل الوفاة)حسب العمر, الجنس و السبب(, المرض, الخصوبة و أهداف التنمية المستدامة المتعلقة بالصحة
.2 **عوامل الخطر**	تشمل العوامل المتعلقة بالتغذية, البيئة, السلوك, العنف بالإضافة إلى العوامل االجتماعية و االقتصادية
.3 **تغطية الخدمة**	تشمل الخدمات الصحية ألاولوية المتعلقة بالتلقيح, فيروس نقص المناعة البشرية, السل, ألامراض الغيرمنقولة, الصحة النفسية و والإدمان .
.4 **النظام الصحي**	ل مدخالت و مخرجات النظام الصحي مثل كثافة المرافق الصحية وتوزيعها ، القوى العاملة في القطاع الصحي ، المعلومات الصحية, جودة وسالمة الرعاية الصحية

Please specify the position that best suits you:

- ☐ Representative of government/policymaker
- ☐ Academia/researcher
- ☐ Health professional association
- ☐ Healthcare director/manager
- ☐ Representative of a non-governmental association
- ☐ Other:______________________________

يرجى اختيار المنصب المناسب لكم:

☐ ممثل عن منظمة حكومية /صانع قرار
☐ أكاديمية / باحث ☐
جمعية الصحة المهنية ☐ مدير
الرعاية الصحية ☐ ممثل عن منظمة
غير حكوميّة
☐ غيره:______________________________

© K2P Center (as part of IDRC-funded project to strengthen national health information systems)

Please rate each indicator based on the following criteria:

Criteria	Definitions	Scoring technique
Important	• The indicator reflects an issue that is important to the general population and relevant stakeholders in the health system;<br>• The indicator represents the most critical issues and priorities of the health systems	Please rate each indicator by selecting Yes or No
Feasible	The indicator is easy to measure in terms of:<br>• Data availability; or<br>• Minimal burden of data collection; or<br>• Minimal costs of data collection.	Please rate each indicator by selecting Yes or No
Actionable	• The indicator can help identify opportunities for health system improvement at the national and regional level<br>• The indicator provides information that is appropriate and useful for guiding policies and programs as well as for decision-making;	Please rate each indicator by selecting High or Low

- If an indicator is not relevant to your context, please select the “Not Applicable” (NA) option.
- Please note that the measurement tools for the selected indicators will be developed at the next stage of implementation and will be discussed with the respective stakeholders in Jordan.

يرجى تقييم كل مؤشر بناء ًعلى المعايير التالية:

المعيار	تعريف المعيار	كيفية التقييم
مـهم	• يعكس المؤشر مشكلة مهمة لعامة السكان وأصحاب المصلحة المعنيين في النظام الصحي<br>• يعكس المؤشر أولويات النظام الصحي<br>• المؤشر مهم لضبط و تنظيم السياسات و النظم الصحية	**يرجى تقييم المؤشر بالإجابة ب نعم أو ال**
عملـي	من السهل قياس المؤشر من ناحية:<br>• توافر البيانات<br>• الحد األدنى من عبء جمع البيانات<br>• الحد األدنى من التكاليف لجمع البيانات	**يرجى تقييم المؤشر بالإجابة ب نعم أو ال**
فعال	يساعد المؤشر في تحديد فرص تحسين النظام الصحي على المستوى الوطني واإلقليمي<br>• يوفر المؤشر معلومات مناسبة ومفيدة لترشيد السياسات والبرامج وكذلك لصنع القرار	**يرجى تقييم المؤشر باإلجابة ب جداً أو قليلً**

- إذا كان المؤشرغير مناسب لسياقك ، فيرجى باإلجابة ب "غير قابل للتطبيق".
- يرجى المالحظة أنه سيتم تطوير أدوات القياس للمؤشرات المختارة في المرحلة التالية من التنفيذ وستتم مناقشتها مع أصحاب المصلحة المعنيين في األردن.

© K2P Center (as part of IDRC-funded project to strengthen national health information systems)

Dimension 1: Health Status

الحالة الصحية

		Important<br>مهم		Feasible<br>عملي		Actionable<br>فعال		NA
		Yes	No	Yes	No	High	Low	
	**Mortality by age and sex**							
1.	Life expectancy at birth [gender-sensitive]<br>متوسط العمر المتوقع عند الوالدة	1	2	1	2	1	2	
2.	Adult mortality rate between 15 and 60 years of age<br>معدل وفيات البالغين بين 15 و 60 سنة	1	2	1	2	1	2	
3.	Premature non-communicable disease (NCD) mortality-disaggregated by type and sex [SDG 3.4.1]<br>الوفيات المبكرة الناجمة عن ألمراض غير المعدية- مصنفة حسب النوع والجنس	1	2	1	2	1	2	
4.	Tuberculosis (TB) mortality rate*<br>معدل الوفيات بالسل*	1	2	1	2	1	2	
5.	AIDS-related mortality rate<br>معدل الوفيات المرتبطة باإليدز	1	2	1	2	1	2	
6.	Drug-related mortality rate<br>معدل الوفيات المرتبطة بالمخدرات	1	2	1	2	1	2	
7.	Mortality from household and ambient air pollution [SDG 3.9.1]<br>الوفيات الناجمة عن تلوث الهواء في المنزل والبيئة المحيطة	1	2	1	2	1	2	
8.	Mortality from unsafe water, unsafe sanitation and lack of hygiene [SDG 3.9.2]<br>الوفيات الناجمة عن المياه غير المأمونة والصرف الصحي غير آالمن ونقص النظافة	1	2	1	2	1	2	
9.	Mortality rate from road traffic injuries [SDG 3.6.1]<br>معدل الوفيات الناجمة عن إصابات حوادث السير	1	2	1	2	1	2	

© K2P Center (as part of IDRC-funded project to strengthen national health information systems)

		Important مهم		Feasible عملي		Actionable فعال		NA
		Yes	No	Yes	No	High	Low	
10.	Mortality from unintentional poisoning [SDG 3.9.3] الوفيات الناجمة عن التسمم غير المقصود	1	2	1	2	1	2	
11.	Suicide rate, by age [SDG 3.4.2] [gender-sensitive] معدل االنتحار ، حسب العمر	1	2	1	2	1	2	
12.	Mortality rate due to homicide [SDG 16.1.1] معدل الوفيات بسبب القتل	1	2	1	2	1	2	
13.	Conflict-related deaths per 100 000 population [SDG 16.1.2] الوفيات المرتبطة بالنزاع لكل 100,000 من السكان	1	2	1	2	1	2	
	**Fertility**							
14.	Total fertility rate* معدل الخصوبة الكلي*	1	2	1	2	1	2	
	**Morbidity**							
15.	New cases of vaccine-preventable diseases حاالت جديدة لألمراض التي يمكن الوقاية منها باللقاحات	1	2	1	2	1	2	
16.	New cases of International Health Regulations (IHR)-notifiable diseases and other notifiable diseases per year حاالت جديدة من األمراض التي يمكن التبليغ عنها سنوياً حسب تشريعات الصحة الدولية	1	2	1	2	1	2	
17.	HIV prevalence rate; by age [gender-sensitive] معدل انتشار فيروس نقص المناعة البشرية ؛ حسب العمر	1	2	1	2	1	2	
18.	HIV incidence rate [SDG 3.3.1] معدل اإلصابة بفيروس نقص المناعة البشرية	1	2	1	2	1	2	
19.	Sexually transmitted infections (STIs) incidence rate (number of new cases of reported STIs (syndromic or etiological reporting) in a specified time period	1	2	1	2	1	2	

© K2P Center (as part of IDRC-funded project to strengthen national health information systems)

		Important مهم		Feasible عملي		Actionable فعال		NA
		Yes	No	Yes	No	High	Low	
	معدل الإصابة بالأمراض المنقولة جنسياً (عدد<br>لجديدة لألمراض المنقولة جنسياً المبلغ عنها-الإبالغ عن<br>المتالزمة أو المسببات<br>المرضية في فترة زمنية محددة)							
20.	Prevalence and incidence rate of neglected tropical diseases (Dracunculiasis; Leishmaniasis; Leprosy; Rabies; Mycetoma; Lymphatic filariasis; Onchocerciasis; Schistosomiasis; Soil-transmitted helminthiases; Trachoma)<br>انتشار ومعدل الإصابة بالأمراض المدارية المهملة<br>(داء الحييات ، داء الليشمانيات ؛ الجذام ؛ داء الكلب ؛<br>الورم العضلي العقلي ؛ داء<br>الفيالريات اللمفاوي ؛ داء كالبية الذنب ؛ داء<br>المنشقات ؛ داء البلهارسيات المنقولة بالتربة ؛<br>التراخوما)	1	2	1	2	1	2	
21.	Cancer incidence, by type of cancer*<br>الإصابة بالسرطان ، حسب نوع السرطان*	1	2	1	2	1	2	
22.	Prevalence of cardiovascular disease in adults- disaggregated by type<br>راض القلب والأوعية الدموية في البالغين -<br>مصنفة حسب النوع	1	2	1	2	1	2	
23.	Acute myocardial infarction (AMI) incidence rate<br>معدل الإصابة بذبحة قلبية حادة	1	2	1	2	1	2	
24.	Stroke incidence rate<br>معدل الإصابة بالسكتة الدماغية	1	2	1	2	1	2	
25.	Prevalence of diabetes (raised blood glucose) in adults<br>انتشار مرض السكري (ارتفاع السكر في الدم)<br>في البالغين	1	2	1	2	1	2	
26.	Prevalence of chronic respiratory diseases –disaggregated by type (such as chronic obstructed pulmonary disease and asthma)<br>انتشار أمراض الجهاز التنفسي المزمنة - مصنفة<br>حسب النوع (مثل مرض الانسداد<br>الرئوي المزمن والربو)	1	2	1	2	1	2	

© K2P Center (as part of IDRC-funded project to strengthen national health information systems)

		Important<br>مهم		Feasible<br>عملي		Actionable<br>فعال		NA
		Yes	No	Yes	No	High	Low	
27.	Prevalence of end-stage kidney disease in adults<br>انتشار مرض الكلى بالمرحلة النهائية عند البالغين	1	2	1	2	1	2	
28.	Prevalence of chronic depression in adults<br>انتشار االكتئاب المزمن عند البالغين	1	2	1	2	1	2	
29.	Prevalence of dementia/Alzheimer<br>انتشار الخرف / مرض الزهايمر	1	2	1	2	1	2	
30.	Prevalence of general musculoskeletal pain in adults<br>انتشار األلم العضلي الهيكلي العام عند البالغين	1	2	1	2	1	2	
31.	Prevalence of severe visual impairment and blindness (0 to 14 years, 15 to 49 years and 50 years and older, non-disaggregated by gender)<br>انتشار ضعف البصر الشديد والعمى)0 إلى 14 سنة ، 15 إلى 49 سنة و 50 سنة وكبار السن ، غير مصنفة حسب الجنس(	1	2	1	2	1	2	
32.	DALYs saved<br>سنوات العمر المصححة بإحتساب مدد العجز التي تم أنقاذها	1	2	1	2	1	2	

© K2P Center (as part of IDRC-funded project to strengthen national health information systems)

I. Please list any additional indicators that are missing from this dimension which you believe are important for inclusion

يرجى ذكر أي مؤشرات إضافية مفقودة من هذا البعد تعتقد أنها مهمة لإلدراج

II. Please state your comments or areas for clarification on this dimension

يرجى ذكر تعليقاتك أو أي نقاط تود توضيحها بالنسبة لهذا البعد

© K2P Center (as part of IDRC-funded project to strengthen national health information systems)

Dimension 2: Risk Factors and Behaviors

عوامل الخطر والسلوكيات

		Important مهم		Feasible عملي		Actionable فعال		NA
		Yes	No	Yes	No	High	Low	
	**Infections**							
1.	Percentage of key populations at higher risk (who inject drugs, sex workers, men who have sex with men) who have received an HIV test in the past 12 months and know their results<br>النسبة المئوية للسكان الرئيسيين ألأكثر عرضة للخطر)الذين يتعاطون المخدرات ، والمشتغلين بالجنس ، والرجال الذين يمارسون الجنس مع الرجال(الذين تلقوا اختبار فيروس نقص المناعة البشرية في ألأشهر الـ 12 الماضية ويعرفون نتائجهم	1	2	1	2	1	2	
2.	Percentage of individuals (aged 15–24 years) reporting using condom at last high-risk sexual encounter; men and women [gender-sensitive]<br>نسبة المئوية لألفراد)الذين تتراوح أعمارهم بين 15 و 24 سنة(الذين يبلغون عن استخدام الواقي الذكري في آخر لقاء جنسي عالي الخطورة)رجال ونساء(	1	2	1	2	1	2	
	**Environmental risk factors**							
3.	Percentage of population using safely managed drinking-water services [SDG 6.1.1] [gender-sensitive]<br>نسبة السكان الذين يستخدمون خدمات مياه الشرب المدارة بأمان	1	2	1	2	1	2	
4.	Percentage of population using safely managed sanitation services [SDG 6.2.1a/6.2.1b] [gender-sensitive]<br>نسبة السكان الذين يستخدمون خدمات الصرف الصحي المدارة بأمان	1	2	1	2	1	2	
5.	Percentage of population with primary reliance on clean fuels and technologies [SDG 7.1.2] [gender-sensitive]<br>نسبة السكان الذين يعتمدون بشكل أساسي على الوقود النظيف والتقنيات النظيفة	1	2	1	2	1	2	
6.	Air pollution level in cities [SDG 11.6.2]	1	2	1	2	1	2	

© K2P Center (as part of IDRC-funded project to strengthen national health information systems)

		Important مهم		Feasible عملي		Actionable فعال		NA
		Yes	No	Yes	No	High	Low	
	مستوى تلوث الهواء في المدن							
	**Non-communicable diseases**							
7.	Total alcohol per capita consumption; by age and sex [SDG 3.5.2] [gender-sensitive]*<br>إجمالي استهلاك الكحول للفرد- مصنفة حسب العمر والجنس*	1	2	1	2	1	2	
8.	Prevalence of tobacco use -disaggregated by age and sex [SDG 3.a.1] [gender-sensitive]*<br>انتشار تعاطي التبغ بين البالغين والمراهقين - مصنفة حسب العمر والجنس*	1	2	1	2	1	2	
9.	Prevalence of hypertension (raised blood pressure) among adults*<br>انتشار ارتفاع ضغط الدم بين البالغين*	1	2	1	2	1	2	
10.	Prevalence of raised cholesterol among adults<br>انتشار الكوليسترول المرتفع بين البالغين	1	2	1	2	1	2	
11.	Mean population salt intake<br>معدل تناول الملح عند السكان	1	2	1	2	1	2	
12.	Prevalence of insufficiently physically active persons by age and sex<br>انتشار ألشخاص غير النشطين بدنياً بما فيه الكفاية حسب العمر والجنس	1	2	1	2	1	2	
13.	Prevalence of overweight and obesity in adults and adolescents- disaggregated by age and sex [gender sensitive] [SDG 2.2.2]*<br>انتشار زيادة الوزن والسمنة لدى البالغين والمراهقين - مصنفة حسب العمر والجنس*	1	2	1	2	1	2	
	**Injuries and harmful traditional practices**							
14.	Prevalence of illicit drug use in population; by age and sex [gender-sensitive]<br>نتشار تعاطي المخدرات غير المشروع عند السكان- حسب العمر والجنس	1	2	1	2	1	2	
15.	Prevalence of intimate partner violence, by type (physical, sexual, psychological) [SDG 5.2.1] [gender-sensitive]*<br>انتشار العنف الممارس من الشريك الحميم- حسب النوع (جسدي ، جنسي ، نفسي)*	1	2	1	2	1	2	

© K2P Center (as part of IDRC-funded project to strengthen national health information systems)

		Important<br>مهم		Feasible<br>عملي		Actionable<br>فعال		NA
		Yes	No	Yes	No	High	Low	
16.	Prevalence of non-partner sexual violence [SDG 5.2.2] [gender-sensitive]<br>انتشار العنف الجنسي الممارس من غير الشريك	1	2	1	2	1	2	
	**Demographic and socioeconomic determinants**							
17.	Population size<br>حجم السكان	1	2	1	2	1	2	
18.	Sex ratio at birth (male births per female births)*<br>نسبة المواليد الذكور مقابل المواليد الإناث*	1	2	1	2	1	2	
19.	Population growth rate<br>معدل النمو السكاني	1	2	1	2	1	2	
20.	Educational attainment, by level, age and sex<br>التحصيل العلمي ، حسب المستوى والعمر والجنس	1	2	1	2	1	2	
21.	Percentage of population living below national poverty line (poverty headcount ratio at 1.25$ a day (PPP) [gender-sensitive]<br>النسبة المئوية للسكان الذين يعيشون تحت خط الفقر الوطني)بمعدل 1.25 دوالر في اليوم(	1	2	1	2	1	2	
22.	Human development index*<br>مؤشر التنمية البشرية*	1	2	1	2	1	2	
23.	Rank in elderly health on Global Watch index*<br>المرتبة في صحة المسنين على مؤشر غلوبال ووتش*	1	2	1	2	1	2	
24.	Percentage of married women age 15-49 participating in the three decisions (own health care, major household purchases, and visiting family) [gender sensitive]<br>النسبة المئوية للنساء المتزوجات الالواتي تتراوح أعمارهن بين 15 و 49 عاًما والمشاركين في القرارات الثلاثة)الرعاية الصحية الخاصة ، والمشتريات الرئيسية ، لألسرة ، وزيارة األسرة(	1	2	1	2	1	2	

© K2P Center (as part of IDRC-funded project to strengthen national health information systems)

I. **Please list any additional indicators that are missing from this dimension which you believe are important for inclusion**

يرجى ذكر أي مؤشرات إضافية مفقودة من هذا البعد تعتقد أنها مهمة لإلدراج

II. **Please state your comments or areas for clarification on this dimension**

يرجى ذكر تعليقاتك أو أي نقاط تود توضيحها بالنسبة لهذا البعد

© K2P Center (as part of IDRC-funded project to strengthen national health information systems)

Dimension 3: Service Coverage

تغطية الخدمة

		Important<br>مهم		Feasible<br>عملي		Actionable<br>فعال		NA
		Yes	No	Yes	No	High	Low	
	**Immunization**							
1.	Immunization coverage rate by vaccine for each vaccine in the national schedule [SDG 3.b.1]*<br>معدل تغطية لقاحات الجدول الوطني- مصنفة حسب اللقاح*	1	2	1	2	1	2	
	**HIV and tuberculosis (TB)**							
2.	Antiretroviral therapy (ART) coverage *<br>تغطية العالج المضاد للفيروسات الرجعية*	1	2	1	2	1	2	
	**Screening and preventive care**							
3.	Cervical cancer screening (proportion of women between the ages of 30–49 screened for cervical cancer at least once, or more often, and for lower or higher age groups according to national programs or policies)<br>فحص سرطان عنق الرحم)نسبة النساء الالئي تتراوح أعمارهن بين 30 و 49 سنة الالواتي يتم فحصهن لسرطان عنق الرحم مرة واحدة على األقل ، أو أكثر ، وللفئات العمرية األدنى أو األعلى وفقًا للبرامج أو السياسات الوطنية(	1	2	1	2	1	2	
4.	Breast cancer screening<br>فحص سرطان الثدي	1	2	1	2	1	2	
5.	Colon cancer screening<br>فحص سرطان القولون	1	2	1	2	1	2	
6.	Coverage of services for severe mental health disorders (percentage of persons with a severe mental disorder (psychosis, bipolar affective disorder, moderate-severe depression) who are using services)<br>لية خدمات اضطرابات الصحة النفسية)النسبة المئوية لألشخاص الذين يعانون من اضطراب نفسي شديد)ذهان ، اضطراب عاطفي ثنائي القطب ، اكتئاب شديد(							

© K2P Center (as part of IDRC-funded project to strengthen national health information systems)

		Important مهم		Feasible عملي		Actionable فعال		NA
		Yes	No	Yes	No	High	Low	
7.	Treatment coverage for alcohol and drug dependence (proportion of people with alcohol or drug dependence that are in contact with treatment services, i.e. currently receiving treatment or in remission or relapse, but still in contact with treatment services) تغطية عالج إدمان الكحول والمخدرات)نسبة األشخاص الذين يتعاطون الكحول أو المخدرات الذين هم على اتصال مع خدمات العالج ، أي يتلقون حاليًا عال ًجا أو في انتكاسة ، لكن ال يزالون على اتصال بخدمات العالج(							
8.	Treatment coverage for opioid dependence تغطية العالج العتماد المواد األفيونية	1	2	1	2	1	2	
9.	Use of assistive devices among people with disabilities استخدام األجهزة المساعدة بين األشخاص ذوي اإلعاقة	1	2	1	2	1	2	
10.	Coverage of essential health services [SDG 3.8.1] تغطية الخدمات الصحية األساسية	1	2	1	2	1	2	
11.	Percentage of controlled hypertensive patients* نسبة المرضى الذين يعانون من ارتفاع ضغط الدم التي تمت السيطرة عليه*	1	2	1	2	1	2	
12.	نسبة Percentage of controlled diabetic المرضى الذين يعانون من السكري التي تمت السيطرة عليه*	1	2	1	2	1	2	
13.	Proportion of population aged 15 years and over receiving regular health examination within 12 months; نسبة by sex and age [gender-sensitive]* السكان الذين تبلغ أعمارهم 15 عا ًما أو أكثر الذين يتلقون فح ًصا صحيًا منتظ ًما في غضون 12 شه ًرا ؛ حسب الجنس والعمر*	1	2	1	2	1	2	
14.	Percentage of population treated for sexually transmitted infections (STI)	1	2	1	2	1	2	

© K2P Center (as part of IDRC-funded project to strengthen national health information systems)

		Important مهم		Feasible عملي		Actionable فعال		NA
		Yes	No	Yes	No	High	Low	
	النسبة المئوية للسكان الذين عولجوا من األمراض المنقولة جنسياً							
15.	Percentage of health facilities providing sexually transmitted infections (STI) services with adequate drug supply النسبة المئوية للمرافق الصحية التي تقدم خدمات العدوى المنقولة جنسياً مع توفير األدوية الكافية	1	2	1	2	1	2	
16.	Percentage of health centers providing immunization نسبة المراكز الصحية التي تقدم التطعيم	1	2	1	2	1	2	
17.	Percentage of health facilities that offer gender-sensitive, patient-centered care (e.g. rape crisis center, [gender-sensitive] نسبة المنشآت الصحية التي تقدم رعاية تراعي الفوارق بين الجنسين وتركز على المريض	1	2	1	2	1	2	
18.	Annual Exam Retina – Referred and tested* امتحان سنوي لشبكية العين - تمت إحالته واختباره*	1	2	1	2	1	2	
19.	Number of qualified centers to provide palliative care and psychological care for cancer patients and families* عدد المراكز المؤهلة لتوفير الرعاية التلطيفية والرعاية النفسية لمرضى السرطان وعائالتهم*	1	2	1	2	1	2	
20.	Number of rehabilitated comprehensive health centers to receive and treat psychiatric cases* عدد مراكز إعادة التأهيل الصحية الشاملة لتلقي وعالج الحاالت النفسية*	1	2	1	2	1	2	

© K2P Center (as part of IDRC-funded project to strengthen national health information systems)

III. Please list any additional indicators that are missing from this dimension which you believe are important for inclusion

يرجى ذكر أي مؤشرات إضافية مفقودة من هذا البعد تعتقد أنها مهمة لإلدراج

IV. Please state your comments or areas for clarification on this dimension

يرجى ذكر تعليقاتك أو أي نقاط تود توضيحها بالنسبة لهذا البعد

© K2P Center (as part of IDRC-funded project to strengthen national health information systems)

Dimension 4: Health System

النظام الصحي

		Important<br>مهم		Feasible<br>عملي		Actionable<br>فعال		NA
		Yes	No	Yes	No	High	Low	
	**Service Quality and Safety**							
1.	Number of healthcare institutions with accreditation- by type of institution*<br>عدد مؤسسات الرعاية الصحية الحاصلة على االعتماد - حسب نوع المؤسسة*	1	2	1	2	1	2	
2.	Number of hospitals that have ambulance and emergency specialist at their disposal*<br>عدد المستشفيات التي لديها سيارة إسعاف و أخصائي طوارئ تحت تصرفها*	1	2	1	2	1	2	
3.	Perioperative mortality rate<br>معدل الوفيات المحيطة بالجراحة	1	2	1	2	1	2	
4.	Antiretroviral therapy (ART) retention rate<br>معدل استبقاء العالج المضاد للفيروسات الرجعية	1	2	1	2	1	2	
5.	Hospital standardized mortality ratio<br>نسبة الوفيات الموحدة في المستشفى	1	2	1	2	1	2	
6.	acute Thirty-day mortality after (AMI) admission to hospital for الوفاة لمدة ثالثين يو myocardial infarction<br>ًما بعد دخول المستشفى<br>بسبب ذبحة قلبية حاد							
7.	Percentage of unplanned readmissions within 28 days of discharge related to the primary admission<br>ة المئوية لعمليات إعادة اإلدخال غير المخططلها<br>في غضون 28 يو ًما من<br>الخروج المتعلقة بالدخول األساسي	1	2	1	2	1	2	
8.	Percentage of unscheduled returns to the Emergency Department within 48 hours related to primary visit	1	2	1	2	1	2	

© K2P Center (as part of IDRC-funded project to strengthen national health information systems)

		Important مهم		Feasible عملي		Actionable فعال		NA
		Yes	No	Yes	No	High	Low	
	بة المئوية للعائدات غير المجدولة إلى إدارة الطوارئ خالل 48 ساعة تتعلق بالزيارة األولية							
9.	Prevalence and incidence rate of hospital-acquired infections (% of patients hospitalized) معدل انتشار ومعدل اإلصابة باألمراض المكتسبة من المستشفيات)٪ من المرضى الذين أدخلوا المستشفى(	1	2	1	2	1	2	
10.	Surgical site infections rate معدل إصابات الموقع الجراحي							
11.	Rate of Methicillin-resistant Staphylococcus aureus (MRSA) infections معدل اإلصابات بالمكورات العنقودية الذهبية المقاومة للميثيسيلين	1	2	1	2	1	2	
12.	Incidence of inpatient hospital-onset Clostridium difficile اإلصابة بالمستشفى بداية المستشفى المطثية العسيرة	1	2	1	2	1	2	
13.	Postoperative pulmonary embolism or deep vein thrombosis rate انسداد رئوي بعد العملية الجراحية أو معدل تجلط األوردة العميقة	1	2	1	2	1	2	
14.	Percentage compliance with hand hygiene نسبة االلتزام بنظافة اليدين	1	2	1	2	1	2	
15.	Ratio of sentinel events reported to total adverse events reported نسبة الحوادث الغير المتوقعة في المرفق الصحي التي قد تؤدي الى الموت أو الى اصابة خطيرة مقارنة بنسبة الحوادث الغير المتوقعة كردفعل خطير عند أخذ دواء أو لقاح	1	2	1	2	1	2	
16.	Blood and body fluid staff exposure rate معدل تعرض الموظفين للدم والسوائل	1	2	1	2	1	2	

© K2P Center (as part of IDRC-funded project to strengthen national health information systems)

		Important مهم		Feasible عملي		Actionable فعال		NA
		Yes	No	Yes	No	High	Low	
17.	Percentage compliance with surgical safety checklist<br>النسبة المئوية لالمتثال لقائمة فحص السالمة الجراحية	1	2	1	2	1	2	
18.	Patient safety culture rate<br>معدل ثقافة سالمة المريض	1	2	1	2	1	2	
19.	Ambulatory Care Sensitive Conditions (ACSC) hospitalization rate (i.e., hospitalization for health conditions that may have been prevented or managed by primary health care)<br>معدل اإلستشفاء للحاالت الصحية التي يمكن الوقاية منها من قبل الرعاية الصحية األولية	1	2	1	2	1	2	
20.	People with diabetes with a prescription of recommended antihypertensive medication in the past year<br>مرضى السكري مع وصفة طبية من األدوية الخافضة للضغط موصى بها في العام الماضي	1	2	1	2	1	2	
21.	Major lower extremity amputation in adults with diabetes<br>بتر األطراف السفلى عند البالغين المصابين بالسكري	1	2	1	2	1	2	
22.	Foreign body left in during procedure<br>جسم غريب ترك في أثناء اإلجراء	1	2	1	2	1	2	
23.	Blood culture contamination rate	1	2	1	2	1	2	
24.	Postoperative sepsis in abdominal surgeries<br>إنتان ما بعد العملية الجراحية في العمليات الجراحية في البطن	1	2	1	2	1	2	
25.	Cancer survival rates*<br>معدالت النجاة من السرطان*	1	2	1	2	1	2	
26.	Five-year survival rates for breast, cervix and colon cancer*	1	2	1	2	1	2	

© K2P Center (as part of IDRC-funded project to strengthen national health information systems)

		Important مهم		Feasible عملي		Actionable فعال		NA
		Yes	No	Yes	No	High	Low	
	ت البقاء على قيد الحياة لمدة خمس سنوات لسرطان الثدي وعنق الرحم والقولون*							
27.	Percentage medication reconciliation at admission تحديث الوصفة الطبية ألدوية المريض عند دخول المستشفى	1	2	1	2	1	2	
28.	Medication error rate معدل خطأ الدواء	1	2	1	2	1	2	
29.	Percentage diabetics aged 18-75 years who received Hemoglobin A1c (HbA1c) test in primary healthcare نسبة المئوية لمرضى السكر الذين تتراوح أعمارهم بين 18 و 75 عاماً والذين تلقوا اختبار الهيموغلوبين في الرعاية الصحية األولية	1	2	1	2	1	2	
30.	Percentage hypertensive patients with BP 140/90 or less in primary healthcare ة المئوية لمرضى ارتفاع ضغط الدم الذين لديهم معدل ضغط 140/90 في الرعاية الصحية األولية	1	2	1	2	1	2	
31.	Average waiting time, in primary care, for patient to see doctor; men and women [gender-sensitive] متوسط وقت االنتظار ، في الرعاية األولية لرؤية المريض ؛ رجال ونساء	1	2	1	2	1	2	
32.	Reported waiting times for access to specialist (care) أوقات االنتظار للوصول إلى متخصص	1	2	1	2	1	2	
33.	Average inpatient waiting time for elective (i.e. non-urgent) surgeries – cataract, coronary angioplasty, hip replacement, knee replacement) [gender-sensitive]	1	2	1	2	1	2	

© K2P Center (as part of IDRC-funded project to strengthen national health information systems)

		Important مهم		Feasible عملي		Actionable فعال		NA
		Yes	No	Yes	No	High	Low	
	متوسط وقت انتظار المرضى الداخلين إلجراء العمليات الجراحية االختيارية (أي غير العاجلة) - إعتام عدسة العين ، رأب األوعية التاجية ، استبدال الورك ، استبدال الركبة							
34.	Waiting time for emergency department care<br>وقت االنتظار لرعاية قسم الطوارئ	1	2	1	2	1	2	
35.	Staff satisfaction rate<br>معدل رضا الموظفين	1	2	1	2	1	2	
36.	Patient/ customer experience/satisfaction with care<br>تجربة المريض / العمالء / الرضا عن الرعاية	1	2	1	2	1	2	
	**Utilization and access**							
37.	Outpatient service utilization (number of outpatient department visits per person per year)<br>استخدام خدمات العيادات الخارجية (عدد زيارات قسم العيادات الخارجية للشخص الواحد في السنة)	1	2	1	2	1	2	
38.	Percentage of outpatient visits obtained from the private sector<br>نسبة زيارات العيادات الخارجية التي تم الحصول عليها من القطاع الخاص	1	2	1	2	1	2	
39.	Number of emergency department visits per year<br>عدد زيارات قسم الطوارئ في السنة	1	2	1	2	1	2	
40.	Hospital (inpatient) admissions per 100 population per year<br>دخول المستشفى (للمرضى الداخليين) لكل 100 نسمة سنويًا	1	2	1	2	1	2	
41.	Surgical volume per 100 000 population<br>حجم الجراحية لكل 000,100 نسمة	1	2	1	2	1	2	

© K2P Center (as part of IDRC-funded project to strengthen national health information systems)

		Important مهم		Feasible عملي		Actionable فعال		NA
		Yes	No	Yes	No	High	Low	
42.	Number of surgeries by type (minor/major, specifics) per 1000 population<br>عدد العمليات الجراحية حسب النوع لكل 1000 نسمة	1	2	1	2	1	2	
43.	Percentage of cancelled elective surgeries<br>نسبة العمليات الجراحية االختيارية الملغاة	1	2	1	2	1	2	
44.	Number, type and distribution of health facilities per 10,000 population<br>عدد ونوع وتوزيع المرافق الصحية لكل 10,000 نسمة	1	2	1	2	1	2	
45.	Hospital bed density and distribution per 10,000 population*<br>كثافة أسرة المستشفى و توزيعها لكل 10,000 نسمة*	1	2	1	2	1	2	
46.	Bed occupancy rate, acute care hospitals only (average number of days when hospital bed was occupied as % of available 365 days)<br>معدل إشغال األسرة ، مستشفيات الرعاية الحادة فقط)متوسط عدد األيام التي شغل فيها سرير المستشفى كنسبة مئوية من 365 يوًما المتاحة(	1	2	1	2	1	2	
47.	Access to emergency surgery (% of population that can access, within 2 hours, a facility that can perform emergency caesarean section, laparotomy and open fraction fixation)<br>الوصول إلى جراحة الطوارئ)٪ من السكان الذين يمكنهم الوصول ، خالل ساعتين ، إلى مرفق يمكنه إجراء عملية قيصرية طارئة ، بضع البطن وتثبيت الكسر المفتوح(	1	2	1	2	1	2	
48.	Access to acute care (% of population who can reach	1	2	1	2	1	2	

© K2P Center (as part of IDRC-funded project to strengthen national health information systems)

		Important مهم		Feasible عملي		Actionable فعال		NA
		Yes	No	Yes	No	High	Low	
	primary, emergency and maternity care services within 15/30 minutes) لحصول على الرعاية الحادة)٪ من السكان الذين يمكنهم الوصول إلى خدمات الرعاية األولية والطوارئ واألمومة في غضون 30/15 دقيقة(							
49.	Proportion of health facilities that have a core set of relevant essential medicines available and affordable on a sustainable basis [SDG 3.b.3] نسبة المنشآت الصحية التي تحتوي على مجموعة أساسية من األدوية األساسية وبأسعار معقولة و على أساس مستدام	1	2	1	2	1	2	
50.	Proportion of men and women accessing provider type of choice [gender-sensitive] ة الرجال والنساء الذين يصلون إلى نوع مقدم الخدمة الذي يختارونه	1	2	1	2	1	2	
51.	General practitioner (GP) utilization (mean number of self-reported visits to general practitioner per person per year) متوسط عدد الزيارات)المبلغ عنها ذاتيا(لطبيب الصحة العامة للشخص الواحد في السنة	1	2	1	2	1	2	
52.	Hospital day-cases as percentage of total patient population (in-patients & day cases), selected diagnoses اليومية في المستشفى كنسبة مئوية من إجمالي عدد المرضى)المرضى الداخليين وحاالت اليوم الواحد(، التشخيصات المختارة	1	2	1	2	1	2	
53.	Number and types of conditions (case mix) treated عدد وأنواع الحاالت المعالجة	1	2	1	2	1	2	
54.	Average length of stay (ALOS) for hospital in-patients, limited diagnoses ط طول مدة اإلقامة للمرضى المقيمين في المستشفى ، مع تشخيصات محدودة	1	2	1	2	1	2	

© K2P Center (as part of IDRC-funded project to strengthen national health information systems)

		Important مهم		Feasible عملي		Actionable فعال		NA
		Yes	No	Yes	No	High	Low	
55.	Percentage of population (rural, poor) served by 24-hour ambulance services<br>النسبة المئوية للسكان)الريف ، الفقراء(الذين تقدم لهم خدمات اإلسعاف على مدار 24 ساعة	1	2	1	2	1	2	
56.	Equity of access to health care services (proportion of people with self-declared unmet needs for medical care due to either financial barriers, waiting times or travelling distances)- by reason [gender sensitive]<br>المساواة في الحصول على خدمات الرعاية الصحية)نسبة األشخاص الذين يحتاجون إلى رعاية طبية غير مستوفين ذاتيًا بسبب إما الحواجز المالية أو أوقات االنتظار أو مسافات السفر(- حسب السبب	1	2	1	2	1	2	
57.	Percentage of referrals from primary healthcare to hospitals (by specific conditions)<br>نسبة اإلحاالت من الرعاية الصحية األولية إلى المستشفيات)حسب ظروف محددة(	1	2	1	2	1	2	
58.	Access to palliative care*<br>الحصول على الرعاية التلطيفية*	1	2	1	2	1	2	
59.	Rehabilitation services utilization *<br>استخدام خدمات إعادة التأهيل*	1	2	1	2	1	2	
	**Health workforce**							
60.	Number of health workers per 10,000 population by type of health worker [SDG 3.c.1] (physicians, nurses, midwives, pharmacists, laboratory technicians, dentists, community and traditional health worker)*<br>عدد العاملين الصحيين لكل 10,000 نسمة حسب نوع العامل الصحي)األطباء والممرضات والقابالت والصيادلة وفنيو المختبرات وأطباء األسنان والعاملون الصحيون المجتمعيون والمعالجون التقليديون(*	1	2	1	2	1	2	
61.	Distribution of health workers, by occupation/specialization,	1	2	1	2	1	2	

© K2P Center (as part of IDRC-funded project to strengthen national health information systems)

		Important مهم		Feasible عملي		Actionable فعال		NA
		Yes	No	Yes	No	High	Low	
	region, level of care, place of work and sex [SDG 3.c.1]<br>زيع العاملين الصحيين حسب المهنة / التخصص والمنطقة ومستوى الرعاية ومكان العمل والجنس							
62.	Number of physicians specialized in elderly medicine*<br>عدد األطباء المتخصصين في رعاية المسنين*	1	2	1	2	1	2	
63.	Output training institutions (density of graduates from health education and training programs) per 100,000 population – by cadre<br>ة الخريجين من برامج التعليم والتدريب الصحي, لكل 100،000 نسمة - حسب الكادر	1	2	1	2	1	2	
64.	Registered recent graduates of health profession educational institutions per 100 000 population<br>تسجيل الخريجين الجدد من المؤسسات التعليمية لمهنة الصحة لكل 100,000 نسمة	1	2	1	2	1	2	
65.	Health worker attrition rates<br>معدالت تناقص العاملين الصحيين	1	2	1	2	1	2	
66.	Health worker absenteeism in public health facilities<br>غياب العامل الصحي في مرافق الصحة العامة	1	2	1	2	1	2	
67.	Turnover rate<br>معدل دوران العمال	1	2	1	2	1	2	
	**Health information**							
68.	Birth registration [SDG 16.9.1]<br>تسجيل الميالد	1	2	1	2	1	2	
69.	Death registration [SDG 17.19.2]<br>تسجيل الموت	1	2	1	2	1	2	
70.	Civil registration coverage of cause-of-death (%)*<br>تغطية السجل المدني لسبب الوفاة)٪(*	1	2	1	2	1	2	
	**Medicines and medical devices**							

© K2P Center (as part of IDRC-funded project to strengthen national health information systems)

		Important مهم		Feasible عملي		Actionable فعال		NA
		Yes	No	Yes	No	High	Low	
71.	Availability of selected essential medicines in public and private health facilities<br>توفر األدوية األساسية المختارة في المرافق الصحية العامة والخاصة	1	2	1	2	1	2	
72.	Number essential NCD medicines reported as "generally available"<br>عدد األدوية ألمراض غير المعدية األساسية المبلغ عنها "متاحة عموًما"	1	2	1	2	1	2	
73.	Density of selected medical devices in public and private health facilities per million population<br>كثافة األجهزة الطبية المختارة في المرافق الصحية العامة والخاصة لكل مليون نسمة	1	2	1	2	1	2	
	**Health financing**							
74.	GDP growth (annual %)<br>نمو الناتج المحلي اإلجمالي)٪ سنويا(	1	2	1	2	1	2	
75.	National health expenditure as % of GDP<br>اإلنفاق الصحي الوطني كنسبة مئوية من الناتج المحلي اإلجمالي	1	2	1	2	1	2	
76.	Total current expenditure on health as % of gross domestic product<br>ي اإلنفاق الحالي على الصحة كنسبة مئوية من الناتج المحلي اإلجمالي	1	2	1	2	1	2	
77.	Total capital expenditure on health as % of current + capital expenditure on health<br>إجمالي اإلنفاق الرأسمالي على الصحة كنسبة مئوية من اإلنفاق الحالي +الرأسمالي على الصحة	1	2	1	2	1	2	
78.	Public domestic sources of current spending on health as % of current health expenditure<br>مصادر المحلية العامة لإلنفاق الحالي على الصحة كنسبة مئوية من اإلنفاق الصحي الحالي	1	2	1	2	1	2	
79.	Private domestic sources of current spending on health as % of current health expenditure	1	2	1	2	1	2	

© K2P Center (as part of IDRC-funded project to strengthen national health information systems)

		Important<br>مهم		Feasible<br>عملي		Actionable<br>فعال		NA
		Yes	No	Yes	No	High	Low	
	سادر المحلية الخاصة لإنفاق الحالي على الصحة<br>كنسبة مئوية من الإنفاق<br>الصحي الحالي							
80.	Percent of government health budget spent on outpatient /inpatient care<br>نسبة ميزانية الصحة الحكومية التي تنفق<br>على رعاية المرضى الخارجيين /<br>المرضى الداخليين	1	2	1	2	1	2	
81.	Out of pocket expenditure per capita<br>الإنفاق من الجيب للفرد الواحد	1	2	1	2	1	2	
82.	Out-of-pocket payment for health as a share of current expenditure on health (%); men and women [gender-sensitive]<br>الدفع من الجيب للصحة كحصة من الإنفاق<br>الحالي على الصحة (٪) ؛ رجال ونساء	1	2	1	2	1	2	
83.	Poverty gap due to out-of-pocket payments<br>فجوة الفقر بسبب المدفوعات من الجيب	1	2	1	2	1	2	
84.	Proportion of the population with large household expenditure on health as a share of total household consumption or income [SDG 3.8.2]*<br>نسبة السكان الذين يعانون من إنفاق كبير على الأسرة<br>على الصحة كنسبة من إجمالي<br>استهلاك الأسرة أو دخلها*	1	2	1	2	1	2	
85.	External source of current spending on health (% of current expenditure on health)*<br>مصدر خارجي لإنفاق الحالي على الصحة<br>(٪ من الإنفاق الحالي على الصحة)*	1	2	1	2	1	2	
86.	Percentage of population covered by insurance; men and women by age and by gender-specific services [gender-sensitive]*<br>لمئوية للسكان المشمولين بالتأمين ؛ الرجال والنساء<br>حسب العمر وحسب<br>الخدمات الجنسانية*	1	2	1	2	1	2	
87.	% of government health spending allocated to PHC*	1	2	1	2	1	2	

© K2P Center (as part of IDRC-funded project to strengthen national health information systems)

		Important<br>مهم		Feasible<br>عملي		Actionable<br>فعال		NA
		Yes	No	Yes	No	High	Low	
	٪ من الإنفاق الحكومي على الصحة المخصصة للرعاية الصحية الأولية*							
88.	Government PHC spending as % of current PHC spending<br>الإنفاق على الرعاية الصحية الأولية الحكومية كنسبة مئوية من الإنفاق الحالي على الرعاية الصحية الأولية	1	2	1	2	1	2	
89.	Total expenditure on pharmaceuticals (% total expenditure on health)*<br>جمالي الإنفاق على الأدوية)٪ إجمالي الإنفاق على الصحة(*	1	2	1	2	1	2	
90.	Government expenditure on pharmaceuticals (per capita average exchange rate)<br>الإنفاق الحكومي على المستحضرات الصيدالنية)متوسط سعر الصرف للفرد(	1	2	1	2	1	2	
91.	Private expenditure on pharmaceuticals (per capita average exchange rate)<br>الإنفاق الخاص على المستحضرات الصيدالنية)متوسط سعر الصرف للفرد(	1	2	1	2	1	2	

I. Please list any additional indicators that are missing from this dimension which you believe are important for inclusion

يرجى ذكر أي مؤشرات إضافية مفقودة من هذا البعد تعتقد أنها مهمة لإلدراج

____

____

____

____

____

II. Please state your comments or areas for clarification on this dimension

يرجى ذكر تعليقاتك أو أي نقاط تود توضيحها بالنسبة لهذا البعد

____

____

____

____

____

© K2P Center (as part of IDRC-funded project to strengthen national health information systems)

Maternal, Child and Adolescent Health Indicators

© K2P Center (as part of IDRC-funded project to strengthen national health information systems)

Priority Setting Tool for Maternal, Child and Adolescent Health Indicators

أداة تحديد األولويات لمؤشرات صحة األم والطفل والمراهق

Instructions

التعليمات

You are kindly invited to complete the priority setting tool to assess a set of 137 indicators compiled from global/regional reference list of core indicators and Jordanian health system and National Health Strategy (NHS), for possible inclusion in the National Health Information System of Jordan.

The purpose of this exercise is to achieve consensus on a core set of indicators on maternal, child and adolescent health, that draw on globally recommended and standardized indicators and that are relevant and useful for decision-makers at each level of the health system. In line with the SDG's commitment to "leaving no one behind" and the articulation of the importance of addressing vulnerabilities and discrimination on a global scale, specific efforts will be invested to improve the gender sensitivity of current data as well as optimize indicators related to maternal, child and adolescent health.

Please note that what you will share with us will remain confidential and anonymous

يرجى من حضرتكم ملء أداة تحديد األولويات لتقييم مجموعة من المؤشرات)137(التي تم جمعها من مراجع اقليمية و عالمية ومن االستراتجية الوطنية للنظام الصحي األردني و ذلك بهدف دراسة إمكانية إدراجها في نظام المعلومات الصحية الوطني في األردن.

الهدف من هذا التمرين هو التوصل إلى إجماع بشأن مجموعة من المؤشرات التي تعنى بصحة األم والطفل والمراهق و المبنية على التوصيات و المعايير العالمية التي تفيد صناع القرار في كافة مستويات النظام الصحي األردني. انطالقا ً من االلتزام بتحقيق أهداف التنمية المستدامة التى تسعى للتصدي لكافة انواع التمييز و عدم المساواة سوف يتم بذل الجهود لتحسين الحساسية الجندرية للبيانات الحالية وكذلك لتحسين المؤشرات المتعلقة بصحة األم والطفل والمراهق.

يرجى مالحظة أن ما ستشاركونه معنا سيظل سريًا ومجهول الهوية

© K2P Center (as part of IDRC-funded project to strengthen national health information systems)

The indicators selected for inclusion encompass the following four **dimensions:**

**1. Health Status**		Indicators include core indicators including maternal, child and adolescent mortality by cause as well as core morbidity and fertility indicators
**2. Risk factor indicators**		Indicators include those relating to nutrition, environmental, behavioral, injuries and violence.
**3. Service coverage indicators**		Indicators reflect priorities across the spectrum of health services including reproductive, maternal, newborn, child and adolescent, HIV, TB, non-communicable diseases, mental health and substance abuse
**4. Health system indicators**		Indicators include indicators of health system inputs and outputs such as health facility density and distribution, health workforce, health information and quality and safety of care

تشمل المؤشرات المختارة األبعاد األربعة التالية:

**1. الحالة الصحية**	تشمل وفيات األمهات واألطفال والمراهقين حسب السبب, المرض و الخصوبة
**2. عوامل الخطر**	تشمل العوامل المتعلقة بالتغذية, البيئة, السلوك و العنف
**3. تغطية الخدمة**	تشمل الخدمات الصحية المتعلقة باإلنجاب, صحة األمهات، حديثي الوالدة ، األطفال والمراهقين ، فيروس نقص المناعة البشرية ، السل ، األمراض غير السارية ، الصحة العقلية واإلدمان
**4. النظام الصحي**	تشمل مدخالت و مخرجات النظام الصحي مثل كثافة المرافق الصحية وتوزيعها ، القوى العاملة في القطاع الصحي ، المعلومات الصحية و جودة وسالمة الرعاية الصحية.

Please specify the position that best suits you:

- ☐ Representative of government/policymaker
- ☐ Academia/researcher
- ☐ Health professional association
- ☐ Healthcare director/manager
- ☐ Representative of a non-governmental association
- ☐ Other:________________________________

يرجى اختيار المنصب المناسب لكم:

☐ ممثل عن منظمة حكومية /صانع قرار
☐ أكاديمية / باحث ☐
جمعية الصحة المهنية ☐ مدير
الرعاية الصحية ☐ ممثل عن منظمة
غير حكوميّة
☐ غيره:____________________________

© K2P Center (as part of IDRC-funded project to strengthen national health information systems)

Please rate each indicator based on the following criteria:

Criteria	Definitions	Scoring technique
Important	• The indicator reflects an issue that is important to the general population and relevant stakeholders in the health system;<br>• The indicator represents the most critical issues and priorities of the health systems	Please rate each indicator by selecting Yes or No
Feasible	The indicator is easy to measure in terms of:<br>• Data availability; or<br>• Minimal burden of data collection; or<br>• Minimal costs of data collection.	Please rate each indicator by selecting Yes or No
Actionable	• The indicator can help identify opportunities for health system improvement at the national and regional level<br>• The indicator provides information that is appropriate and useful for guiding policies and programs as well as for decision-making;	Please rate each indicator by selecting High or Low

- If an indicator is not relevant to your context, please select the “Not Applicable” (NA) option.
- Please note that the measurement tools for the selected indicators will be developed at the next stage of implementation and will be discussed with the respective stakeholders in Jordan.

يرجى تقييم كل مؤشر بناء ًعلى المعايير التالية:

المعيار	تعريف المعيار	كيفية التقييم
مـهم	• يعكس المؤشر مشكلة مهمة لعامة السكان وأصحاب المصلحة المعنيين في النظام الصحي<br>• يعكس المؤشر أولويات النظام الصحي<br>• المؤشر مهم لضبط و تنظيم السياسات و النظم الصحية	**يرجى تقييم المؤشر بالإجابة ب نعم أو ال**
عملي	من السهل قياس المؤشر من ناحية:<br>• توافر البيانات<br>• الحد األدنى من عبء جمع البيانات<br>• الحد األدنى من التكاليف لجمع البيانات	**يرجى تقييم المؤشر بالإجابة ب نعم أو ال**
فعال	يساعد المؤشر في تحديد فرص تحسين النظام الصحي على المستوى الوطني والإقليمي<br>• يوفر المؤشر معلومات مناسبة ومفيدة لترشيد السياسات والبرامج وكذلك لصنع القرار	**يرجى تقييم المؤشر بالإجابة ب جداً أو قليلً**

- إذا كان المؤشرغير مناسب لسياقك ، فيرجى بالإجابة ب "غير قابل للتطبيق".
- يرجى المالحظة أنه سيتم تطوير أدوات القياس للمؤشرات المختارة في المرحلة التالية من التنفيذ وستتم مناقشتها مع أصحاب المصلحة المعنيين في األردن.

© K2P Center (as part of IDRC-funded project to strengthen national health information systems)

Dimension 1: Health Status

الحالة الصحية

	Important مهم		Feasible عملي		Actionable فعال		NA
	Yes	No	Yes	No	High	Low	
**Mortality- Maternal and Child**							
1. Maternal mortality ratio (per 100 000 live births) [SDG 3.1.1] [gender-sensitive] معدل وفيات ألامهات (لكل 100,000 مولود حي)*	1	2	1	2	1	2	
2. Under-five mortality rate; boys and girls [SDG 3.2.1] [gender sensitive] * معدل وفيات ألاطفال دون سن الخامسة ؛ الفتيان والفتيات*	1	2	1	2	1	2	
3. Infant mortality rate; boys and girls [gender-sensitive]* معدل وفيات الرضع؛ الفتيان والفتيات*	1	2	1	2	1	2	
4. Neonatal mortality rate (per 1000 live births) [SDG 3.2]* معدل وفيات حديثي الوالدة (لكل 1000 مولود حي)*	1	2	1	2	1	2	
5. Distribution of causes of death among children aged <5 years (%)* توزيع أسباب الوفاة بين ألاطفال الذين تقل أعمارهم عن 5 سنوات (%)*	1	2	1	2	1	2	
6. Stillbirth rate (per 1000 total births) معدل والدة جنين ميت (لكل 1000 مولود)	1	2	1	2	1	2	
7. Perinatal mortality rate معدل وفيات الفترة المحيطة بالوالدة	1	2	1	2	1	2	
**Mortality- Adolescents**							
8. Adolescent mortality rate معدل وفيات المراهقين	1	2	1	2	1	2	

© K2P Center (as part of IDRC-funded project to strengthen national health information systems)

	Important مهم		Feasible عملي		Actionable فعال		NA
	Yes	No	Yes	No	High	Low	
9. Adolescent mortality rate from road traffic injuries معدل وفيات المراهقين من إصابات حوادث السير	1	2	1	2	1	2	
10. Adolescent mortality rate from suicide معدل وفيات المراهقين من االنتحار	1	2	1	2	1	2	
11. Adolescent mortality rate from homicide معدل وفيات المراهقين من القتل							
12. Adolescent mortality rate from violence معدل وفيات المراهقين من العنف							
13. Adolescent maternal mortality ratio (per 100,000 live births) سبب وفيات األمهات المراهقات)لكل 100000 مولودحي(	1	2	1	2	1	2	
**Morbidity-Maternal and Child**							
14. Preterm birth rate معدل المواليد قبل األوان	1	2	1	2	1	2	
15. Incidence of low birth weight; boys and girls [gender sensitive]* حدوث انخفاض الوزن عند الوالدة ؛ الفتيان والفتيات*	1	2	1	2	1	2	
16. Small for gestational age rate معدل صغير بالنسبة لسن الحمل							
17. Children under 5 years who are stunted [SDG 2.2.1]							
18. Prevalence of anemia in children انتشار فقر الدم عند األطفال	1	2	1	2	1	2	
19. Prevalence of HIV infection in pregnant women انتشار اإلصابة بفيروس نقص المناعة البشرية في النساء الحوامل	1	2	1	2	1	2	

© K2P Center (as part of IDRC-funded project to strengthen national health information systems)

	Important مهم		Feasible عملي		Actionable فعال		NA
	Yes	No	Yes	No	High	Low	
20. Prevalence of positive syphilis serology in pregnant women<br>معدل انتشار مرض الزهري في النساء الحوامل	1	2	1	2	1	2	
21. Neonatal preterm birth complications<br>مضاعفات الوالدة المبكرة عند األطفال حديثي الوالدة	1	2	1	2	1	2	
22. New cases of cancer per 100,000 children aged 0–14 years<br>حاالت سرطان جديدة لكل 100،000 طفل تتراوح أعمارهم بين صفر و 14 سنة	1	2	1	2	1	2	
23. Proportion of children aged 0–14 years with asthma as a long-term condition<br>نسبة األطفال الذين تتراوح أعمارهم بين صفر و 14 سنة والذين يعانون من الربو المزمن	1	2	1	2	1	2	
24. Prevalence of acute respiratory infection (ARI) in children under 5<br>انتشار عدوى الجهاز التنفسي الحادة عند األطفال دون سن الخامسة	1	2	1	2	1	2	
25. New cases of children aged 0–14 years receiving insulin on the National Diabetes Register as a rate per 100,000 children<br>معدل الحاالت الجديدة ألطفال تتراوح أعمارهم بين صفر و 14 سنة يتلقون األنسولين لكل 100،000 طفل في السجل الوطني للسكري	1	2	1	2	1	2	
26. Proportion of children aged 0–14 years with disability<br>نسبة األطفال الذين تتراوح أعمارهم بين صفر و 14 سنة ذوي اإلعاقة	1	2	1	2	1	2	
27. Proportion of children aged 6–14 years with mental health disorders (e.g. ADHD, depressive disorder, conduct disorder)<br>نسبة األطفال الذين تتراوح أعمارهم بين 6 و 14 سنة والذين يعانون من اضطرابات في الصحة النفسية	1	2	1	2	1	2	

© K2P Center (as part of IDRC-funded project to strengthen national health information systems)

	Important مهم		Feasible عملي		Actionable فعال		NA
	Yes	No	Yes	No	High	Low	
28. Prevalence of dental caries among children<br>انتشار تسوس االسنان بين االطفال							
29. Hospitalization rate for children aged 0–14 years for injuries from assault<br>معدل االستشفاء لألطفال الذين تتراوح أعمارهم بين صفر و 14 سنة بسبب إصابات ناجمة عن اعتداء	1	2	1	2	1	2	
30. Hospitalization rate for children aged 0–14 years for accidental injuries (poisoning, burns and scalds, pedestrian accidents)<br>معدل االستشفاء لألطفال الذين تتراوح أعمارهم بين صفر و 14 سنة بسبب اإلصابات العرضية)التسمم, الحروق وحوادث المشاة(	1	2	1	2	1	2	
31. Proportion of children with congenital anomalies (by type)<br>نسبة األطفال الذين يعانون من التشوهات الخلقية)حسب النوع(	1	2	1	2	1	2	
32. Epilepsy prevalence in children<br>انتشار الصرع عند األطفال	1	2	1	2	1	2	
33. Five-year relative survival rate for cancer in children aged 0–14 years<br>معدل النجاة النسبي لمدة خمس سنوات لمرض السرطان عند األطفال الذين تتراوح أعمارهم بين صفر و 14 عاًما	1	2	1	2	1	2	
**Morbidity- Adolescents**							
34. Prevalence of HIV infection among adolescents<br>انتشار اإلصابة بفيروس نقص المناعة البشرية بين المراهقين	1	2	1	2	1	2	
35. Prevalence of injuries among adolescents (sex and type of injury)<br>انتشار اإلصابات بين المراهقين)الجنس ونوع اإلصابة(	1	2	1	2	1	2	
36. Prevalence of mental disorders among adolescents<br>انتشار االضطرابات النفسية بين المراهقين	1	2	1	2	1	2	

	Important مهم		Feasible عملي		Actionable فعال		NA
	Yes	No	Yes	No	High	Low	
37. Prevalence of suicide attempts among adolescents<br>انتشار محاوالت االنتحار بين المراهقين	1	2	1	2	1	2	
38. Prevalence of anemia among adolescents<br>انتشار فقر الدم بين المراهقين	1	2	1	2	1	2	
39. DALY estimates due to communicable, maternal, and nutritional diseases in individuals aged 10-24<br>سنوات العمر المصححة بإحتساب مدد العجز بسبب ألمراض المعدية واألمومية والتغذوية لدى األفراد الذين تتراوح أعمارهم بين 10 و 24 عاماً	1	2	1	2	1	2	
40. DALY estimates due to injury and violence in individuals aged 10-24<br>سنوات العمر المصححة بإحتساب مدد العجز بسبب اإلصابة والعنف في األفراد الذين تتراوح أعمارهم بين 10-24	1	2	1	2	1	2	
41. DALY estimates due to non-communicable diseases in individuals aged 10-24<br>سنوات العمر المصححة بإحتساب مدد العجز بسبب ألمراض غير المعدية لدى األفراد الذين تتراوح أعمارهم بين 10 و 24 سنة	1	2	1	2	1	2	
**Fertility**							
42. Adolescent fertility rate (adolescent birth rate) [SDG 3.7.2]*<br>معدل خصوبة المراهقين)معدل والدة المراهقين(*							

© K2P Center (as part of IDRC-funded project to strengthen national health information systems)

I. Please list any additional indicators that are missing from this dimension which you believe are important for inclusion

يرجى ذكر أي مؤشرات إضافية مفقودة من هذا البعد تعتقد أنها مهمة للإدراج

II. Please state your comments or areas for clarification on this dimension

يرجى ذكر تعليقاتك أو أي نقاط تود توضيحها بالنسبة لهذا البعد

© K2P Center (as part of IDRC-funded project to strengthen national health information systems)

Dimension 2: Risk Factors and Behaviors

عوامل الخطر والسلوكيات

	Important مهم		Feasible عملي		Actionable فعال		NA
	Yes	No	Yes	No	High	Low	
**Maternal and Child**							
1. Exclusive breastfeeding rate 0–6 months of age* معدل الرضاعة الطبيعية الحصرية من عمر صفر إلى 6 أشهر*	1	2	1	2	1	2	
2. Percentage of men and women who know that using condoms and limiting sexual intercourse to one uninfected partner reduces the risk of contracting HIV* النسبة المئوية للرجال والنساء الذين يعرفون أن استخدام الواقي الذكري و حصر العالقة الجنسية بشريك واحد غير مصاب يقلل من خطر اإلصابة بفيروس نقص المناعة البشرية*	1	2	1	2	1	2	
3. Prevalence of anemia in women of reproductive age [gender sensitive]* انتشار فقر الدم عند النساء في سن اإلنجاب*	1	2	1	2	1	2	
4. Proportion of pregnant women who smoke نسبة النساء الحوامل الالواتي يدخن	1	2	1	2	1	2	
5. Proportion of children exposed to passive smoking نسبة األطفال المعرضين للتدخين السلبي	1	2	1	2	1	2	
6. Rate of children aged 0–14 years who have been the victim of physical assault معدل األطفال الذين تتراوح أعمارهم بين صفر و 14 سنة الذين وقعوا ضحية اعتداء جسدي	1	2	1	2	1	2	
7. Sexual violence against children [SDG 16.2.3] العنف الجنسي ضد األطفال	1	2	1	2	1	2	
8. Percentage of child labor نسبة عمالة األطفال	1	2	1	2	1	2	

© K2P Center (as part of IDRC-funded project to strengthen national health information systems)

	Important مهم		Feasible عملي		Actionable فعال		NA
	Yes	No	Yes	No	High	Low	
9. Proportion of children under 5 years of age who are developmentally on track in health, learning and psychosocial well-being [SDG 4.2.1]<br>نسبة األطفال الذين تقل أعمارهم عن 5 سنوات والذين هم ضمن مسار النمو الطبيعي في الصحة والتعلم والرفاه النفسي االجتماعي	1	2	1	2	1	2	
10. Proportion of women aged 15-49 years who make their own informed decisions regarding sexual relations, contraceptive use and reproductive health care [SDG 5.6.1]<br>سبة النساء الالواتي تتراوح أعمارهن بين 15 و 49 سنة و يتخذن قرارات مبنية على اساس من المعرفة بشأن العالقات الجنسية واستخدام<br>وسائل منع الحمل والرعاية الصحية اإلنجابية	1	2	1	2	1	2	
11. Percentage of women of reproductive age who have heard about at least three methods of family planning<br>ة المئوية للنساء في سن اإلنجاب الالواتي سمعن عن ثالث طرق لتنظيم األسرة على األقل	1	2	1	2	1	2	
12. Gender inequality index*<br>مؤشر عدم المساواة بين الجنسين*	1	2	1	2	1	2	
**Adolescents**							
13. Prevalence of iron deficiency anemia in 10–24-year-old<br>انتشار فقر الدم الناجم عن نقص الحديد في عمر 10-24 عاماً	1	2	1	2	1	2	
14. Prevalence of cannabis use among adolescents<br>انتشار تعاطي القنب بين المراهقين	1	2	1	2	1	2	
15. Percentage of adolescents who report early initiation of sexual activity<br>النسبة المئوية للمراهقين الذين يبلغون عن البدء المبكر للنشاط الجنسي	1	2	1	2	1	2	

© K2P Center (as part of IDRC-funded project to strengthen national health information systems)

	Important مهم		Feasible عملي		Actionable فعال		NA
	Yes	No	Yes	No	High	Low	
16. Condom use at most recent sex among adolescents استخدام الواقي الذكري في أحدث ممارسة للجنس بين المراهقين	1	2	1	2	1	2	
17. Percent of youth who believe they could seek sexual and reproductive health information and services if they needed them نسبة الشباب الذين يعتقدون ان بإمكانهم الحصول على المعلومات و الخدمات المتعلقة بالصحة الجنسية و االنجابية عندما يكونون بحاجة اليها	1	2	1	2	1	2	
18. Proportion of adolescents who report that their parents or guardians understand their problems or worries most of the Time نسبة المراهقين الذين يبلغون أن آبائهم أو أولياء أمورهم يتفهمون مشاكلهم أو مخاوفهم في معظم األوقات	1	2	1	2	1	2	
19. Proportion of adolescents who report that their parents or guardians really know what they are doing in their free time نسبة المراهقين الذين يبلغون أن آبائهم أو أولياء أمورهم يعرفون حقًا ما يفعلونه في أوقات فراغهم	1	2	1	2	1	2	
20. Percentage of adolescents who reported being bullied at least once in the last couple of months النسبة المئوية للمراهقين الذين بلغوا عن تعرضهم للتنمر مرة واحدة على األقل في الشهرين األخيرين	1	2	1	2	1	2	
21. Adolescent literacy rate, and by age category and sex (%) معدل معرفة القراءة والكتابة لدى المراهقين ، حسب الفئة العمرية والجنس)%(	1	2	1	2	1	2	
22. Adolescent school enrolment تسجيل المراهقين في المدارس	1	2	1	2	1	2	
23. Percentage of adolescents who do hazardous or heavy work النسبة المئوية للمراهقين الذين يقومون بأعمال خطرة أو ثقيلة	1	2	1	2	1	2	

© K2P Center (as part of IDRC-funded project to strengthen national health information systems)

	Important مهم		Feasible عملي		Actionable فعال		NA
	Yes	No	Yes	No	High	Low	
24. Percentage of women aged 20–24 years who were married before age 18 [gender-sensitive] النسبة المئوية للنساء الالواتي تتراوح أعمارهن بين 20 و 24 سنة المتزوجات قبل سن 18	1	2	1	2	1	2	

I. Please list any additional indicators that are missing from this dimension which you believe are important for inclusion

يرجى ذكر أي مؤشرات إضافية مفقودة من هذا البعد تعتقد أنها مهمة لإلدراج

____

____

____

____

____

II. Please state your comments or areas for clarification on this dimension

يرجى ذكر تعليقاتك أو أي نقاط تود توضيحها بالنسبة لهذا البعد

____

____

____

____

____

© K2P Center (as part of IDRC-funded project to strengthen national health information systems)

Dimension 3: Service Coverage and Use

تغطية الخدمة واالستخدام

	Important مهم		Feasible عملي		Actionable فعال		NA
	Yes	No	Yes	No	High	Low	
**Maternal and child**							
1. Availability of basic essential obstetric and newborn care facilities per 500,000 population توفر المرافق ألساسية لرعاية التوليد وحديثي الو الدة لكل 500,000 نسمة	1	2	1	2	1	2	
2. Availability of Emergency Obstetric and Newborn Care facilities per 500,000 population توفر مرافق الطوارىء لرعاية التوليد وحديثي الو الدة لكل 500,000 نسمة	1	2	1	2	1	2	
3. Percentage of health centers providing family planning method*	1	2	1	2	1	2	
4. Percentage of postpartum clients receiving modern family planning method from health centers* نسبة الزبائن الذين يحصلون من المراكز الصحية على وسيلة حديثة لتنظيم ألسرة بعد الوالدة*	1	2	1	2	1	2	
5. Referral rates for women with obstetric complications* معدالت إلحالة للنساء الالواتي يعانين من مضاعفات الوالدة*	1	2	1	2	1	2	
6. Met need for Emergency Obstetric and Newborn Care تلبية الحاجة للرعاية الطارئة للتوليد واالطفال حديثي الوالدة	1	2	1	2	1	2	
7. Demand for family planning satisfied with modern methods [SDG 3.7.1] تلبية الحاجة لتنظيم ألسرة بالوسائل الحديثة	1	2	1	2	1	2	
8. Contraceptive prevalence rate; men and women by age [gender-sensitive]* معدل انتشار وسائل منع الحمل)الرجال والنساء حسب العمر(*	1	2	1	2	1	2	

© K2P Center (as part of IDRC-funded project to strengthen national health information systems)

	Important مهم		Feasible عملي		Actionable فعال		NA
	Yes	No	Yes	No	High	Low	
9. Antenatal care (at least one visit) (%)* رعاية ما قبل الوالدة (زيارة واحدة على األقل) (٪)*	1	2	1	2	1	2	
10. Antenatal care (at least four visits) (%)* رعاية ما قبل الوالدة (على األقل أربع زيارات) (٪)*	1	2	1	2	1	2	
11. Antenatal care (eight or more visits) (%)* رعاية ما قبل الوالدة (ثماني زيارات أو أكثر) (٪)*	1	2	1	2	1	2	
12. Percentage of women who received prenatal care in the first trimester نسبة النساء الالواتي تلقين رعاية ما قبل الوالدة في الثلوث األول	1	2	1	2	1	2	
13. Proportion of pregnant women receiving iron and folic acid supplements نسبة النساء الحوامل الالوتي يتلقين مكمالت الحديد وحمض الفوليك	1	2	1	2	1	2	
14. Percentage of antenatal care attendees positive for syphilis who received treatment نسبة النين تلقوا العالج لمرض الزهري خالل رعاية ما قبل الوالدة	1	2	1	2	1	2	
15. Percentage of HIV+ pregnant women receiving ARVs for prevention of mother-to-child transmission (PMTCT)* نسبة المئوية للنساء الحوامل المصابات بفيروس نقص المناعة البشرية الالوتي يتلقين العقاقير المضادة للفيروسات لمنع انتقال العدوى من األم إلى الطفل*	1	2	1	2	1	2	

© K2P Center (as part of IDRC-funded project to strengthen national health information systems)

	Important مهم		Feasible عملي		Actionable فعال		NA
	Yes	No	Yes	No	High	Low	
16. Antenatal corticosteroid use for prevention of neonatal respiratory distress syndrome (RDS) in preterm labor (%)<br>استخدام كورتيكوستيرويد ما قبل الوالدة للوقاية من متالزمة الضائقة التنفسية لدى حديثي الوالدة في الوالدة المبكرة)٪(	1	2	1	2	1	2	
17. Proportion of live newborns with suspected or confirmed neonatal infection receiving antibiotics<br>نسبة حديثي الوالدة األحياء الذين يتلقون المضادات الحيوية كونه مشتبه به أو مؤكد انهم يعانون من عدوى حديثي الوالدة	1	2	1	2	1	2	
18. Percentage of births delivered in a health facility among all births in the population *<br>نسبة الوالدات السكانية التي تتم في مرافق صحية*	1	2	1	2	1	2	
19. Neonatal tetanus protection<br>حماية حديثي الوالدة من الكزاز	1	2	1	2	1	2	
20. Percentage of babies weighed at birth<br>نسبة األطفال الذين يتم قياس وزنهم عند الوالدة	1	2	1	2	1	2	
21. Percentage of newborns receiving essential newborn care<br>نسبة حديثي الوالدة الذين يتلقون الرعاية األساسية لحديثي الوالدة	1	2	1	2	1	2	
22. Percentage of newborns receiving thermal care (to prevent hypothermia)<br>نسبة حديثي الوالدة الذين يتلقون رعاية حرارية)لمنع انخفاض حرارة الجسم(	1	2	1	2	1	2	
23. Proportion of pregnant women with hypertension receiving antihypertensive drugs<br>نسبة النساء الحوامل المصابات بارتفاع ضغط الدم اللواتي يتلقين األدوية الخافضة للضغط	1	2	1	2	1	2	
24. Proportion of women diagnosed with pre-labor rupture of membranes (pPROM) receiving antibiotics<br>نسبة النساء المصابات بتمزق األغشية قبل المخاض اللواتي يتلقين المضادات الحيوية	1	2	1	2	1	2	

© K2P Center (as part of IDRC-funded project to strengthen national health information systems)

	Important مهم		Feasible عملي		Actionable فعال		NA
	Yes	No	Yes	No	High	Low	
25. Care of small and sick newborns<br>رعاية األطفال حديثي الوالدة المرضى	1	2	1	2	1	2	
26. Early postnatal care contact for mothers and infants (%)<br>الرعاية المبكرة لألمهات و الرضع بعد الوالدة	1	2	1	2	1	2	
27. Percentage of mothers who received counselling, support or messages on optimal breastfeeding at least once in the last year*<br>نسبة األمهات االلواتي تلقين المشورة أو الدعم أو الرسائل المتعلقة بالرضاعة الطبيعية المثلى على األقل مرة واحدة في العام الماضي*	1	2	1	2	1	2	
28. Percentage of postpartum service utilization by previous Antenatal clients with an identified expected delivery date*<br>نسبة استخدام خدمات ما بعد الوالدة من قبل زبائن ما قبل الوالدة السابقين الذين تم تحديد موعد والدتهم المتوقع*	1	2	1	2	1	2	
29. Proportion of service delivery points offering PAP smear tests<br>نسبة نقاط تقديم الخدمة التي تقدم فحص عنق الرحم	1	2	1	2	1	2	
30. Percentage of children under 5 years of age with suspected pneumonia taken to an appropriate health provider*<br>نسبة األطفال دون سن 5 سنوات المشتبه بإصابتهم بااللتهاب الرئوي الذين تم أخذهم إلى مقدم الرعاية الصحية المناسب*	1	2	1	2	1	2	
31. Percentage of children under 5 with diarrhea receiving oral rehydration therapy*<br>نسبة األطفال دون سن 5 سنوات المصابين بالسهال و الذين تلقوا عالج تعويض السوائل الفموي*	1	2	1	2	1	2	

© K2P Center (as part of IDRC-funded project to strengthen national health information systems)

	Important<br>مهم		Feasible<br>عملي		Actionable<br>فعال		NA
	Yes	No	Yes	No	High	Low	
32. Vitamin A supplementation coverage (Percent of children aged 6–59 months who received two age-appropriate doses of vitamin A in the past 12 months)*<br>تغطية مكمالت فيتامين)أ( )نسبة األطفال الذين تتراوح أعمارهم بين 6 و 59 شهًرا والذين تلقوا جرعتين من فيتامين)أ(مناسبين للعمر في األشهر الـ 12 الماضية(*	1	2	1	2	1	2	
33. Percentage of children screened for disability<br>نسبة األطفال الذين تم فحصهم بحثًا عن اإلعاقة	1	2	1	2	1	2	
**Adolescents**							
34. Health service utilization among adolescents<br>استخدام الخدمات الصحية بين المراهقين	1	2	1	2	1	2	
35. Prevalence of HIV testing among adolescents<br>انتشار اختبار فيروس نقص المناعة البشرية بين المراهقين	1	2	1	2	1	2	
36. Percentage of population aged 15-24 years with comprehensive correct knowledge of HIV/AIDS*<br>بة السكان الذين تتراوح أعمارهم بين 15 و 24 سنة والذين لديهم معرفة صحيحة شاملة بفيروس نقص المناعة البشرية / اإليدز*	1	2	1	2	1	2	
37. Percentage of 15–24 year-old with met needs for modern contraception<br>نسبة الذين تتراوح أعمارهم بين 15 و 24 عاماً الذين تمت تلبية احتياجاتهم لوسائل منع الحمل الحديثة	1	2	1	2	1	2	
38. Percentage of service delivery points providing youth friendly services<br>نسبة نقاط تقديم الخدمات التي تقدم خدمات مالئمة للشباب	1	2	1	2	1	2	
39. Percentage of facilities with health service providers trained in the provision of adolescent health services<br>المرافق الصحية التي لديها مقدمي خدمات صحية مدربين على تقديم الخدمات الصحية للمراهقين	1	2	1	2	1	2	

© K2P Center (as part of IDRC-funded project to strengthen national health information systems)

	Important مهم		Feasible عملي		Actionable فعال		NA
	Yes	No	Yes	No	High	Low	
40. Percentage of adolescents who are aware of what adolescent health services are being provided, where and when they are provided and how to obtain them<br>ة المراهقين الذين يدركون ما هي الخدمات الصحية المقدمة للمراهقين ، وأين ومتى يتم تقديمها وكيفية الحصول عليها	1	2	1	2	1	2	

I. Please list any additional indicators that are missing from this dimension which you believe are important for inclusion

يرجى ذكر أي مؤشرات إضافية مفقودة من هذا البعد تعتقد أنها مهمة لإلدراج

____

____

____

____

____

____

II. Please state your comments or areas for clarification on this dimension

يرجى ذكر تعليقاتك أو أي نقاط تود توضيحها بالنسبة لهذا البعد

____

____

____

____

____

© K2P Center (as part of IDRC-funded project to strengthen national health information systems)

Dimension 4: Health System الصحي

النظام

	Important مهم		Feasible عملي		Actionable فعال		NA
	Yes	No	Yes	No	High	Low	
**Service Quality and Safety**							
1. Proportion of antenatal care visits at which blood pressure was measured نسبة زيارات الرعاية السابقة للوالدة التي تم فيها قياس ضغط الدم	1	2	1	2	1	2	
2. Proportion of women with severe pre-eclampsia or eclampsia treated with magnesium sulfate injection اللواتي نسبة النساء المصابات بتسمم الحمل تمت معالجتهن بحقن كبريتات المغنيسيوم	1	2	1	2	1	2	
3. Proportion of women receiving oxytocin within 1 minute of birth of infant نسبة النساء االلواتي يتلقين األوكسيتوسين خالل دقيقة واحدة من والدة الرضيع	1	2	1	2	1	2	
4. Proportion of women with prolonged labor نسبة النساء ذوات المخاض الطويل	1	2	1	2	1	2	
5. Intrapartum stillbirth rate معدل والدة جنين ميت أثناء الوالدة	1	2	1	2	1	2	
6. Proportion of women with severe systemic infection or sepsis in postnatal period, including readmissions نسبة النساء المصابات بعدوى الدم الحادة أو تعفن الدم في فترة ما بعد الوالدة، بما في ذلك النساء اللواتي أعيد إدخالهم إلى المستشفى	1	2	1	2	1	2	
7. Proportion of women who developed severe post-partum hemorrhage (PPH) نسبة النساء االلواتي أصبن بنزيف حاد بعد الوالدة	1	2	1	2	1	2	

© K2P Center (as part of IDRC-funded project to strengthen national health information systems)

	Important مهم		Feasible عملي		Actionable فعال		NA
	Yes	No	Yes	No	High	Low	
8. Percentage of births attended by skilled health personnel * نسبة الوالدات التي تتم بحضور عاملين صحيين لديهم المهارة*	1	2	1	2	1	2	
9. Percentage of deliveries by female adolescents attended by skilled birth attendants نسبة الوالدات للمراهقات التي تتم بحضور عاملين صحيين لديهم المهارة	1	2	1	2	1	2	
10. Proportion of newborns who received all four elements of essential care:<br>• immediate and thorough drying<br>• immediate skin-to-skin contact<br>• delayed cord clamping<br>• initiation of breastfeeding in the first hour<br>نسبة المواليد الجدد الذين تلقوا جميع عناصر الرعاية األساسية األربعة:<br>• التجفيف الفوري والشامل<br>• مالمسة الجلد مباشرة للجلد<br>• تأخير قطع الحبل السري<br>• بدء الرضاعة الطبيعية في الساعة األولى	1	2	1	2	1	2	
11. Proportion of health facilities in which kangaroo mother care is operational, by level of facility نسبة المرافق الصحية التي تقدم رعاية أم الكنغر, حسب مستوى المرفق	1	2	1	2	1	2	
12. Facility neonatal mortality rate disaggregated by birth weight: > 4000 g, 2500–3999 g, 2000–2499 g, 1500–1999 g, < 1500 g معدل وفيات حديثي الوالدة في المنشأة مصنفة حسب وزن الوالدة:< 4000 غرام ، 2500-3999 جم ، 2000-2499 جم ، 1500-1999 جم ، >1500 جم	1	2	1	2	1	2	
13. Newborn resuscitation (%) إنعاش حديثي الوالدة)٪(	1	2	1	2	1	2	

© K2P Center (as part of IDRC-funded project to strengthen national health information systems)

	Important<br>مهم		Feasible<br>عملي		Actionable<br>فعال		NA
	Yes	No	Yes	No	High	Low	
14. Proportion of health facilities offering maternity services certified by the Baby-friendly Hospital Initiative<br>نسبة المرافق الصحية التي تقدم خدمات مة و المصدقة من قبل مبادرة المستشفيات الصديقة لألطفال	1	2	1	2	1	2	
15. Proportion of health facilities that have stock-outs of essential lifesaving medicines for mothers and newborns in a specified period<br>نسبة المرافق الصحية التي ينفذ لديها مخزون ألدوية ألساسية المنقذة للحياة لألمهات واألطفال حديثي الوالدة في فترة محددة	1	2	1	2	1	2	
16. Proportion of health facilities with safe, uninterrupted oxygen supply in childbirth, neonatal and pediatric wards<br>نسبة المرافق الصحية التي تحتوي على إمدادات آمنة من األكسجين دون انقطاع في أجنحة الوالدة وحديثي الوالدة واألطفال	1	2	1	2	1	2	
17. Maternal death review coverage (percentage of maternal deaths occurring in the facility that were audited)<br>تغطية مراجعة وفاة األم)النسبة المئوية لوفيات األمهات التي تحدث في المنشأة التي تم تدقيقها(	1	2	1	2	1	2	
18. Neonatal death review coverage (%<br>تغطية مراجعة وفيات حديثي الوالدة)٪(	1	2	1	2	1	2	
19. Facility stillbirth review (audit) in place<br>مراجعة موت الجنين عند الوالدة في المنشأة)التدقيق(	1	2	1	2	1	2	

© K2P Center (as part of IDRC-funded project to strengthen national health information systems)

	Important مهم		Feasible عملي		Actionable فعال		NA
	Yes	No	Yes	No	High	Low	
20. Birth trauma rate in neonate per 1,000 live birth معدل الصدمات عند حديثي الوالدة لكل 1000 مولود حي	1	2	1	2	1	2	
21. Obstetric trauma, vaginal delivery (with or without instrument) صدمة الوالدة الطبيعية (مع أو بدون أداة)	1	2	1	2	1	2	
22. Percentage of births delivered by caesarean section* نسبة الوالدات القيصرية*	1	2	1	2	1	2	
23. Percentage of health-care providers following evidence-based guidelines and protocols in delivering care to adolescents نسبة مقدمي الرعاية الصحية الذين يتبعون ت والبروتوكوالت القائمة على األدلة في تقديم الرعاية للمراهقين	1	2	1	2	1	2	
**Financing**							
24. Percentage of total health expenditure spent on reproductive, maternal, newborn, and child health النسبة المئوية من إجمالي اإلنفاق الصحي اقه على الصحة اإلنجابية وصحة األم والوليد والطفل	1	2	1	2	1	2	
25. Reproductive, maternal, newborn, and child health (RMNCH) expenditure by source اإلنفاق على الصحة اإلنجابية وصحة األم والوليد والطفل حسب المصدر	1	2	1	2	1	2	
26. Percentage of government health budget allocated to adolescent health. النسبة المئوية للميزانية الصحية الحكومية المخصصة لصحة المراهقين	1	2	1	2	1	2	
**Health workforce**							

© K2P Center (as part of IDRC-funded project to strengthen national health information systems)

	Important مهم		Feasible عملي		Actionable فعال		NA
	Yes	No	Yes	No	High	Low	
27. Percentage and distribution of health workers trained to provide reproductive, maternal and child health<br>بة المئوية وتوزيع العاملين الصحيين المدربين على توفير الصحة اإلنجابية وصحة األم والطفل	1	2	1	2	1	2	
28. Density of midwives, by district (by births)*<br>كثافة القابالت ، حسب المقاطعة)حسب الوالدات(*	1	2	1	2	1	2	
29. . Percentage and distribution of health workers trained to provide adolescent and youth-friendly services<br>النسبة المئوية وتوزيع العاملين الصحيين المدربين على تقديم خدمات مالئمة للشباب والمراهقين	1	2	1	2	1	2	
**Supply chain**							
30. Percentage of maternal lifesaving commodities in essential medicine list<br>النسبة المئوية للسلع المنقذة للحياة األم في قائمة األدوية األساسية	1	2	1	2	1	2	
31. Percentage of newborn lifesaving commodities in essential medicine list<br>النسبة المئوية للسلع المنقذة لحياة حديثي الوالدة في قائمة األدوية األساسية	1	2	1	2	1	2	

© K2P Center (as part of IDRC-funded project to strengthen national health information systems)

I. Please list any additional indicators that are missing from this dimension which you believe are important for inclusion

يرجى ذكر أي مؤشرات إضافية مفقودة من هذا البعد تعتقد أنها مهمة لإلدراج

II. Please state your comments or areas for clarification on this dimension

يرجى ذكر تعليقاتك أو أي نقاط تود توضيحها بالنسبة لهذا البعد

Thank you

شكرا جزيال

REFUGEE HEALTH INDICATORS

Indicators Refugee for Tool Setting Priority

أداة تحديد الأولويات لمؤشرات

Instructions

التعليمات

You are kindly invited to complete the priority setting tool to assess a set of 127 health and health-related indicators for *refugees* which have been compiled from the international literature as well as from regional/national reports, for possible inclusion in the National Health Information System of Jordan.

The purpose of this exercise is to achieve consensus on a core set of health and health-related indicators for refugees that are relevant and useful for decision-makers in Jordan.

Please note that what you will share with us will remain confidential and anonymous

Arabic translation

The indicators selected for inclusion encompass the following four **dimensions:**

**1.**	**Health Status**	Indicators include core indicators including maternal, child and adolescent mortality by cause as well as core morbidity and fertility indicators
**2.**	**Risk factor indicators**	Indicators include those relating to nutrition, environmental, behavioral, injuries and violence.
**3.**	**Service coverage indicators**	Indicators reflect priorities across the spectrum of health services including reproductive, maternal, newborn, child and adolescent, HIV, TB, non-communicable diseases, mental health and substance abuse
**4.**	**Health system indicators**	Indicators include indicators of health system inputs and outputs such as health facility density and distribution, health workforce, health information and quality and safety of care

تشمل المؤشرات المختارة الأبعاد الأربعة التالية:

**1. الحالة الصحية**	تشمل وفيات الأمهات والأطفال والمراهقين حسب السبب, المرض والخصوبة
**2. عوامل الخطر**	تشمل العوامل المتعلقة بالتغذية, البيئة, السلوك و العنف

3. تغطية الخدمة	تشمل الخدمات الصحية المتعلقة بالإنجاب, صحة ألمهات، حديثي الوالدة ، ألطفال والمراهقين ، فيروس نقص المناعة البشرية ، السل ، ألمراض غير السارية ، الصحة العقلية والإدمان
4. النظام الصحي	تشمل مدخالت و مخرجات النظام الصحي مثل كثافة المرافق الصحية وتوزيعها ، القوى العاملة في القطاع الصحي ، المعلومات الصحية و جودة وسالمة الرعاية الصحية.

Please specify the position that best suits you:

☐ Representative of government/policymaker
☐ Academia/researcher
☐ Health professional association
☐ Healthcare director/manager
☐ Representative of a non-governmental association
☐ Other:______________________________

يرجى اختيار المنصب المناسب لكم:

☐ ممثل عن منظمة حكومية /صانع قرار
☐ أكاديمية / باحث
☐ جمعية الصحة المهنية
☐ مدير الرعاية الصحية
☐ ممثل عن منظمة غير حكوميّة
☐ غيره:_______________

Please rate each indicator based on the following criteria:

- If an indicator is not relevant to your context, please select the "Not Applicable" (NA) option.
- Please note that the measurement tools for the selected indicators will be developed at the next stage of implementation and will be discussed with the respective stakeholders in Jordan.

**Criteria**	**Definitions**	**Scoring technique**
Important	• The indicator reflects an issue of concern for stakeholders working with refugees;<br>• The indicator represents an important aspect of refugee health<br>• The indicator can help ease pressures on host countries<br>• The indicator can help give early warning of pending changes<br>• The indicator is acceptable to stakeholders	Please rate each indicator by selecting Yes or No
Feasible	The indicator is easy to measure in terms of:<br>• Data availability and accessibility<br>• Minimal burden (financial, administrative) of data collection & reporting	Please rate each indicator by selecting Yes or No

	• Disaggregation (by geographical region, sex, income or special population group)<br>• Interpretation and communication	
Actionable	• The indicator is easily understood and applied by potential users<br>• The indicator is relevant to policy and management needs<br>• The indicator provides information that is useful to decision-makers and can be acted upon at various levels (local, national and international).<br>• The indicator can lead to set of targets or thresholds<br>• Intervention is possible when improvement is needed (i.e. information provided by indicator will galvanize action)	Please rate each indicator by selecting High or Low

يرجى تقييم كل مؤشر بناء ًعلى المعايير التالية:

- إذا كان المؤشرغير مناسب لسياقك ، فيرجى بالإجابة ب "غير قابل للتطبيق".
- يرجى المالحظة أنه سيتم تطوير أدوات القياس للمؤشرات المختارة في المرحلة التالية من التنفيذ وستتم مناقشتها مع أصحاب المصلحة المعنيين في األردن.

المعيار	تعريف المعيار	كيفية التقييم
مـهم		يرجى تقييم المؤشر بالإجابة ب نعم أو ال
عملي		يرجى تقييم المؤشر بالإجابة ب نعم أو ال
فعال		يرجى تقييم المؤشر بالإجابة ب جداً أو قليلاً

Dimension 1: Health Status
الحالة الصحية

	Important مهم		Feasible عملي		Actionable فعال		NA
	Yes	No	Yes	No	High	Low	
**Mortality**							
1. Crude death rate	1	2	1	2	1	2	
2. Mortality rate by age group and gender							
3. Maternal mortality ratio (per 100 000 live births) * معدل وفيات ألمهات)لكل 100,000 مولود حي(	1	2	1	2	1	2	
4. Under-five mortality rate; boys and girls معدل وفيات ألطفال دون سن الخامسة ؛ الفتيان والفتيات	1	2	1	2	1	2	
5. Distribution of causes of death among children aged <5 years (%)* توزيع أسباب الوفاة بين ألطفال الذين تقل أعمارهم عن 5 سنوات)%(							
6. Infant mortality rate; boys and girls * معدل وفيات الرضع؛ الفتيان والفتيات	1	2	1	2	1	2	
7. Neonatal mortality rate (per 1000 live births)* معدل وفيات حديثي الوالدة)لكل 1000 مولود حي(	1	2	1	2	1	2	
8. Perinatal mortality rate * معدل وفيات الفترة المحيطة بالوالدة	1	2	1	2	1	2	
9. Stillbirth rates (per 1000 birth)							
**Morbidity**							
10. Incidence of communicable diseases among refugees							
11. Prevalence of acute respiratory infection (ARI) in children under 5 *							

	Important مهم		Feasible عملي		Actionable فعال		NA
	Yes	No	Yes	No	High	Low	
12. Prevalence of diarrhea among children under 5 *							
13. % of refugees with a chronic health condition with a need for medical follow-up, by condition and gender							
14. Percentage of Syrian refugees having a lasting health problem (Psychological illness, Physical illness, Injury, Handicap/functional difficulty, Age-related). By nature of problem, reporting domain, gender and age							
15. Prevalence of diabetes in adults*	1	2	1	2	1	2	
16. Preterm birth rate<br>معدل المواليد قبل األوان	1	2	1	2	1	2	
17. Low birth weight; boys and girls *<br>حدوث انخفاض الوزن عند الوالدة ؛ الفتيان والفتيات	1	2	1	2	1	2	
18. Prevalence of anemia in children *<br>انتشار فقر الدم عند األطفال	1	2	1	2	1	2	
19. Prevalence of anemia in women of reproductive age*							
20. Prevalence of wasting among children under 5 years of age							
21. Prevalence of stunting among children under 5 years of age							
22. Incidence of complications due to unsafe or spontaneous abortion (ICUSA)							
**Fertility**							
23. Crude Birth Rate							
24. Adolescent fertility rate (adolescent birth rate) *<br>معدل خصوبة المراهقين)معدل والدة المراهقين(							

	Important<br>مهم		Feasible<br>عملي		Actionable<br>فعال		NA
	Yes	No	Yes	No	Hig h	Low	
25. Total fertility rate expressed per 1,000 women age 15-44 *<br>معدل الخصوبة الكلي							
26. Median age at first birth among women age 25-49 and age 30-49, according to background characteristics							
27. Mean number of children ever born to women age 40-49*							

I. Please list any additional indicators that are missing from this dimension which you believe are important for inclusion

يرجى ذكر أي مؤشرات إضافية مفقودة من هذا البعد تعتقد أنها مهمة لإلدراج

____

____

____

____

____

____

II. Please state your comments or areas for clarification on this dimension

يرجى ذكر تعليقاتك أو أي نقاط تود توضيحها بالنسبة لهذا البعد

____

____

____

____

____

____

Dimension 2: Risk Factors and Behaviors

عوامل الخطر والسلوكيات

	Important مهم		Feasible عملي		Actionable فعال		NA
	Yes	No	Yes	No	High	Low	
**Risk Factors**							
1. Exclusive breastfeeding rate 0–6 months of age							
2. Early child development index *							
3. Incidence of sexually transmitted diseases among refugee population							
4. Proportion of sexually transmitted infections among adolescents							
5. Infant HIV positive rate							
6. Condom distribution rate per month							
7. Condom use among adolescents							
8. Availability of culturally appropriate menstrual protection materials for women and girls (%)							
9. Number of cases of sexual violence reported to health services							
10. Percentage of ever-married women aged 15-49 who experienced emotional, physical, or sexual violence by their current or most recent husband in the past 12 months *							
11. Number of reported rape cases							
12. Knowledge of sexually transmitted infections (STIs) among ever married women aged 15-49*							
13. Knowledge of sexually transmitted infections (STIs) among men*							
14. Number of children fully vaccinated							
15. Prevalence of overweight and obesity in adults disaggregated by age and sex *							

	Important مهم		Feasible عملي		Actionable فعال		NA
	Yes	No	Yes	No	High	Low	
16. Prevalence of overweight and obesity in adolescents disaggregated by age and sex*							
17. Prevalence of overweight & obesity among children under 5 years of age							
18. Percentage of ever-married women and men aged 15-49 who smoke various tobacco products (Cigarettes, Water pipe and any type of tobacco) *							
19. Percentage of men aged 15-49 by smoking frequency*							
20. Nutritional status of women aged 15-49 (Percentage of height < 145 cm and specific BMI levels)							
21. Prevalence of moderate to severe acute malnutrition in children under 5 years of age							
22. Severity of food insecurity							
23. Incidence of work-related accidents and illness in the past 12 months and time absent from work due to such incidents (percentage of employed Syrian refugees aged 15 and above) *							
**Demographic and socioeconomic determinants**							
24. Distribution of refugees aged (13 years and above) by Marital Status, Sex and Governorate *							
25. Number of registered refugees inside and outside the camps *							
26. Distribution of registered refugees by age group, gender and districts *							
27. Educational attainment by level age and sex *							
28. Percentage of refugees aged 6 to 25 currently enrolled in formal education*							

	Important مهم		Feasible عملي		Actionable فعال		NA
	Yes	No	Yes	No	High	Low	
29. Percent of children under age 18 living with both parents*							
30. Percent of children under age 18 not living with either parent but both are alive*							
31. Percent distribution of ever-married women and men aged 15-49 by employment status (currently employed, not currently employed and never employed)							
32. Employment status by reporting domain and gender. (out of percentage of all employed refugees aged 15 and above)							
33. Child employment (and school attendance). By gender and age. (out of percentage of refugee children aged 9 to 14)							
34. % of women aged 20-24 years who were married before age 18 *							
35. Percentage of children under 5 whose births are registered and who had a birth certificate							
36. Main income sources in the past 12 months, by reporting domain*							

I. Please list any additional indicators that are missing from this dimension which you believe are important for inclusion

يرجى ذكر أي مؤشرات إضافية مفقودة من هذا البعد تعتقد أنها مهمة لإلدراج

____

____

____

____

____

II. Please state your comments or areas for clarification on this dimension

يرجى ذكر تعليقاتك أو أي نقاط تود توضيحها بالنسبة لهذا البعد

____

____

____

____

Dimension 3: Service Coverage and Use
تغطية الخدمة والاستخدام

	Important مهم		Feasible عملي		Actionable فعال		NA
	Yes	No	Yes	No	High	Low	
1. Percentage of new refugee arrivals screened for malnutrition, epidemic-prone diseases							
2. Treatment of symptoms of acute respiratory infection (ARI) for children under 5							
3. Percentage of children under 5 with diarrhea who were given Oral Rehydration Therapy *							
4. Antibiotic treatment of pneumonia for children under age of 5 (%)							
5. Recovery rate among children admitted with severe acute malnutrition							
6. Percent distribution of currently married women age 15-49 by contraceptive method currently used (Modern and Traditional) *							
7. Breast cancer exam *							
8. Cervical cancer screening for women aged 30-49 (% of PAP Test) *							
9. Utilization rates of mental health services (compared with general population)							
10. Antenatal care (at least one visit) (%) * رعاية ما قبل الوالدة)زيارة واحدة على الأقل( )٪(	1	2	1	2	1	2	

	Important مهم		Feasible عملي		Actionable فعال		NA
	Yes	No	Yes	No	High	Low	
11. Antenatal care (at least four visits) (%) * رعاية ما قبل الوالدة)على األقل أربع زيارات( )٪(	1	2	1	2	1	2	
12. Proportion of pregnant women receiving iron and folic acid supplements * نسبة النساء الحوامل الالواتي يتلقين مكمالت الحديد وحمض الفوليك	1	2	1	2	1	2	
13. Percentage of births delivered in a health facility among all births in the population * نسبة الوالدات السكانية التي تتم في مرافق صحية	1	2	1	2	1	2	
14. Percentage of live births delivered by C-section *							
15. Percentage of babies weighed at birth * نسبة األطفال الذين يتم قياس وزنهم عند الوالدة	1	2	1	2	1	2	
16. Percentage of newborns receiving essential newborn care * نسبة حديثي الوالدة الذين يتلقون الرعاية األساسية لحديثي الوالدة	1	2	1	2	1	2	
17. Timing of first postnatal check for the mother *							
18. Percentage of mothers who received counselling, support or messages on optimal breastfeeding at least once in the last year * نسبة األمهات الالواتي تلقين المشورة أو الدعم أو لرضاعة الطبيعية المثلى على األقل مرة واحدة في العام الماضي	1	2	1	2	1	2	
19. Neonatal tetanus protection * حماية حديثي الوالدة من الكزاز							
20. Percentage of mothers aged 15-49 receiving two or more tetanus toxoid injections during the pregnancy for the last live birth *							

	Important مهم		Feasible عملي		Actionable فعال		NA
	Yes	No	Yes	No	High	Low	
21. % of postpartum clients receiving modern family planning method from health centers *							
22. Demand for family planning satisfied with modern methods *							
23. Unmet need for family planning *							
24. Vitamin A supplementation coverage (Percent of children aged 6–59 months who received two age-appropriate doses of vitamin A in the past 12 months) * تغطية مكمالت فيتامين)أ( )نسبة األطفال الذين تتراوح أعمارهم بين 6 و 59 شهًرا والذين تلقوا جرعتين من فيتامين)أ(مناسبين للعمر في األشهر الـ 12 الماضية(	1	2	1	2	1	2	
25. Pregnant women known to be HIV positive have received ARV drugs for PMTCT							
26. Comprehensive knowledge about HIV among ever married young women and young men aged 15-24							
27. Coverage of HIV Rapid Tests for Safe Blood Transfusion							
28. Proportion of rape survivors who receive HIV post-exposure prophylaxis (PEP) within 72 h of an incident occurring.							
29. Prevention of mother-to-child transmission (PMTCT) coverage							
30. Coverage of induced abortion							
31. Coverage of STI/STD screening							
32. Syndromic treatment of sexually transmitted infections (STIs) to patients presenting with symptoms							
33. Availability of clinical care for survivors of sexual violence							
34. Emergency obstetric care (EmOC) services availability							
35. Emergency obstetric care (EmOC) services utilization							

	Important مهم		Feasible عملي		Actionable فعال		NA
	Yes	No	Yes	No	High	Low	
36. There is at least one health facility with Comprehensive Emergency Obstetric and Newborn Care (CEmOC) and newborn care/500,000 population							
37. Obstetric referral rates, defined as the number of documented obstetric referrals by camp health staff to a referral hospital per 100 000 live births							
38. Availability of supplemental food programs for pregnant women							
39. Proportion of population using safely managed drinking water services *							
40. Proportion of population using safely managed sanitation services, including a hand-washing facility with soap and water *							
41. Immunization coverage rate by vaccine for each vaccine in the national schedule *							
42. Percentage of children age 12-23 months who ever had a vaccination card							
43. Percentage of children age 24-35 months who ever had a vaccination card							
44. Percentage of members who received outpatient care from a health facility by type of facility (public and private) where outpatient care was provided most recently							

I. Please list any additional indicators that are missing from this dimension which you believe are important for inclusion

يرجى ذكر أي مؤشرات إضافية مفقودة من هذا البعد تعتقد أنها مهمة لإلدراج

____

____

____

____

____

II. Please state your comments or areas for clarification on this dimension

يرجى ذكر تعليقاتك أو أي نقاط تود توضيحها بالنسبة لهذا البعد

Dimension 4: Health System النظام الصحي

	Important مهم		Feasible عملي		Actionable فعال		NA
	Yes	No	Yes	No	High	Low	
**Service Quality and Safety**							
1. Annual number of consultations at primary health care facilities by health care worker (Physicians/ Nurses/ Dentists)							
2. Refugee access to health facilities *							
3. Problems in accessing health care *							
4. Proportion of births attended by skilled health personnel							
5. Percentage of refugees requiring hospital care in the last 6 months *							
6. Percentage of refugees with chronic health failure who receive medical follow-up by main type of provider, and the percentage who do not receive such follow-up*							
7. Level of satisfaction with health services after consultation with acute illness in the past 12 month (percentage of individuals who visited a medical doctor) *							

	Important مهم		Feasible عملي		Actionable فعال		NA
	Yes	No	Yes	No	High	Low	
8. All primary health care facilities have adequate medication for continuation of treatment of individuals with NCDs who were receiving treatment before the emergency							
9. All primary health care facilities have antimicrobials to provide syndromic management to patients presenting with symptoms of a sexually transmitted infection							
10. Availability of antiretroviral medicines to continue treatment for people already on antiretroviral including for prevention of mother to child transmission							
11. All health facilities have trained staff, sufficient supplies and equipment for clinical management of rape survivor services based on national or WHO protocols							
12. All health facilities have trained staff and systems for the management of mental health problems							
13. Birth registration *							
14. Percentage increase in funds for health sector refugee response							
15. Per capita expenditure on health ($)							
16. Out of pocket healthcare expenditure							
17. Percentage of ever-married women age 15-49 with specific types of health insurance coverage, and percentage with any health insurance, according to background characteristics							

	Important<br>مهم		Feasible<br>عملي		Actionable<br>فعال		NA
	Yes	No	Yes	No	High	Low	
18. Coverage of health insurance							

I. Please list any additional indicators that are missing from this dimension which you believe are important for inclusion

يرجى ذكر أي مؤشرات إضافية مفقودة من هذا البعد تعتقد أنها مهمة لإلدراج

____

____

____

____

____

____

II. Please state your comments or areas for clarification on this dimension

يرجى ذكر تعليقاتك أو أي نقاط تود توضيحها بالنسبة لهذا البعد

____

____

____

____

____

____

Thank you

شكرا جزيال
