# Supplementary material for: Promoting data-driven decision-making in Jordan: strengthening national health information system and achieving consensus on core set of health system indicators
Source: Reprod Health. 2025 May 31;22(Suppl 1):72. doi: 10.1186/s12978-025-01988-1 (PMC12125760; doi:10.1186/s12978-025-01988-1)
Supplement: Supplementary file 2 — Additional file 2. Jordan action plan [file 12978_2025_1988_MOESM2_ESM.pdf]

Action Plan for Strengthening the National Health Information Systems to Promote Data-Informed Decision-Making and Achieve SDGs in Jordan

Strategic Area I: Governance and Planning

Initiative	Recommended Actions	Short-Medium term	Long term	Leading department/ entity	Supporting partner (s)	Requirements/ Resources/ Pre-requisites	Expected Outcome (s)
**I- Update and implement health information system policies, laws and regulations**	**1-** Develop a national HIS policy that outlines a deliberate system of principles and documented purposes/objectives to guide decisions and achieve better HIS outcomes.	x		Minister;<br><br>Secretary General	MOH Department Heads		This will ensure that the required legislative, ethical, regulatory, and policy frameworks are in place to enable an effective national health information system and pave the path for appropriate use of e-health solutions in Jordan.
	**2-** Reinforce existing efforts to adopt unified electronic medical records at the facility level, and electronic health records at the national level.		x	Minister;<br><br>Electronic Transformation and Information Technology Directorate	Royal Medical Services;<br><br>Health Facilities;<br><br>International Donors	Funding sources for eHealth;<br><br>Multilingualism in eHealth;<br><br>eHealth capacity building;<br><br>the existence of unique patient identifier at the national level	Improved accuracy, legibility, structuring, reliability and retrieval of information; Transparency of actions with audit trails and tracking; Fewer errors in drug prescribing, dispensing and administration; Evidence-based decision support with improved adherence to clinical guidelines; and easier investigation of incidents and discrepancies.
**II- Develop an explicit HIS strategy or plan and integrate it within the broader health sector strategy**	**1-** Develop a national HIS strategy that defines strategic priorities, cycles and budgets for health information; describes expectations both for producers and users of HIS-generated data at all levels of the health system; and documents the guiding principles, mission, and vision for HIS. Ensure that the strategy for the health information system is integrated with and responds to the information needs of overall health sector strategic plan.	x		Minister	MOH Department Heads;<br><br>Electronic Transformation and Information Technology Directorate	The strategy or plan is informed by ongoing assessments of HIS performance that identify gaps and needs. The baseline assessment that was conducted can serve as a starting point.	This will demonstrate explicit commitment to strengthening health information system as well as ensure its sustainability.

Initiative	Recommended Actions	Short-Medium term	Long term	Leading department/ entity	Supporting partner (s)	Requirements/ Resources/ Pre-requisites	Expected Outcome (s)
	**2-** Identify changes in roles, skills and staffing required to support the updated operational plan for health information system, and align among the different departments as required.	**x**		Human Resources and Personnel Planning Directorate	Minister;<br><br>Secretary General;<br><br>Electronic Transformation and Information Technology Directorate	Mapping of existing positions;<br><br>Projection models	This will help ensure adequate and competent staffing at all levels.
**III- Improve coordination and planning of health information management at the national level**	**1-** Establish an interagency coordinating body or steering committee to oversee implementation and operationalization of the HIS strategy. This body should include representatives from ministry of health, high health council, national department of statistics, academia, local government, and the private healthcare sector.	**x**		MOH;<br><br>High Health Council	National Department of Statistics;<br><br>Academia;<br><br>Government;<br><br>Private Healthcare Sector	Buy-in and commitment of the different bodies	This committee can provide a technical advisory role for health and social welfare data managers in collaboration with other partners. In addition, it can oversee HIS coordination at the national level; and be responsible for approving and commissioning health data collection activities to avoid duplication of efforts and ensure added value.
	**2-** Conduct routine meetings between the different departments at MOH to manage HIS performance and review HIS data.	**x**		Secretary General;<br><br>MOH Department Heads	Electronic Transformation and Information Technology Directorate	Agreeing on a meeting date, venue and time.	This will result in clear task distribution for each department at MOH, avoid duplication of efforts and loss of resources; and eventually promote the use of HIS information at the national level.

Strategic Area II: Infrastructure and Resources

Initiative	Recommended Actions	Short-Medium term	Long term	Responsible entity	Supporting partner	Requirements/ Resources/ Pre-requisites	Expected Outcome (s)
**I- Ensure adequate human resources are available for the health information system**	**1-** Conduct a workforce assessment within MOH departments to map existing cadres to required job positions and to identify gaps in positions and capacities as they pertain to the functioning of the information systems	x		Human Resources and Personnel Planning Directorate	MOH Department Heads	Assessment tool;<br><br>Human resources	This will help in identifying the needed human resources for optimal operation of the health information system at all levels of the health system.
	**2-**Develop a medium-term staffing plan, identifying areas where new staff are most needed, especially projecting on the need for additional HMIS-related staff at the ministry, recruiting the needed human resources and outsourcing with specific tasks & deliverables.	x		Human Resources and Personnel Planning Directorate	Assistant Secretary General for Administrative and Financial Affairs	Adequate human resources are needed for developing the staffing plan;<br><br>Funds for recruiting additional needed staff at MOH	Defining short and long-term national health information and statistics human resources requirements based on updated operational models.
**II- Ensure adequate financial resources are dedicated for the health information system**	Increase budget allocations for the information systems (e.g., registers, forms, guidelines, maintenance, equipment, training, etc.)	X		Minister;<br><br>Secretary General	MOH Department Heads;<br><br>High Health Council	Securing budget line;<br><br>Proper forecasting of budget	This will enable smooth and sustainable operation of the information systems.
**III- Ensure availability of necessary hardware/ infrastructure**	**1-** Enhance computerization in public hospitals and primary healthcare centers by ensuring the availability of computers and basic Information and communication technology (ICT) infrastructure (telephones, internet access and e-mail). Ensure that the computer-based system is	x		Electronic Transformation and Information Technology Directorate;	Public Hospitals;<br><br>Primary Healthcare Centers	Adequate resources are needed (PCs, good internet connection, skilled human resources, follow up and training) at public hospitals and PHCCs in order to move	This will avoid duplication of data entry efforts at public hospitals and primary healthcare centers level (the parallel systems) and reduce time wasted on manual data entry.

Initiative	Recommended Actions	Short-Medium term	Long term	Responsible entity	Supporting partner	Requirements/ Resources/ Pre-requisites	Expected Outcome (s)
	comprehensive and contains all the needed fields of information. Require from all public hospitals and primary healthcare centers to rely on the computer-based system for data entry instead of using both the paper-based forms and the computer-based ones (possible incentive: link compliance to reimbursement).			Assistant Secretary General for Technical Health Affairs and Health Directorates in Governorates;<br><br>Assistant Secretary General for Primary Healthcare		fully to the computer-based system.	
	**2**- Create a dashboard system for hospitals to monitor healthcare key performance indicators (KPIs) in a dynamic and interactive way and enable healthcare professionals to access important patient statistics in real-time**.**	**x**		Electronic Transformation and Information Technology Directorate;<br><br>Assistant Secretary General for Technical Health Affairs and Health Directorates in Governorates	Private Hospitals Association	All the systems used at hospitals level should be interoperable and can feed into the ministry; Commitment from hospitals and collaboration with the private hospitals association are needed; Allocated financial resources.	The dashboard system will increase the overall hospital performance and patient satisfaction.

Initiative	Recommended Actions	Short-Medium term	Long term	Responsible entity	Supporting partner	Requirements/ Resources/ Pre-requisites	Expected Outcome (s)
	**3-** Rely on mobile data entry (using tablets) at the primary health care center level (instead of entering data on papers, then re-entering the same data into the PCs at the end of the working day).	**x**		Assistant Secretary General for Primary Healthcare	International Donors	Funds required for the provision of tablets for primary healthcare centers staff;<br>Training staff on using the tablets for data entry	This will ensure real time data entry by nurses and physicians, thus better data accuracy, improved data reporting and more compliance.<br>It will also overcome the barriers faced at the primary healthcare center level: lack of data entry staff, lack of time for using HIS at the primary healthcare centers level, and lack of PCs for data entry. It will also eliminate dual paper and computer entry of data.

Strategic Area III: Data Management

Initiative	Recommended Actions	Short-Medium term	Long term	Leading department/ entity	Supporting partner	Requirements/ Resources/ Pre-requisites	Expected outcome (s)
**I- Improve data generation at facility and national levels**	**1-**Develop, revise, and update the list of indicators (health systems, performance, quality, etc.) reported on and used by each department at MOH:<br>- Establish a National Health Indicator Compendium for national level programs and priorities, harmonized with international standards and reporting requirements.<br>- Ensure indicators cover different dimensions of the health system, including process and outcome indicators (e.g. avoidable hospital admissions which can help in evaluating the role of primary healthcare centers in prevention; patient-reported experience measures (instead of relying solely on patient satisfaction as an outcome indicator).<br>- Agree on a subset of indicators that require in-time data visualization and benchmarking	x		Institutional Development and Quality Directorate;<br><br>National Department of Statistics	MOH Department Heads	A baseline information on the indicators currently used by the different departments at MOH;<br>Priority setting exercise with key policymakers and stakeholders to prioritize the most needed indicators;<br>Assessment of readiness for undertaking measurement and reporting activities as well as potential training needs (that will be required);<br>Any changes may require new data collection forms and/or software updates as well as revising how indicators are collected;<br>National Health Data Dictionary to support the data requirements of the National Health Indicator Compendium and to serve as a standard for discrete data elements.	This approach will generate consensus on a core set of data and indicators that are meaningful, action-oriented and appropriate to the country situation. This, in turn, will enhance the relevance and responsiveness of the health information system to changing national needs and disease priorities as it will be able to focus on collecting information that is more directly linked to decision-making.

Initiative	Recommended Actions	Short-Medium term	Long term	Leading department/ entity	Supporting partner	Requirements/ Resources/ Pre-requisites	Expected outcome (s)
	**2-**Develop procedures manuals that include the following information:<br>- Standard forms for data collection and reporting<br>- Description of the following attributes for each indicator: case definitions, rationale for measurement, target population (denominator/numerator inclusion/exclusion criteria), source of data, measurement and estimation methods, expected frequency of data collection and reporting, and risk adjustments (where applicable).<br>- Individuals responsible for data collection and reporting<br>- Level of disaggregation of data<br>- Mechanisms for data processing and validation<br>- Log sheets for both manual and electronic data collection<br>- Data dissemination mode (may differ depending on the data/indicator and need for visualizations)	**x**		Institutional Development and Quality Directorate;<br><br>National Department of Statistics;<br><br>Electronic Transformation and Information Technology Directorate	MOH Department Heads	Standard forms for data collection and reporting;<br><br>Competency to properly establish key attributes of each indicator.	Procedure manuals will enable standardization of all aspects of data handling from collection, quality-assurance and flow, to processing, compilation and analysis. It will also provide guidance on key aspects including individuals responsible for data collection, type and methods of data collection, timing of data reporting, and data interpretation and use. This, in turn, will help ensure that data and indicators are comparable across settings and achieve compatible degrees of aggregation
	**3-** Link reporting on National Health Indicator Compendium to contractual agreements with MOH, especially for private sector		X	Secretary General	Health Facilities;<br><br>Third Party Payers;<br><br>Accreditation Personnel	Financial and human resources;<br><br>Training of health workers on reporting requirements;<br><br>Trained inspectors to ensure regular inspection and follow up;	This will help ensure compliance and standardization of reporting of national health indicators by health facilities.

Initiative	Recommended Actions	Short-Medium term	Long term	Leading department/ entity	Supporting partner	Requirements/ Resources/ Pre-requisites	Expected outcome (s)
						Systems to minimize gaming and data manipulation.	
	**4-** Standardize the regular production of health reports, disease surveillance reports, and health performance assessment reports (for example: monthly email-based Newsletter to be shared by each department within the ministry in addition to selected target audience)	x		All MOH Departments		This may require additional attention to the format and content of such reports, and support for design and production.	This will promote consistency, reproducibility, interchangeability and improve data generation and dissemination at MOH.
**II- Strengthen existing data sources**	**1-** Promote a well-functioning **civil registration** at the national level<br>- Establish an online system to directly send birth and death registration forms from health facilities to the Civil Registration Authority for verification.<br>- Use the most recent international Classification of Diseases (ICD) for coding causes of death.<br>- Unify and use the international form of the medical certificate of the cause of death across all facilities<br>- Certify physicians that have the knowledge and skills needed to accurately complete the international form of the medical certificate of the cause of death and are aware of the importance of correct cause-of-death certification<br>- Ensure that statistical clerks and health information officers have the training and reference materials needed to code deaths and disabilities according to the ICD	x		MOH;<br><br>National Department of Statistics;<br><br>Ministry of Interior	Private Hospitals Association;<br><br>Jordan Medical Association;<br><br>Higher Population Council;<br><br>Municipalities	Commitment from hospitals and health facilities;<br><br>Updated training and reporting manuals;<br><br>Human resources	Vital registration systems record the occurrence and characteristics of vital population events (e.g., births and deaths) and are a main source of population statistics. Countries with complete vital statistics registries (at least 90% coverage) may have more accurate and timely demographic indicators.

Initiative	Recommended Actions	Short-Medium term	Long term	Leading department/ entity	Supporting partner	Requirements/ Resources/ Pre-requisites	Expected outcome (s)
	- Ensure that systems for the automated coding of the causes of death are progressively used						
	**2-** Strengthen the Maternal Mortality Surveillance System and ensure proper coordination with the Maternal and Child Health Directorate to ensure a timely and effective response to reduce maternal and neonatal deaths.	**x**		MOH;<br><br>National Department of Statistics;<br><br>Maternal and Child Health Directorate	Private Hospitals Association;<br><br>Jordan Medical Association	Commitment from hospitals and health facilities;<br><br>Updated training and reporting manuals;<br><br>Human resources	A well-functioning Maternal Mortality Surveillance System will provide timely and quality information to identify causes of maternal and neonatal deaths, which can inform remedial actions to prevent future maternal and neonatal deaths.
**III –Optimize data flow within MOH**	**1-** Agree on a standard set of data and information to be shared regularly between the heads of departments as well as with the Minister.	**x**		All MOH Departments	Minister;<br><br>Secretary General	An inventory of existing information generated and shared by each department;<br><br>Commitment and buy-in of the Minister and the different departmental heads.	This will facilitate access to relevant information and avoid duplication of data collection and reporting.

Initiative	Recommended Actions	Short-Medium term	Long term	Leading department/ entity	Supporting partner	Requirements/ Resources/ Pre-requisites	Expected outcome (s)
	**2-** Automate all MOH departments and establish interoperability across all the systems used.		X	Electronic Transformation and Information Technology Directorate	MOH Department Heads	Technology that is fully developed and considered feasible;<br><br>Standardization in the ways data are collected and processed;<br><br>Mechanisms to ensure security and privacy of sensitive information;<br><br>Change management plan to overcome user resistance to automated systems.	Automation and unification will generate data and information that is far more accessible, more complete and accurate, can increase staff productivity, and may reduce operating costs.<br><br>This system could bring in transparency to the Department's processes as well as help overcome roadblocks in the Department's ability to function properly and manage its appreciably large information load.
	**3-** Provide systematic and constructive feedback to all sub-reporting departments/units on the quality of their reporting (that is, accuracy, completeness, and timeliness).	x		Minister;<br><br>Secretary General;<br><br>MOH Department Heads	Electronic Transformation and Information Technology Directorate		Fostering a closer link between management processes and information, so that constructive feedback from decision makers can be integrated into future practices.
	**4-** Integrate alerts into the existing systems used at MOH to notify decision makers about urgent numbers (outliers) that must have their attention.		x	Electronic Transformation and Information Technology Directorate	All MOH Departments	The alerting functionality must be capable of sending notifications in a variety of channels and priorities depending on defined conditions.	Fostering a closer link between management processes and information, so that the information can more effectively influence the decision-making process.

Initiative	Recommended Actions	Short-Medium term	Long term	Leading department/ entity	Supporting partner	Requirements/ Resources/ Pre-requisites	Expected outcome (s)
**IV –Optimize data flow between MOH and health facilities**	**1**- Reach out to physicians working in private clinics on data reporting requirement from MOH and train them on appropriate methods. Partner with the Jordan Medical Association to provide incentives (e.g. CME credits) for physicians working in private clinics to report data to the MOH.	x		Institutional Development and Quality Directorate	Jordan Medical Association	Training;<br><br>Transportation costs	This will fill the gap in information received from private clinics and it will lead to better-informed decisions.
	**2-** Reach out to health facilities located in remote areas and provide them with continuous support and follow up to encourage and enhance adherence to reporting requirements.		x	Institutional Development and Quality Directorate	Assistant Secretary General for Technical Health Affairs and Health Directorates in Governorates;<br><br>Assistant Secretary General for Primary Healthcare	Transportation costs	This will ensure that all health facilities are complying with data entry and reporting requirements.<br><br>It will also improve the communication with remote health facilities.
**V- Optimize data flow between MOH and external stakeholders**	**1-** Establish policies to enhance data sharing within and between governmental institutions including linking databases, and promoting inter-sectorial coordination.		x	Minister	All relevant ministries	IT maturity levels for data and information sharing among government institutions;<br><br>Clear policies, standards, and	These coordination mechanisms will enhance information exchange; help avoid widespread duplication of efforts and ensure resources are used in the most efficient manner.

Initiative	Recommended Actions	Short-Medium term	Long term	Leading department/ entity	Supporting partner	Requirements/ Resources/ Pre-requisites	Expected outcome (s)
						communication protocols;<br><br>Addressing incompatibility (interoperability issues) and data security;<br><br>Cooperation among sectors	Importantly, they will enable effective sharing of information for collaborative cross-sectoral planning & development.
	**2-** Create unified forms for external requests with clear guidelines and publish them on MOH website.	**x**		All MOH Departments	Electronic Transformation and Information Technology Directorate	Compiling all types of requests received at MOH, dividing them into categories and creating a form for every possible type of request.	This will help ensure that departments no longer receive vague external requests.
**VI – Improve data analysis at the departmental level at MOH**	**1-** Define general principles for data analysis process including how to deal with incompleteness, inconsistency, implausibility, estimation of denominators, imputation of missing values, and data reconciliation across data sources.	**x**		All MOH Departments		Technical support and consultancy services	This will standardize the data analysis process across staff and departments at MOH
	**2-** Develop Trainers' Guide explaining how to use and calculate indicators at the level of each department, and disseminate them to current and newly recruited staff.	**x**		All MOH Departments		The Trainers' Guide need to be available in soft copies or printed and supplied to all concerned staff at MOH in adequate quantities	The Trainers' Guide will help promote transparency, consistency, reproducibility, interchangeability and facilitate communication between the different departments at MOH.

Initiative	Recommended Actions	Short-Medium term	Long term	Leading department/ entity	Supporting partner	Requirements/ Resources/ Pre-requisites	Expected outcome (s)
	**3-** Establish collaborative partnership with research and academic institutions to conduct analytical reviews of facility data on a periodic basis.		x	Minister;<br><br>Secretary General;	Academic Institutions		Such partnership is a potential method for improving data analysis at the facility level, and producing more relevant research with greater potential to impact policy and practice at the national level
**VII- Implement improved data quality assurance and monitoring mechanisms at MOH**	**1-**Develop and implement a comprehensive consolidated plan for Quality Assurance at MOH across all the departments, starting with a written set of procedures for data collection, storage, cleaning, quality control, analysis and presentation for target audiences, and ensuring their proper implementation across all departments. Plan should incorporate a data quality system that ensures there is routine data quality checks and assessments across all the repositories of data within the health information system.<br>The procedures and their documentation are essential for each HIS data source.	x		Minister;<br><br>Secretary General;<br><br>Internal Audit and Control Directorate	All MOH Departments	Adopt validated data quality assurance tools and guidelines used internationally at the level of health ministries to the Jordanian context;<br><br>Technical support & consultancy services	Data quality assurance ensures that information is accurate and reliable; measuring what is intended to be measured and has been collected and measured in the same way (consistently) by all data collection units/programs during all reporting periods.<br><br>The will contribute to a stronger HIS that can produce high-quality and reliable data that can be used for more effective decision-making processes.
	**2-** Assign roles and responsibilities for data quality assurance at each level (health facility, district, provincial, and governorate) including verification of data, summarizing data quality issues, as well as developing and implementing data quality improvement strategies.	x		All MOH Departments	Internal Audit and Control Directorate	Managers and users must have the required expertise to interpret and use the available information in resource management	This will promote transparency, consistency, reproducibility, interchangeability and improve monitoring mechanisms for data

Initiative	Recommended Actions	Short-Medium term	Long term	Leading department/ entity	Supporting partner	Requirements/ Resources/ Pre-requisites	Expected outcome (s)
							quality assurance at MOH
	**3-** Develop standard operating procedures (SOPs) for routine data quality audits:<br>- Organize supervision visits to support implementation of SOPs, including the use of routine data quality audits tools<br>- Provide technical support for routine data quality audits cascade trainings for governorate managers to provide trainings for facility health workers	**X**		Internal Audit and Control Directorate	All MOH Departments	Human resources;<br><br>Transport costs	
	**4-** Improve the quality of health information from private sector health care providers:<br>- Define a set of minimum discrete data elements for routine reporting from private health sector.<br>- Implement mechanisms to support engagement and coordination with private sector health providers and other private sector stakeholders to ensure alignment and compliance with information reporting standards.	**X**		Institutional Development and Quality Directorate; Internal Audit and Control Directorate	Jordan Medical Association	Commitment from health professionals;<br><br>Training of health professionals on reporting requirements	This will promote transparency, consistency, reproducibility, interchangeability and improve monitoring mechanisms for data quality assurance at MOH.

Initiative	Recommended Actions	Short-Medium term	Long term	Leading department/ entity	Supporting partner	Requirements/ Resources/ Pre-requisites	Expected outcome (s)
**VIII- Implement improved data quality assurance and monitoring mechanisms at the level of health facilities**	**1-** Set routine health data quality assurance standards (data accuracy, completeness, and timeliness) and share them with all health facilities		x	All MOH Departments dealing with Health Facilities	Institutional Development and Quality Directorate;<br><br>Internal Audit and Control Directorate	Performance targets must be printed and supplied to all concerned health facilities in adequate quantities or sent to them by email	This will encourage health facilities to comply with MOH requirements and will increase their level of voluntary information reporting
	**2-** Conduct regular checks and periodical supervisory field visits to health facilities (using standard checklists derived from the SOPs) to audit data quality and to assess the accuracy and completeness of data received by the different MOH departments from health facilities prior to submitting reports to decision makers.	x		All MOH Departments dealing with Health Facilities	Institutional Development and Quality Directorate;<br><br>Internal Audit and Control Directorate	Inspectors are needed at the level of key departments to conduct regular checks;<br><br>Updating the existing checklists used during site visits with guidelines and a well-trained supervision team or using automated electronic checks, where applicable;<br><br>Transportation costs	This will result in improved data quality monitoring mechanisms at the national and health region levels.
	**3-** Collaborate with health facilities to develop internal processes for checks within the facility, such as:<br>– Written instructions/guidelines on how to perform a data quality reviews or data quality checks<br>– Policy requiring verification of collated summaries to be validated and signed off by senior staff and discussed before submission to higher levels.	x		All MOH Departments dealing with Health Facilities	Health facilities (hospitals, PHCCs, dispensaries, pharmacies, etc.)		This will ensure that a facility-based health information system is in place to provide a base for the good functioning of the overall health information system.

Initiative	Recommended Actions	Short-Medium term	Long term	Leading department/ entity	Supporting partner	Requirements/ Resources/ Pre-requisites	Expected outcome (s)
	**4-** Provide systematic and constructive feedback to health facilities/entities on the quality of their reporting (that is, accuracy, completeness, and timeliness) and provide recommendations for improvements. Establish a creative non-monetary mechanism to recognizegood quality data produced by health facilities.	**x**		All MOH Departments dealing with Health Facilities		Contact information of a focal person at the level of each health facility that can receive feedback and is committed to regular follow up with the ministry	This will motivate and enhance the performance of human resources working on producing data for the health information system and result in improved data quality at the national, and health region levels.

Strategic Area IV: Institutional capacity to expand the use of information to support evidence-infomed decision-making

Initiative	Recommended Actions	Short-Medium term	Long term	Responsible entity	Supporting partner	Requirements/ Resources/ Pre-requisites	Expected outcome (s)
**I – Build health information capacity of MOH staff**	**1-** Conduct a training needs assessment for MOH staff especially in health information management, data quality assurance, data compilation and reporting	**x**		Human Resources and Personnel Planning Directorate;<br><br>Electronic Transformation and Information Technology Directorate	MOH Department Heads	Established procedures for assessing the training and capacity needs of both new and long-term staff;<br><br>Human and financial resources to conduct the assessment	This will eventually strengthen the organizational capacity for health information management and data quality assurance within MOH

Initiative	Recommended Actions	Short-Medium term	Long term	Responsible entity	Supporting partner	Requirements/ Resources/ Pre-requisites	Expected outcome (s)
	**2-** Develop a costed training and capacity development plan that has benchmarks, timelines, and mechanisms for on-the-job RHIS training, RHIS workshops, and orientation for new staff.	**x**		Electronic Transformation and Information Technology Directorate	MOH Department Heads	A standardized routine health information system training curriculum that is readily available	
	**3-** Deliver annual routine health information system training using a standard training manual and ensure training materials are available as handbooks as well as online learning module. Ensure training covers the key features of the existing information systems at the level of each department.	**x**		Electronic Transformation and Information Technology Directorate	MOH Department Heads	Training plan;<br><br>Allocated financial and human resources	This will help ensure that the MOH staff have the needed skills and competencies decision-making and are acquainted with the latest features of the information system which will help optimize use of data and information to support evidence-informed decision-making.
	**4-** Recognize or reward staff for good work performance related to RHIS data at MOH. Provide regular feedback on reported data quality (e.g., accuracy of data compilation/reporting) to the staff responsible for compiling and reporting the data.	**x**		Minister;<br><br>Secretary General;<br><br>MOH Department Heads		Incentive system;<br><br>Systemic feedback loop	This will contribute to improved individual productivity and increased retention of quality employees at MOH
**II- Build health information capacity of healthcare professionals in Jordan**	**1-** Conduct training needs assessment for health professionals working in Jordan (nurses, physicians, information specialists including those working in private sector).	**x**		Human Resources Development and Education Directorate	Orders of health professionals in Jordan;<br><br>Private Hospitals Association	Training needs assessment tools;<br><br>Focal points outside MOH (for example at each governorate) to help with provision and coordination of trainings	Health professionals who understand the role of high-quality data and information in decision-making and who are motivated to report on the key data requirements needed from the Ministry;

Initiative	Recommended Actions	Short-Medium term	Long term	Responsible entity	Supporting partner	Requirements/ Resources/ Pre-requisites	Expected outcome (s)
	**2-** Collaborate with the Orders of Health professionals in Jordan (Nurses, physicians especially those working in private clinics, pharmacists, etc.) to provide regular trainings in health information (which can be integrated into continuing education or through special workshops) including data reporting requirements and methods for the ministry	**x**		Human Resources Development and Education Directorate	Orders of health professionals in Jordan;<br><br>Private Hospitals Association	Training materials and tools;<br><br>Focal points outside MOH (for example at each governorate) to help with provision and coordination of trainings	Better data received by ministry (completeness, quality and timeliness);<br><br>Filling the gap in information received from the private sector
**III- Conduct periodic assessment of Information needs**	Establish a platform that periodically brings together data producers and users to discuss ways of making routine data more relevant to policy makers and planners and to enhance understanding of routine health statistical findings.	**x**		Secretary General	MOH Department Heads	Procedures for the regular review of data needs and adaption of the HIS to meet health sector needs	This will enhance resilience and adaptability of the health information systems to changing needs and demands
**IV- Develop an Information Products Plan to meet specific needs of different information users at the MOH**	Develop and implement improved information products, aligned with the availability of information and human resources. Update MOH website with frequently requested information products.	**x**		Electronic Transformation and Information Technology Directorate	MOH Department Heads; Minister	Eliciting inputs of different users on information and format required to facilitate decision-making process;<br><br>Data visualization skills and data visualization platforms;<br><br>MOH Web governance and operational model to ensure currency and sustainability of MOH website.	This will enhance responsiveness of products to the needs of different information users at the MOH, thus, subsequently promoting their uptake and use in informing decision-making

Initiative	Recommended Actions	Short-Medium term	Long term	Responsible entity	Supporting partner	Requirements/ Resources/ Pre-requisites	Expected outcome (s)
**V- Establish mechanisms to increase access to health information and analysis tools for MOH staff**	**1-** Secure institutional access of MOH staff to online journals and research databases through subscriptions, networks, intranet sites, and physical libraries to support staff access and use of data and research in policy and action.<br>Provide written guidance on how to access, appraise and apply evidence; and re-enforce skills through periodic trainings	x		Secretary General;<br><br>Electronic Transformation and Information Technology Directorate		Selecting the relevant staff that need online access to research databases;<br><br>Funds to subscribe to online libraries and databases	This will support generation and utilization of operational research from the MOH departments. It will also allow MOH staff to stay up to date with the latest publications concerning health services and provision of health care
	**2-** Create an integrated common data repository (i.e. a data warehouse) for all data collected and generated by the different departments at the ministry; this repository should have a reporting utility that is accessible to various users at MOH. For instance, IERS (Integrated Electronic Reporting System) which currently acts a data repository to collect data from UNHCR, refugee camps and Hakeem can be upgraded and transformed into a data repository for the entire HIS (i.e. to include data from other sources).	x		Secretary General	Electronic Transformation and Information Technology Directorate	Document requirements and operational approach;<br><br>Repository must be able to grow and adapt to changes and new requirements and it should be accessible to key people at the ministry based on need (permission of access must be facilitated)	Facilitate data sharing between the different departments and overcome fragmentation of data sources.<br>The use of a unified, cross-programmatic identifier is a pre-requisite to improve continuity of care across health services within as well as between health facilities.
**VI- Strengthen data and Information dissemination mechanisms**	**1-** Disaggregate and disseminate data from population census and population-based surveys to the district level for monitoring service coverage and performance.	x		Assistant Secretary General for Technical Health Affairs and Health Directorates in Governorates;<br><br>Assistant Secretary General for		There is a need for functional central administrative unit responsible for population censuses and household surveys that designs, develops and supports health-information collection, management, analysis, dissemination and use for	This will enhance the performance of health facilities at the district level as it will allow them to keep track of their performance and monitor their service coverage

Initiative	Recommended Actions	Short-Medium term	Long term	Responsible entity	Supporting partner	Requirements/ Resources/ Pre-requisites	Expected outcome (s)
				Primary Healthcare		planning and management	
	**2-** Conduct training on appropriate use of social media for health information and implement the use of social media for disseminating health information.	X		Electronic Transformation and Information Technology Directorate			Reinforcing the use of social media at MOH for disseminating health information provides cost-effective ways to raise awareness concerning key health issues and promote the exchange of health-related information and experiences at the national level
	**3-** Hold regular monthly meetings with the different departments at MOH where data and information are discussed, performance reports are presented and reviewed, decisions are made, follow-up actions are identified, and their implementation is monitored.	X		Secretary General	MOH Department Heads	A platform that brings together the different policymakers and stakeholders	This will strengthen the rigor of policymaking process and enhance achievement of impact
**VII- Enhance accountability & transparency of decision-making processes**	**1-** Establish policies and guidelines mandating the use of data and evidence as an input in decision-making. As part of the mandate, policymakers could be required to submit summaries of whether and how data and evidence informed a given policy decision.		X	MOH;<br><br>Secretary General		Political will and regulatory support from policymakers;<br><br>A culture conducive to the use of evidence in decision-making processes	This will enhance the culture of transparency and accountability at the level of ministry and among external stakeholders as well as ensure the institution as a whole is receptive towards the use of date and research in policymaking process. This will eventually support the institutionalization of evidence-informed policymaking and action
	**2-** Create incentive systems to motivate the use of data and research in policymaking processes (e.g. recognition/award, promotions; inclusion of evidence use as part of performance appraisals)	x		MOH;<br><br>Secretary General		Awareness and recognition among staff of the important role of data and evidence in informing policy decisions and practice	

Initiative	Recommended Actions	Short-Medium term	Long term	Responsible entity	Supporting partner	Requirements/ Resources/ Pre-requisites	Expected outcome (s)
	**3-** Develop documented processes for how policies or national programs should be evaluated		x	MOH;<br><br>Secretary General		Political will and commitment<br><br>Monitoring and evaluation tools	at different levels of the health system
	**4-** Serve as a role model to raise the standards for policymaking in Jordan through advocating for the use of evidence to support decision making, and promoting a culture of information use that is reflected in:<br>- Health information (population health status, health system, risk factors) being demonstrably used in the planning and in the resource-allocation processes (e.g. for annual integrated development plans, medium-term expenditure frameworks, long-term strategic plans, and annual health sector reviews)<br>- Use of facility and community-based data in planning, monitoring, and evaluation reports<br>- Published case studies highlighting the important role of evidence in informing policies and programs		x	MOH;<br><br>Secretary General		Policy champions who will advocate for the important role of data and research in informing different steps of policymaking processes, programs, and practices and who will lead by example	
