# Supplementary material for: Promoting data-driven decision-making in Jordan: strengthening national health information system and achieving consensus on core set of health system indicators
Source: Reprod Health. 2025 May 31;22(Suppl 1):72. doi: 10.1186/s12978-025-01988-1 (PMC12125760; doi:10.1186/s12978-025-01988-1)
Supplement: Supplementary file 3 — Additional file 3. Overview and evolution of indicators from Jordan’s national health information system [file 12978_2025_1988_MOESM3_ESM.pdf]

Additional file 3: Overview and evolution of indicators from Jordan's national health information system

Section 1: Initial list of indicators compiled from the different national sources within Jordan (n=215)

1. Adolescent birth rate (per 1,000 women)
2. Annual Exam Retina – Referred
3. Annual Exam Retina – Tested
4. Antenatal care coverage - at least four visits (%)
5. Antenatal care coverage - at least one visit (%)
6. Women with 7+ ANC visits (%)
7. Antiretroviral therapy coverage among people with advanced HIV infection (%)
8. Average number of growth and development follow- up visits by children under one
9. Births attended by skilled health personnel (%)
10. Births delivered by C-section (%)
11. Births delivered in a health facility (%)
12. Blood pressure control in DM
13. Cancer cases (breast, colon) detected at early phases (national record)
14. Breast cancer screening
15. Pap Test
16. Cancer survival rate (after 5 years of treatment)
17. Children age 12-23 months who received all age-appropriate vaccinations (%)
18. Children age 12-23 months who received all basic vaccinations (%)
19. Children aged <5 years with ARI symptoms receiving antibiotics (%)
20. Children aged <5 years with diarrhea receiving oral rehydration therapy (%)
21. Prevalence and treatment of diarrhea and feeding practices during diarrhea
22. Children aged 6-59 months who received vitamin A supplementation (%)
23. Children with suspected pneumonia taken to an appropriate health provider (%)
24. Contraceptive Prevalence
25. Crude birth rate (per 1000 population)
26. Crude death rate (per 1000 population)
27. Density of computed tomography units (per million population)
28. Density of dentistry personnel (per 1,000 population)
29. Density of environment and public health workers (per 1,000 population)
30. Density of nursing and midwifery personnel (per 1,000 population)
31. Density of pharmaceutical personnel (per 1,000 population)
32. Density of physicians (per 1,000 population)
33. Dentist rate per 10,000 population
34. Number of dentistry personnel
35. Number of environment and public health workers
36. Number of healthcare institutions with accreditation (accumulated)
37. Number of nursing and midwifery personnel
38. Number of pharmaceutical personnel

39. Number of physicians
40. Midwife per 10,000 population
41. Psychiatrists working in mental health sector (per 100,000 population)
42. Pharmacist rate per 10,000 population
43. Physician rate per 10,000 population
44. Registered nurse per 10,000 population
45. Number of physicians specialized in elderly medicine
46. Problems in Accessing Health Care
47. Diabetes prevalence among citizens of 18+ years
48. Diphtheria tetanus toxoid and pertussis (DTP3) immunization coverage among 1-year-olds (%)
49. Distribution of causes of death among children aged <5 years (%)
50. Distribution of years of life lost by major cause group (%)
51. Estimated deaths due to tuberculosis, excluding HIV (per 100,000 population)
52. Estimated incidence of tuberculosis (per 100,000 population)
53. Estimated pregnant women living with HIV who received antiretroviral medicine for preventing mother-to-child transmission (%)
54. Ever-married women age 15-49 who smoke any type of tobacco (%)
55. Current smoking of any tobacco product (age-standardized rate)
56. Men age 15-49 who smoke any type of tobacco (%)
57. Smoking prevalence among citizens of 18+ years
58. Estimated prevalence of tuberculosis (per 100,000 population)
59. Ever-married women age 15-19 who are mothers or currently pregnant (%)
60. Ever-married women who have experienced emotional, physical, or sexual violence committed by their spouse (%)
61. Ever-married women who have experienced physical violence by their spouse (%)
62. Ever-married women who have experienced sexual violence by their spouse (%)
63. Ever-married women who know that using condoms and limiting sexual intercourse to one uninfected partner reduces the risk of contracting HIV (%)
64. Men who know that using condoms and limiting sexual intercourse to one uninfected partner reduces the risk of contracting HIV (%)
65. External resources for health as a percentage of total expenditure on health
66. Gender inequality index
67. General government expenditure on health as a percentage of total government expenditure
68. General government expenditure on health as a percentage of total expenditure on health
69. Ever-married women age 15-49 with any health insurance (%)
70. Health insurance coverage rate among Jordanians
71. Health insurance coverage rate among population
72. Men age 15-49 with any health insurance (%)
73. Hepatitis B (HepB3) immunization coverage among 1-year-olds (%)
74. Hib (Hib3) immunization coverage among 1-year-olds (%)
75. HIV prevalence among adults aged 15-49 years (%)
76. AIDS 10,000 of the population
77. HIV incidence rate
78. Hospital beds (per 10,000 population)

79. Human development index
80. Prevalence and treatment of fever
81. Prevalence and treatment of childhood illnesses
82. Percentage of Emaciated Children Under One Year of Age
83. Infant mortality rate (per 1000 live birth)
84. Jordan rank in elderly health on Global Watch index
85. Leprosy - Number of new leprosy cases
86. Life expectancy at birth (years)
87. Malaria incidence rate among Jordanians
88. Maternal mortality ratio (per 100,000 live births)
89. Measles-containing-vaccine first-dose (MCV1) immunization coverage among 1-year-olds (%)
90. Median duration of any breastfeeding (months)
91. Median duration of exclusive breastfeeding (months)
92. Infants exclusively breastfed for the first 6 months of life (%)
93. Percentage of Infants Exclusively Breastfed for 6 Entire Months
94. Men who report having an STI symptom in past 12 months (among those who have heard of STIs) (%)
95. Low-birth-weight (%)
96. Percentage of Underweight Children Under One Year of Age
97. Mental hospitals (per 100,000 population)
98. Perinatal Mortality
99. Neonatal mortality rate (per 1000 live births)
100. Neonates protected at birth against neonatal tetanus (%)
101. Notified cases of tuberculosis
102. Number of primary and comprehensive centers qualified to treat physiological cases
103. Number of qualified centers to provide palliative care and psychological care for cancer patients and families
104. Median age at first birth for women age 25-49 (years)
105. Median age at first marriage for women age 25-49 (years)
106. Out of pocket expenditure
107. Out-of-pocket expenditure as a percentage of private expenditure on health
108. Per capita government expenditure on health (PPP int. $)
109. Per capita government expenditure on health at average exchange rate (US$)
110. Per Capita Healthcare Expenditure (JD)
111. Per capita total expenditure on health (PPP int. $)
112. Per capita total expenditure on health at average exchange rate (US$)
113. Percentage Distribution of Antenatal Clients Registered Per Each Trimester
114. Percentage of all live births to adolescents
115. Percentage of Anemia Cases Based on Antenatal Hemoglobin Testing
116. Percentage of Anemic Antenatal Clients
117. (%) Women age 15-49 with any anemia (by type: mild, moderate, severe)
118. Percentage of Mildly Anemic Antenatal Clients
119. Percentage of Moderately Anemic Antenatal Clients
120. Percentage of Severely Anemic Antenatal Clients

121. The Percentage of Anemic Postpartum Clients Based on Hemoglobin Testing
122. Percentage of Antenatal Clients Receiving a General Examination
123. Percentage of Antenatal Clients Receiving Counseling on Family Planning during their Third Trimester
124. No. of people reach through MOH Health Centers Antenatal Health Promotion Activities
125. Number of Breastfeeding Health Promotion Activities Conducted at MOH Health Centers
126. Number of Child Care Health Promotion Activities Conducted at MOH Health Centers
127. Number of Couple Years of Protection (CYP) at all Facilities within SDP
128. Number of Couple Years of Protection (CYP) at MOH Facilities
129. Number of FP Health Promotion Activities Conducted at MOH Health Centers
130. Number of MOH SDPs Providing Implants Services
131. Number of People Reached through MOH Health Centers' Child Health Care promotion Activities
132. Number of SDPs within JCLS Providing Implants Services
133. Number of Women Referred from MCH Centers to Another SDP to Receive Modern FP Method
134. Number of Women/men Reached through MOH Health Centers’ FP Health Promotion Activities
135. Percentage of IUDs Inserted by Midwives at MCH Centers
136. Percentage of IUDs Inserted by Physicians at MCH Centers
137. Number of People Reached through MOH Health Centers' Breastfeeding Health Promotion Activities
138. Percentage of MCH Center Clients Presenting with Postpartum Complications
139. Percentage of MOH SDPs Providing IUD Services
140. Percentage of MOH SDPs Stocked out from FP method
141. Percentage of MOH Service Delivery Points Providing 3 Modern Contraceptives
142. Percentage of MOH Service Delivery Points Providing 4 Modern Contraceptives including IUD or Contraceptive Implants
143. Percentage of SDPs within JCLS Providing IUD Services
144. Percentage of SDPs within JCLS Stocked out from FP Method
145. Percentage of Service Delivery Points within JCLS Providing 3Modern Contraceptives
146. Percentage of Service Delivery Points within JCLS Providing 4 Modern Contraceptives including IUD or Contraceptive Implants
147. Percentage of Yearly increase in Couple Years of Protection (CYP) at MOH Facilities
148. Percentage of Yearly increase in Couple Years of Protection (CYP) at all Facilities included within JCLS
149. Percentile Contribution of all Sectors Within JCLS in Couple Years of Protection CYP
150. No. of Antenatal Care Health Promotion Activities Conducted at MOH Health Centers
151. Percentage of Antenatal Clients Receiving Health Education
152. Percentage of Child Visits to Health Centers for Treatment Purposes
153. Percentage of Children Under One Receiving a General Examination
154. Percentage of Children Under One Year of Age Receiving Hemoglobin Testing
155. Percentage of Clients Receiving Counseling on Breastfeeding During Postpartum Visits
156. Percentage of Clients Receiving Counseling on Family Planning during Postpartum Visits
157. Percentage of Clients Receiving Health Education during Postpartum Visits
158. Percentage of Controlled Diabetics
159. Percentage of Controlled Diabetics, using HbA1C

160. Percentage of Exclusively Breastfed Infants 4 to 6 Months of Age
161. Percentage of Exclusively Breastfed Infants under 4 Months of Age
162. Percentage of growth and development follow-up visits conducted by children under 1 year of age
163. Percentage of Anemic Children Under One Year of Age Based on Hemoglobin Testing
164. Percentage of Hemoglobin Test Result Values Less Than 11gm/dL Performed for Children more than 1 Year to 5 Years of Age
165. Percentage of Mildly Anemic Children Under One Year of Age
166. Percentage of Moderately Anemic Children Under One Year of Age
167. Percentage of Severely Anemic Children Under One Year of Age
168. Children age 6-59 months with any anemia (%)
169. Percentage of High-Risk Pregnancy Referrals
170.
171. Percentage of Newly Registered Antenatal Clients Undergoing Hemoglobin Testing
172. Percentage of Obese Children Under One Year of Age
173. Percentage of Overweight Children Under One Year of Age
174. Children aged <5 years overweight (%)
175. Percentage of High Birth Weight Newborn Infants
176. Newborns with birth weight >2.5 kg
177. Percentage of Postpartum Care Service Utilization by Clients Previously Registered at the Center for Antenatal Care
178. Percentage of Postpartum Clients Receiving Modern FP Method from Health Centers
179. Percentage of High-Risk Pregnancies
180. Percentage of Low-Risk Pregnancies
181. Percentage of Moderate-Risk Pregnancies
182. Percentage of Postpartum Clinic Clients Receiving Hemoglobin Testing
183. Premarital Medical Exam
184. Children aged <5 years stunted (%)
185. Percentage of Stunting in Children Under One Year of Age
186. Stunting rate in children under 5Percentage of Low Birth Weight Newborn Infants
187. Percentage of Third-Trimester Antenatal Clients Receiving Counseling on Breastfeeding
188. Pharmaceutical expenditure as percent of total health expenditure
189. Population aged 15-24 years with comprehensive correct knowledge of HIV/AIDS (%)
190. Poverty headcount ratio at 1.25$ a day (PPP) (% of population)
191. Practitioners of modest physical activities
192. Children aged <5 years wasted (%)
193. Wasting rate in children under 5 years
194. Primary healthcare expenditure as percent of public health expenditure
195. Private expenditure on health as a percentage of total expenditure on health
196. Public health expenditure as percent of GDP
197. Public health expenditure as percent of total health expenditure
198. Rate of using postnatal services
199. The Average Number of Postpartum Client Visits – annually
200. Social security expenditure on health as a percentage of general government expenditure on health
201. TB incidence rate per 100,000

202. The Percentage of Postpartum Service Utilization by Previous Antenatal Clients with an Identified Expected Delivery Date
203. Total expenditure on health as a percentage of gross domestic product
204. Total fertility rate
205. Tuberculosis treatment coverage for all forms of tuberculosis
206. Treatment success rate for new pulmonary smear-positive tuberculosis cases
207. Under 5 mortality rate (per 1000 live birth)
208. Unmet need for family planning (%)
209. Family Panning Demand satisfied by modern methods (%)
210. Current use of a modern method of family planning (%)
211. Use of modern family planning
212. Percentage of Controlled Hypertensive Patients
213. Updated Hypertension Register
214. Women age 15-49 who are overweight or obese (%)
215. GDP growth (annual %)

Section 2: Overview of the revised list of indicators being reported within Jordan's national HIS after the first round of assessment (n=104)

1. Adolescent birth rate (per 1,000 women)
2. Total fertility rate
3. Life expectancy at birth (years)
4. Annual Exam Retina – Referred and tested
5. Antenatal care coverage - at least one visit (%)
6. Antenatal care coverage - at least four visits (%)
7. Antenatal care (eight or more visits)
8. Antiretroviral therapy coverage among people with advanced HIV infection (%)
9. Births attended by skilled health personnel (%)
10. Births delivered by C-section (%)
11. Births delivered in a health facility (%)
12. Blood pressure control in DM
13. Cancer cases (breast, colon) detected at early phases (national record)
14. Breast cancer screening
15. Pap Test
16. Cancer survival rate (after 5 years of treatment)
17. Percentage of Children Under One Receiving a General Examination
18. Proportion of children who are fully vaccinated at 1, 2 and 6 years of age
19. Coverage of DPT containing vaccine (3rd dose)
20. Average number of growth and development follow- up visits by children under one
21. Prevalence of and obesity by age; men and women [gender sensitive]
22. Premarital Medical Exam
23. Children aged <5 years stunted (%)
24. Children aged <5 years wasted (%)
25. Children aged <5 years with ARI symptoms receiving antibiotics (%)
26. Children aged <5 years with diarrhea receiving oral rehydration therapy (%)
27. Children aged 6-59 months who received vitamin A supplementation (%)
28. Children with suspected pneumonia taken to an appropriate health provider (%)
29. Crude birth rate (per 1000 population)
30. Crude death rate (per 1000 population)
31. Tobacco use among persons aged 15+ years, men and women by age
32. Contraceptive Prevalence
33. Current use of a modern method of family planning (%)
34. Family Panning Demand satisfied by modern methods (%)
35. Problems in Accessing Health Care
36. Density of computed tomography units (per million population)
37. Number of health workers per 10,000 population by type of health worker (physicians, nurses, midwives, pharmacists, laboratory technicians, dentists, community and traditional health worker)
38. Diabetes prevalence among citizens of 18+ years
39. Distribution of causes of death among children aged <5 years (%)
40. Distribution of years of life lost by major cause group (%)
41. Estimated deaths due to tuberculosis, excluding HIV (per 100,000 population)

42. Incidence and prevalence of tuberculosis (per 100,000 population)
43. Tuberculosis treatment coverage for all forms of tuberculosis and success rate for new pulmonary smear-positive tuberculosis cases
44. Estimated pregnant women living with HIV who received antiretroviral medicine for preventing mother-to-child transmission (%)
45. Ever-married women age 15-19 who are mothers or currently pregnant (%)
46. Prevalence of intimate partner violence, by type (physical, sexual, psychological)
47. Percentage of men and women who know that using condoms and limiting sexual intercourse to one uninfected partner reduces the risk of contracting HIV
48. Gender inequality index
49. Percentage of Emaciated Children Under One Year of Age
50. Prevalence and treatment of childhood illnesses
51. External resources for health as a percentage of total expenditure on health
52. General government expenditure on health as a percentage of total government expenditure
53. General government expenditure on health as a percentage of total expenditure on health
54. Percentage of population covered by insurance; men and women by age and by gender- specific services Health promotion program effectiveness
55. Hepatitis B (HepB3) immunization coverage among 1-year-olds (%)
56. Hib (Hib3) immunization coverage among 1-year-olds (%)
57. HIV prevalence among adults aged 15-49 years (%)
58. HIV incidence rate
59. Hospital beds (per 10,000 population)
60. Human development index
61. Infant mortality rate (per 1000 live births)
62. Jordan rank in elderly health on Global Watch index
63. Malaria incidence rate among Jordanians
64. Leprosy - Number of new leprosy cases
65. Child’s size and weight at birth
66. Percentage of Low Birth Weight Newborn Infants
67. Newborns with birth weight >2.5 kg
68. Measles-containing-vaccine first-dose (MCV1) immunization coverage among 1-year-olds (%)
69. Median age at first marriage and at first birth for women age 25-49 (years)
70. Men who report having an STI symptom in past 12 months (among those who have heard of STIs) (%)
71. Mental hospitals (per 100,000 population)
72. Maternal mortality ratio (per 100,000 live births)
73. Under 5 mortality rate (per 1000 live births)
74. Perinatal Mortality rate
75. Neonatal mortality rate (per 1000 live births)
76. Neonates protected at birth against neonatal tetanus (%)
77. Rate of using postnatal services
78. Number of healthcare institutions with accreditation (accumulated)
79. Number of physicians specialized in elderly medicine
80. Number of primary and comprehensive centers qualified to treat physiological cases

81. Number of qualified centers to provide palliative care and psychological care for cancer patients and families
82. Out-of-pocket expenditure
83. Out-of-pocket expenditure as a percentage of private expenditure on health
84. Per capita government expenditure on health (PPP int. $)
85. Per Capita Healthcare Expenditure (JD)
86. Per capita total expenditure on health (PPP int. $)
87. Percentage of Controlled Diabetics
88. Percentage of Controlled Hypertensive Patients
89. Percentage of High-Risk Pregnancy Referrals
90. Infants exclusively breastfed for the first 6 months of life (%)
91. (%) Women age 15-49 with any anemia (by type: mild, moderate, severe)
92. Percentage of Anemic Children by age (by type: mild, moderate, severe)
93. Percentage of Children Under One Year of Age Receiving Hemoglobin Testing
94. Percentage of Postpartum Clients Receiving Modern FP Method from Health Centers
95. Pharmaceutical expenditure as percent of total health expenditure
96. Population aged 15-24 years with comprehensive correct knowledge of HIV/AIDS (%)
97. Poverty headcount ratio at 1.25$ a day (PPP) (% of population)
98. Prevalence of insufficiently physically active persons by age and sex activities
99. Primary healthcare expenditure as percent of public health expenditure
100. Private expenditure on health as a percentage of total expenditure on health
101. Social security expenditure on health as a percentage of general government expenditure on health
102. The Percentage of Postpartum Service Utilization by Previous Antenatal Clients with an Identified Expected Delivery Date
103. Total expenditure on health as a percentage of gross domestic product
104. GDP growth (annual %)

Section 3: Overview of the revised list of indicators from Jordan's National HIS included in the priority setting tools (after the second round of assessment) and those retained after the prioritization and validation exercises

Jordan's National HIS indicators	Included in the priority setting tools (Yes/No)	Retained in the final set of short-listed and validated indicators (Yes/No)
1. Adolescent birth rate	**Yes**	**No**
2. Total fertility rate	**Yes**	**No**
3. Life expectancy at birth (years)	**Yes**	**No**
4. Annual Exam Retina – Referred and tested	**Yes**	**No**
5. Antenatal care coverage - at least one visit (%)	**Yes**	**Yes**
6. Antenatal care coverage - at least four visits (%)	**Yes**	**Yes**
7. Antenatal care (eight or more visits)	**Yes**	**No**
8. Antiretroviral therapy coverage among people with advanced HIV infection (%)	**Yes**	**No**
9. Births attended by skilled health personnel (%)	**Yes**	**Yes**
10. Births delivered by C-section (%)	**Yes**	**No**
11. Births delivered in a health facility (%)	**Yes**	**No**
12. Blood pressure control in DM	**No**	**N/A**
13. Cancer cases (breast, colon) detected at early phases (national record)	**Yes**	**Yes**
14. Breast cancer screening	**Yes**	**No**
15. Pap Test	**Yes**	**No**
16. Cancer survival rate (after 5 years of treatment)	**Yes**	**Yes**
17. Percentage of Children Under One Receiving a General Examination	**No**	**N/A**
18. Proportion of children who are fully vaccinated at 1, 2 and 6 years of age	**Yes**	**Yes**
19. Coverage of DPT containing vaccine (3rd dose)	**Yes**	**Yes**
20. Average number of growth and development follow-up visits by children under one	**No**	**N/A**
21. Prevalence of overweight and obesity by age; men and women	**Yes**	**Yes**
22. Premarital Medical Exam	**No**	**N/A**
23. Children aged <5 years stunted (%)	**Yes**	**Yes**
24. Children aged <5 years wasted (%)	**No**	**N/A**
25. Children aged <5 years with ARI symptoms receiving antibiotics (%)	**Yes**	**No**
26. Children aged <5 years with diarrhea receiving oral rehydration therapy (%)	**Yes**	**No**
27. Children aged 6-59 months who received vitamin A supplementation (%)	**Yes**	**Yes**

Jordan's National HIS indicators	Included in the priority setting tools (Yes/No)	Retained in the final set of short-listed and validated indicators (Yes/No)
28. Children with suspected pneumonia taken to an appropriate health provider (%)	**Yes**	**No**
29. Crude birth rate (per 1000 population)	**Yes**	**Yes**
30. Crude death rate (per 1000 population)	**Yes**	**Yes**
31. Tobacco use among persons aged 15+ years, men and women by age	**Yes**	**No**
32. Contraceptive Prevalence	**Yes**	**Yes**
33. Current use of a modern method of family planning (%)	**Yes**	**No**
34. Family Panning Demand satisfied by modern methods (%)	**Yes**	**Yes**
35. Problems in Accessing Health Care	**Yes**	**No**
36. Density of computed tomography units (per million population)	**No**	**N/A**
37. Number of health workers per 10,000 population by type of health worker (physicians, nurses, midwives, pharmacists, laboratory technicians, dentists, community and traditional health workers)	**Yes**	**Yes**
38. Diabetes prevalence among citizens of 18+ years	**Yes**	**Yes**
39. Distribution of causes of death among children aged <5 years (%)	**Yes**	**Yes**
40. Distribution of years of life lost by major cause group (%)	**No**	**N/A**
41. Estimated deaths due to tuberculosis, excluding HIV (per 100,000 population)	**Yes**	**No**
42. Incidence and prevalence of tuberculosis (per 100,000 population)	**No**	**N/A**
43. Tuberculosis treatment coverage for all forms of tuberculosis and success rate for new pulmonary smear-positive tuberculosis cases	**No**	**N/A**
44. Estimated pregnant women living with HIV who received antiretroviral medicine for preventing mother-to-child transmission (%)	**Yes**	**No**
45. Ever-married women age 15-19 who are mothers or currently pregnant (%)	**No**	**N/A**
46. Prevalence of intimate partner violence, by type (physical, sexual, psychological)	**Yes**	**No**
47. Percentage of men and women who know that using condoms and limiting sexual intercourse to one uninfected partner reduces the risk of contracting HIV	**Yes**	**No**

Jordan’s National HIS indicators	Included in the priority setting tools (Yes/No)	Retained in the final set of short-listed and validated indicators (Yes/No)
48. Gender inequality index	**Yes**	**No**
49. Percentage of Emaciated Children Under One Year of Age	**No**	**N/A**
50. Prevalence and treatment of childhood illnesses	**No**	**N/A**
51. External resources for health as a percentage of total expenditure on health	**Yes**	**No**
52. General government expenditure on health as a percentage of total government expenditure	**Yes**	**No**
53. General government expenditure on health as a percentage of total expenditure on health	**Yes**	**No**
54. Percentage of population covered by insurance; men and women by age and by gender- specific services	**No**	**N/A**
55. Hepatitis B (HepB3) immunization coverage among 1-year-olds (%)	**No**	**N/A**
56. Hib (Hib3) immunization coverage among 1-year-olds (%)	**No**	**N/A**
57. HIV prevalence among adults aged 15-49 years (%)	**Yes**	**No**
58. HIV incidence rate	**Yes**	**Yes**
59. Hospital beds (per 10,000 population)	**Yes**	**Yes**
60. Human development index	**Yes**	**Yes**
61. Infant mortality rate (per 1000 live births)	**Yes**	**Yes**
62. Jordan rank in elderly health on Global Watch index	**Yes**	**Yes**
63. Malaria incidence rate among Jordanians	**No**	**N/A**
64. Leprosy - Number of new leprosy cases	**Yes**	**No**
65. Child’s size and weight at birth	**Yes**	**No**
66. Percentage of Low Birth Weight Newborn Infants	**Yes**	**Yes**
67. Children aged <5 years overweight (%)	**Yes**	**No**
68. Measles-containing-vaccine first-dose (MCV1) immunization coverage among 1-year-olds (%)	**Yes**	**Yes**
69. Median age at first marriage and at first birth for women age 25-49 (years)	**No**	**N/A**
70. Men who report having an STI symptom in past 12 months (among those who have heard of STIs) (%)	**Yes**	**No**
71. Mental hospitals (per 100,000 population)	**Yes**	**No**
72. Maternal mortality ratio (per 100,000 live births)	**Yes**	**Yes**

Jordan’s National HIS indicators	Included in the priority setting tools (Yes/No)	Retained in the final set of short-listed and validated indicators (Yes/No)
73. Under 5 mortality rate (per 1000 live births)	**Yes**	**Yes**
74. Neonatal mortality rate (per 1000 live births)	**Yes**	**Yes**
75. Perinatal Mortality rate	**Yes**	**No**
76. Neonates protected at birth against neonatal tetanus (%)	**Yes**	**No**
77. Rate of using postnatal services	**Yes**	**No**
78. Number of healthcare institutions with accreditation (accumulated)	**Yes**	**Yes**
79. Number of physicians specialized in elderly medicine	**Yes**	**No**
80. Number of primary and comprehensive centers qualified to treat physiological cases	**Yes**	**No**
81. Number of qualified centers to provide palliative care and psychological care for cancer patients and families	**Yes**	**No**
82. Out-of-pocket expenditure per capita	**Yes**	**No**
83. Out-of-pocket expenditure as a percentage of private expenditure on health	**Yes**	**No**
84. Per capita government expenditure on health (PPP int. $)	**Yes**	**No**
85. Per Capita Healthcare Expenditure (JD)	**Yes**	**No**
86. Per capita total expenditure on health (PPP int. $)	**Yes**	**No**
87. Percentage of Controlled Diabetics	**Yes**	**No**
88. Percentage of Controlled Hypertensive Patients	**Yes**	**No**
89. Percentage of High-Risk Pregnancy Referrals	**No**	**N/A**
90. Infants exclusively breastfed for the first 6 months of life (%)	**Yes**	**No**
91. (%) Women age 15-49 with any anemia (by type: mild, moderate, severe)	**Yes**	**Yes**
92. Percentage of Anemic Children by age (by type: mild, moderate, severe)	**Yes**	**Yes**
93. Percentage of Children Under One Year of Age Receiving Hemoglobin Testing	**No**	**N/A**
94. Percentage of Postpartum Clients Receiving Modern FP Method from Health Centers	**Yes**	**Yes**
95. Pharmaceutical expenditure as percent of total health expenditure	**Yes**	**Yes**
96. Population aged 15-24 years with comprehensive correct knowledge of HIV/AIDS (%)	**Yes**	**No**

Jordan's National HIS indicators	Included in the priority setting tools (Yes/No)	Retained in the final set of short-listed and validated indicators (Yes/No)
97. Poverty headcount ratio at 1.25$ a day (PPP) (% of population)	**Yes**	**No**
98. Prevalence of insufficiently physically active persons by age and sex	**Yes**	**No**
99. Primary healthcare expenditure as a percent of public health expenditure	**Yes**	**Yes**
100. Private expenditure on health as a percentage of total expenditure on health	**Yes**	**No**
101. Social security expenditure on health as a percentage of general government expenditure on health	**No**	**N/A**
102. The Percentage of Postpartum Service Utilization by Previous Antenatal Clients with an Identified Expected Delivery Date	**Yes**	**No**
103. Total expenditure on health as a percentage of gross domestic product	**Yes**	**Yes**
104. GDP growth (annual %)	**Yes**	**Yes**
**Total**	**84/104 (80.7%)**	**35/84 (41.6%)**
