# Supplementary material for: Promoting data-driven decision-making in Jordan: strengthening national health information system and achieving consensus on core set of health system indicators
Source: Reprod Health. 2025 May 31;22(Suppl 1):72. doi: 10.1186/s12978-025-01988-1 (PMC12125760; doi:10.1186/s12978-025-01988-1)
Supplement: Supplementary file 4 — Additional file 4. Procedure manual for maternal, child and adolescent health. [file 12978_2025_1988_MOESM4_ESM.pdf]

Procedures Manual

Maternal, Child and Adolescent Health Indicators

PROCEDURES MANUAL 1

MATERNAL, CHILD AND ADOLESCENT HEALTH INDICATORS 1

MATERNAL INDICATORS 4

DIMENSION 1 4

MATERNAL MORTALITY RATIO (PER 100,000 LIVE BIRTHS) 4

UNDER FIVE MORTALITY RATE; BOYS AND GIRLS 9

INFANT MORTALITY RATE 13

NEONATAL MORTALITY RATE (PER 1000 LIVE BIRTHS) 18

DISTRIBUTION OF CAUSES OF DEATH AMONG CHILDREN AGED <5 YEARS (%) 23

LOW-BIRTH-WEIGHT NEWBORNS (%) 28

PREVALENCE OF STUNTING AMONG CHILDREN AGED <5 YEARS (%) 32

PREVALENCE OF ANAEMIA IN CHILDREN AGED 6–59 MONTHS (%) 33

DIMENSION 2 37

PREVALENCE OF ANAEMIA IN WOMEN AGED 15-49, BY PREGNANCY STATUS (%) 72

DIMENSION 3 75

AVAILABILITY OF EMERGENCY OBSTETRIC SERVICES (EMOC) ERROR! BOOKMARK NOT DEFINED.

% OF HEALTH CENTERS PROVIDING FAMILY PLANNING METHOD ERROR! BOOKMARK NOT DEFINED.

PERCENT OF WOMEN WHO DELIVERED IN A FACILITY AND RECEIVED COUNSELLING ON FAMILY PLANNING PRIOR TO DISCHARGE .... ERROR! BOOKMARK NOT DEFINED.

% OF POSTPARTUM CLIENTS RECEIVING MODERN FAMILY PLANNING METHOD FROM HEALTH CENTERS .... ERROR! BOOKMARK NOT DEFINED.

REFERRAL RATES FOR WOMEN WITH OBSTETRIC COMPLICATIONS 92

MET NEED FOR EMERGENCY OBSTETRIC AND NEWBORN CARE 101

DEMAND FOR FAMILY PLANNING SATISFIED - USE OF MODERN METHODS (%) 109

ANTENATAL CARE COVERAGE (AT LEAST ONE VISIT) 115

ANTENATAL CARE COVERAGE (AT LEAST FOUR VISITS) 119

PERCENT OF PREGNANT WOMEN WHO RECEIVE THE RECOMMENDED NUMBER OF IRON/FOLATE SUPPLEMENTS DURING PREGNANCY 122

% OF BIRTHS DELIVERED IN A HEALTH FACILITY AMONG ALL BIRTHS IN THE POPULATION 127

NEONATAL TETANUS PROTECTION ....ERROR! BOOKMARK NOT DEFINED.
% OF BABIES WEIGHED AT BIRTH.. 129
% OF NEWBORNS RECEIVING ESSENTIAL NEWBORN CARE 133
PROPORTION OF PREGNANT WOMEN WITH HYPERTENSION RECEIVING ANTIHYPERTENSIVE DRUGS 138
% OF MOTHERS WHO RECEIVED COUNSELLING, SUPPORT OR MESSAGES ON OPTIMAL BREASTFEEDING AT LEAST ONCE IN THE LAST YEAR... 143
POSTPARTUM CARE COVERAGE FOR MOTHERS (%)... 148
VITAMIN A SUPPLEMENTATION COVERAGE (% OF CHILDREN AGED 6-59 MONTHS WHO RECEIVED TWO AGE APPROPRIATE DOSES OF VITAMIN A IN THE PAST 12 MONTHS)* 150
DIMENSION 4 155
FACILITY MORTALITY RATE DISAGGREGATED BY BIRTH WEIGHT >4000G, 2500-3999G, 2000-2499G, 1500-1999G, <1500G ....ERROR! BOOKMARK NOT DEFINED.
NEWBORN RESUSCITATION (%) ....ERROR! BOOKMARK NOT DEFINED.
PROPORTION OF HEALTH FACILITIES OFFERING MATERNITY SERVICES CERTIFIED BY THE BABY-FRIENDLY HOSPITAL INITIATIVE ....ERROR! BOOKMARK NOT DEFINED.
PROPORTION OF HEALTH FACILITIES WITH SAFE, UNINTERRUPTED OXYGEN SUPPLY IN CHILDBIRTH, NEONATAL AND PEDIATRIC WARDS 161
NEONATAL DEATH REVIEW COVERAGE (%) 165
% AND DISTRIBUTION OF HEALTH WORKERS TRAINED TO PROVIDE REPRODUCTIVE MATERNAL AND CHILD HEALTH – OPTION 1 166
% AND DISTRIBUTION OF HEALTH WORKERS TRAINED TO PROVIDE REPRODUCTIVE MATERNAL AND CHILD HEALTH – OPTION 2 167
DENSITY OF MIDWIVES BY DISTRICT (BY BIRTHS).. 171
ADOLESCENT INDICATORS 176
DIMENSION 1 177
ADOLESCENT MORTALITY RATE (PER 100 000 POPULATION) 177
ADOLESCENT MORTALITY RATE FROM ROAD TRAFFIC INJURIES....................................... 181
ADOLESCENT MORTALITY RATE FROM SUICIDE 186
PREVALENCE OF HIV INFECTION AMONG ADOLESCENTS.. 187
ADOLESCENT FERTILITY RATE (PER 1000 WOMEN AGED 15-19 YEARS)............................ 197
DIMENSION 2 236
YOUTH LITERACY RATE FOR 15-24 YEARS ....ERROR! BOOKMARK NOT DEFINED.

PERCENTAGE (%) OF WOMEN AGED 20-24 YEARS WHO WERE MARRIED BEFORE AGE 18ERROR! BOOKMARK NOT DEFINED.

AGE-STANDARDIZED SUICIDE RATES (PER 100 000 POPULATION)ERROR! BOOKMARK NOT DEFINED.

Maternal Indicators

Dimension 1

SDG 3.1.1 Maternal mortality ratio (per 100,000 live births)

Formula

$$\frac{\text{Number of maternal deaths}}{\text{Number of live births}} \times 100{,}000$$

- Continuous information collection for this indicator.
- Dissemination every 3-5 years
- The specific codes used under ICD-10 (the 10th revision of the ICD) to define a maternal death are: O00-O96; O98, O99 and A34

Methods of Measure

The maternal mortality ratio can be calculated by dividing recorded (or estimated) maternal deaths by total recorded (or estimated) live births in the same period and multiplying by 100,000. Measurement requires information on pregnancy status, timing of death (during pregnancy, childbirth, or within 42 days of termination of pregnancy), and cause of death. Maternal mortality ratio = (Number of maternal deaths / Number of live births) X 100,000. The maternal mortality ratio can be calculated directly from data collected through vital registration systems, household surveys or other sources.

1. Collect Data on Denominator
Denominator: Live births defined as "The complete expulsion or extraction from its mother of a product of conception, irrespective of the duration of the pregnancy, which, after such separation, breathes or shows any other evidence of life such as beating of the heart, pulsation of the umbilical cord, or definite movement of voluntary muscles, whether or not the umbilical cord has been cut or the placenta is attached".

1.1 Include

- All live births.

1.2 Exclude

- NA

2. Collect Data on Numerator
Numerator: Female deaths from a cause related to or aggravated by pregnancy or management during pregnancy and childbirth or termination of pregnancy, within 42 days, irrespective of the duration and site of the pregnancy.

2.1 Include

- All female deaths caused by or aggravated by pregnancy

- All female deaths caused or aggravated by management during pregnancy or childbirth
- All female death caused or aggravated by pregnancy, irrespective of the duration and site of the pregnancy within 42 days.

2.2 Exclude

- Female deaths from accidental or incidental causes related to or aggravated by pregnancy or its management

3. This indicator will be stratified based on: Age

- Education level
- Location (urban/rural)
- Wealth: Wealth quintile
- Boundaries: Administrative regions/ Health regions

4. Data Sources

4.1 Preferred data Sources:

- Civil registration with complete coverage and medical certification of cause of death

4.2 Other data Sources:

- Household surveys
- Population census
- Sample or sentinel registration systems
- Special studies

5. Calculate:

Divide recorded (or estimated) maternal deaths by total recorded (or estimated) live births in the same period and multiply by 100,000

6. Possible Limitations:

- Underreporting and misclassification of maternal deaths
- Weak CRVS and HIS systems causing inaccurate assessment of maternal mortality.
- Misclassification of deaths in complete CRVS systems
- Under reporting of deaths in complete CRVS systems

Data Collection Sheet

The following table contains detailed information and support from the literature on the measurement of the indicator.

Indicator	Maternal mortality ratio
Definition	The maternal mortality ratio (MMR) is defined as the number of maternal deaths during a given time period per 100,000 live births during the same time period. It depicts the risk of maternal death relative to the number of live births and essentially captures the risk of death in a single pregnancy or a single live birth.<br><br>**Maternal deaths:** The annual number of female deaths from any cause related to or aggravated by pregnancy or its management (excluding accidental or incidental causes) during pregnancy and childbirth or within 42 days of termination of pregnancy, irrespective of the duration and site of the pregnancy, expressed per 100,000 live births, for a specified time period.<br><br>**Live birth:** The complete expulsion or extraction from its mother of a product of conception, irrespective of the duration of the pregnancy, which, after such separation, breathes or shows any other evidence of life such as beating of the heart, pulsation of the umbilical cord, or definite movement of voluntary muscles, whether or not the umbilical cord has been cut or the placenta is attached. (ICD-10)<br><br>For the purpose of international reporting of maternal mortality, only those maternal deaths occurring before the end of the 42-day reference period should be included in the calculation of the various ratios and rates. The recording of later deaths is encouraged to inform national, regional, and global understanding of these events [1]
Rationale	The World Health Organization (WHO) and partners have released a consensus statement and full strategy paper on ending preventable maternal mortality (EPMM) back in 2015 .The EPMM target for reducing the global maternal mortality ratio (MMR) by 2030 was adopted as SDG target 3.1: reduce global MMR to less than 70 per 100 000 live births by 2030. WHO leads the UN Maternal Mortality Estimation Interagency Group (MMEIG) composed of WHO, UNICEF, UNFPA, the United Nations Population Division, and the World Bank Group. The MMEIG is tasked with generating internationally comparable estimates of maternal mortality for the purposes of global monitoring. [1]<br>Complications during pregnancy and childbirth are a leading cause of death and disability among women of reproductive age in developing countries. The maternal mortality ratio represents the risk associated with each pregnancy, i.e. the obstetric risk. It is also a Millennium Development Goal Indicator for monitoring Goal 5, improving maternal health. The indicator monitors deaths related to pregnancy and childbirth. It reflects the capacity of the health systems to provide effective health care in preventing and addressing the complications occurring during pregnancy and childbirth.
Type of indicator	Impact
Unit of measure	Deaths per 100 000 live births

Formula	Number of maternal deaths X 100,000<br>Number of live births
Target	Aggregates presented only if available data cover 50% of total live births
Frequency	▪ Continuous information collection for this indicator.<br>▪ Dissemination every 3-5 years
Possible Limitations	▪ Underreporting and misclassification of maternal deaths<br>▪ Weak CRVS and HIS systems causing inaccurate assessment of maternal mortality<br>▪ Misclassification of deaths in complete CRVS systems<br>▪ Under reporting of deaths in complete CRVS systems
Denominator inclusion criteria	All live births [1]
Denominator exclusion criteria	NA
Numerator inclusion criteria	▪ All female deaths caused by or aggravated by pregnancy<br>▪ All female deaths caused or aggravated by management during pregnancy or childbirth<br>▪ All female death caused or aggravated by pregnancy, irrespective of the duration and site of the pregnancy within 42 days.1
Numerator exclusion criteria	▪ Female deaths from accidental or incidental causes related to or aggravated by pregnancy or its management 1
Data sources	▪ Civil registration with complete coverage and medical certification of cause of death<br>▪ Household surveys<br>▪ Population census<br>▪ Sample or sentinel registration systems<br>▪ Special studies

References

1. Maternal mortality ratio (per 100 000 live births). (2020). Retrieved 5 December 2020, from https://www.who.int/data/gho/data/indicators/indicator-details/GHO/maternal-mortality-ratio-(per-100-000-live-births)

   https://www.who.int/data/gho/indicator-metadata-registry/imr-details/3140SDG Indicators — SDG Indicators (un.org)

For the above indicator, can you provide the below information:

- Can the data elements (numerator and denominator) be collected in the context of Jordan?

[X] Yes Comments:

[ ] No Comments:

- Are the data sources listed above applicable (i.e. information can be extracted from the suggested data source) in the context of Jordan? If no, please provide the source.

[ ] Yes Comments: Hospital based reports

[ ] No Comments:

- Can the data for the above indicator be segregated for nationals and refugees?

[X] Yes Comments: could be segregated based on social security number for Jordanians and not Jordanians

[ ] No Comments:

- Is the data for Jordan reported in the same way as indicated above? *If you answer "No", please indicate in the comments section how it is being reported. If the indicator is not being reported in Jordan, please insert "Not reported" in the comments section*

[X] Yes Comments: It is reported as mortality rate in general; could be segregated for different groups.

[ ] No Comments:

- Is the data for this indicator available for the period extending from January 2019 to December 2021? (if yes, please provide the data)

[ ] Yes Comments:

[ ] No Comments:

SDG3.2.1 Under five mortality rate; boys and girls

Formula

$$\frac{\text{Number of deaths of children under five in a calendar year or specific period (broken down by age group)}}{\text{Number of live births in the same year}} \times 1000$$

- Collect information on this indicator on annual basis.
- Dissemination of information on annual basis.

Methods of Measure

1. Collect Data on Denominator
Denominator: number of live births in a specific period or year

1.1 Include
- All live births for a specific period or year

1.2 Exclude
- NA

2. Collect Data on Numerator
Numerator: number of children who were under 5 years during the specific period or year and who died during that period.

2.1 Include
- Number of children who were under 5 years during the specific period or year and who died during that period

2.2 Exclude
- NA

3. This indicator will be stratified based on:
- Age
- Sex
- Economic status (wealth quintile and wealth decile)
- Education (mother's education)
- Place of residence
- Subnational region

4. Data Sources
4.1 Preferred data Sources:
- Civil registration system with complete coverage

4.2 Other data Sources:

- Demographic and Health Surveys (DHS)
- Multiple Indicator Cluster Surveys (MICS)
- Reproductive Health Surveys (RHS)
- micro-data which are publicly available using the standard indicator definitions as published in DHS, MICS or RHS documentation
- Household surveys
- Population census

5. Calculate:

The indicator is calculated as equal to the number of deaths of children under five in a calendar year divided by the number of live births in the same year and multiplied by 1,000.

6. Possible Limitations:

- Some countries lack a single source of high-quality data
- Data from different sources require different calculation methods and may suffer from different errors, causing different surveys to yield widely different estimates
- Available data collected by countries are often inconsistent across sources
- Sampling or non-sampling errors (such as misreporting of age, age heaping and survivor selection bias; underreporting of child deaths)

Data Collection Sheet

The following table contains detailed information and support from the literature on the measurement of the indicator.

Indicator	Under-five mortality rate (deaths per 1000 live births)
Alternate name	Under-five mortality rate (deaths per 1000 live births)<br>Under-five mortality rate (probability of dying by age 5 per 1000 live births)
Dimension	D1
Definition	The probability (expressed as a rate per 1000 live births) of a child born in a specific year or period dying before reaching the age of five, if subject to age-specific mortality rates of that period. Under-five mortality rate as defined here is strictly speaking not a rate (i.e. the number of deaths divided by the number of population at risk during a certain period of time) but a probability of death derived from a life table and expressed as rate per 1000 live births.
Rationale	Under-five mortality rate measures child survival. It also reflects the social, economic and environmental conditions in which children (and others in society) live, including their health care. Because data on the incidences and prevalence of diseases (morbidity data) frequently are unavailable, mortality rates are often used to identify vulnerable populations. Under-five mortality rate is an SDG indicator.
Type of indicator	Impact
Unit of measure	Deaths per 1000 live births
Formula	(Nb of deaths of children under five (broken down by age group) in a calendar year or specific period/<br>Number of live births in the same year) x 1000
Target	Complete vital statistics registration system—one covering at least 90 per cent of vital events in the population
Frequency	▪ Collect information on this indicator on annual basis.<br>▪ Dissemination of information on annual basis.<br>▪
Possible Limitations	▪ Some countries lack a single source of high-quality data<br>▪ Data from different sources require different calculation methods and may suffer from different errors, causing different surveys to yield widely different estimates<br>▪ Available data collected by countries are often inconsistent across sources<br>▪ Sampling or non-sampling errors (such as misreporting of age, age heaping and survivor selection bias; underreporting of child deaths)<br>▪
Denominator inclusion criteria	▪ Number of live births in a specific period or year
Denominator exclusion criteria	▪ NA
Numerator inclusion criteria	▪ Number of children who were under 5 years during the specific period or year and who died during that period
Numerator exclusion criteria	▪ NA

Data sources	▪ Civil registration system with complete coverage<br>▪ Demographic and Health Surveys (DHS)<br>▪ Multiple Indicator Cluster Surveys (MICS)<br>▪ Reproductive Health Surveys (RHS)<br>▪ Micro-data which are publicly available using the standard indicator definitions as published in DHS, MICS or RHS documentation<br>▪ Household surveys<br>▪ Population census

References

- Under-five mortality rate (probability of dying by age 5 per 1000 live births). (2020). Retrieved 5 December 2020, from https://www.who.int/data/gho/data/indicators/indicator-details/GHO/under-five-mortality-rate-(probability-of-dying-by-age-5-per-1000-live-births)
- Under-five mortality rate (deaths per 1000 live births). (2020). Retrieved 5 December 2020, from https://www.who.int/data/gho/data/indicators/indicator-details/GHO/under-five-mortality-rate-(deaths-per-1000-live-births)
- (We no more talk MDG now we talk SDG)
- SDG Indicators — SDG Indicators (un.org)
- chi_2015_26_mortality_under5.pdf (who.int)

For the above indicator, can you provide the below information:

- Can the data elements (numerator and denominator) be collected in the context of Jordan?

[X] Yes Comments:

[ ] No Comments:

- Are the data sources listed above applicable (i.e. information can be extracted from the suggested data source) in the context of Jordan? If no, please provide the source.

[ ] Yes Comments: Hospital based reports

[ ] No Comments:

- Can the data for the above indicator be segregated for nationals and refugees?

[X] Yes Comments: could be segregated based on social security number for Jordanians and not Jordanians

[ ] No Comments:

- Is the data for Jordan reported in the same way as indicated above? *If you answer "No", please indicate in the comments section how it is being reported. If the indicator is not being reported in Jordan, please insert "Not reported" in the comments section*

[X] Yes Comments: It is reported as mortality rate in general; could be segregated for different groups.

[ ] No Comments:

- Is the data for this indicator available for the period extending from January 2019 to December 2021? (if yes, please provide the data)

[ ] Yes Comments:

[ ] No Comments:

Infant mortality rate

Formula

$$\frac{\text{Number of deaths of infants under one year of age in a calendar year}}{\text{Number of live births in the same year}} \times 1{,}000$$

Data collection & dissemination:

- Annual data collection
- Annual data dissemination

Method of measurement:

- **Civil registration:** Number of deaths at age 0 and population for the same age are used to calculate death rate which are then converted into age-specific probability of dying.
- **Census and surveys**: An indirect method is used based on questions to each woman of reproductive age as to how many children she has ever born and how many are still alive. The Brass method and model life tables are then used to obtain an estimate of infant mortality.
- **Surveys:** A direct method is used based on birth history - a series of detailed questions on each child a woman has given birth to during her lifetime. To reduce sampling errors, the estimates are generally presented as period rates, for five or 10 years preceding the survey.

1. Collect Data on Denominator
Denominator: number of live births occurring during a specific year

1.1 Include
Number of live births occurring during a specific year

1.2 Exclude
- NA

2. Collect Data on Numerator
Numerator: number of infants aged 0–11 months during a specific year and who died during that year

2.1 Include
Number of infants aged 0–11 months during a specific year and who died during that year

Exclude
- NA

3. This indicator will be stratified based on:

- Age : 0-27 days, Age : 28 days - <1 year
- Economic status (wealth quintile and wealth decile)
- Place of residence
- Sex
- Subnational region
- neonatal vs. postneonatal periods

4. Data Sources

4.1 Preferred data Sources:

- Civil registration with complete coverage

4.2 Other data Sources:

- Census and surveys

5. Calculate:

The indicator is calculated dividing the number of deaths of infants under one year of age in a calendar year by the number of live births in the same year and multiplied by 1,000.

6. Possible Limitations:

- Underestimation of civil registration systems
- Substantial variation in data quality and consistency across countries.
- Recent child mortality trend information is difficult to meet through household surveys.
- Necessity of high quality of civil registration systems (completeness of registration) and high quality of survey or census data collection
- Sampling or non-sampling errors (such as misreporting of age, age heaping and survivor selection bias; underreporting of child deaths is also common)

Data Collection Sheet

The following table contains detailed information and support from the literature on the measurement of the indicator.

Indicator	Infant mortality rate
Alternate name	Infant mortality rate (deaths per 1000 live births)<br><br>Infant mortality rate (probability of dying between birth and age 1 per 1000 live births)
Definition	Probability (expressed as a rate per 1000 live births) of a child born in a specific year or period dying before reaching the age of five years, if subject to age-specific mortality rates of that period.[4]
Rationale	Infant mortality represents an important component of under-five mortality. Like under-five mortality, infant mortality rates measure child survival. They also reflect the social, economic and environmental conditions in which children (and others in society) live, including their health care. Since data on the incidence and prevalence of diseases (morbidity data) frequently are unavailable, mortality rates are often used to identify vulnerable populations.
Type of indicator	Impact
Unit of measure	Deaths per 1000 live births
Method of measurement	▪ **Civil registration:** Number of deaths at age 0 and population for the same age are used to calculate death rate which are then converted into age-specific probability of dying.<br>▪ **Census and surveys**: An indirect method is used based on questions to each woman of reproductive age as to how many children she has ever born and how many are still alive. The Brass method and model life tables are then used to obtain an estimate of infant mortality.<br>▪ **Surveys:** A direct method is used based on birth history - a series of detailed questions on each child a woman has given birth to during her lifetime. To reduce sampling errors, the estimates are generally presented as period rates, for five or 10 years preceding the survey.
Formula	(Number of deaths of infants under one year of age in a calendar year/Number of live births in the same year) x 1,000
Target	To be determined
Frequency	▪ Annual data collection.<br>▪ Annual data dissemination.
Possible Limitations	▪ Underestimation of civil registration systems<br>▪ Substantial variation in data quality and consistency across countries.<br>▪ Recent child mortality trend information is difficult to meet through household surveys.<br>▪ Necessity of high quality of civil registration systems (completeness of registration) and high quality of survey or census data collection<br>▪ Sampling or non-sampling errors (such as misreporting of age, age heaping and survivor selection bias; underreporting of child deaths is also common)
Denominator inclusion criteria	Number of live births occurring during a specific year

Denominator exclusion criteria	NA
Numerator inclusion criteria	Number of infants aged 0–11 months during a specific year and who died during that year
Numerator exclusion criteria	▪ NA
Data sources	▪ Civil registration with complete coverage<br>▪ Census and Surveys<br>▪ Surveys

References:

Infant mortality rate (deaths per 1000 live births). (2020). Retrieved 5 December 2020, from https://www.who.int/data/gho/data/indicators/indicator-details/GHO/infant-mortality-rate-(deaths-per-1000-live-births)

https://www.who.int/data/gho/data/indicators/indicator-details/GHO/infant-mortality-rate-(probability-of-dying-between-birth-and-age-1-per-1000-live-b

http://mdgs.un.org/unsd/mi/wiki/4-2-Infant-mortality-rate.ashx

chi_2015_27_mortality_infant.pdf (who.int)

For the above indicator, can you provide the below information:

- Can the data elements (numerator and denominator) be collected in the context of Jordan?

[X] Yes Comments:

[ ] No Comments:

- Are the data sources listed above applicable (i.e. information can be extracted from the suggested data source) in the context of Jordan? If no, please provide the source.

[ ] Yes Comments: Hospital based reports

[ ] No Comments:

- Can the data for the above indicator be segregated for nationals and refugees?

[X] Yes Comments: could be segregated based on social security number for Jordanians and not Jordanians

[ ] No Comments:

- Is the data for Jordan reported in the same way as indicated above? *If you answer "No", please indicate in the comments section how it is being reported. If the indicator is not being reported in Jordan, please insert "Not reported" in the comments section*

[X] Yes Comments: It is reported as mortality rate in general; could be segregated for different groups.

[ ] No Comments:

- Is the data for this indicator available for the period extending from January 2019 to December 2021? (if yes, please provide the data)

[ ] Yes Comments:

[ ] No Comments:

SDG 3.2.2 Neonatal mortality rate (per 1000 live births)

Formula

$$\frac{\text{Number of neonatal deaths at less than 28 days of age during a specific time}}{\text{Number of live births}} \times 1{,}000$$

Data collection & dissemination:

- Annual data collection.
- Annual data dissemination.

Method of measurement:

- **Data from civil registration:** Number of children who died during the first 28 days of life and the number of births used to calculate neonatal mortality rates. This system provides annual data.
- Censuses and surveys: Censuses and surveys often include questions on household deaths in the last 12 months, which can be used to calculate mortality estimates.
- **Data from household surveys:** Calculations are based on full birth history, whereby women are asked for the date of birth of each of their children, whether the child is still alive, and if not the age at death. Neonatal, post-neonatal, infant, child and under-five mortality estimates can be derived from the full birth history module.

1. Collect Data on Denominator
Denominator: number of live births

1.1 Include
- Number of live births

1.2 Exclude
- NA

2. Collect Data on Numerator
Numerator: Number of neonatal deaths at less than 28 days of age during a specific time

2.1 Include
- Number of neonatal deaths at less than 28 days of age during a specific time

2.2 Exclude
- NA

3. This indicator will be stratified based on:

- Sex
- Age (subdivided into early neonatal deaths, occurring during the first 7 days of life, and late neonatal deaths, occurring after the 7th day but before the 28th completed day of life)
- Socio economic status
- Residence
- Mother's education
- Geographic location (at the regional or provincial level)

4. Data Sources

4.1 Preferred data Sources:

- Civil Registration with complete coverage

4.2 Other data Sources:

- Population census and household survey

5. Calculate:

Divide the number of neonatal deaths at less than 28 days of age during a specific time by the number of live births and multiply by 1,000

6. Possible Limitations:

- Some countries lack a single source of high-quality data
- Data from different sources require different calculation methods and may suffer from different errors, causing different surveys to yield widely different estimates
- Available data collected by countries are often inconsistent across sources
- Sampling or non-sampling errors (such as misreporting of age, age heaping and survivor selection bias; underreporting of child deaths)

Data Collection Sheet

The following table contains detailed information and support from the literature on the measurement of the indicator.

Indicator	Neonatal mortality rate (per 1000 live births)
Definition	The probability that a child born in a specific year or period will die during the first 28 completed days of life if subject to age-specific mortality rates of that period, expressed per 1000 live births.. Neonatal deaths (deaths among live births during the first 28 completed days of life) may be subdivided into early neonatal deaths, occurring during the first 7 days of life, and late neonatal deaths, occurring after the 7th day but before the 28th completed day of life.
Rationale	Mortality rates among young children are a key output indicator for child health and well-being, and, more broadly, for social and economic development. It is a closely watched public health indicator because it reflects the access of children and communities to basic health interventions such as vaccination, medical treatment of infectious diseases and adequate nutrition.
Type of indicator	Impact
Unit of measure	Deaths per 1000 live births
Method of measurement	**Data from civil registration:** Number of children who died during the first 28 days of life and the number of births used to calculate neonatal mortality rates.. This system provides annual data.<br>**Censuses and surveys:** Censuses and surveys often include questions on household deaths in the last 12 months, which can be used to calculate mortality estimates.<br>**Data from household surveys:** Calculations are based on full birth history, whereby women are asked for the date of birth of each of their children, whether the child is still alive, and if not the age at death. Neonatal, post-neonatal, infant, child and under-five mortality estimates can be derived from the full birth history module.
Formula	(Number of neonatal deaths at less than 28 days of age during a specific time/Number of live births) x 1,000
Target	To be determined
Frequency	▪ Annual data collection.<br>▪ Annual data dissemination.
Possible Limitations	▪ Some countries lack a single source of high-quality data<br>▪ Data from different sources require different calculation methods and may suffer from different errors, causing different surveys to yield widely different estimates<br>▪ Available data collected by countries are often inconsistent across sources<br>▪ Sampling or non-sampling errors (such as misreporting of age, age heaping and survivor selection bias; underreporting of child deaths)
Denominator inclusion criteria	▪ Number of live births
Denominator exclusion criteria	▪ NA
Numerator inclusion criteria	▪ Number of neonatal deaths at less than 28 days of age during a specific time

Numerator exclusion criteria	▪ NA
Data sources	▪ Civil registration with complete coverage<br>▪ Population census and household survey
Indicator responsibility	▪ Ministry of Health at the central level and lower administrative units<br>▪ National Statistical Office or the Ministry of Health are mostly involved in generating neonatal mortality data at the national level.<br>▪ UN IGME

References:

Neonatal mortality rate (per 1000 live births). (2020). Retrieved 5 December 2020, from https://www.who.int/data/gho/data/indicators/indicator-details/GHO/neonatal-mortality-rate-(per-1000-live-births)

UNICEF, Indicator 3.2.2: Neonatal mortality rate, https://unstats.un.org/sdgs/metadata/files/Metadata-03-02-02.pdf

UNICEF, Indicator Profiles, Neonatal mortality Rate, https://data.unicef.org/indicator-profile/CME_MRM0/

For the above indicator, can you provide the below information:

- Can the data elements (numerator and denominator) be collected in the context of Jordan?

[X] Yes Comments:

[ ] No Comments:

- Are the data sources listed above applicable (i.e. information can be extracted from the suggested data source) in the context of Jordan? If no, please provide the source.

[ ] Yes Comments: Hospital based reports

[ ] No Comments:

- Can the data for the above indicator be segregated for nationals and refugees?

[X] Yes Comments: could be segregated based on social security number for Jordanians and not Jordanians

[ ] No Comments:

- Is the data for Jordan reported in the same way as indicated above? *If you answer "No", please indicate in the comments section how it is being reported. If the indicator is not being reported in Jordan, please insert "Not reported" in the comments section*

[X] Yes Comments: It is reported as mortality rate in general; could be segregated for different groups.

[ ] No Comments:

- Is the data for this indicator available for the period extending from January 2019 to December 2021? (if yes, please provide the data)

[ ] Yes Comments:

[ ] No Comments:

Distribution of causes of death among children aged <5 years (%)

Formula

$$\frac{\text{Number of children aged} < 5 \text{ years with one specific cause of death}}{\text{Number of all deaths in children aged} < 5 \text{ years}} \times 1{,}000$$

Data collection & dissemination:

- Annual data collection.
- Annual data dissemination.

Method of measurement:

- **Data from civil registration:** Data from civil registration with complete coverage (80% or over) and medical certification of cause of death.
- **Epidemiological studies (representative):** Nationally representative epidemiological studies of causes of child death (special studies analysing causes of death based on verbal autopsy studies or other sources for countries without civil registration data).

1. Collect Data on Denominator
Denominator: number of all death in children aged < 5 years

1.1 Include
- Number of all deaths in children aged < 5 years

1.2 Exclude
- NA

2. Collect Data on Numerator
Numerator: Number of children aged < 5 years with one specific cause of death

2.1 Include
- Number of children aged < 5 years with one specific cause of death

2.2 Exclude
- NA

3. This indicator will be stratified based on:
- Age

4. Data Sources

4.1 Preferred data Sources:

- Civil registration with complete coverage and medical certification of cause of death

4.2 Other data Sources:

- Special studies

5. Calculate:

Divide the number of children aged < 5 years with one specific cause of death by the number of all deaths in children aged < 5 years and multiply by 100

6. Possible Limitations:

- A better understanding of the indirect contributions of diseases to child deaths is needed in order to assess disease control priorities and evaluate interventions.

Data Collection Sheet

The following table contains detailed information and support from the literature on the measurement of the indicator.

Indicator	Distribution of causes of death among children aged <5 years (%)
Definition	Distribution of main causes of death among children aged < 5 years, expressed as percentage of total deaths. The causes of death refers to the concept of the 'underlying cause of death' as defined by ICD-10 (WHO, 1992).[7]
Rationale	Efforts to improve child survival can be effective only if they are based on reasonably accurate information about the causes of childhood deaths. Cause-of-death information is needed to prioritize interventions and plan for their delivery, to determine the effectiveness of disease-specific interventions, and to assess trends in disease burden in relation to national and international goals.[7]
Type of indicator	Impact
Unit of measure	Percentage
Method of measurement	**Data from civil registration** with complete coverage (80% or over) and medical certification of cause of death.<br>**OR**<br>**Nationally representative epidemiological studies** of causes of child death (special studies analyzing causes of death based on verbal autopsy studies or other sources for countries without civil registration data).[7]
Formula	(Number of children aged < 5 years with one specific cause of death/<br>Number of all deaths in children aged < 5 years) x 100
Target	Complete coverage (80% or over)
Frequency	▪ Annual data collection.<br>▪ Annual data dissemination.
Possible Limitations	▪ A better understanding of the indirect contributions of diseases to child deaths is needed in order to assess disease control priorities and evaluate interventions.7
Denominator inclusion criteria	▪ number of all deaths in children aged < 5 years
Denominator exclusion criteria	▪ NA
Numerator inclusion criteria	▪ Number of children aged < 5 years with one specific cause of death
Numerator exclusion criteria	▪ NA
Data sources	▪ Civil registration with complete coverage and medical certification of cause of death<br>▪ Special Studies

References:

Distribution of causes of death among children aged < 5 years (%). (2020). Retrieved 5 December 2020, from https://www.who.int/data/gho/data/indicators/indicator-details/GHO/distribution-of-causes-of-death-among-children-aged-5-years-(-)

For the above indicator, can you provide the below information:

- Can the data elements (numerator and denominator) be collected in the context of Jordan?

[X] Yes Comments:

[ ] No Comments:

- Are the data sources listed above applicable (i.e. information can be extracted from the suggested data source) in the context of Jordan? If no, please provide the source.

[ ] Yes Comments: Hospital based reports

[ ] No Comments:

- Can the data for the above indicator be segregated for nationals and refugees?

[X] Yes Comments: could be segregated based on social security number for Jordanians and not Jordanians

[ ] No Comments:

- Is the data for Jordan reported in the same way as indicated above? *If you answer "No", please indicate in the comments section how it is being reported. If the indicator is not being reported in Jordan, please insert "Not reported" in the comments section*

[X] Yes Comments: Could be segregated based on diagnosis

[ ] No Comments:

- Is the data for this indicator available for the period extending from January 2019 to December 2021? (if yes, please provide the data)

[ ] Yes Comments:

[ ] No Comments:

Incidence of Low-birth-weight among newborns (%)

Definition:

The percentage of live births that weigh less than 2,500 g out of the total of live births during the same time period

Formula

$$\frac{\text{Number of live-born babies with birth weight less than 2,500 g}}{\text{Number of live births}} \times 100$$

Data collection & dissemination:

- Data dissemination every 5 years.

Method of measurement:

- **Service or facility records:** the proportion of live births with low birth weight, among births occurring in health institutions.
- **Household surveys:** DHS include questions on birth weight as well as the mothers' subjective assessment of the infant's size at birth (i.e. very large, larger than average, average, smaller than average, very small), for births in the last 5 to 10 years.

1. Collect Data on Denominator
Denominator: Number of live births

1.1 Include
- Number of live births

1.2 Exclude
- NA

2. Collect Data on Numerator
Numerator: Number of live-born babies with birth weight less than 2,500 g
2.1 Include
- Number of live-born babies with birth weight less than 2,500 g

2.2 Exclude
- NA

3. This indicator will be stratified based on:

- Sex
- Location (urban/rural)
- Education level: Maternal education,
- Wealth: Wealth quintile
- Boundaries: Administrative regions
- Boundaries: Health regions

4. Data Sources

4.1 Preferred data Sources:

- Facility reporting system
- Household surveys

4.2 Other data Sources:

- NA

5. Calculate:

Divide the Number of live-born babies with birth weight less than 2,500 g by the Number of live births and multiply by 100

6. Possible Limitations:

- NA

Data Collection Sheet

The following table contains detailed information and support from the literature on the measurement of the indicator.

Indicator	Low-birth-weight newborns (%)
Definition	The percentage of live births that weigh less than 2,500 g out of the total of live births during the same time period
Rationale	At the population level, the proportion of babies with a low birth weight is an indicator of a multifaceted public-health problem that includes long-term maternal malnutrition, ill health, hard work and poor health care in pregnancy. On an individual basis, low birth weight is an important predictor of newborn health and survival.
Type of indicator	Impact
Unit of measure	Percentage
Method of measurement	**Service or facility records:** the proportion of live births with low birth weight, among births occurring in health institutions.<br>**Household surveys:** DHS include questions on birth weight as well as the mothers' subjective assessment of the infant's size at birth (i.e. very large, larger than average, average, smaller than average, very small), for births in the last 5 to 10 years.
Formula	Number of live-born babies with birth weight less than 2,500g x100<br>Number of live births
Target	To be determined
Frequency	▪ Data dissemination every 5 years.
Possible Limitations	▪ NA
Denominator inclusion criteria	▪ Number of live births
Denominator exclusion criteria	▪ NA
Numerator inclusion criteria	▪ Number of live-born babies with birth weight less than 2,500 g
Numerator exclusion criteria	▪ NA
Data sources	▪ Facility reporting system<br>▪ Household surveys

References:

Indicator Metadata Registry Details. (2020). Retrieved 5 December 2020, from https://www.who.int/data/gho/indicator-metadata-registry/imr-details/76

For the above indicator, can you provide the below information:

- Can the data elements (numerator and denominator) be collected in the context of Jordan?

[X] Yes Comments:

[ ] No Comments:

- Are the data sources listed above applicable (i.e. information can be extracted from the suggested data source) in the context of Jordan? If no, please provide the source.

[ ] Yes Comments: Hospital based reports

[ ] No Comments:

- Can the data for the above indicator be segregated for nationals and refugees?

[X] Yes Comments: could be segregated based on social security number for Jordanians and not Jordanians

[ ] No Comments:

- Is the data for Jordan reported in the same way as indicated above? *If you answer "No", please indicate in the comments section how it is being reported. If the indicator is not being reported in Jordan, please insert "Not reported" in the comments section*

[X] Yes Comments:

[ ] No Comments:

- Is the data for this indicator available for the period extending from January 2019 to December 2021? (if yes, please provide the data)

[ ] Yes Comments:

[ ] No Comments:

Prevalence of anaemia in children aged 6–59 months (%)

Formula

$$\frac{\text{Number of children aged 6−59 months with a haemoglobin concentration less than 110 g/}}{\text{Total number of children aged 6−59 months}} \times 100$$

Data collection & dissemination:

- Continuous data collection.
- Data dissemination every 2-3 years.

Method of measurement:

The anaemia status of children is assessed using blood haemoglobin concentrations. In surveys, blood haemoglobin concentrations are typically measured using the direct cyanmethemoglobin method in a laboratory or with a portable, battery-operated, haemoglobin photometer in the field that uses the azide-methaemoglobin method.

Collect Data on Denominator

Denominator: Total number of children aged 0-5 years that were measured

1.1 Include

- NA

1.2 Exclude

- NA

2. Collect Data on Numerator

Numerator: Number of children aged 0-5 years with a haemoglobin concentration less than 110 g/L

2.1 Include

- NA

2.2 Exclude

- NA

3. This indicator will be stratified based on:

- Altitude

4. Data Sources

4.1 Preferred data Sources:

- Population based surveys

4.2 Other data Sources:

- Surveillance systems

5. Calculate:

Divide the number of children aged 6−59 months with a haemoglobin concentration less than 110 g/L by the total number of children aged 6−59 months and multiply by 100

6. Possible Limitations:

- NA

Data Collection Sheet

The following table contains detailed information and support from the literature on the measurement of the indicator.

Indicator	Prevalence of anaemia in children aged 6–59 months (%)
Definition	Percentage of children aged 6−59 months with a haemoglobin concentration less than 110 g/L, adjusted for altitude[10].
Rationale	Anaemia is highly prevalent globally, disproportionately affecting children and women of reproductive age. It negatively affects cognitive and motor development and work capacity, and among pregnant women iron deficiency anaemia is associated with adverse reproductive outcomes, including preterm delivery, low-birth-weight infants, and decreased iron stores for the baby, which may lead to impaired development. Iron deficiency is considered the most common cause of anaemia, but there are other nutritional and non-nutritional causes. Blood haemoglobin concentrations are affected by many factors, including altitude (metres above sea level), smoking, trimester of pregnancy, age and sex. Anaemia can be assessed by measuring blood haemoglobin, and when used in combination with other indicators of iron status, blood haemoglobin provides information about the severity of iron deficiency. The anaemia prevalence for the population is used to classify the public health significance of the problem[10].
Type of indicator	Outcome
Unit of measure	Percentage
Method of measurement	The anaemia status of children is assessed using blood haemoglobin concentrations. In surveys, blood haemoglobin concentrations are typically measured using the direct cyanmethemoglobin method in a laboratory or with a portable, battery-operated, haemoglobin photometer in the field that uses the azide-methaemoglobin method[10].
Formula	Number of children aged 6−59 months with a haemoglobin concentration less than 110 g/L/ X100<br>Total number of children aged 6−59 months
Target	To be determined
Frequency	▪ Data dissemination every 2-3 years<br>▪ Continuous data collection
Possible Limitations	▪ NA
Denominator inclusion criteria	▪ Total number of children aged 6−59 months that were measured
Denominator exclusion criteria	▪ NA
Numerator inclusion criteria	• Number of children aged 6−59 months with a haemoglobin concentration less than 110 g/L
Numerator exclusion criteria	▪ NA
Data sources	▪ Population based survey<br>▪ Surveillance systems

Indicator responsibility	Ministry of Health at the central level and lower administrative units

References:

Indicator Metadata Registry Details. (2020). Retrieved 17 December 2020, from https://www.who.int/data/gho/indicator-metadata-registry/imr-details/4801

For the above indicator, can you provide the below information:

- Can the data elements (numerator and denominator) be collected in the context of Jordan?

[X] Yes Comments:

[ ] No Comments:

- Are the data sources listed above applicable (i.e. information can be extracted from the suggested data source) in the context of Jordan? If no, please provide the source.

[ ] Yes Comments: Hospital based reports

[ ] No Comments:

- Can the data for the above indicator be segregated for nationals and refugees?

[X] Yes Comments: could be segregated based on social security number for Jordanians and not Jordanians

[ ] No Comments:

- Is the data for Jordan reported in the same way as indicated above? *If you answer "No", please indicate in the comments section how it is being reported. If the indicator is not being reported in Jordan, please insert "Not reported" in the comments section*

[ ] Yes Comments:

[x] No Comments: Total cases

- Is the data for this indicator available for the period extending from January 2019 to December 2021? (if yes, please provide the data)

[ ] Yes Comments:

[ ] No Comments:

Crude Incidence rate of cancer per 100,000 children aged 0-14 years

Definition:

New cases of cancer per 100,000 children aged 0-14

Formula:

Number of new cases of cancer among children aged 0-14 in a given calendar year x 100,000/ Total children aged 0-14 in the same calendar year

Method of measurement:

1. Collect Data on Denominator

1.1 Include
2 Children aged 0-14

2.1 Exclude

2. Collect Data on Numerator

2.1. Include
New cases of cancer among children aged 0-14

2.2. Exclude

3. Stratification Options
Age, sex

4. Preferred Data Sources
Cancer Registry

5. Other Data Sources

Data collection sheet

Indicator	Crude Incidence rate of cancer per 100,000 children aged 0-14 years<br><br>Age Standardized Incidence rate of cancer per 100,000 children aged 0-14 (alternative indicator if stratified by age)
Definition	New cases of cancer per 100,000 children aged 0-14
Rationale	Overall, in children less than 15 years of age, in the industrialized world, childhood cancer is listed as the 4th most common cause of death. Incidence trend patterns of common childhood cancers have recently been evaluated because of concerns that they may be on the rise: -For childhood leukaemia there was an abrupt increase in incidence between 1983 and 1984, however, rates have been declining between 1989 and 1995. -For brain and CNS cancers there was a modest increase in incidence from 1983 to 1986 and rates then stabilized between 1986 and 1995. The statistically significant increases that were reported in the mid 80's are now thought to be a result of diagnostic improvement or changes in reporting patterns. -For rare skin cancers such as dermatofibrosarcoms, there has been a 40% increase between 1975 and 1995. Data from the United States (US) shows that the incidence rate of cutaneous malignant melanoma (CMM) in 15-19 year olds increased 2.6% per year between 1973 and 1995, for a total increase of 85%
Type of indicator	
Unit of measure	
Formula	**Number of new cases of cancer among children aged 0-14 in a given calendar year x 100,000/ Total children aged 0-14 in the same calendar year**
Target	
Frequency of collection	Annual
Frequency of Dissemination	
Denominator inclusion criteria	Children aged 0-14
Denominator exclusion criteria	

Numerator inclusion criteria	New cases of children aged 0-14 diagnosed with cancer
Numerator exclusion criteria	
Preferred Data sources	Cancer Registry
Other Data Sources	
Limitations/comments	

References:

03_04_Metadata_Cancer.pdf (healthybelgium.be)

Australia's children, Cancer incidence and survival - Australian Institute of Health and Welfare (aihw.gov.au)

Cancer in Children | The Cancer Atlas

Microsoft PowerPoint - CANCER AND CHILDREN.ppt (who.int)

For the above indicator, can you provide the below information:

- Can the data elements (numerator and denominator) be collected in the context of Jordan?

☐ Yes Comments:

☐ No Comments:

- Are the data sources listed above applicable (i.e. information can be extracted from the suggested data source) in the context of Jordan? If no, please provide the source.

☐ Yes Comments:

☐ No Comments:

- Can the data for the above indicator be segregated for nationals and refugees?

☐ Yes Comments:

☐ No Comments:

- Is the data for Jordan reported in the same way as indicated above? *If you answer "No", please indicate in the comments section how it is being reported. If the indicator is not being reported in Jordan, please insert "Not reported" in the comments section*

☐ Yes Comments:

☐ No Comments:

- Is the data for this indicator available for the period extending from January 2019 to December 2021? (if yes, please provide the data)

☐ Yes Comments:

☐ No Comments:

Incidence rate of type 1 diabetes mellitus among children aged 0-14

Definition:

New cases of children aged 0-14 diagnosed with type 1 diabetes (Insulin treated) per 100,000 children

Formula:

(New cases of children aged 0-14 diagnosed with type 1 diabetes (Insulin treated)/ Total number of children aged 0-14)*100,000

Method of measurement:

1. Collect Data on Denominator
Total number of children aged 0-14

2.2 Include
3

3.1 Exclude

2. Collect Data on Numerator
Newly diagnosed children aged 0-14 with type 1 diabetes (insulin treated)

2.1. Include

2.2. Exclude

3. Stratification Options
Age, sex, geographical area

4. Preferred Data Sources
National Diabetes Registery

5. Other Data Sources

Data collection sheet

Indicator	Incidence rate of type 1 diabetes mellitus among children aged 0-14
Definition	New cases of children aged 0-14 diagnosed with type 1 diabetes (Insulin treated) per 100,000 children
Rationale	The monitoring of incidence and prevalence of type 1 diabetes in children and young people is important to improve capacity to plan treatment services, to target priority population groups, to track the impact of environmental change and to make decisions for cost-effective allocation of resources
Type of indicator	
Unit of measure	Rate
Formula	(New cases of children aged 0-14 diagnosed with type 1 diabetes (Insulin treated)/ Total number of children aged 0-14)*100,000
Target	
Frequency of collection	
Frequency of Dissemination	
Denominator inclusion criteria	Children aged 0-14
Denominator exclusion criteria	
Numerator inclusion criteria	Newly diagnosed cases for diabetes type 1(insulin treated) among children aged 0-14
Numerator exclusion criteria	
Preferred Data sources	National Diabetes Registry
Other Data Sources	
Limitations/comments	

References:

Type 1 Diabetes - Children and Young People (act.gov.au)

Recent incidence of type 1 diabetes mellitus in children 0-14 years in Newfoundland and Labrador, Canada climbs to over 45/100,000: a retrospective time trend study (mun.ca)

For the above indicator, can you provide the below information:

- Can the data elements (numerator and denominator) be collected in the context of Jordan?

☐ Yes Comments:

☐ No Comments:

- Are the data sources listed above applicable (i.e. information can be extracted from the suggested data source) in the context of Jordan? If no, please provide the source.

☐ Yes Comments:

☐ No Comments:

- Can the data for the above indicator be segregated for nationals and refugees?

☐ Yes Comments:

☐ No Comments:

- Is the data for Jordan reported in the same way as indicated above? *If you answer "No", please indicate in the comments section how it is being reported. If the indicator is not being reported in Jordan, please insert "Not reported" in the comments section*

☐ Yes Comments:

☐ No Comments:

- Is the data for this indicator available for the period extending from January 2019 to December 2021? (if yes, please provide the data)

☐ Yes Comments:

☐ No Comments:

Proportion of children aged 0-14 years with disability

Definition:

The disability rate is the proportion of people reporting a disability. This is the estimate of the number of people with a disability divided by the estimate of the total number of people with and without a disability.

The following disability types are provided under the detailed classification of disability type for children: hearing, seeing, speaking, use of technical equipment, chronic health problem, intellectual, psychiatric/psychological, special education, other.

Formula:

Number of children aged between 0-14 with disabilityx100 / Total number of children 0-14

Method of measurement:

1. Collect Data on Denominator

3.2 Include
4 Surveyed children aged between 0-14

4.1 Exclude

2. Collect Data on Numerator

2.1. Include
5 Surveyed children aged between 0-14 with disability

2.2. Exclude

3. Stratification Options
sex

4. Preferred Data Sources
Disability Survey

5. Other Data Sources

Data collection sheet

Indicator	Proportion of children aged 0-14 years with disability
Definition	The disability rate is the proportion of people reporting a disability. This is the estimate of the number of people with a disability divided by the estimate of the total number of people with and without a disability.<br><br>The following disability types are provided under the detailed classification of disability type for children: hearing, seeing, speaking, use of technical equipment, chronic health problem, intellectual, psychiatric/psychological, special education, other.
Rationale	
Type of indicator	
Unit of measure	
Formula	**Number of children aged between 0-14 with disabilityx100 / Total number of children 0-14**
Target	
Frequency of collection	
Frequency of Dissemination	
Denominator inclusion criteria	Children aged 0-14
Denominator exclusion criteria	
Numerator inclusion criteria	Children aged 0-14 with disability
Numerator exclusion criteria	
Preferred Data sources	Disability Survey
Other Data Sources	
Limitations/comments	

References:

DisabilitySurvey200606HOTP.pdf (cbg.co.nz)

For the above indicator, can you provide the below information:

- Can the data elements (numerator and denominator) be collected in the context of Jordan?

☐ Yes Comments:

☐ No Comments:

- Are the data sources listed above applicable (i.e. information can be extracted from the suggested data source) in the context of Jordan? If no, please provide the source.

☐ Yes Comments:

☐ No Comments:

- Can the data for the above indicator be segregated for nationals and refugees?

☐ Yes Comments:

☐ No Comments:

- Is the data for Jordan reported in the same way as indicated above? *If you answer "No", please indicate in the comments section how it is being reported. If the indicator is not being reported in Jordan, please insert "Not reported" in the comments section*

☐ Yes Comments:

☐ No Comments:

- Is the data for this indicator available for the period extending from January 2019 to December 2021? (if yes, please provide the data)

☐ Yes Comments:

☐ No Comments:

Prevalence of birth defects (per 10,000 live birth) by type

Definition:

Congenital anomalies, also commonly referred to as birth defects, congenital disorders, congenital malformations, or congenital abnormalities, are conditions of prenatal origin that are present at birth, potentially impacting an infant's health, development and/or survival.

Formula:

Number of birth defect cases among live birthsx10,000/ Total Live birth

Method of measurement:

1. Collect Data on Denominator

5.1 Include

6 Total Live birth

6.1 Exclude

2. Collect Data on Numerator

Number of birth defect cases among live births

2.1. Include

2.2. Exclude

3. Stratification Options

Maternal age, maternal race/ethnicity, infant sex, selected major types(Anencephaly, Cleft Lip with Cleft Palate, Cleft Lip without Cleft Palate, Cleft Palate without Cleft Lip, Gastroschisis, Hypoplastic Left Heart Syndrome, Hypospadias, Limb Deficiencies, Spina Bifida without Anencephaly, Tetralogy of Fallot, Trans+position of the Great Arteries (Vessels),Trisomy 21 (Down Syndrome))

4. Preferred Data Sources

Data are provided by state birth defects monitoring systems

5. Other Data Sources

Data collection sheet

Indicator	Prevalence (per 10,000 live births) of birth defects, by type
Definition	Congenital anomalies, also commonly referred to as birth defects, congenital disorders, congenital malformations, or congenital abnormalities, are conditions of prenatal origin that are present at birth, potentially impacting an infant's health, development and/or survival
Rationale	
Type of indicator	
Unit of measure	Rate per 10,000
Formula	Number of birth defect cases among live birthsx10,000/ Total Live birth
Target	
Frequency of collection	
Frequency of Dissemination	
Denominator inclusion criteria	
Denominator exclusion criteria	
Numerator inclusion criteria	
Numerator exclusion criteria	
Preferred Data sources	Data are provided by state birth defects monitoring systems
Other Data Sources	
Limitations/comments	-Not all states have birth defects monitoring programs<br>- Variability in the birth defects monitoring methodology among states means that there are limitations to combining, comparing, or contrasting birth defects statistics for national purposes<br>- Some birth defects may be underreported since not all defects are present at birth

	- Classification as isolated, multiple, or syndromic poses challenges for coding birth defects and ensuring cases are not over-represented.<br><br>- Address data reflects where the mother lived at delivery. This residence may not reflect true exposure at conception or during pregnancy if the mother moved during pregnancy.

References:

BirthDefectsIndicatorpdf.pdf

National Environmental Public Health Tracking Network | Indicators and Data (cdc.gov)

ANENCEPHALUS (nbdpn.org)

Congenital anomalies: Case definition and guidelines for data collection, analysis, and presentation of immunization safety data (nih.gov)

For the above indicator, can you provide the below information:

- Can the data elements (numerator and denominator) be collected in the context of Jordan?

☐ Yes Comments:

☐ No Comments:

- Are the data sources listed above applicable (i.e. information can be extracted from the suggested data source) in the context of Jordan? If no, please provide the source.

☐ Yes Comments:

☐ No Comments:

- Can the data for the above indicator be segregated for nationals and refugees?

☐ Yes Comments:

☐ No Comments:

- Is the data for Jordan reported in the same way as indicated above? *If you answer "No", please indicate in the comments section how it is being reported. If the indicator is not being reported in Jordan, please insert "Not reported" in the comments section*

☐ Yes Comments:

☐ No Comments:

- Is the data for this indicator available for the period extending from January 2019 to December 2021? (if yes, please provide the data)

☐ Yes Comments:

☐ No Comments:

SDG 2.2.1 Prevalence of stunting among children aged <5 years (%)

Formula

$$\frac{\text{Number of children aged 0-5 years that fall below minus two standard deviations from the median height-for-age of the WHO Child Growth Standards}}{\text{Total number of children aged 0-5 years that were measured}} \times 100$$

Data collection & dissemination:

- Annual data collection.
- Biannual data dissemination (one coinciding with the annual release of the regional/global estimates and at least one other update at another time of the year).

Method of measurement:

Survey estimates are based on standardized methodology using the WHO Child Growth Standards as described elsewhere (Ref: Anthro software manual). Global and regional estimates are based on methodology outlined in UNICEF-WHO-The World Bank: Joint child malnutrition estimates - Levels and trends (UNICEF/WHO/WB 2012)
Data are derived from re-analysis of Demographic and Health Surveys (DHS), Multiple Indicator Cluster Surveys (MICS) and Reproductive Health Surveys (RHS) micro-data which are publicly available using the WHO Child Growth Standards and standardized methodology recommended by the Technical Expert Advisory Group on Nutrition Monitoring. UNICEF, WHO and the World Bank group jointly review new data sources to update the country level estimates.

Collect Data on Denominator
Denominator: Total number of children aged 0-5 years that were measured

1.1 Include

- Total number of children aged 0-5 years that were measured

1.2 Exclude

- NA

2. Collect Data on Numerator
Numerator: Number of children aged 0-5 years that fall below minus two standard deviations from the median height-for-age of the WHO Child Growth Standards

2.1 Include

- Number of children aged 0-5 years that fall below minus two standard deviations from the median height-for-age of the WHO Child Growth Standards

2.2 Exclude

- NA

3. This indicator will be stratified based on:

- Age,
- Sex,
- Location (urban/rural)
- Boundaries: Administrative/ Health regions
- Wealth
- Mothers' education

4. Data Sources

4.1 Preferred data Sources:

- Nationally representative population-based surveys
- Household surveys
- Specific population surveys
- Surveillance systems

4.2 Other data Sources:

- NA

5. Calculate:

Divide the number of children aged 0-5 years that fall below minus two standard deviations from the median height-for-age of the WHO Child Growth Standards by the total number of children aged 0-5 years that were measured and multiply by 100

6. Possible Limitations:

- Sampling error and non-sampling error (e.g. measurement technical error, recording error etc.,). None of the two sources of errors have been fully taken into account for deriving estimates neither at country nor at regional and global levels.
- Surveys are carried out in a specific period of the year, usually over a few months. However, this indicator can be affected by seasonality, factors related to food availability (e.g. pre-harvest periods), disease (e.g. rainy season and diarrhoea, malaria, etc.), and natural disasters and conflicts. Hence, country-year estimates may not necessarily be comparable over time. Consequently, only latest estimates are provided.

Data Collection Sheet

The following table contains detailed information and support from the literature on the measurement of the indicator.

Indicator	Prevalence of stunting among children aged <5 years (%)
Alternate name	Children under 5 years who are stunted
Definition	Percentage of stunting (height-for-age less than -2 standard deviations of the WHO Child Growth Standards median) among children aged 0-5 years[9]
Rationale	Child growth is an internationally accepted outcome reflecting child nutritional status. Child stunting refers to a child who is too short for his or her age and is the result of chronic or recurrent malnutrition. Stunting is a contributing risk factor to child mortality and is also a marker of inequalities in human development. Stunted children fail to reach their physical and cognitive potential. Child stunting is one of the World Health Assembly nutrition target indicators. Child growth is the most widely used indicator of nutritional status in a community and is internationally recognized as an important public-health indicator for monitoring health in populations. In addition, children who suffer from growth retardation as a result of poor diets and/or recurrent infections tend to have a greater risk of suffering illness and death.[9]
Type of indicator	Impact
Unit of measure	Percentage
Method of measurement	Survey estimates are based on standardized methodology using the WHO Child Growth Standards as described elsewhere (Ref: Anthro software manual). Global and regional estimates are based on methodology outlined in UNICEF-WHO-The World Bank: Joint child malnutrition estimates - Levels and trends (UNICEF/WHO/WB 2012)<br><br>Data are derived from re-analysis of Demographic and Health Surveys (DHS), Multiple Indicator Cluster Surveys (MICS) and Reproductive Health Surveys (RHS) micro-data which are publicly available using the WHO Child Growth Standards and standardized methodology recommended by the Technical Expert Advisory Group on Nutrition Monitoring. UNICEF, WHO and the World Bank group jointly review new data sources to update the country level estimates
Formula	(Number of children aged 0-5 years that fall below minus two standard deviations from the median height-for-age of the WHO Child Growth Standards/Total number of children aged 0-5 years that were measured) x 100
Target	By 2030, end all forms of malnutrition, including achieving, by 2025, the internationally agreed targets on stunting and wasting in children under 5 years of age, and address the nutritional needs of adolescent girls, pregnant and lactating women and older persons
Frequency	▪ Data dissemination two annual updates<br>▪ Annual data collection
Possible Limitations	▪ Sampling error and non-sampling error (e.g. measurement technical error, recording error etc). None of the two sources of errors have

	been fully taken into account for deriving estimates neither at country nor at regional and global levels.<br>▪ Surveys are carried out in a specific period of the year, usually over a few months. However, this indicator can be affected by seasonality, factors related to food availability (e.g. pre-harvest periods), disease (e.g. rainy season and diarrhoea, malaria, etc.), and natural disasters and conflicts. Hence, country-year estimates may not necessarily be comparable over time. Consequently, only latest estimates are provided.[9]
Denominator inclusion criteria	▪ Total number of children aged 0-5 years that were measured
Denominator exclusion criteria	▪ NA
Numerator inclusion criteria	▪ Number of children aged 0-5 years that fall below minus two standard deviations from the median height-for-age of the WHO Child Growth Standards
Numerator exclusion criteria	▪ NA
Data sources	▪ Nationally representative population-based surveys<br>▪ Household surveys<br>▪ Specific population surveys<br>▪ Surveillance systems

References:

Indicator Metadata Registry Details. (2020). Retrieved 17 December 2020, from https://www.who.int/data/gho/indicator-metadata-registry/imr-details/3137

World Health organization, Global Health Observatory, Prevalence of stunting among children aged <5 years (%) https://www.who.int/data/gho/data/indicators/indicator-details/GHO/gho-jme-country-children-aged-5-years-stunted-(-height-for-age--2-sd)

World Health organization, Global Health Observatory, Health equity monitor: Stunting prevalence in children aged < 5 years (%) https://www.who.int/data/gho/data/indicators/indicator-details/GHO/hem-stunting-prevalence-in-children-aged-5-years

World Health organization, Global Health Observatory, Stunting prevalence among children under 5 years of age (%) (JME) https://www.who.int/data/gho/data/indicators/indicator-details/GHO/gho-jme-stunting-prevalence

Metadata-02-02-01.pdf (un.org)

For the above indicator, can you provide the below information: Not reported

- Can the data elements (numerator and denominator) be collected in the context of Jordan?

[x] Yes Comments:

[ ] No Comments:

- Are the data sources listed above applicable (i.e. information can be extracted from the suggested data source) in the context of Jordan? If no, please provide the source.

[ ] Yes Comments: Hospital based reports

[ ] No Comments:

- Can the data for the above indicator be segregated for nationals and refugees?

[X] Yes Comments:

[ ] No Comments:

- Is the data for Jordan reported in the same way as indicated above? *If you answer "No", please indicate in the comments section how it is being reported. If the indicator is not being reported in Jordan, please insert "Not reported" in the comments section*

[ ] Yes Comments:

[ ] No Comments: Not reported

- Is the data for this indicator available for the period extending from January 2019 to December 2021? (if yes, please provide the data)

[ ] Yes Comments:

[ ] No Comments:

Prevalence rate of gestational diabetes

Definition:

Gestational diabetes mellitus (GDM) is defined as a glucose intolerance which is first diagnosed in pregnancy and remains below the cutoff value for manifest diabetes

Formula:

Method of measurement:

1. Collect Data on Denominator

6.2 Include
7

7.1 Exclude

2. Collect Data on Numerator

2.1. Include

2.2. Exclude

3. Stratification Options

4. Preferred Data Sources

5. Other Data Sources

Data collection sheet

Indicator	
Definition	
Rationale	
Type of indicator	
Unit of measure	
Formula	
Target	
Frequency of collection	
Frequency of Dissemination	
Denominator inclusion criteria	
Denominator exclusion criteria	
Numerator inclusion criteria	
Numerator exclusion criteria	
Preferred Data sources	
Other Data Sources	
Limitations/comments	

References:

For the above indicator, can you provide the below information:

- Can the data elements (numerator and denominator) be collected in the context of Jordan?

☐ Yes Comments:

☐ No Comments:

- Are the data sources listed above applicable (i.e. information can be extracted from the suggested data source) in the context of Jordan? If no, please provide the source.

☐ Yes Comments:

☐ No Comments:

- Can the data for the above indicator be segregated for nationals and refugees?

☐ Yes Comments:

☐ No Comments:

- Is the data for Jordan reported in the same way as indicated above? *If you answer "No", please indicate in the comments section how it is being reported. If the indicator is not being reported in Jordan, please insert "Not reported" in the comments section*

☐ Yes Comments:

☐ No Comments:

- Is the data for this indicator available for the period extending from January 2019 to December 2021? (if yes, please provide the data)

☐ Yes Comments:

☐ No Comments:

Facility neonatal mortality rate disaggregated by birth weight >4000g, 2500-3999g, 2000-2499g, 1500-1999g, <1500g

Definition:

The percent of neonates (children within the first 28 completed days of life) who die in the facility, in a specific year or period.

Formula:

$$\frac{\text{Number of children who died during the first 28 days of life}}{\text{Total number of live births in the health facility}} \times 1000$$

Method of measurement:

1. Collect Data on Denominator

2.1 Include denominator

Total number of live births in the health facility

2.2 Exclude denominator

NA

2. Collect Data on Numerator

Number of children who died during the first 28 days of life

2.1 Include numerator

2.2 Exclude numerator

3. Stratification Options

Age in days/weeks, birth weight (i.e. >4000 g, 2500–3999 g, 2000–2499 g, 1500–1999 g, <1500 g), place of residence, sex, socioeconomic status

4. Preferred Data Sources
Hospital records and registers, outcome forms and death case reviews

5. Other Data Sources
Household surveys, population census

Data collection sheet

Indicator	Facility neonatal mortality rate disaggregated by birth weight >4000g, 2500-3999g, 2000-2499g, 1500-1999g, <1500g
Alternative Indicator	Facility neonatal mortality rate
Definition	The percent of neonates (children within the first 28 completed days of life) who die in the facility, in a specific year or period.
Rationale	Neonatal deaths (deaths among live births during the first 28 completed days of life) may be subdivided into early neonatal deaths, occurring during the first 7 days of life, and late neonatal deaths, occurring after the 7th day but before the 28th completed day of life.<br><br>This is a key outcome indicator for newborn care and directly reflects prenatal, intrapartum, and neonatal care. When collected at the facility level, the indicator can be used to monitor the outcome of delivery and newborn care in health facilities. Reliable estimates for individual facilities can only be obtained for very large facilities if there are large numbers of deliveries and neonatal admissions.
Type of indicator	Outcome
Unit of measure	Per 1000
Formula	(Number of children who died during the first 28 days of life/Total number of live births in the health facility) x 1000
Target	Not specified
Frequency of collection	Annual
Frequency of Dissemination	
Denominator inclusion criteria	Total number of live births in the health facility
Denominator exclusion criteria	

Numerator inclusion criteria	Number of children who died during the first 28 days of life
Numerator exclusion criteria	
Preferred Data sources	Hospital records and registers, outcome forms and death case reviews
Other Data Sources	
Limitations/comments	Data from routine health information systems: Routine health information systems may collect data for this indicator to obtain estimates of the facility neonatal mortality rate. Facility data are not recommended for estimating the neonatal mortality rate for the general population, because in many settings, many neonatal deaths and live births occur outside the health system, which will cause substantial selection bias.<br><br>Data from household surveys: Calculations are based on full birth history, whereby women are asked for the date of birth of each of their children, whether each child is still alive and if not the age at death.<br><br>To ensure consistency with mortality rates in children younger than 5 years (under-five mortality rate) produced by the UN-IGME and to account for variation in survey-to-survey measurement errors, country data points for the under-five and neonatal mortality rates were rescaled for all years to match the latest time series estimates of the under-five mortality rate produced by UN-IGME. This rescaling assumes that the proportionate measurement error in neonatal and under-five mortality rates is equal for each data point.<br><br>The following multilevel statistical model was then applied to estimate neonatal mortality rates: log (neonatal mortality rate/1000) = $\alpha 0 + \beta 1$*log(under-five mortality rate/1000) + $\beta 2$*[log(under-five mortality rate/1000)] 2) with random effects parameters or both level and trend regression parameters, and random effects parameters influenced by the country itself.<br><br>For countries with high-quality civil registration data for neonatal deaths – defined as (i) 100% complete for adults and only civil registration data is used for child mortality, (ii) population greater than 800 000, (iii) and with at least three civil registration data points for the periods 1990−1994, 1995−1999, 2000−2004 and 2005 onwards – we used the same basic equation, but with random effects parameters for both level and trend regression parameters, and random effects parameters influenced by the country itself.<br><br>Predominant type of statistics: adjusted and predicted.

	These neonatal rates are estimates, derived from the estimated UN-IGME neonatal rate infant population for World population prospects to calculate the live births; hence they are not necessarily the same as the official national statistics.

References:

Measure Evaluation, Results based financing, Facility neonatal mortality rate, https://www.measureevaluation.org/rbf/indicator-collections/health-outcome-impact-indicators/neonatal-mortality-rate.html

For the above indicator, can you provide the below information:

- Can the data elements (numerator and denominator) be collected in the context of Jordan?

[X] Yes Comments:

[ ] No Comments:

- Are the data sources listed above applicable (i.e. information can be extracted from the suggested data source) in the context of Jordan? If no, please provide the source.

[ ] Yes Comments: Hospital based reports

[ ] No Comments:

- Can the data for the above indicator be segregated for nationals and refugees?

[X] Yes Comments: could be segregated based on social security number for Jordanians and not Jordanians

[ ] No Comments:

- Is the data for Jordan reported in the same way as indicated above? *If you answer "No", please indicate in the comments section how it is being reported. If the indicator is not being reported in Jordan, please insert "Not reported" in the comments section*

[x] Yes Comments: Could be segregated from collected data

[ ] No Comments:

- Is the data for this indicator available for the period extending from January 2019 to December 2021? (if yes, please provide the data)

[ ] Yes Comments:

[ ] No Comments:

Case fatality rate for Postpartum Haemorrhage in health facilities (%)

Definition:

Proportion of women admitted to health facilities with postpartum hemorrhage (defined as a blood loss of 500 ml or more within 24 hours after birth) , or who develop postpartum hemorrhage after admission, and die before discharge.

Number of cases or deaths classified under the ICD 10 codes O71-O72

Formula:

(Number of women admitted to health facilities with postpartum hemorrhage, or who develop postpartum hemorrhage after admission, and die before discharge/ Total number of postpartum women in health facility)*100

Method of measurement:

1. Collect Data on Denominator

7.2 Include
8 Total number of postpartum women in health facility

8.1 Exclude

2. Collect Data on Numerator

2.1. Include
Number of women admitted to health facilities with postpartum hemorrhage, or who develop postpartum hemorrhage after admission, and die before discharge

2.2. Exclude

3. Stratification Options
Provider type (public/private)

4. Preferred Data Sources
Facility reporting system

5. Other Data Sources

Data collection sheet

Indicator	
Definition	Proportion of women admitted to health facilities with postpartum hemorrhage(defined as a blood loss of 500 ml or more within 24 hours after birth) , or who develop postpartum hemorrhage after admission, and die before discharge.<br><br>Number of cases or deaths classified under the ICD 10 codes O71-O72
Rationale	This indicators shows quality of care at the health facilities, performance of the health facilities, and maternal care system
Type of indicator	
Unit of measure	Percentage
Formula	(Number of women admitted to health facilities with postpartum hemorrhage, or who develop postpartum hemorrhage after admission, and die before discharge/ Total number of postpartum women in health facility)*100
Target	
Frequency of collection	
Frequency of Dissemination	
Denominator inclusion criteria	Total number of postpartum women in health facility
Denominator exclusion criteria	
Numerator inclusion criteria	Number of women admitted to health facilities with postpartum hemorrhage, or who develop postpartum hemorrhage after admission, and die before discharge
Numerator exclusion criteria	
Preferred Data sources	Facility reporting system
Other Data Sources	
Limitations/comments	

References:

Number of postpartum haemorrhage (who.int)

Case fatality rate for Postpartum Haemorrhage in health facilities (%) (who.int)

For the above indicator, can you provide the below information:

- Can the data elements (numerator and denominator) be collected in the context of Jordan?

☐ Yes Comments:

☐ No Comments:

- Are the data sources listed above applicable (i.e. information can be extracted from the suggested data source) in the context of Jordan? If no, please provide the source.

☐ Yes Comments:

☐ No Comments:

- Can the data for the above indicator be segregated for nationals and refugees?

☐ Yes Comments:

☐ No Comments:

- Is the data for Jordan reported in the same way as indicated above? *If you answer "No", please indicate in the comments section how it is being reported. If the indicator is not being reported in Jordan, please insert "Not reported" in the comments section*

☐ Yes Comments:

☐ No Comments:

- Is the data for this indicator available for the period extending from January 2019 to December 2021? (if yes, please provide the data)

☐ Yes Comments:

☐ No Comments:

Dimension 2

SDG2.2.3 Prevalence of anaemia in women aged 15-49, by pregnancy status (%)

Formula

$$\frac{\text{Number of women ages 15 to 49 with inadequate hemoglobin levels [<12 g/dl non-pregnant women and <11g/dl pregnant women]}}{\text{Total number of women ages of 15 to 49 screened for hemoglobin levels during a specified period}} \times 100$$

Data collection & dissemination:

- Continuous data collection
- Data Dissemination every 2-3 years

Method of measurement:

The. anaemia status of women is assessed using blood haemoglobin concentrations. In surveys, blood haemoglobin concentrations are typically measured using the direct cyanmethemoglobin method in a laboratory or with a portable, battery-operated, haemoglobin photometer in the field that uses the azide-methaemoglobin method.

Collect Data on Denominator
Denominator: Total number of women ages of 15 to 49 screened for hemoglobin levels during a specified period

1.1 Include
- NA

1.2 Exclude
- NA

2. Collect Data on Numerator
Numerator: Number of women ages 15 to 49 with inadequate hemoglobin levels [<12 g/dl non-pregnant women and <11g/dl pregnant women]

2.1 Include
NA

2.2 Exclude
- NA

3. This indicator will be stratified based on:

- Altitude
- Smoking
- Pregnancy status

4. Data Sources

4.1 Preferred data Sources:

- Population based surveys

4.2 Other data Sources:

- Surveillance systems

5. Calculate:

Divide the number of women ages 15 to 49 with inadequate hemoglobin levels by the total number of women ages of 15 to 49 screened for hemoglobin levels and multipy by 100

6. Possible Limitations:

NA

Data Collection Sheet

The following table contains detailed information and support from the literature on the measurement of the indicator.

Indicator	Prevalence of anaemia in women aged 15-49, by pregnancy status (%)
Definition	Percentage of women aged 15−49 years with a haemoglobin concentration less than 120 g/L for non-pregnant women and lactating women, and less than 110 g/L for pregnant women, adjusted for altitude and smoking[11].
Rationale	Anaemia is highly prevalent globally, disproportionately affecting children and women of reproductive age. It negatively affects cognitive and motor development and work capacity, and among pregnant women iron deficiency anaemia is associated with adverse reproductive outcomes, including preterm delivery, low-birth-weight infants, and decreased iron stores for the baby, which may lead to impaired development. Iron deficiency is considered the most common cause of anaemia, but there are other nutritional and non-nutritional causes. Blood haemoglobin concentrations are affected by many factors, including altitude (metres above sea level), smoking, trimester of pregnancy, age and sex. Anaemia can be assessed by measuring blood haemoglobin, and when used in combination with other indicators of iron status, blood haemoglobin provides information about the severity of iron deficiency. The anaemia prevalence for the population is used to classify the public health significance of the problem[11].
Type of indicator	Outcome
Unit of measure	Percentage
Method of measurement	The anaemia status of women is assessed using blood haemoglobin concentrations. In surveys, blood haemoglobin concentrations are typically measured using the direct cyanmethemoglobin method in a laboratory or with a portable, battery-operated, haemoglobin photometer in the field that uses the azide-methaemoglobin method[11].
Formula	(Number of women ages 15 to 49 with inadequate hemoglobin levels [<12 g/dl non-pregnant women and <11g/dl pregnant women]/ Total number of women ages of 15 to 49 screened for hemoglobin levels during a specified period) x 100
Target	To be determined
Frequency	▪ Continuous data collection<br>▪ Data Dissemination every 2-3 years
Possible Limitations	▪ NA
Denominator inclusion criteria	▪ Total number of women ages of 15 to 49 screened for hemoglobin levels during a specified period
Denominator exclusion criteria	▪ NA
Numerator inclusion criteria	▪ Number of women ages 15 to 49 with inadequate hemoglobin levels [<12 g/dl non-pregnant women and <11g/dl pregnant women]
Numerator exclusion criteria	▪ NA
Data sources	▪ Population-based surveys<br>▪ Surveillance systems

References:

Indicator Metadata Registry Details. (2020). Retrieved 17 December 2020, from https://www.who.int/data/gho/indicator-metadata-registry/imr-details/4552

Metadata-02-02-03.pdf (un.org)For the above indicator, can you provide the below information:

- Can the data elements (numerator and denominator) be collected in the context of Jordan?

[X] Yes Comments:

[ ] No Comments:

- Are the data sources listed above applicable (i.e. information can be extracted from the suggested data source) in the context of Jordan? If no, please provide the source.

[ ] Yes Comments: Hospital based reports

[ ] No Comments:

- Can the data for the above indicator be segregated for nationals and refugees?

[X] Yes Comments: could be segregated based on social security number for Jordanians and not Jordanians

[ ] No Comments:

- Is the data for Jordan reported in the same way as indicated above? *If you answer "No", please indicate in the comments section how it is being reported. If the indicator is not being reported in Jordan, please insert "Not reported" in the comments section*

[ ] Yes Comments:

[x] No Comments: Total cases

- Is the data for this indicator available for the period extending from January 2019 to December 2021? (if yes, please provide the data)

[ ] Yes Comments:

[ ] No Comments:

Prevalence of vitamin B12 deficiency among children aged 6-59 months

Definition:

Vitamin B-12 deficiency was defined as serum or plasma total vitamin B-12 concentrations <148 pmol/L.

Formula:

Total number of children aged 6-59 month with vitamin B12 deficiency *100/ Total number of children aged 6-59

Method of measurement:

1. Collect Data on Denominator

8.2 Include
9

9.1 Exclude

2. Collect Data on Numerator

2.1. Include

2.2. Exclude

3. Stratification Options
Sex, age

4. Preferred Data Sources
Population based study

5. Other Data Sources

Data collection sheet

Indicator	Prevalence of vitamin B12 deficiency among children aged 6-59 months
Definition	Total number of children aged 6-59 month with vitamin B12 deficiency *100/ Total number of children aged 6-59
Rationale	
Type of indicator	
Unit of measure	
Formula	Total number of children aged 6-59 month with vitamin B12 deficiency *100/ Total number of children aged 6-59
Target	
Frequency of collection	
Frequency of Dissemination	
Denominator inclusion criteria	
Denominator exclusion criteria	
Numerator inclusion criteria	
Numerator exclusion criteria	
Preferred Data sources	Population based study
Other Data Sources	
Limitations/comments	No clear metadata

References:

Vitamin B-12 and Cognition in Children (nih.gov)

Prevalence and Disparities in Folate and Vitamin B12 Deficiency Among Preschool Children in Guatemala (nih.gov)

For the above indicator, can you provide the below information:

- Can the data elements (numerator and denominator) be collected in the context of Jordan?

☐ Yes Comments:

☐ No Comments:

- Are the data sources listed above applicable (i.e. information can be extracted from the suggested data source) in the context of Jordan? If no, please provide the source.

☐ Yes Comments:

☐ No Comments:

- Can the data for the above indicator be segregated for nationals and refugees?

☐ Yes Comments:

☐ No Comments:

- Is the data for Jordan reported in the same way as indicated above? *If you answer "No", please indicate in the comments section how it is being reported. If the indicator is not being reported in Jordan, please insert "Not reported" in the comments section*

☐ Yes Comments:

☐ No Comments:

- Is the data for this indicator available for the period extending from January 2019 to December 2021? (if yes, please provide the data)

☐ Yes Comments:

☐ No Comments:

Exclusive breastfeeding under 6 months (%)

Definition:

Proportion of infants 0–5 months of age (0 to < 6 months) who are fed exclusively with breast milk.

Formula:

(Infants 0–5 months of age who received only breast milk during the previous day)/Infants 0–5 months of age) x 100

Method of measurement:

Percentage of infants 0–5 months of age who are fed exclusively with breast milk = (Infants 0–5 months of age who received only breast milk during the previous day/Infants 0–5 months of age) x 100 . Demographic and Health Surveys (DHS) and Multiple Indicator Cluster Surveys (MICS) include questions on liquids and foods given the previous day, and number of milk feeds the previous day, to learn if the child is being exclusively breastfed.

1. Collect Data on Denominator

9.2 Include
Infants 0–5 months of age

9.3 Exclude

2. Collect Data on Numerator
2.1. Include
Infants 0–5 months of age who received only breast milk
2.2. Exclude

3. Stratification Options
Sex, Location (urban/rural), Boundaries : Administrative regions, Boundaries : Health regions

4. Preferred Data Sources
Household surveys
Specific population surveys
Surveillance systems

5. Other Data Sources

Data collection sheet

Indicator	Exclusive breastfeeding under 6 months (%)
Definition	Proportion of infants 0–5 months of age (0 to < 6 months) who are fed exclusively with breast milk.
Rationale	This indicator belongs to a set of indicators whose purpose is to measure infant and young child feeding practices, policies and programmes. Infant and young child feeding practices directly affect the nutritional status, development and survival of children. Infant and young child feeding practices directly affect the nutritional status and survival of children. Exclusive breastfeeding is the single most effective intervention to improve the survival of children. Improving infant and young child feeding practices is therefore critical to improved nutrition, health and development of children
Type of indicator	Outcome
Unit of measure	
Formula	(Infants 0–5 months of age who received only breast milk during the previous day/Infants 0–5 months of age) x 100
Target	
Frequency of collection	3-5 years
Frequency of Dissemination	
Denominator inclusion criteria	Infants 0–5 months of age
Denominator exclusion criteria	
Numerator inclusion criteria	Infants 0–5 months of age who received only breast milk
Numerator exclusion criteria	
Preferred Data sources	Household surveys<br>Specific population surveys<br>Surveillance systems
Other Data Sources	
Limitations/comments	

References:

Exclusive breastfeeding under 6 months (%) (who.int)

For the above indicator, can you provide the below information:

- Can the data elements (numerator and denominator) be collected in the context of Jordan?

☐ Yes Comments:

☐ No Comments:

- Are the data sources listed above applicable (i.e. information can be extracted from the suggested data source) in the context of Jordan? If no, please provide the source.

☐ Yes Comments:

☐ No Comments:

- Can the data for the above indicator be segregated for nationals and refugees?

☐ Yes Comments:

☐ No Comments:

- Is the data for Jordan reported in the same way as indicated above? *If you answer "No", please indicate in the comments section how it is being reported. If the indicator is not being reported in Jordan, please insert "Not reported" in the comments section*

☐ Yes Comments:

☐ No Comments:

- Is the data for this indicator available for the period extending from January 2019 to December 2021? (if yes, please provide the data)

☐ Yes Comments:

☐ No Comments:

Out-of-school rate

Definition:

Out-of-school children rate (SDG4.1.4) – Percentage of children or young people in the official age range for a given level of education who are not attending either pre-primary(5+), primary(6+ to 11+), secondary(12+ to 15+), or higher levels of education

Formula:

Method of measurement:

The number of students of the official age for the given level of education enrolled in pre-primary, primary, secondary or higher levels of education is subtracted from the total population of the same age. The result is expressed as a percentage of the population of the official age for the respective level of education. For primary, lower secondary and upper secondary education, the official age groups for the respective level of education are used in the indicator calculation. For pre-primary education, the indicator is calculated for children aged one year before the official age of entry into primary education.

1. Collect Data on Denominator

9.4 Include
10

10.1 Exclude

2. Collect Data on Numerator

2.1. Include

2.2. Exclude

3. Stratification Options
By age or age-group and sex (administrative data); by age or age-group and sex, location, and socio-economic status (household surveys) and others as available

4. Preferred Data Sources
School or household surveys

5. Other Data Sources

Data collection sheet

Indicator	Out of school rate
Definition	Out-of-school rate (SDG4.1.4) – Percentage of children or young people in the official age range for a given level of education who are not attending either pre-primary(5+), primary(6+ to 11+), secondary(12+ to 15+), or higher levels of education
Rationale	To identify the size of the population in the official age range for the given level of education who are not enrolled in school in order that they can be better targeted and appropriate policies can be put in place to ensure they have access to education.
Type of indicator	
Unit of measure	
Formula	
Target	
Frequency of collection	
Frequency of Dissemination	
Denominator inclusion criteria	
Denominator exclusion criteria	
Numerator inclusion criteria	
Numerator exclusion criteria	
Preferred Data sources	
Other Data Sources	
Limitations/comments	Inconsistencies between enrolment and population data from different sources may result in inaccurate estimates of out-of-school children and adolescents. Data from household surveys conducted late in the school year where ages are recorded at the enumeration date may result in over-estimates. For primary, lower secondary and upper secondary education, the official age groups for the respective level of education are used in the indicator calculation. The out-of-school rate for pre-primary

	education is calculated for children aged one year before the official age of entry into primary education.

References:

Children Out of School: Malaysia, The Sabah Context (unicef.org)

Out-of-school rate (1 year before primary, primary education, lower secondary education, upper secondary education) | UNESCO UIS

new-methodology-shows-258-million-children-adolescents-and-youth-are-out-school.pdf (unesco.org)

For the above indicator, can you provide the below information:

- Can the data elements (numerator and denominator) be collected in the context of Jordan?

☐ Yes Comments:

☐ No Comments:

- Are the data sources listed above applicable (i.e. information can be extracted from the suggested data source) in the context of Jordan? If no, please provide the source.

☐ Yes Comments:

☐ No Comments:

- Can the data for the above indicator be segregated for nationals and refugees?

☐ Yes Comments:

☐ No Comments:

- Is the data for Jordan reported in the same way as indicated above? *If you answer "No", please indicate in the comments section how it is being reported. If the indicator is not being reported in Jordan, please insert "Not reported" in the comments section*

☐ Yes Comments:

☐ No Comments:

- Is the data for this indicator available for the period extending from January 2019 to December 2021? (if yes, please provide the data)

☐ Yes Comments:

☐ No Comments:

Dimension 3

Births by caesarean section (%)

Definition:

Percentage of births by caesarean section among all live births in a given time period.

Formula:

The number of women having given birth by caesarean section/ The number of live births to women surveyed x 100%

Method of measurement:

Household surveys: birth history—detailed questions on the last-born child or all children a woman has given birth to during a given period preceding the survey (usually 3 to 5 years), including characteristics of the birth(s). The number of live births to women surveyed provides the denominator. Service or facility records: the number of women having given birth by caesarean section (numerator). Census projections or, in some cases, vital registration data can be used to provide the denominator (numbers of live births).

1. Collect Data on Denominator

10.2 Include

11 The number of live births to women surveyed

11.1 Exclude

2. Collect Data on Numerator

2.1. Include

The number of women having given birth by caesarean section

2.2. Exclude

3. Stratification Options

4. Preferred Data Sources
Facility reporting system
Household surveys
5. Other Data Sources

Data collection sheet

Indicator	Births by caesarean section (%)
Definition	Percentage of births by caesarean section among all live births in a given time period.
Rationale	The percentage of births by caesarean section is an indicator of access to and use of emergency health care during childbirth.
Type of indicator	Outcome
Unit of measure	
Formula	The number of women having given birth by caesarean section/ The number of live births to women surveyed x 100%
Target	
Frequency of collection	Annual
Frequency of Dissemination	
Denominator inclusion criteria	The number of live births to women surveyed
Denominator exclusion criteria	
Numerator inclusion criteria	The number of women having given birth by caesarean section
Numerator exclusion criteria	
Preferred Data sources	Facility reporting system<br>Household surveys
Other Data Sources	
Limitations/comments	

References:

Births by caesarean section (%) (who.int)

For the above indicator, can you provide the below information:

- Can the data elements (numerator and denominator) be collected in the context of Jordan?

☐ Yes Comments:

☐ No Comments:

- Are the data sources listed above applicable (i.e. information can be extracted from the suggested data source) in the context of Jordan? If no, please provide the source.

☐ Yes Comments:

☐ No Comments:

- Can the data for the above indicator be segregated for nationals and refugees?

☐ Yes Comments:

☐ No Comments:

- Is the data for Jordan reported in the same way as indicated above? *If you answer "No", please indicate in the comments section how it is being reported. If the indicator is not being reported in Jordan, please insert "Not reported" in the comments section*

☐ Yes Comments:

☐ No Comments:

- Is the data for this indicator available for the period extending from January 2019 to December 2021? (if yes, please provide the data)

☐ Yes Comments:

☐ No Comments:

Postpartum care coverage for mothers (%)

Formula

$$\frac{\text{Number of women who received postpartum or postabortion FP counselling}}{\text{Total number of women who received postpartum or postabortion care in a health facility or community-based program during a specified time period}} \times 100$$

Data collection & dissemination:

- Collect information on this indicator every 3-5 years.
- Dissemination of information on annual basis.

Method of measurement:

Female survey respondents are asked about their most recent live birth, and when, if at all, their health was checked following delivery. This should include births at home and those in a health facility.

1. Collect Data on Denominator
Denominator: Total number of women who received postpartum or postabortion care in a health facility or community-based program during a specified time period

1.1 Include
- NA

1.2 Exclude
- NA

1.3 Denominator Collection modality

2. Collect Data on Numerator
Numerator: Number of women who received postpartum or postabortion FP counselling

2.1 Include
- NA

2.2 Exclude
- NA

3. This indicator will be stratified based on:
- Location (urban/rural)
- Education level : Maternal education
- Wealth : Wealth quintile
- postpartum or postabortion
- Age
- Type of facility or program (public, private, non-governmental, community-based)

4. Data Sources

4.1 Preferred data Sources:

- Household surveys such as Demographic and Health Surveys (DHS) and Multiple Indicator Cluster Surveys (MICS).
- UNICEF database

Other data Sources:

- Facility reporting system

5. Calculate:

Divide the Number of women who received postpartum or postabortion FP counselling by the Total number of women who received postpartum or postabortion care in a health facility or community-based program during a specified time period then multiply by 100

6. Possible Limitations:

- This indicator does not measure the quality of the FP counselling services, and whether the facility has methods available, makes referrals, or follows up with women on the adoption, correct use, or continuation of FP methods.
- Women who deliver at home and/or do not receive postpartum care will not be included in the calculation of this indicator, nor will women who do not receive postabortion care, particularly in settings where abortion is illegal or having an abortion is highly stigmatized and women do not seek care for complications.

Data Collection Sheet

The following table contains detailed information and support from the literature on the measurement of the indicator.

Indicator	Postpartum care coverage for mothers (%)
Alternate name	Percent of women receiving postpartum care by a skilled health personnel within two days of childbirth<br>OR<br>% of postpartum service utilization by previous antenatal clients with an identified expected delivery date<br>OR<br>Percent women receiving postpartum/ postabortion family planning counseling (as a percent of women seen)
Definition	Percentage of women age 15-49 years who received a health check within 2 days after delivery of their most recent live birth in the last 2 years.
Rationale	Many maternal and newborn deaths occur within a few hours after birth. Postnatal care contacts, especially within the first few days following birth, are a critical opportunity for improving maternal and newborn health and survival and for provision of information about birth spacing.
Type of indicator	Output
Unit of measure	Percentage
Method of measurement	Female survey respondents are asked about their most recent live birth, and when, if at all, their health was checked following delivery. This should include births at home and those in a health facility.
Formula	(Number of women who received postpartum or postabortion FP counselling / Total number of women who received postpartum or postabortion care in a health facility or community-based program during a specified time period) x 100
Target	NA
Frequency	▪ Collect information on this indicator every 3-5 years.<br>▪ Dissemination of information on annual basis.
Possible Limitations	▪ This indicator does not measure the quality of the FP counselling services, and whether the facility has methods available, makes referrals, or follows up with women on the adoption, correct use, or continuation of FP methods.<br>▪ Women who deliver at home and/or do not receive postpartum care will not be included in the calculation of this indicator, nor will women who do not receive postabortion care, particularly in settings where abortion is illegal or having an abortion is highly stigmatized and women do not seek care for complications.
Denominator inclusion criteria	Total number of women who received postpartum or postabortion care in a health facility or community-based program during a specified time period
Denominator exclusion criteria	NA
Numerator inclusion criteria	Number of women who received postpartum or postabortion FP counselling
Numerator exclusion criteria	NA
Data sources	▪ Household surveys such as Demographic and Health Surveys (DHS)

	and Multiple Indicator Cluster Surveys (MICS).<br>▪ UNICEF database<br>▪ Facility reporting system

References:

WHO, MATERNAL, NEWBORN, CHILD AND ADOLESCENT HEALTH AND AGEING Data portal https://www.who.int/data/maternal-newborn-child-adolescent-ageing/indicator-explorer-new/mca/proportion-of-mothers-who-had-postnatal-contact-with-a-health-provider-within-2-days-of-delivery

USAID, Percent women receiving postpartum/ postabortion family planning counseling (as a percent of women seen) https://www.data4impactproject.org/prh/womens-health/safe-motherhood/percent-women-receiving-postpartum-postabortion-family-planning-counseling-as-a-percent-of-women-seen/

For the above indicator, can you provide the below information:

- Can the data elements (numerator and denominator) be collected in the context of Jordan?

[X] Yes Comments:

[ ] No Comments:

- Are the data sources listed above applicable (i.e. information can be extracted from the suggested data source) in the context of Jordan? If no, please provide the source.

[ ] Yes Comments: Hospital based reports

[ ] No Comments:

- Can the data for the above indicator be segregated for nationals and refugees?

[X] Yes Comments: could be segregated based on social security number for Jordanians and not Jordanians

[ ] No Comments:

- Is the data for Jordan reported in the same way as indicated above? *If you answer "No", please indicate in the comments section how it is being reported. If the indicator is not being reported in Jordan, please insert "Not reported" in the comments section*

[ ] Yes Comments:

[x] No Comments: Total cases

- Is the data for this indicator available for the period extending from January 2019 to December 2021? (if yes, please provide the data)

[ ] Yes Comments:

[ ] No Comments:

Referral rates for women with obstetric complications

Formula

$$\frac{\text{Number of women with a potential or actual obstetric complication moved to another site for care}}{\text{Number of all women with obstetric complications (or deliveries or live births) in the same area and within the same time frame as in the numerator}} \times 100$$

Data collection & dissemination:

- NA

Method of measurement:

There are many types of referral possible, including those from the family directly, or between facilities:

1. Self/family referral to: Traditional Birth Attendants/ Healthcare worker training (TBA/CHW), First level skilled health care provider, Basic EOC facility, Comprehensive EOC facility
2. TBA/CHW to: First level skilled delivery care provider (SDC), Basic EOC facility, Comprehensive EOC facility
3. First level SDC to: Basic EOC facility, Comprehensive EOC facility,
4. Comprehensive EOC facility to: Tertiary center

1. Collect Data on Denominator

Denominator: Number of all women with obstetric complications (or deliveries or live births) in the same area and within the same time frame as in the numerator

1.1 Include

- Number of all women with obstetric complications (or deliveries or live births) in the same area and within the same time frame as in the numerator

1.2 Exclude

- NA

1.3 Denominator Collection modality

Gathered at community level, the denominator is anticipated to be all live births, although "all women with complications" could be calculated. Obviously if the data are being drawn from birth register for a facility-based Referral Rate, the denominator could be all deliveries in the facility, or all women with complications as diagnosed in the facility.

2. Collect Data on Numerator

Numerator: Number of women with a potential or actual obstetric complication moved to another site for care

2.1 Include

- Number of women with a potential or actual obstetric complication moved to another site for care

2.2 Exclude

- women with "potential risk factor"

3. This indicator will be stratified based on:

- Referral source
- Traditional Birth Attendants/ Healthcare worker training

4. Data Sources

4.1 Preferred data Sources:

- Health facility registers
- Antenatal cards
- delivery records
- vehicle log books
- special referral forms
- community surveys
- community data collection systems

4.2 Other data Sources:

- Case histories

5. Calculate:

Divide the number of women with a potential or actual obstetric complication moved to another site for care by the number of all women with
obstetric complications (or deliveries or live births) in the same area and within the same time frame as in the numerator. Then multiply the number by 100.

6. Possible Limitations:

- NA

Data Collection Sheet

The following table contains detailed information and support from the literature on the measurement of the indicator.

Indicator	Referral rates for women with obstetric complications
Definition	Proportion of women with potential or actual obstetric complications moving from one level of care to another (e.g., community to basic EOC facility or to comprehensive EOC facility)
Rationale	Referral Rates indicate both access and quality of care in a particular facility. They contribute to the evaluation of communications campaigns aimed at improving referral at the community level (self-referral, or by TBA/CHW). A variety of factors are known to affect Referral Rates, including the perceived quality of care at the Basic and Comprehensive EOC facilities, availability of transport, costs of transport and of services at the referral site, the population's knowledge of complications and referral sites, linkages between service levels and mechanisms that affect referral (e.g., prenatal care referral), and special attention to referral needs (e.g., maternity waiting homes). Experience with referral rates is minimal. The most useful information has come from registers at the facility level for a defined group of conditions or for a single complication. They can be expressed as a percent of deliveries or of women with complications in the facility. Rates are not likely to be useful for comparison across facilities, districts, or countries. Receiving timely information about FP is a critical step in ensuring optimal birth spacing. Delivery in a facility is an important opportunity to provide information on postpartum FP, especially since coverage of postnatal care is often low (WHO 2013, Pfitzer 2015). Systematically reaching women postpartum has the potential to provide FP information and services to over 90 percent of women of reproductive age in high fertility settings (USAID, 2008). Increased referrals are a positive feature if they signify an increased recognition of life threatening conditions, improved decision making at community and Basic EOC levels, response to removal of barriers (payment schemes, transport mechanisms, waiting homes), or a previous low level of utilization.
Type of indicator	Impact
Unit of measure	Percentage
Method of measurement	There are many types of referral possible, including those from the family directly, or between facilities:<br>1. Self/family referral to: Traditional Birth Attendants/ Healthcare worker training (TBA/CHW), First level skilled health care provider, Basic EOC facility, Comprehensive EOC facility<br>2. TBA/CHW to: First level skilled delivery care provider (SDC), Basic EOC facility, Comprehensive EOC facility<br>3. First level SDC to: Basic EOC facility, Comprehensive EOC facility,<br>4. Comprehensive EOC facility to: Tertiary center
Formula	(Number of women with a potential or actual obstetric complication moved to another site for care/Number of all women with obstetric complications (or deliveries or live births) in the same area and within the same time frame as in the numerator) x 100
Target	To be determined

Frequency	NA
Possible Limitations	▪ NA
Denominator inclusion criteria	▪ Number of all women with obstetric complications (or deliveries or live births) in the same area and within the same time frame as in the numerator
Denominator exclusion criteria	▪ NA
Numerator inclusion criteria	▪ Number of women with a potential or actual obstetric complication moved to another site for care
Numerator exclusion criteria	▪ women with "potential risk factor"
Data sources	▪ Health facility registers<br>▪ Antenatal cards<br>▪ delivery records<br>▪ vehicle log books<br>▪ special referral forms<br>▪ community surveys<br>▪ community data collection systems<br>▪ Case histories

References:

USAID, JSI, Mothercare, Safe Motherhood Indicators— Lessons Learned in Measuring Progresshttps://publications.jsi.com/JSIInternet/Inc/Common/_download_pub.cfm?id=10439&lid=3

For the above indicator, can you provide the below information:

- Can the data elements (numerator and denominator) be collected in the context of Jordan?

X	Yes	Comments:	
	No	Comments:	

- Are the data sources listed above applicable (i.e. information can be extracted from the suggested data source) in the context of Jordan? If no, please provide the source.

	Yes	Comments:	Hospital based reports
	No	Comments:	

- Can the data for the above indicator be segregated for nationals and refugees?

X	Yes	Comments:	could be segregated based on social security number for Jordanians and not Jordanians
	No	Comments:	

- Is the data for Jordan reported in the same way as indicated above? *If you answer "No", please indicate in the comments section how it is being reported. If the indicator is not being reported in Jordan, please insert "Not reported" in the comments section*

	Yes	Comments:	
x	No	Comments:	Total cases

- Is the data for this indicator available for the period extending from January 2019 to December 2021? (if yes, please provide the data)

	Yes	Comments:	
	No	Comments:	

Met need for emergency obstetric and newborn care

Formula

$$\frac{\text{Number of women treated for direct obstetric complications at EmOC facilities, over a defined period}}{\text{Estimated number of women who would have major obstetric complications (or 15\% of expected births), during the same defined period}} \times 100$$

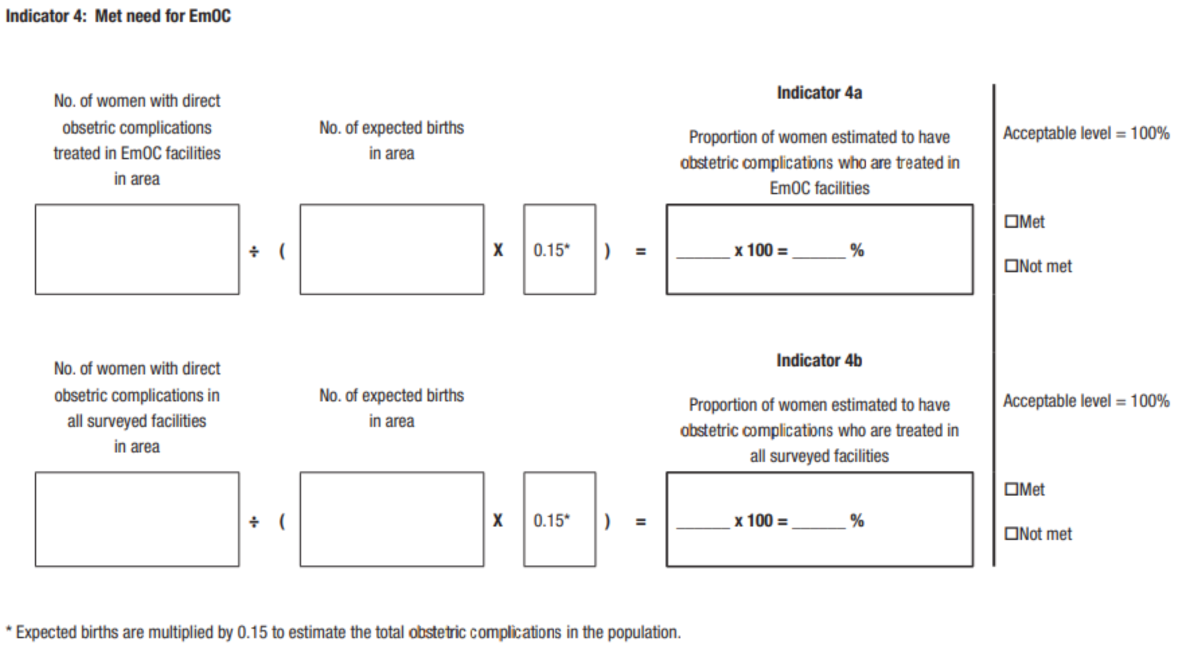
Indicator 4: Met need for EmOC

No. of women with direct obstetric complications treated in EmOC facilities in area ÷ (No. of expected births in area X 0.15*) = Indicator 4a: Proportion of women estimated to have obstetric complications who are treated in EmOC facilities: ______ x 100 = ______ %

Acceptable level = 100%

☐Met

☐Not met

No. of women with direct obsetric complications in all surveyed facilities in area ÷ (No. of expected births in area X 0.15*) = Indicator 4b: Proportion of women estimated to have obstetric complications who are treated in all surveyed facilities: ______ x 100 = ______ %

Acceptable level = 100%

☐Met

☐Not met

* Expected births are multiplied by 0.15 to estimate the total obstetric complications in the population.

Data collection & dissemination:

- NA

Method of measurement:

To calculate met need, information is needed on women in these facilities who are treated for the major obstetric complications. The definitions were derived from WHO (Managing complications in pregnancy and childbirth and Pregnancy, childbirth, postpartum and newborn care) and the International Federation of Gynaecology and Obstetrics Save the Mothers Project. Standardization of definitions can be improved by training and supervision. These definitions are critical for training health workers, enumerators or interviewers who collect such data either routinely or as part of an EmOC needs assessment

Collect Data on Denominator
Denominator: Estimated number of women who would have major obstetric complications (or 15% of expected births), during the same defined period)

1.1 Include

- Number of women with a major obstetric complication includes both women admitted with the complication and women who develop the complication in the facility.

1.2 Exclude

- NA

1.3 Denominator Collection modality
The number of pregnant women who develop obstetric complications requiring medical care to avoid death or disability is estimated to be 15 percent (WHO, 1994a). The number of live births frequently serves as a proxy for all births or pregnancies; when data on the numbers of live births are absent, evaluators can estimate them from total expected births = population x crude birth rate.

2. Collect Data on Numerator
Numerator: Number of women treated for direct obstetric complications at EmOC facilities, over a defined period

2.1 Include
The direct or major obstetric complications include:

- Hemorrhage: antepartum, intrapartum, or postpartum;
- Prolonged/obstructed labor;
- Postpartum sepsis;
- Complications of abortion;
- Severe pre-eclampsia/eclampsia;
- Ectopic pregnancy; and
- Ruptured uterus.

EmOC facilities include both basic and comprehensive levels of essential obstetric care.

2.2 Exclude

- women with “potential risk factor”

3. This indicator will be stratified based on:

- NA

4. Data Sources
4.1 Preferred data Sources:
Facility records (for number of women treated)

4.2 Other data Sources:

5. Calculate:

Divide the Number of women treated for direct obstetric complications at EmOC facilities, over a defined period by Estimated number of women who would have major obstetric complications (or 15% of expected births), during the same defined period. Then multiply the number by 100

6. Possible Limitations:

- Facility record-keeping systems may require adjustments for the routine collection of data on obstetric complications. A useful system will record major complications in the patient register or maternity logbook. Evaluators must ensure that they gather information from all relevant parts of the facility (e.g., gynecology ward, surgical ward, abortion ward, morgue) and not just from the maternity ward. They must also include complications from all EmOC facilities in the area under study in the numerator.
- UNICEF/WHO/UNFPA has set the minimum acceptable level of "met need" as 100 percent, but in most developing country settings, this target is unrealistic. If evaluators find less than 100 percent, they conclude that some women with complications are not receiving the necessary medical care. However, if "met need" is low, researchers should seek other data to determine whether the problem lies in the availability, accessibility, quality of care provided, or other factors, such as cultural factors, that determine the utilization of services.
- Theoretically "met need" can exceed 100 percent, if more than 15 percent of pregnant women in the population develop major obstetric complications. In developed countries, the proportion of women with complications managed in EmOC facilities may be greater than 15 percent of all births. Over-diagnosis of complications, which is seen in parts of Eastern Europe, can also cause this ratio to exceed 100 percent.
- One difficulty with "met need" is that complications are subject to numerous recording biases and, even when standard definitions are in place, results can vary greatly with the data collection system being used and the training of the staff. Furthermore, routine maternity record systems in many countries may not register the "reason for admission" or "maternal complications", although complications can lead to maternal deaths (WHO, 2009).
- "Met need" is also particularly sensitive to the number of abortions included in the numerator. If the incidence of unsafe abortion is high, "met need" is likely to be high. The inclusion of all abortions can cause "met need" to be twice or three times as high as it would be without the abortions. Given this inflation of "met need" as a result of the inclusion of all abortion complications, a growing number of advocates for the indicator calculate it both ways, with and without all abortions. By excluding postabortion complications, estimates may be more comparable.
- The appropriateness of using 15 percent of all births/ pregnancies to estimate the number of women who experience obstetric complications is also open to discussion. WHO‘s estimates of births with complications may be higher than 15 percent: hemorrhage, 10 percent of pregnancies; sepsis, 8 percent; hypertensive disorders of pregnancy, 5 percent; obstructed labor, 5 percent (WHO, 1996a). However, prospective data from West Africa suggest that 6 percent more reasonably estimates severe obstetric complications (Prual, 2000). The narrower the definition of what is considered a direct or major obstetric complication, the more reliable and comparable the estimates will be (MotherCare, 2000a). However, birth records and registries will likely lack sufficient detail on complications to allow much refinement regarding the severity of a complication.
- The issue of double-counting a woman in the numerator (one who is admitted to the same facility more than once during her pregnancy or postpartum period or one who is admitted to more than one facility) is unlikely to seriously bias the results. If this situation were to occur, it would bias the indicators by presenting a more positive view of the health system than merited.

- Given that the crude birth rate (CBR), the total population, and 15 percent are all estimates and that the accuracy of the CBR and population may vary according to the source, "met need" will likely be imprecise and may over- or underestimate the true value. To make the indicator useful for comparisons across facilities and districts or over time, one must use the same definitions and document the criteria used in each definition.

Data Collection Sheet

The following table contains detailed information and support from the literature on the measurement of the indicator.

Indicator	Met need for emergency obstetric and newborn care
Alternate name	Met need for EmOC
Definition	The percent of all women with major direct obstetric complications who are treated in a health facility providing emergency obstetric care (EmOC) in a given reference period.<br><br>Met need should be calculated at all health facilities as well as at EmOC facilities to provide a more complete picture of the use of the health system and where women are being treated (WHO, 2009).
Rationale	The purpose of this indicator is to gauge the level of use of EmOC services by women experiencing a major obstetric complication in a specified time period and geographical area. Met need is a more refined measure of the use of EmOC than Proportion of all births in EmOC facilities.
Type of indicator	For more detailed monitoring of abortion complications, we recommend a set of 'process indicators for safe abortion', which include 11 signal functions that define basic and comprehensive care. Like the EmOC indicators, the safe abortion indicators measure the availability, distribution, use and quality of safe abortion services
Unit of measure	Percentage
Method of measurement	To calculate met need, information is needed on women in these facilities who are treated for the major obstetric complications. The definitions were derived from WHO (Managing complications in pregnancy and childbirth and Pregnancy, childbirth, postpartum and newborn care) and the International Federation of Gynaecology and Obstetrics Save the Mothers Project. Standardization of definitions can be improved by training and supervision. These definitions are critical for training health workers, enumerators or interviewers who collect such data either routinely or as part of an EmOC needs assessment
Formula	(Number of women treated for direct obstetric complications at EmOC facilities, over a defined period / Estimated number of women who would have major obstetric complications (or 15% of expected births), during the same defined period) x 100
Target	As the goal is that all women who have obstetric complications will receive EmOC, the minimum acceptable level is 100%.
Frequency	NA
Possible Limitations	▪ Facility record-keeping systems may require adjustments for the routine collection of data on obstetric complications. A useful system will record major complications in the patient register or maternity logbook. Evaluators must ensure that they gather information from all relevant parts of the facility (e.g., gynecology ward, surgical ward, abortion ward, morgue) and not just from the maternity ward. They must also include complications from all EmOC facilities in the area under study in the numerator.<br>▪ UNICEF/WHO/UNFPA has set the minimum acceptable level of "met need" as 100 percent, but in most developing country settings,

	this target is unrealistic. If evaluators find less than 100 percent, they conclude that some women with complications are not receiving the necessary medical care. However, if "met need" is low, researchers should seek other data to determine whether the problem lies in the availability, accessibility, quality of care provided, or other factors, such as cultural factors, that determine the utilization of services.<br>▪ Theoretically "met need" can exceed 100 percent, if more than 15 percent of pregnant women in the population develop major obstetric complications. In developed countries, the proportion of women with complications managed in EmOC facilities may be greater than 15 percent of all births. Over-diagnosis of complications, which is seen in parts of Eastern Europe, can also cause this ratio to exceed 100 percent.<br>▪ One difficulty with "met need" is that complications are subject to numerous recording biases and, even when standard definitions are in place, results can vary greatly with the data collection system being used and the training of the staff. Furthermore, routine maternity record systems in many countries may not register the "reason for admission" or "maternal complications", although complications can lead to maternal deaths (WHO, 2009).<br>▪ "Met need" is also particularly sensitive to the number of abortions included in the numerator. If the incidence of unsafe abortion is high, "met need" is likely to be high. The inclusion of all abortions can cause "met need" to be twice or three times as high as it would be without the abortions. Given this inflation of "met need" as a result of the inclusion of all abortion complications, a growing number of advocates for the indicator calculate it both ways, with and without all abortions. By excluding postabortion complications, estimates may be more comparable.<br>▪ The appropriateness of using 15 percent of all births/ pregnancies to estimate the number of women who experience obstetric complications is also open to discussion. WHO‘s estimates of births with complications may be higher than 15 percent: hemorrhage, 10 percent of pregnancies; sepsis, 8 percent; hypertensive disorders of pregnancy, 5 percent; obstructed labor, 5 percent (WHO, 1996a). However, prospective data from West Africa suggest that 6 percent more reasonably estimates severe obstetric complications (Prual, 2000). The narrower the definition of what is considered a direct or major obstetric complication, the more reliable and comparable the estimates will be (MotherCare, 2000a). However, birth records and registries will likely lack sufficient detail on complications to allow much refinement regarding the severity of a complication.<br>▪ The issue of double-counting a woman in the numerator (one who is admitted to the same facility more than once during her pregnancy or postpartum period or one who is admitted to more than one facility) is unlikely to seriously bias the results. If this situation were to occur, it would bias the indicators by presenting a more positive view of the health system than merited.<br>▪ Given that the crude birth rate (CBR), the total population, and 15 percent are all estimates and that the accuracy of the CBR and population may vary according to the source, "met need" will likely

	be imprecise and may over- or underestimate the true value. To make the indicator useful for comparisons across facilities and districts or over time, one must use the same definitions and document the criteria used in each definition.
Denominator inclusion criteria	Estimated number of women who would have major obstetric complications (or 15% of expected births), during the same defined period)<br><br>Number of women with a major obstetric complication includes both women admitted with the complication and women who develop the complication in the facility.
Denominator exclusion criteria	NA
Numerator inclusion criteria	Number of women treated for direct obstetric complications at EmOC facilities, over a defined period<br>The direct or major obstetric complications include:<br>▪ Hemorrhage: antepartum, intrapartum, or postpartum;<br>▪ Prolonged/obstructed labor;<br>▪ Postpartum sepsis;<br>▪ Complications of abortion;<br>▪ Severe pre-eclampsia/eclampsia;<br>▪ Ectopic pregnancy; and<br>▪ Ruptured uterus.<br><br>EmOC facilities include both basic and comprehensive levels of essential obstetric care
Numerator exclusion criteria	NA
Data sources	Facility records (for number of women treated)

References:

USAID, Met need for EmOC, https://www.measureevaluation.org/prh/rh_indicators/womens-health/sm/met-need-for-emoc

UNICEF, WHO and UNFPA, 1997, Monitoring emergency obstetric care: a handbook. https://www.unfpa.org/sites/default/files/pub-pdf/obstetric_monitoring.pdf

9789241547734_eng.pdf;jsessionid=DCD36F52433F86410933E83A0F2AF3B5 (who.int)

For the above indicator, can you provide the below information:

- Can the data elements (numerator and denominator) be collected in the context of Jordan?

[X] Yes Comments:

[ ] No Comments:

- Are the data sources listed above applicable (i.e. information can be extracted from the suggested data source) in the context of Jordan? If no, please provide the source.

[ ] Yes Comments: Hospital based reports

[ ] No Comments:

- Can the data for the above indicator be segregated for nationals and refugees?

[X] Yes Comments: could be segregated based on social security number for Jordanians and not Jordanians

[ ] No Comments:

- Is the data for Jordan reported in the same way as indicated above? *If you answer "No", please indicate in the comments section how it is being reported. If the indicator is not being reported in Jordan, please insert "Not reported" in the comments section*

[ ] Yes Comments:

[ ] No Comments: Not reported

- Is the data for this indicator available for the period extending from January 2019 to December 2021? (if yes, please provide the data)

[ ] Yes Comments:

[ ] No Comments:

Demand for family planning satisfied - use of modern methods (%)

Formula

$$\frac{\text{Number of women aged 15–49 that are fecund and are married or in union and need contraception, who are currently using any modern method of contraception}}{\text{Total number of women aged 15-49 that are fecund and are married / have a partner and need contraception}} \times 100$$

Data collection & dissemination:

- Frequency of collection: Every 3–5 years

Method of measurement:

Data are derived from re-analysis of Demographic and Health Surveys (DHS), Multiple Indicator Cluster Surveys (MICS) and Reproductive Health Surveys (RHS) micro-data which are publicly available using the standard indicator definitions as published in DHS, MICS or RHS documentation. The analysis was done by the WHO Collaborating Center for Health Equity Monitoring (International Center for Equity in Health, Federal University of Pelotas, Brazil).

Collect Data on Denominator

Denominator: Total number of women aged 15-49 that are fecund and are married / have a partner and need contraception.

1.1 Include

A woman is considered to have a demand for family planning if:

- She or her partner is currently using a contraceptive method.; or,
- She has an unmet need for family planning:
- Women who are currently pregnant or postpartum amenorrhoeic whose current pregnancy or last birth was unwanted or mistimed, or,
- Women who are currently married or sexually active and able to become pregnant who say that they want to delay pregnancy by two or more years or do not know when or whether they want any more children and who are not currently using any contraceptive method.

The denominator includes women who are not using any contraceptive method as well as those who are using a modern or a traditional contraceptive method.

1.2 Exclude

- NA

1.3 Denominator Collection modality

2. Collect Data on Numerator

Numerator: Number of women aged 15–49 that are fecund and are married or in union and need contraception, who are currently using any modern method of contraception.

2.1 Include

The numerator includes all women 15–49 years old who were using modern contraceptive methods at the time of the survey. The following are considered modern contraceptive methods:

- The pill (oral contraceptives)
- Intrauterine device (IUD)
- Injectables
- Female sterilization
- Male sterilization
- Female condoms
- Male condoms
- Implants
- Emergency contraception
- Standard days method
- Lactational amenorrhoea method (LAM)
- The diaphragm
- Foam or jelly.

2.2 Exclude

3. This indicator will be stratified based on:

- Age (15–19, 20–24, 25–49 and 15–49 years)
- Economic status
- Education
- Place of residence
- Subnational region

4. Data Sources

4.1 Preferred data Sources:

NA

4.2 Other data Sources:

NA

5. Calculate:

Divide the Number of women aged 15–49 that are fecund and are married or in union and need contraception, who are currently using any modern method of contraception by Total number of women aged 15-49 that are fecund and are married / have a partner and need contraception. Then multiply the number by 100

6. Possible Limitations:

This indicator measures access to more effective methods of contraception, which will lead to fewer unwanted pregnancies and improved maternal and child health.

Construction of this indicator requires complex calculations. The consistent application of a standard definition can provide measures of demand for family planning satisfied by modern methods that are comparable over time and across countries.

Data Collection Sheet

The following table contains detailed information and support from the literature on the measurement of the indicator.

Indicator	Demand for family planning satisfied - use of modern methods (%)
Alternate name	Percentage of women of reproductive age (15–49 years old) who have their demand for family planning satisfied with modern methods<br>OR<br>Demand for family planning satisfied with modern methods
Definition	Percentage of women aged 15-49 years, married or in union, who are currently using any modern method of contraception, among those in need of contraception. Women in need of contraception include women who are fecund but report wanting to space their next birth or stop childbearing altogether as well as women with a mistimed or unwanted pregnancy. Modern methods of contraception include: oral contraceptive pills, implants, injectables, contraceptive patch and vaginal ring, intrauterine device (IDU), female and male condoms, female and male sterilization, vaginal barrier methods (including the diaphragm, cervical cap and spermicidal agents), lactational amenorrhea method (LAM), emergency contraception pills, standard days method (SDM), basal body temperature (BBT) method, TwoDay method and sympto-thermal method.
Rationale	This indicator assesses progress towards increasing the capacity of women and adolescent girls to access sexual and reproductive health services and being able to exercise their right to control and freely decide on matters related to their sexuality and sexual and reproductive health. It reflects the right of women and adolescent girls to decide whether and when to have children and having the methods to implement this decision.<br>This indicator is also used to measure progress towards Sustainable Development Goals target 3.7, which aims to ensure universal access to sexual and reproductive health-care services, including for family planning, information and education, and integrating reproductive health into national strategies and programmes by 2030.<br>Sexual and reproductive health services are also an entry point for HIV prevention, treatment, care and support services, and their integration will be key to ensuring the sustainability of HIV-related services.
Type of indicator	NA
Unit of measure	Percentage
Method of measurement	Data are derived from re-analysis of Demographic and Health Surveys (DHS), Multiple Indicator Cluster Surveys (MICS) and Reproductive Health Surveys (RHS) micro-data which are publicly available using the standard indicator definitions as published in DHS, MICS or RHS documentation. The analysis was done by the WHO Collaborating Center for Health Equity Monitoring (International Center for Equity in Health, Federal University of Pelotas, Brazil).
Formula	[Number of women aged 15–49 that are fecund and are married or in union and need contraception, who are currently using any modern method of contraception / Total number of women aged 15-49 that are fecund and are married / have a partner and need contraception] X 100
Target	NA

Frequency	Frequency of collection: Every 3–5 years
Possible Limitations	This indicator measures access to more effective methods of contraception, which will lead to fewer unwanted pregnancies and improved maternal and child health.<br><br>Construction of this indicator requires complex calculations. The consistent application of a standard definition can provide measures of demand for family planning satisfied by modern methods that are comparable over time and across countries.
Denominator inclusion criteria	Total number of women aged 15-49 that are fecund and are married / have a partner and need contraception.
Denominator exclusion criteria	NA
Numerator inclusion criteria	Number of women aged 15–49 that are fecund and are married or in union and need contraception, who are currently using any modern method of contraception.
Numerator exclusion criteria	NA
Data sources	NA
Indicator responsibility	WHO

References:

WHO, Global Health Observatory, Demand for family planning satisfied - use of modern methods (%)
https://www.who.int/data/gho/indicator-metadata-registry/imr-details/3813

UNAIDS, Demand for family planning satisfied by modern methods
https://indicatorregistry.unaids.org/indicator/demand-family-planning-satisfied-modern-methods

For the above indicator, can you provide the below information: NA

- Can the data elements (numerator and denominator) be collected in the context of Jordan?

☐ Yes Comments:

☐ No Comments:

- Are the data sources listed above applicable (i.e. information can be extracted from the suggested data source) in the context of Jordan? If no, please provide the source.

☐ Yes Comments:

☐ No Comments:

- Can the data for the above indicator be segregated for nationals and refugees?

☐ Yes Comments:

☐ No Comments:

- Is the data for Jordan reported in the same way as indicated above? *If you answer "No", please indicate in the comments section how it is being reported. If the indicator is not being reported in Jordan, please insert "Not reported" in the comments section*

☐ Yes Comments:

☐ No Comments:

- Is the data for this indicator available for the period extending from January 2019 to December 2021? (if yes, please provide the data)

☐ Yes Comments:

☐ No Comments:

Antenatal care coverage

Definition:

Percentage of women who utilized antenatal care provided by skilled health personnel for reasons related to pregnancy at least once during pregnancy as a percentage of live births in a given time period

FormulaThe number of women aged 15-49 with a live birth in a given time period that received antenatal care provided by skilled health personnel (doctors, nurses or midwives) at least once during pregnancy*100/ Total number of women aged 15-49 with a live birth in the same period

---

The same formula is used for calculating 4+ visits and 8+ visits

Data collection & dissemination:

- Collect information on this indicator every 2 or 5 years.
- Dissemination of information on annual basis.

Method of measurement:

1. Collect Data on Denominator
Denominator:

Total number of women aged 15-49 with a live birth in the same period

1.1 Include
The indicator refers to women who had a live birth in a recent time period, generally two years for MICS and five years for DHS.

1.2 Exclude
- NA

1.3 Denominator Collection modality

2. Collect Data on Numerator
Numerator: The number of women aged 15-49 with a live birth in a given time period that received antenatal care provided by skilled health personnel (doctors, nurses or midwives) at least once during pregnancy

2.1 Include
The indicator refers to women who had a live birth in a recent time period, generally two years for MICS and five years for DHS.

2.2 Exclude

- NA

3. This indicator will be stratified based on:

- Geographical area
- Population groups (socio-economic groups)
- Level of facility
- location of facility (e.g. urban, rural)
- Type of health personnel
- Timing of ANC visit

4. Data Sources

4.1 Preferred data Sources:

- MICS, DHS and other national surveys
- Household surveys

4.2 Other data Sources:

- Facility reporting system

6. Possible Limitations:

- Receiving antenatal care during pregnancy does not guarantee the receipt of interventions that are effective in improving maternal health
- standardization of the definition of skilled health personnel is sometimes difficult because of differences in training of health personnel in different countries.
- Recall error is a potential source of bias in the data.
- The respondent may or may not know or remember the qualifications of the person providing antenatal care.

Data Collection Sheet

The following table contains detailed information and support from the literature on the measurement of the indicator.

Indicator	Antenatal care coverage
Alternate name	
Definition	Antenatal care coverage (at least one visit) is the percentage of women aged 15 to 49 with a live birth in a given time period that received antenatal care provided by skilled health personnel (doctor, nurse or midwife) at least once during pregnancy.<br>Skilled health personnel refers to workers/attendants that are accredited health professionals – such as a midwife, doctor or nurse – who have been educated and trained to proficiency in the skills needed to manage normal (uncomplicated) pregnancies, childbirth and the immediate postnatal period, and in the identification, management and referral of complications in women and newborns. Both trained and untrained traditional birth attendants are excluded.
Rationale	Antenatal visits present opportunities for reaching pregnant women with interventions that may be vital to their health and well-being and that of their infants.<br><br>WHO guidelines are specific on the content of antenatal care visits, which should include:<br>blood pressure measurement<br>urine testing for bacteriuria and proteinuria<br>blood testing to detect syphilis and severe anaemia<br>weight/height measurement (optional).
Type of indicator	Outcome
Unit of measure	Percentage
Method of measurement	
Formula	The number of women aged 15-49 with a live birth in a given time period that received antenatal care provided by skilled health personnel (doctors, nurses or midwives) at least once during pregnancy*100/ Total number of women aged 15-49 with a live birth in the same period<br><br><u>The same formula can be sued to calculate 4+ and 8+ visits</u>
Target	The indicator values range from 0 to 100, with 100 being the ideal situation in which all pregnant women between 15 and 49 years have seen a doctor at least once—or four times—during their pregnancy. For ANC 1+, indicator values generally fall between 50 and 100 per cent. For ANC 4+ and ANC 8+, values tend to be lower, often substantially.
Frequency	▪ Collect information on this indicator every 2 or 5 years.<br>▪ Dissemination of information on annual basis.
Possible Limitations	▪ Receiving antenatal care during pregnancy does not guarantee the receipt of interventions that are effective in improving maternal health

	▪ standardization of the definition of skilled health personnel is sometimes difficult because of differences in training of health personnel in different countries.<br>▪ Recall error is a potential source of bias in the data.<br>▪ The respondent may or may not know or remember the qualifications of the person providing antenatal care.
Denominator inclusion criteria	Total number of women aged 15-49 with a live birth in the same period<br><br>The indicator refers to women who had a live birth in a recent time period, generally two years for MICS and five years for DHS.
Denominator exclusion criteria	NA
Numerator inclusion criteria	The Number of women aged 15-49 with a live birth in a given time period that received antenatal care provided by skilled health personnel (doctors, nurses or midwives) at least once during pregnancy<br><br>The indicator refers to women who had a live birth in a recent time period, generally two years for MICS and five years for DHS.
Numerator exclusion criteria	NA
Data sources	▪ MICS, DHS and other national surveys<br>▪ Household surveys<br>▪ Facility reporting system

References:

UNICEF, Antenatal Care, https://data.unicef.org/topic/maternal-health/antenatal-care/#:~:text=Definition%20of%20indicators,at%20least%20once%20during%20pregnancy.

United Nations, Indicators for Monitoring the Millennium Development goals, 5.5 Antenatal care coverage (at least one visit and at least four visits) http://mdgs.un.org/unsd/mi/wiki/5-5-Antenatal-care-coverage-at-least-one-visit-and-at-least-four-visits.ashx

Monitor Indicator sheet Antenatal care eigth visits.pdf (srhr.org)

RHIndicatorsPVL.indd (who.int)

whostat2006definitionsandmetadata.doc

For the above indicator, can you provide the below information:

- Can the data elements (numerator and denominator) be collected in the context of Jordan?

[X] Yes Comments:

☐ No Comments:

- Are the data sources listed above applicable (i.e. information can be extracted from the suggested data source) in the context of Jordan? If no, please provide the source.

☐ Yes Comments: Hospital based reports

☐ No Comments:

- Can the data for the above indicator be segregated for nationals and refugees?

X Yes Comments: could be segregated based on social security number for Jordanians and not Jordanians

☐ No Comments:

- Is the data for Jordan reported in the same way as indicated above? *If you answer "No", please indicate in the comments section how it is being reported. If the indicator is not being reported in Jordan, please insert "Not reported" in the comments section*

☐ Yes Comments:

x No Comments: Total cases

- Is the data for this indicator available for the period extending from January 2019 to December 2021? (if yes, please provide the data)

☐ Yes Comments:

☐ No Comments:

Percent of pregnant women who receive the recommended number of iron/folate supplements during pregnancy

Formula

$$\frac{\text{Number of pregnant women who received or purchased the recommended number of iron/folic acid tablets during last pregnancy}}{\text{Total number of pregnant women with a birth in last two years}} \times 100$$

Data collection & dissemination:

- Collect information on this indicator is continuous, or every 3-5 years.
- Dissemination of information every 2–5 years.

Method of measurement:

- NA

1. Collect Data on Denominator
Denominator: Total number of pregnant women with a birth in last two years

1.1 Include
- Total number of pregnant women with a birth in last two years

1.2 Exclude
- NA

1.3 Denominator Collection modality

2. Collect Data on Numerator
Numerator: Number of pregnant women who received or purchased the recommended number of iron/folic acid tablets during last pregnancy

2.1 Include
Number of pregnant women who received or purchased the recommended number of iron/folic acid tablets during last pregnancy

2.2 Exclude
- NA

3. This indicator will be stratified based on:

- location (near/ far from the nearest health facility; rural/ urban areas)
- source of supplement (purchased in a shop/ received in a health facility)
- mother's age
- number of children
- maternal education
- socioeconomic status

4. Data Sources

4.1 Preferred data Sources:

- Health facility and antenatal care (ANC) clinic records
- Population based surveys (e.g., DHS, RHS, UNICEF Multiple Indicator Cluster Surveys)

4.2 Other data Sources:

5. Calculate:

Divide the Number of pregnant women who received or purchased the recommended number of iron/folic acid tablets during last pregnancy by the Total number of pregnant women with a birth in last two years. Then multiply by 100

6. Possible Limitations:

- This indicator captures the distribution of iron/folate supplements, but not the actual consumption.
- This indicator is primarily intended to measure supplementation during the last two trimesters of pregnancy.
- Accurate reporting of the numbers of supplements received or purchased by women is problematic, even when measured specifically for the second and third trimesters of pregnancy.
- Heath facility client records may not be consistently accurate.
- Some women may be purchasing supplements from community-based pharmacies and other sources and their recall for amounts purchased may be subject to error.
- This indicator assumes that the mothers will remember for up to two and half years the information your survey is asking about. Since this is not very likely, IndiKit recommends using for this indicator a shorter recall period by interviewing mothers of children aged 0-11.99 months. Use it only if the data is supposed to be used purely for the purpose of your intervention (i.e. making programming decisions, measuring its results, etc.) and does not need to be comparable with the statistics of other stakeholders which use longer recall period.

Data Collection Sheet

The following table contains detailed information and support from the literature on the measurement of the indicator.

Indicator	Percent of pregnant women who receive the recommended number of iron/folate supplements during pregnancy
Alternate name	Proportion of pregnant women receiving iron and folic acid supplements<br>OR<br>% of mothers of children aged 0-11 months who consumed the recommended number of iron/ folic acid supplements during pregnancy
Definition	The percent of women with a birth in the last two years who received or bought iron/folic acid supplements for at least six months during their last pregnancy in amounts that were in accordance with recommended protocols.
Rationale	This indicator measures the percentage of women who received or purchased the recommended amounts of supplements for iron and folic acid during pregnancy. It provides information about the quality of ANC services and/or women's access to purchasing supplements through local pharmacies and community-based sources.<br><br>Iron deficiency is a common nutrient deficiency and the resulting iron deficiency anemia is a major contributor to the global burden of disease (WHO/CDC, 2008). Anemia is a common problem among women of reproductive age, especially in low and middle income countries where low dietary intake of bioavailable iron combined with endemic infectious diseases such as helminthiasis puts women at increased risk in the preconception period. Low preconception hemoglobin and ferritin levels increase the risk of poor fetal growth and low birth weight (Dean, Lassi, Imam and Bhutta, 2014). Anemia during pregnancy (hemoglobin levels < 11g/dl) is associated with increased risks for maternal mortality, premature birth, and low birth weight. Pregnant women need iron to support their enlarged blood volume, to provide for placental and fetal needs,and to replace blood loss in childbirth. The fetus relies on maternal iron stores to create adequate reserves of its own, which in tandem with the iron in breast milk, will meet the iron needs of the normal birth weight infant through the first six months of life. The 2008 Copenhagen Consensus panel ranked the provision of micronutrients, including iron and folic acid, as the world's best investment for development (Micronutrient Initiative [MI], 2009). This indicator relates to three of the Millennium Development Goals: #1. Reduce poverty and hunger; #4. Reduce child mortality; and #5. Improve maternal health.<br><br>Iron supplementation is universally recommended during the second and third trimesters when iron stores become depleted over the course of pregnancy (WHO, 2010; MI, 2009). The high physiologic requirement for iron during pregnancy is difficult to meet with most diets even where more iron-rich foods are available (INACG/WHO/UNICEF, 1998). Providing iron/folate supplements for women pre-pregnancy or early in the pregnancy is desirable, particularly where deficiency levels are high (≥40%). Because the efficiency of absorption of iron increases as iron deficiency anemia becomes more severe, the recommended 60 mg dose should provide adequate supplemental

	iron to women who do not have clinically severe anemia if it is given for an adequate duration.<br><br>Guidelines for treating severe anemia in pregnant women (Hg <7g/dl) includes three months of therapeutic supplementation (120 mg iron and 400 ug folic acid) followed by the preventive regimen (60 mg iron and 400 ug folic acid) for the duration of pregnancy and three months postpartum (INACG/WHO/UNICEF, 1998). Complementary parasite control measures and dietary counseling are also recommended.<br><br>Supplementation with 400 µg of folic acid around the time of conception significantly reduces the incidence of neural tube defects. These defects cause serious disabilities and infant mortality, and commonly arise in the first weeks of pregnancy before a woman may realize she is pregnant. Folate supplementation begun after the first trimester of pregnancy is too late to prevent birth defects. A daily dose of 400 µg folic acid is considered a safe and healthy intake for women during pregnancy and lactation but is more than the amount required to produce an optimal hemoglobin response in pregnant women.<br><br>An alternative indicator that reflects the adequacy of the program in meeting the needs of specific clients is 'Number of iron/folate tablets distributed per eligible client.'
Type of indicator	Outcome/Output
Unit of measure	Percentage
Method of measurement	NA
Formula	(Number of pregnant women who received or purchased the recommended number of iron/folic acid tablets during last pregnancy/ Total number of pregnant women with a birth in last two years) x 100
Target	The current WHO recommendations are 60 mg of iron and 400 ug folic acid daily during pregnancy for all women, beginning as soon as possible during gestation and no later than the third month<br><br>A coverage of less than 80%<br><br>(different references)
Frequency	▪ Collect information on this indicator is continuous, or every 3-5 years.<br>▪ Dissemination of information every 2–5 years.
Possible Limitations	▪ This indicator captures the distribution of iron/folate supplements, but not the actual consumption.<br>▪ This indicator is primarily intended to measure supplementation during the last two trimesters of pregnancy.<br>▪ Accurate reporting of the numbers of supplements received or purchased by women is problematic, even when measured specifically for the second and third trimesters of pregnancy.<br>▪ Heath facility client records may not be consistently accurate.

	▪ Some women may be purchasing supplements from community-based pharmacies and other sources and their recall for amounts purchased may be subject to error.<br>▪ This indicator assumes that the mothers will remember for up to two and half years the information your survey is asking about. Since this is not very likely, IndiKit recommends using for this indicator a shorter recall period by interviewing mothers of children aged 0-11.99 months. Use it only if the data is supposed to be used purely for the purpose of your intervention (i.e. making programming decisions, measuring its results, etc.) and does not need to be comparable with the statistics of other stakeholders which use longer recall period.
Denominator inclusion criteria	Total number of pregnant women with a birth in last two years
Denominator exclusion criteria	NA
Numerator inclusion criteria	Number of pregnant women who received or purchased the recommended number of iron/folic acid tablets during last pregnancy
Numerator exclusion criteria	NA
Data sources	▪ Health facility and antenatal care (ANC) clinic records<br>▪ Population based surveys (e.g., DHS, RHS, UNICEF Multiple Indicator Cluster Surveys)

References:

IndiKit, Maternal Iron/ Folic Acid Supplement Coverage https://www.indikit.net/indicator/7-maternal-and-child-health/244-maternal-iron-folic-acid-supplement-coverage

WHO, UNICEF, Developing and Validating an iron and folic acid supplementation indicator for tracking progress towards Global Nutrition Monitoring Framework Targets, Final Report, June 2018 https://apps.who.int/iris/rest/bitstreams/1150724/retrieve

USAID, Percent of pregnant women who receive the recommended number of iron/folate supplements during pregnancy, https://www.measureevaluation.org/prh/rh_indicators/womens-health/womens-nutrition/percent-of-pregnant-women-who-receive-the#:~:text=The%20current%20WHO%20recommendations%20are,2010%3B%20WHO%2C%202011).

For the above indicator, can you provide the below information:

- Can the data elements (numerator and denominator) be collected in the context of Jordan?

[X] Yes Comments:

[ ] No Comments:

- Are the data sources listed above applicable (i.e. information can be extracted from the suggested data source) in the context of Jordan? If no, please provide the source.

[ ] Yes Comments: Hospital based reports

[ ] No Comments:

- Can the data for the above indicator be segregated for nationals and refugees?

[X] Yes Comments: could be segregated based on social security number for Jordanians and not Jordanians

[ ] No Comments:

- Is the data for Jordan reported in the same way as indicated above? *If you answer "No", please indicate in the comments section how it is being reported. If the indicator is not being reported in Jordan, please insert "Not reported" in the comments section*

[ ] Yes Comments:

[X] No Comments: Total cases

- Is the data for this indicator available for the period extending from January 2019 to December 2021? (if yes, please provide the data)

[ ] Yes Comments:

[ ] No Comments:

Percentage of babies weighed at birth

Formula

$$\frac{\text{Number of children under age of five whose birth weight are reported as being registered with the relevant national civil authorities}}{\text{The total number of children under the age of five in the population}} \times 100$$

Data collection & dissemination:

- Collect information on this indicator on annual basis.

Method of measurement:

Estimates of coverage are taken from two sources: 1. United Nations demographic yearbook Only those with the code "C" (Civil registration, estimated over 90% complete) are reported in the World Health Statistics. 2. UNICEF's State of the World's Children Estimates refer to the percentage of children less than five years old who were registered at the moment of the survey. The numerator of this indicator includes children whose birth certificate was seen by the interviewer or whose mother or caretaker says the birth has been registered. MICS data refer to children alive at the time of the survey.

Collect Data on Denominator
Denominator: the total number of children under the age of five in the population

1.1 Include
- NA

1.2 Exclude
- NA

1.3 Denominator Collection modality

2. Collect Data on Numerator
Numerator: Number of children under age of five whose births are reported as being registered with the relevant national civil authorities

2.1 Include
The numerator of this indicator includes children whose birth certificate was seen by the interviewer or whose mother or caretaker says the birth has been registered

2.2 Exclude
- NA

3. This indicator will be stratified based on:

- Sex
- Age
- Income
- Place of residence
- Geographic location

4. Data Sources

4.1 Preferred data Sources:

- Censuses, household surveys such as MICS and DHS
- National civil registration systems
- Civil registration systems
- Household or other population-based surveys

4.2 Other data Sources:

- MICS questionnaire

5. Calculate:

Number of children under age of five whose births are reported as being registered with the relevant national civil authorities divided by the total number of children under the age of five in the population multiplied by 100

6. Possible Limitations:

- In the absence of reliable administrative data, household surveys have become a key source of data to monitor levels and trends in birth registration. In most low- and middle-income countries, such surveys represent the sole source of this information.
- Data from household surveys like MICS or DHS sometimes refer only to children with a birth certificate.
- UNICEF methodically notes this difference when publishing country-level estimates for global SDG monitoring.
- Nationally produced data are not adjusted or recalculated.

Data Collection Sheet

The following table contains detailed information and support from the literature on the measurement of the indicator.

Indicator	% of babies weighed at birth
Alternate name	Proportion of children under 5 years of age whose births have been registered with a civil authority.<br>OR<br>Civil registration coverage of births (%)
Definition	Proportion of children under 5 years of age whose births have been registered with a civil authority.
Rationale	Registering children at birth is the first step in securing their recognition before the law, safeguarding their rights, and ensuring that any violation of these rights does not go unnoticed. Children without official identification documents may be denied health care or education. Later in life, the lack of such documentation can mean that a child may enter into marriage or the labour market, or be conscripted into the armed forces, before the legal age. In adulthood, birth certificates may be required to obtain social assistance or a job in the formal sector, to buy or prove the right to inherit property, to vote and to obtain a passport. Children's right to a name and nationality is enshrined in the Convention on the Rights of the Child (CRC) under Article 7
Type of indicator	INPUT
Unit of measure	Percent
Method of measurement	Estimates of coverage are taken from two sources: 1. United Nations demographic yearbook Only those with the code "C" (Civil registration, estimated over 90% complete) are reported in the World Health Statistics. 2. UNICEF's State of the World's Children Estimates refer to the percentage of children less than five years old who were registered at the moment of the survey. The numerator of this indicator includes children whose birth certificate was seen by the interviewer or whose mother or caretaker says the birth has been registered. MICS data refer to children alive at the time of the survey.
Formula	[Number of children under age of five whose births are reported as being registered with the relevant national civil authorities] / [the total number of children under the age of five in the population] X 100
Target	NA
Frequency	▪ Collect information on this indicator on annual basis.
Possible Limitations	▪ In the absence of reliable administrative data, household surveys have become a key source of data to monitor levels and trends in birth registration. In most low- and middle-income countries, such surveys represent the sole source of this information.<br>▪ Data from household surveys like MICS or DHS sometimes refer only to children with a birth certificate.<br>▪ UNICEF methodically notes this difference when publishing country-level estimates for global SDG monitoring.<br>▪ Nationally produced data are not adjusted or recalculated.
Denominator inclusion criteria	the total number of children under the age of five in the population

Denominator exclusion criteria	NA
Numerator inclusion criteria	Number of children under age of five whose births are reported as being registered with the relevant national civil authorities<br><br>The numerator of this indicator includes children whose birth certificate was seen by the interviewer or whose mother or caretaker says the birth has been registered
Numerator exclusion criteria	NA
Data sources	▪ Censuses, household surveys such as MICS and DHS<br>▪ National civil registration systems<br>▪ Civil registration systems<br>▪ Household or other population-based surveys<br>▪ MICS questionnaire

References:

UNICEF, Indicator 16.9.1: Proportion of children under 5 years of age whose births have been registered with a civil authority, by age, https://www.unescwa.org/sites/www.unescwa.org/files/u1461/metadata-16-09-01.pdf

WHO, Data Portal, MATERNAL, NEWBORN, CHILD AND ADOLESCENT HEALTH AND AGEING https://www.who.int/data/maternal-newborn-child-adolescent-ageing/indicator-explorer-new/mca/proportion-of-children-under-5-years-of-age-whose-births-are-registered

For the above indicator, can you provide the below information:

- Can the data elements (numerator and denominator) be collected in the context of Jordan?

[X] Yes Comments:

[ ] No Comments:

- Are the data sources listed above applicable (i.e. information can be extracted from the suggested data source) in the context of Jordan? If no, please provide the source.

[ ] Yes Comments: Hospital based reports

[ ] No Comments:

- Can the data for the above indicator be segregated for nationals and refugees?

[X] Yes Comments: could be segregated based on social security number for Jordanians and not Jordanians

[ ] No Comments:

- Is the data for Jordan reported in the same way as indicated above? *If you answer "No", please indicate in the comments section how it is being reported. If the indicator is not being reported in Jordan, please insert "Not reported" in the comments section*

[ ] Yes Comments:

[x] No Comments: Total cases

- Is the data for this indicator available for the period extending from January 2019 to December 2021? (if yes, please provide the data)

[ ] Yes Comments:

[ ] No Comments:

Percentage of newborns receiving essential newborn care

Definition:

Percent of newborns who received all four elements of essential newborn care:

- immediate and thorough drying,
- immediate skin-to-skin contact,
- delayed cord clamping, and
- initiation of breastfeeding in the first hour

Formula

$$\frac{\text{Number of newborns who received all four elements of essential newborn care}}{\text{Total number of live births in the health facility}} \times 100$$

Data collection & dissemination:

- NA

Method of measurement:

- NA

1. Collect Data on Denominator
Denominator: Total number of live births in the health facility.

1.1 Include
- NA

1.2 Exclude
- NA

2. Collect Data on Numerator
Numerator: Number of newborns who received all four elements of essential newborn care.

2.1 Include
- NA

2.2 Exclude
- NA

3. This indicator will be stratified based on:
- NA

4. Data Sources

4.1 Preferred data Sources:

- Perinatal information system
- Charts

4.2 Other data Sources:

- case observation
- direct observation
- chart reviews
- exit interviews of mothers

5. Calculate:

Divide Number of newborns who received all four elements of essential newborn care by the Total number of live births in the health facility then multiply by 100

6. Possible Limitations:

- For the newborn record to be a reliable data source, staff must fill the record out consistently and accurately.

Data Collection Sheet

The following table contains detailed information and support from the literature on the measurement of the indicator.

Indicator	% of newborns receiving essential newborn care
Alternate name	Newborns receiving essential newborn care
Definition	Percent of newborns who received all four elements of essential newborn care:<br>immediate and thorough drying,<br>immediate skin-to-skin contact,<br>delayed cord clamping, and<br>initiation of breastfeeding in the first hour
Rationale	This indicator assesses the provision of a core component of recommended newborn thermal care at delivery, and can be used as a measure for the quality of and adherence to service protocols (Gage et al., 2005). Drying the newborn immediately after birth, skin-to-skin contact with the mother, wrapping the infant with a dry cloth or towel, keeping the newborn's head covered, and delayed bathing, ideally for 24 hours, are essential care practices for keeping the newborn warm. Early skin-to-skin contact with the mother also promotes bonding and facilitates the initiation of breastfeeding. These strategies can be used effectively at home deliveries, as well as at facilities, and can improve newborn health and survival. Since the highest period of risk for neonatal deaths is within the first 24 hours, this indicator measures the delivery of several key thermal care practices that can improve infant health outcomes.
Type of indicator	Impact
Unit of measure	Percentage
Method of measurement	NA
Formula	(Number of newborns who received all four elements of essential newborn care/Total number of live births in the health facility) x 100
Target	NA
Frequency	NA
Possible Limitations	▪ For the newborn record to be a reliable data source, staff must fill the record out consistently and accurately
Denominator inclusion criteria	▪ Total number of live births in the health facility.
Denominator exclusion criteria	▪ NA
Numerator inclusion criteria	▪ Number of newborns who received all four elements of essential newborn care.
Numerator exclusion criteria	▪ NA
Data sources	▪ Perinatal information system

	▪ Charts<br>▪ case observation<br>▪ direct observation<br>▪ chart reviews<br>▪ exit interviews of mothers

References:

USAID, Results Based Financing, Newborns receiving essential newborn care, https://www.measureevaluation.org/rbf/indicator-collections/service-quality-indicators/quality-of-newborn-care

For the above indicator, can you provide the below information:

- Can the data elements (numerator and denominator) be collected in the context of Jordan?

[X] Yes Comments:

[ ] No Comments:

- Are the data sources listed above applicable (i.e. information can be extracted from the suggested data source) in the context of Jordan? If no, please provide the source.

[ ] Yes Comments: Hospital based reports

[ ] No Comments:

- Can the data for the above indicator be segregated for nationals and refugees?

[X] Yes Comments: could be segregated based on social security number for Jordanians and not Jordanians

[ ] No Comments:

- Is the data for Jordan reported in the same way as indicated above? *If you answer "No", please indicate in the comments section how it is being reported. If the indicator is not being reported in Jordan, please insert "Not reported" in the comments section*

[ ] Yes Comments:

[x] No Comments: Total cases

- Is the data for this indicator available for the period extending from January 2019 to December 2021? (if yes, please provide the data)

[ ] Yes Comments:

[ ] No Comments:

Proportion of pregnant women with hypertension receiving antihypertensive drugs

Definition:

The percent of women ages 15 to 49 with a live birth within a given time period who had their blood pressure checked at their first visit for antenatal care (ANC).

Formula

$$\frac{\text{Number of women ages 15 to 49 with a live birth whose blood pressure was checked at their first ANC visit}}{\text{Total number of women ages 15 - 49 with live births within reference period}} \times 100$$

Data collection & dissemination:

- Data dissemination in a period up to five years

Method of measurement:

- NA

1. Collect Data on Denominator
Denominator: Total number of women ages 15 - 49 with live births within reference period

1.1 Include

- NA

1.2 Exclude

- NA

2. Collect Data on Numerator
Numerator: Number of women ages 15 to 49 with a live birth whose blood pressure was checked at their first ANC visit

2.1 Include

- NA

2.2 Exclude

- NA

3. This indicator will be stratified based on:

- Type of facility (public, private, non-governmental, community-based),
- District
- Urban rural location.

4. Data Sources

4.1 Preferred data Sources:

- Population-based surveys, such as DHS, RHS, and MICS
- Facility records
- Health services data

4.2 Other data Sources:

- NA

5. Calculate:

Divide Number of women ages 15 to 49 with a live birth whose blood pressure was checked at their first ANC visit by the Total number of women ages 15 – 49 with live births within reference period then multiply by 100

6. Possible Limitations:

- Recall error is a potential source of bias
- Routine health records will not include information for pregnancies occurring outside the public health sector, including home and private facility deliveries.

Data Collection Sheet

The following table contains detailed information and support from the literature on the measurement of the indicator.

Indicator	Proportion of pregnant women with hypertension receiving antihypertensive drugs
Alternate name	Percent of pregnant women whose blood pressure was checked at first ANC visit
Definition	The percent of women ages 15 to 49 with a live birth within a given time period who had their blood pressure checked at their first visit for antenatal care (ANC).
Rationale	This indicator measures whether ANC facilities are consistently measuring women's blood pressure as early as possible in their pregnancies, and can serve as a proxy for the quality of ANC care. Women's blood pressure should be monitored at each ANC visit and during delivery. A blood pressure measure early in pregnancy (ideally in the first trimester) can help distinguish whether women have chronic high blood pressure (or hypertension), which was present before pregnancy, or a pregnancy-induced hypertension which occurs after 20 weeks gestation, during labor, or within 48 hours of delivery (WHO, 2008). Women with chronic hypertension can benefit from treatment and continued monitoring during pregnancy. For women with pregnancy-induced hypertension after 20 weeks, their condition may progress from a mild hypertension to pre-eclampsia, then to the life-threatening condition of eclampsia. If pre-eclampsia is detected and appropriately managed before the onset of convulsions and other life-threatening complications, women's risk of developing eclampsia can be reduced. Eclampsia accounts for about 12 percent of maternal deaths (WHO, 2008). This indicator relates to achieving Millennium Development Goals #5. improve maternal health and reduce child mortality.
Type of indicator	Impact
Unit of measure	Percentage
Method of measurement	NA
Formula	(Number of women ages 15 to 49 with a live birth whose blood pressure was checked at their first ANC visit/Total number of women ages 15 - 49 with live births within reference period) x 100
Target	NA
Frequency	▪ Data dissemination in a period up to five years
Possible Limitations	▪ Recall error is a potential source of bias<br>▪ Routine health records will not include information for pregnancies occurring outside the public health sector, including home and private facility deliveries
Denominator inclusion criteria	▪ Total number of women ages 15 - 49 with live births within reference period
Denominator exclusion criteria	▪ NA

Numerator inclusion criteria	▪ Number of women ages 15 to 49 with a live birth whose blood pressure was checked at their first ANC visit
Numerator exclusion criteria	▪ NA
Data sources	▪ Population-based surveys, such as DHS, RHS, and MICS<br>▪ Facility records<br>▪ Health services data

References:

USAID, Percent of pregnant women whose blood pressure was checked at first ANC visit https://www.data4impactproject.org/prh/womens-health/safe-motherhood/percent-of-pregnant-women-whose-blood-pressure-was-checked-at-first-anc-visit/

For the above indicator, can you provide the below information:

- Can the data elements (numerator and denominator) be collected in the context of Jordan?

[X] Yes Comments:

[ ] No Comments:

- Are the data sources listed above applicable (i.e. information can be extracted from the suggested data source) in the context of Jordan? If no, please provide the source.

[ ] Yes Comments: Hospital based reports

[ ] No Comments:

- Can the data for the above indicator be segregated for nationals and refugees?

[X] Yes Comments: could be segregated based on social security number for Jordanians and not Jordanians

[ ] No Comments:

- Is the data for Jordan reported in the same way as indicated above? *If you answer "No", please indicate in the comments section how it is being reported. If the indicator is not being reported in Jordan, please insert "Not reported" in the comments section*

[ ] Yes Comments:

[x] No Comments: Total cases

- Is the data for this indicator available for the period extending from January 2019 to December 2021? (if yes, please provide the data)

[ ] Yes Comments:

[ ] No Comments:

Percentage of mothers who received counselling, support or messages on optimal breastfeeding at least once in the last year

Definition:

The indicator gives the percentage of mothers of children aged 0-23 months who have received counselling, support or messages on optimal breastfeeding at least once in the last year.

Formula

$$\frac{\text{Number of mothers of children aged 0-23 months who have received counselling, support or messages on optimal breastfeeding at least once in the last year}}{\text{Total number of mothers of children aged 0-23 months in the last year}} \times 100$$

Data collection & dissemination:

- Collect information on this indicator on annual basis.
- Dissemination of information on annual basis.

Method of measurement:

- NA

1. Collect Data on Denominator
Denominator: Total number of mothers of children aged 0-23 months in the last year

1.1 Include

1.2 Exclude
- NA

1.3 Denominator Collection modality

2. Collect Data on Numerator
Numerator: Number of mothers of children aged 0-23 months who have received counselling, support or messages on optimal breastfeeding at least once in the last year

2.1 Include

2.2 Exclude

3. This indicator will be stratified based on:
- Urban/rural, region

4. Data Sources
4.1 Preferred data Sources:
- NutriDash (UNICEF internal data collection platform)

4.2 Other data Sources:

5. Calculate:

Divide the Number of mothers of children aged 0-23 months who have received counselling, support or messages on optimal breastfeeding at least once in the last year by the Total number of mothers of children aged 0-23 months in the last year then multiply by 100

6. Possible Limitations:

- WHO and UNICEF are in the process of further developing and validating this indicator.

Data Collection Sheet

The following table contains detailed information and support from the literature on the measurement of the indicator.

Indicator	Percentage of mothers who received counselling, support or messages on optimal breastfeeding at least once in the last year
Alternate name	Mothers of children 0-23 months receiving counselling, support or messages on optimal breastfeeding at least once in the last year<br>OR<br>Proportion of mothers of children 0-23 months who have received counselling, support or messages on optimal breastfeeding at least once in the last year
Definition	The indicator gives the percentage of mothers of children aged 0-23 months who have received counselling, support or messages on optimal breastfeeding at least once in the last year.
Rationale	Breastfeeding is an unequalled way of providing ideal food for the healthy growth and development of infants; it is also an integral part of the reproductive process, with important implications for the health of mothers. Optimal practices include early initiation of breastfeeding within 1 hour and exclusive breastfeeding for 6 months, followed by appropriate complementary with continued breastfeeding for 2 years or beyond. Although it is a natural act, breastfeeding is also a learnt behaviour. Almost all mothers can breastfeed, provided they have accurate information and have support within their families and communities, and from the health care system. Mothers should also have access to skilled practical help from, for example, trained health workers, lay and peer counsellors, and certified lactation consultants. These professionals can help to build a mother's confidence, improve feeding technique, and prevent or resolve breastfeeding problems.<br><br>This indicator has been established to measure the proportion of mothers receiving breastfeeding counselling, support or messages. The proportion of mothers of children aged 0-23 months who have received counselling, support or messages on optimal breastfeeding at least once in the previous 12 months is included as a process indicator in the core set of indicators for the Global Nutrition Monitoring Framework.
Type of indicator	Process
Unit of measure	Percentage
Method of measurement	NA
Formula	(Number of mothers of children aged 0-23 months who have received counselling, support or messages on optimal breastfeeding at least once in the last year/ Total number of mothers of children aged 0-23 months in the last year) X100
Target	NA
Frequency	▪ Collect information on this indicator on annual basis.<br>▪ Dissemination of information on annual basis.
Possible Limitations	▪ WHO and UNICEF are in the process of further developing and validating this indicator.
Denominator	Total number of mothers of children aged 0-23 months in the last year

inclusion criteria	
Denominator exclusion criteria	NA
Numerator inclusion criteria	Number of mothers of children aged 0-23 months who have received counselling, support or messages on optimal breastfeeding at least once in the last year
Numerator exclusion criteria	NA
Data sources	▪ NutriDash (UNICEF internal data collection platform)

References:

WHO, NUTRITION LANDSCAPE INFORMATION SYSTEM (NLiS), Nutrition and nutrition-related health and development data, Mothers of children 0-23 months receiving counselling, support or messages on optimal breastfeeding at least once in the last year https://www.who.int/data/nutrition/nlis/info/mothers-of-children-0-23-months-receiving-counselling-support-or-messages-on-optimal-breastfeeding-at-least-once-in-the-last-year

WHO, Indicators for the Global Monitoring Framework on Maternal, Infant and Young Child Nutrition (24 November 2014) https://www.who.int/nutrition/topics/indicators_monitoringframework_miycn_background.pdf?ua=1

For the above indicator, can you provide the below information:

- Can the data elements (numerator and denominator) be collected in the context of Jordan?

[X] Yes Comments:

[ ] No Comments:

- Are the data sources listed above applicable (i.e. information can be extracted from the suggested data source) in the context of Jordan? If no, please provide the source.

[ ] Yes Comments: Hospital based reports

[ ] No Comments:

- Can the data for the above indicator be segregated for nationals and refugees?

[X] Yes Comments: could be segregated based on social security number for Jordanians and not Jordanians

[ ] No Comments:

- Is the data for Jordan reported in the same way as indicated above? *If you answer "No", please indicate in the comments section how it is being reported. If the indicator is not being reported in Jordan, please insert "Not reported" in the comments section*

[ ] Yes Comments:

[x] No Comments: Total cases

- Is the data for this indicator available for the period extending from January 2019 to December 2021? (if yes, please provide the data)

[ ] Yes Comments:

[ ] No Comments:

Vitamin A supplementation coverage (% of children aged 6-59 months who received two age appropriate doses of vitamin A in the past 12 months)*

Definition:

Percentage of children aged 6–59 months who received a high dose vitamin A supplement within the six months prior to the survey. A high dose vitamin A supplement, according to the International Vitamin A Consultative Group (IVACG) definition, refers to doses equal to or greater than 25 000 IU.

Formula

$$\frac{\text{Number of children aged 6–59 months receiving at least one high dose vitamin A supplement within the six months prior to the survey}}{\text{Total number of children aged 6–59 months surveyed}} \times 100$$

Data collection & dissemination:

- NA

Method of measurement:

Data are derived from re-analysis of Demographic and Health Surveys (DHS), Multiple Indicator Cluster Surveys (MICS) and Reproductive Health Surveys (RHS) micro-data which are publicly available using the standard indicator definitions as published in DHS, MICS or RHS documentation. The analysis was done by the WHO Collaborating Center for Health Equity Monitoring (International Center for Equity in Health, Federal University of Pelotas, Brazil).

1. Collect Data on Denominator
Denominator: Total number of children aged 6–59 months surveyed.

1.1 Include
- NA

1.2 Exclude
- NA

1.3 Denominator Collection modality

2. Collect Data on Numerator
Numerator: Number of children aged 6–59 months receiving at least one high dose vitamin A supplement within the six months prior to the survey.

2.1 Include
- NA

2.2 Exclude
- NA

3. This indicator will be stratified based on:

- Economic status (wealth quintile and wealth decile)
- Education (mother's education)
- Place of residence
- Sex
- Subnational region
- Age

4. Data Sources

4.1 Preferred data Sources:

- Demographic and Health Surveys (DHS)
- Multiple Indicator Cluster Surveys (MICS)
- Reproductive Health Surveys (RHS) micro-data
- Household surveys
- Routine facility information systems

4.2 Other data Sources:

- NA

5. Calculate:

Divide the Number of children aged 6–59 months receiving at least one high dose vitamin A supplement within the six months prior to the survey by the Total number of children aged 6–59 months surveyed and multiply by 100

6. Possible Limitations:

- Please note that the above definition applies to the Health Equity Monitor topic of the WHO Indicator and Measurement Registry. In some cases, indicators listed in this topic may not be equivalent to similar indicators listed in other topics of the registry, due to small discrepancies in the definition and calculation of numerator and denominator values.
- This indicator is a coverage indicator and does not provide any information regarding the prevalence of vitamin A deficiency.
- Although oral supplementation is used for both treatment and prevention of vitamin A deficiency, it is not recommended as the only long-term approach. In the home, vitamin A deficiency can be prevented by the regular consumption of vitamin A-rich foods, including fortified foods.
- Service statistics are generally not representative of an entire population. Since the quality of health statistics can vary among facilities, indicators calculated from service statistics may be less accurate than those based on survey data in places where the quality of routine data is poor.
- It may be difficult to estimate the denominator for indicators based on service statistics. The population denominators are often extrapolated from census data that are several years old. If population growth and rural-urban migration patterns have substantially changed over time, then census information may be unsuitable for providing appropriate denominators for local program managers to determine vitamin A supplementation coverage.

Data Collection Sheet

The following table contains detailed information and support from the literature on the measurement of the indicator.

Indicator	Vitamin A supplementation coverage (% of children aged 6-59 months who received two age appropriate doses of vitamin A in the past 12 months)
Alternate name	Children aged 6–59 months who received vitamin A supplementation (%)<br>OR<br>Vitamin A supplementation coverage
Definition	Percentage of children aged 6–59 months who received a high dose vitamin A supplement within the six months prior to the survey. A high dose vitamin A supplement, according to the International Vitamin A Consultative Group (IVACG) definition, refers to doses equal to or greater than 25 000 IU.
Rationale	The indicator is defined as the proportion of children aged 6–59 months who received two age-appropriate doses of vitamin A supplements in the last 12 months. This indicator measures the coverage achieved through national vitamin A supplementation program efforts in a specified period.<br><br>Vitamin A deficiency (VAD) is a major public health problem in developing countries. WHO estimates that between 100 and 140 million children are vitamin A deficient. For children, lack of vitamin A causes visual impairment, blindness, and significantly increases the risk of severe illness and death from common childhood infections such as diarrheal disease and measles. Supplementation as a vitamin A deficiency control strategy is the most immediate and direct approach to improving vitamin A status and the one most widely implemented.<br><br>Programmes to control vitamin A deficiency enhance children's chances of survival, reduce the severity of childhood illnesses, ease the strain on health systems and hospitals and contribute to the well-being of children, their families and communities. Supplementation with vitamin A is a safe, cost-effective, efficient means for eliminating its deficiency and improving child survival.
Type of indicator	NA
Unit of measure	Percentage
Method of measurement	Data are derived from re-analysis of Demographic and Health Surveys (DHS), Multiple Indicator Cluster Surveys (MICS) and Reproductive Health Surveys (RHS) micro-data which are publicly available using the standard indicator definitions as published in DHS, MICS or RHS documentation. The analysis was done by the WHO Collaborating Center for Health Equity Monitoring (International Center for Equity in Health, Federal University of Pelotas, Brazil).
Formula	(Number of children aged 6–59 months receiving at least one high dose vitamin A supplement within the six months prior to the survey / Total number of children aged 6–59 months surveyed) X100
Target	NA
Frequency	NA
Possible Limitations	▪ Please note that the above definition applies to the Health Equity Monitor topic of the WHO Indicator and Measurement Registry. In

	some cases, indicators listed in this topic may not be equivalent to similar indicators listed in other topics of the registry, due to small discrepancies in the definition and calculation of numerator and denominator values.<br>▪ This indicator is a coverage indicator and does not provide any information regarding the prevalence of vitamin A deficiency.<br>▪ Although oral supplementation is used for both treatment and prevention of vitamin A deficiency, it is not recommended as the only long-term approach. In the home, vitamin A deficiency can be prevented by the regular consumption of vitamin A-rich foods, including fortified foods.<br>▪ Service statistics are generally not representative of an entire population. Since the quality of health statistics can vary among facilities, indicators calculated from service statistics may be less accurate than those based on survey data in places where the quality of routine data is poor.<br>▪ It may be difficult to estimate the denominator for indicators based on service statistics. The population denominators are often extrapolated from census data that are several years old. If population growth and rural-urban migration patterns have substantially changed over time, then census information may be unsuitable for providing appropriate denominators for local program managers to determine vitamin A supplementation coverage.
Denominator inclusion criteria	Total number of children aged 6–59 months surveyed.
Denominator exclusion criteria	NA
Numerator inclusion criteria	Number of children aged 6–59 months receiving at least one high dose vitamin A supplement within the six months prior to the survey.
Numerator exclusion criteria	NA
Data sources	▪ Demographic and Health Surveys (DHS)<br>▪ Multiple Indicator Cluster Surveys (MICS)<br>▪ Reproductive Health Surveys (RHS) micro-data<br>▪ Household surveys<br>▪ Routine facility information systems

References:

WHO, Global Health Observatory, Health equity monitor: Children aged 6-59 months who received vitamin A supplementation (%), https://www.who.int/data/gho/data/indicators/indicator-details/GHO/hem-children-aged-6-59-months-who-received-vitamin-a-supplementation-(-)

World Health Organization (WHO). 2015 Global Reference List of 100 Core Health Indicators.; 2015. http://apps.who.int/iris/bitstream/10665/173589/1/WHO_HIS_HSI_2015.3_eng.pdf

World Health Organization. Nutrition Landscape Information System (NLIS). Country Profile Indicators: Interpretation Guide. Geneva, Switzerland; 2010. http://apps.who.int/iris/bitstream/10665/44397/1/9789241599955_eng.pdf

Gage AJ, Ali D, Suzuki C. A Guide for Monitoring and Evaluating Child Health Programs. MEASURE Evaluation. Carolina Population Center, University of North Carolina at Chapel Hill.; 2005. http://www.coregroup.org/storage/documents/Workingpapers/ms-05-15.pdf

Measure Evaluation, Vitamin A supplementation coverage https://www.measureevaluation.org/rbf/indicator-collections/service-use-and-coverage-indicators/vitamin-a-supplementation-coverage.html

For the above indicator, can you provide the below information:

- Can the data elements (numerator and denominator) be collected in the context of Jordan?

[X] Yes Comments:

[ ] No Comments:

- Are the data sources listed above applicable (i.e. information can be extracted from the suggested data source) in the context of Jordan? If no, please provide the source.

[X] Yes Comments:

[ ] No Comments:

- Can the data for the above indicator be segregated for nationals and refugees?

[x] Yes Comments:

[ ] No Comments:

- Is the data for Jordan reported in the same way as indicated above? *If you answer "No", please indicate in the comments section how it is being reported. If the indicator is not being reported in Jordan, please insert "Not reported" in the comments section*

[ ] Yes Comments:

[ ] No Comments: Not reported

- Is the data for this indicator available for the period extending from January 2019 to December 2021? (if yes, please provide the data)

[ ] Yes Comments:

[ ] No Comments:

Dimension 4:

The proportion of women with severe pre-eclampsia or eclampsia who receive magnesium sulfate therapy

Definition:

The proportion of women with severe pre-eclampsia or eclampsia who receive magnesium sulfate therapy.

Pre-eclampsia: Onset of a new episode of hypertension during pregnancy, characterized by:

• Persistent hypertension (diastolic blood pressure $\geq$ 90 mm Hg) and

• Substantial proteinuria (> 0.3 g/24 hours)

Eclampsia:

• Generalized seizures, generally in addition to pre-eclampsia criteria

Formula:

(Number of women with severe pre-eclampsia/eclampsia receiving magnesium sulfate *100/ The total number of women who present with severe pre-eclampsia/eclampsia)

Method of measurement:

1. Collect Data on Denominator
The total number of women who present with severe pre-eclampsia/eclampsia

11.2 Include
12

12.1 Exclude

2. Collect Data on Numerator
Number of women with severe pre-eclampsia/eclampsia receiving magnesium sulfate

2.1. Include

2.2. Exclude

3. Stratification Options

4. Preferred Data Sources
Health facilities Surveys

5. Other Data Sources

Data collection sheet

Indicator	The proportion of women with severe pre-eclampsia or eclampsia who receive magnesium sulfate therapy
Definition	The proportion of women with severe pre-eclampsia or eclampsia who receive magnesium sulfate therapy.<br><br>Pre-eclampsia: Onset of a new episode of hypertension during pregnancy, characterized by:<br><br>• Persistent hypertension (diastolic blood pressure ≥ 90 mm Hg) and<br><br>• Substantial proteinuria (> 0.3 g/24 hours)<br><br>Eclampsia:<br><br>• Generalized seizures, generally in addition to pre-eclampsia criteria
Rationale	Nearly one-tenth of maternal deaths in Asia and Africa and one-quarter of maternal deaths in Latin America are associated with hypertensive disorders of pregnancy. Among the hypertensive disorders, pre-eclampsia and eclampsia have the greatest impact on maternal and newborn morbidity and mortality. Yet the majority of deaths related to pre-eclampsia and eclampsia could be avoided if women received timely and effective care, delivered according to evidence-based standards
Type of indicator	
Unit of measure	Percentage
Formula	(Number of women with severe pre-eclampsia/eclampsia receiving magnesium sulfate *100/ The total number of women who present with severe pre-eclampsia/eclampsia)
Target	
Frequency of collection	
Frequency of Dissemination	
Denominator inclusion criteria	Total number of women who present with severe pre-

	eclampsia/eclampsia
Denominator exclusion criteria	
Numerator inclusion criteria	Number of women with severe pre-eclampsia/eclampsia receiving magnesium sulfate
Numerator exclusion criteria	
Preferred Data sources	Health Facilities Survey
Other Data Sources	
Limitations/comments	

References:

Microsoft Word - PEE Briefer (who.int)

9789241507417_eng.pdf (who.int)

For the above indicator, can you provide the below information:

- Can the data elements (numerator and denominator) be collected in the context of Jordan?

☐ Yes Comments:

☐ No Comments:

- Are the data sources listed above applicable (i.e. information can be extracted from the suggested data source) in the context of Jordan? If no, please provide the source.

☐ Yes Comments:

☐ No Comments:

- Can the data for the above indicator be segregated for nationals and refugees?

☐ Yes Comments:

☐ No Comments:

- Is the data for Jordan reported in the same way as indicated above? *If you answer "No", please indicate in the comments section how it is being reported. If the indicator is not being reported in Jordan, please insert "Not reported" in the comments section*

☐ Yes Comments:

☐ No Comments:

- Is the data for this indicator available for the period extending from January 2019 to December 2021? (if yes, please provide the data)

☐ Yes Comments:

☐ No Comments:

Proportion of health facilities with safe, uninterrupted oxygen supply in childbirth, neonatal and pediatric wards

Definition:

This indicator measures the availability of physical resources at the facility. Access to oxygen in labor and delivery, neonatal, and pediatric wards is intended to be available within the context of functioning health facility at all times and in adequate amounts.

Formula:

$$\frac{\text{Number of health facilities with safe, uninterrupted oxygen supply in childbirth, neonatal and paediatric wards}}{\text{Total number of health facilities}} \times 100$$

Method of measurement:

1. Collect Data on Denominator

2.3 Include denominator

Total number of health facilities

2.4 Exclude denominator

NA

2. Collect Data on Numerator

2.1 Include numerator

Number of health facilities with safe, uninterrupted oxygen supply in childbirth, neonatal and paediatric wards

2.3 Exclude numerator

3. Stratification Options
Ward or facility type, geographic location, managing authority, programme

4. Preferred Data Sources
Health facility survey

5. Other Data Sources

Data collection sheet

Indicator	Percent of health facilities with safe, uninterrupted oxygen supply in childbirth, neonatal and paediatric wards
Alternative Indicator	
Definition	Percent of health facilities with safe, uninterrupted oxygen supply in childbirth, neonatal and paediatric wards
Rationale	This indicator measures the availability of physical resources at the facility. Access to oxygen in labor and delivery, neonatal, and pediatric wards is intended to be available within the context of functioning health facility at all times and in adequate amounts.
Type of indicator	Structure
Unit of measure	%
Formula	(Number of health facilities with safe, uninterrupted oxygen supply in childbirth, neonatal and paediatric wards/ Total number of health facilities) x 100
Target	Not specified
Frequency of collection	Annual
Frequency of Dissemination	Annual
Denominator inclusion criteria	Total number of health facilities
Denominator exclusion criteria	
Numerator inclusion criteria	Number of health facilities with safe, uninterrupted oxygen supply in childbirth, neonatal and paediatric wards
Numerator exclusion criteria	
Preferred Data sources	Health facility survey

Other Data Sources	
Limitations/comments	

References:

Measure evaluation, Percent of health facilities with safe, uninterrupted oxygen supply in childbirth, neonatal and paediatric wards, https://www.measureevaluation.org/rbf/indicator-collections/structural-indicators/proportion-of-health-facilities-with-safe-uninterrupted-oxygen-supply-in-childbirth-neonatal-and-paediatric-wards/generate_doc

World Health Organization (WHO). Consultation on Improving Measurement of the Quality of Maternal, Newborn and Child Care in Health Facilities.;
2013. http://apps.who.int/iris/bitstream/10665/128206/1/9789241507417_eng.pdf

For the above indicator, can you provide the below information: NA

- Can the data elements (numerator and denominator) be collected in the context of Jordan?

☐ Yes Comments:

☐ No Comments:

- Are the data sources listed above applicable (i.e. information can be extracted from the suggested data source) in the context of Jordan? If no, please provide the source.

☐ Yes Comments:

☐ No Comments:

- Can the data for the above indicator be segregated for nationals and refugees?

☐ Yes Comments:

☐ No Comments:

- Is the data for Jordan reported in the same way as indicated above? *If you answer "No", please indicate in the comments section how it is being reported. If the indicator is not being reported in Jordan, please insert "Not reported" in the comments section*

☐ Yes Comments:

☐ No Comments:

- Is the data for this indicator available for the period extending from January 2019 to December 2021? (if yes, please provide the data)

☐ Yes Comments:

☐ No Comments:

Percentage and distribution of health workers trained to provide reproductive maternal and child health

Formula

$$\frac{\text{Number of Physicians, nurses, midwives}}{\text{Total number of health workers}} \times 100$$

Data collection & dissemination:

- NA

Method of measurement:

- NA

1. Collect Data on Denominator

Denominator: Total number of health workers

1.1 Include

- NA

1.2 Exclude

- NA

2. Collect Data on Numerator

Numerator: Number of physicians, nurses and midwives (or other categories of health service providers)

2.1 Include

- NA

2.2 Exclude

- NA

3. This indicator will be stratified based on:

- Occupational classification – with distinction between headcounts versus job positions (with positions weighted for full-time equivalency on the basis of working

4. Data Sources

4.1 Preferred data Sources:

- Population census
- Labour force survey
- Civil service payroll registries
- Registries of professional regulatory bodies

4.2 Other data Sources:

- Health facility assessment

5. Calculate:

divide Number of physicians, nurses and midwives (or other categories of health service providers) by total number of health workers and multiply by 100

6. Possible Limitations:

- Sources of data need to be evaluated based on strengths and limitations to avoid exclusion of certain professional groups and double counting

Data Collection Sheet

The following table contains detailed information and support from the literature on the measurement of the indicator.

Indicator	Percenatge and distribution of health workers trained to provide reproductive maternal and child health
Alternate name	Distribution of HRH by occupation, specialization or other skill-related characteristic
Definition	NA
Rationale	Assessments of HRH are required for various purposes, notably for planning, implementing, monitoring and evaluating health sector strategies, programmes and interventions. The importance of sound empirical evidence for informed policy decision-making and monitoring of progress in strengthening health workforce development and management is widely recognized. Precisely describing HRH can help to identify opportunities and constraints for scaling up health interventions.
Type of indicator	NA
Unit of measure	Percentage
Method of measurement	▪ NA
Formula	Number of Physicians, nurses, midwives X 100<br>Total number of health workers
Target	NA
Frequency	NA
Possible Limitations	▪ Sources of data need to be evaluated based on strengths and limitations to avoid exclusion of certain professional groups and double counting
Denominator inclusion criteria	▪ Total number of health workers
Denominator exclusion criteria	▪ NA
Numerator inclusion criteria	▪ Number of physicians, nurses and midwives (or other categories of health service providers)
Numerator exclusion criteria	▪ NA
Data sources	▪ Population census<br>▪ Labour force survey<br>▪ Civil service payroll registries<br>▪ Registries of professional regulatory bodies<br>▪ Health facility assessment

References:

WHO, World Bank, USAID, Handbook on Monitoring and Evaluation of Human Resources for Health with special applications for low- and middle-income countries, https://apps.who.int/iris/bitstream/handle/10665/44097/9789241547703_eng.pdf?sequence=1

For the above indicator, can you provide the below information:

- Can the data elements (numerator and denominator) be collected in the context of Jordan?

[x] Yes Comments:

[ ] No Comments:

- Are the data sources listed above applicable (i.e. information can be extracted from the suggested data source) in the context of Jordan? If no, please provide the source.

[x] Yes Comments:

[ ] No Comments:

- Can the data for the above indicator be segregated for nationals and refugees?

[ ] Yes Comments:

[ ] No Comments:

- Is the data for Jordan reported in the same way as indicated above? *If you answer "No", please indicate in the comments section how it is being reported. If the indicator is not being reported in Jordan, please insert "Not reported" in the comments section*

[ ] Yes Comments:

[x] No Comments: Total cases

- Is the data for this indicator available for the period extending from January 2019 to December 2021? (if yes, please provide the data)

[ ] Yes Comments:

[ ] No Comments:

Density of midwives by district (by births)

Formula

$$\frac{\text{Number of midwives in year t}}{\text{Total population in a country in year t}} \text{ X 100}$$

Data collection & dissemination:

- NA

Method of measurement:

- The ratio expresses the frequency of cases (midwives) for a given number (10,000) of inhabitants. It is calculated by dividing the total number of midwives for a given year by the existing population in that same year.
- The number of people used as a reference in a density is conventional and depends on the obtained figures: it can vary from 1 inhabitant (per capita) and 100,000. In this case, it was defined by multiplying by 10,000.
- The year "t" refers to the year when the midwives data were collected and should coincide with the year in which the population data were collected.

1. Collect Data on Denominator
Denominator: Total population in the geographic unit (country, district…) in a specific year

1.1 Include
- NA

1.2 Exclude
- NA

2. Collect Data on Numerator
Numerator: Health Human Resources (midwives) employed full time in public and private sector in a specific year

2.1 Include
- NA

2.2 Exclude
- NA

3. This indicator will be stratified based on:

Facilitate type (international and inter-Regional)

4. Data Sources
4.1 Preferred data Sources:
- Administrative registries
- Census

4.2 Other data Sources:

- NA

5. Calculate:

It is calculated by dividing the total number of human resources (midwives) for a given year by the existing population in that same year and multiply by 10,000

6. Possible Limitations:

When uncertain whether a number of registered health human resources (midwives) still work in the country, it is best not to use the data. By taking only the data of employed personnel, you avoid the complications that would emerge if you took the records of those who graduated from universities, given that (1) it may include personnel that do not work or that changed their profession or occupation, or (2) may be migrant personnel who will be leaving the country.

Data Collection Sheet

The following table contains detailed information and support from the literature on the measurement of the indicator.

Indicator	Density of midwives by district (by births)
Alternate name	Health Human resources (Midwives) density ratio per 10,000 inhabitants
Definition	Number of health personnel (midwives) that are employed full-time in a given year in public and private health establishments expressed as the density per 10,000 population.
Rationale	The purpose of this goal is to illustrate the relationship between the population of a country and the number of midwives with the aim of identifying the possible under or over-supply of these resources. Global studies have found that few countries have been able to reach the minimum population health targets set out in the Millennium Development Goals with fewer than 25 professionals per 10,000 inhabitants.
Type of indicator	To be determined
Unit of measure	per 10,000 inhabitants
Method of measurement	▪ The ratio expresses the frequency of cases (midwives) for a given number (10,000) of inhabitants. It is calculated by dividing the total number of midwives for a given year by the existing population in that same year.<br>▪ The number of people used as a reference in a density is conventional and depends on the obtained figures: it can vary from 1 inhabitant (per capita) and 100,000. In this case, it was defined by multiplying by 10,000.<br>▪ The year "t" refers to the year when the midwives data were collected and should coincide with the year in which the population data were collected.
Formula	Number of midwives in year t x 10,000<br>Total population in a country in year t
Target	25 PROFESSIONALS PER 10,000 INHABITANTS
Frequency	NA
Possible Limitations	▪ personnel that do not work or that changed their profession or occupation,<br>▪ migrant personnel who will be leaving the country.
Denominator inclusion criteria	▪ Total population in the geographic unit (country, district…) in a specific year
Denominator exclusion criteria	▪ NA
Numerator inclusion criteria	▪ Health Human Resources (midwives) employed full time in public and private sector in a specific year
Numerator exclusion criteria	▪ NA
Data sources	▪ Administrative registries<br>▪ Census
Indicator	To be determined

responsibility	

References:

Pan American Health Organization. “Handbook for Measurement and Monitoring Indicators of the Regional Goals for Human Resources for Health: A Shared Commitment”. Washington, D.C.: PAHO, 2011. https://www.paho.org/hq/dmdocuments/2011/HSS-Regional-Goals-Handbook-2011.pdf

For the above indicator, can you provide the below information: NA

- Can the data elements (numerator and denominator) be collected in the context of Jordan?

☐ Yes Comments:

☐ No Comments:

- Are the data sources listed above applicable (i.e. information can be extracted from the suggested data source) in the context of Jordan? If no, please provide the source.

☐ Yes Comments:

☐ No Comments:

- Can the data for the above indicator be segregated for nationals and refugees?

☐ Yes Comments:

☐ No Comments:

- Is the data for Jordan reported in the same way as indicated above? *If you answer "No", please indicate in the comments section how it is being reported. If the indicator is not being reported in Jordan, please insert "Not reported" in the comments section*

☐ Yes Comments:

☐ No Comments:

- Is the data for this indicator available for the period extending from January 2019 to December 2021? (if yes, please provide the data)

☐ Yes Comments:

☐ No Comments:

Adolescent indicators

Dimension 1

Adolescent mortality rate (per 100 000 population)

Formula

$$\frac{\text{Number of deaths among adolescents [10-19 years old] in a given period}}{\text{Total number of adolescents during that period}} \times 100$$

Data collection & dissemination:

- Data collection continuous
- Data dissemination every 2-3 years

Method of measurement:

- **Civil or sample registration:** Mortality by age and sex are used to calculate age specific rates.
- **Census:** Mortality by age and sex tabulated from questions on recent deaths that occurred in the household during a given period preceding the census (usually 12 months).
- **Census or surveys:** Direct or indirect methods provide adult mortality rates based on information on survival of parents or siblings**.**

Collect Data on Denominator
Denominator: Total number of adolescents during that period

1.1 Include
- Total number of adolescents during that period

1.2 Exclude
- NA

2. Collect Data on Numerator
Numerator: Number of deaths among adolescents [10-19 years old] in a given period

2.1 Include
- Number of deaths among adolescents [10-19 years old] in a given period

2.2 Exclude
- NA

3. This indicator will be stratified based on:

- Sex Age

4. Data Sources

4.1 Preferred data Sources:

- Civil registration with complete coverage

4.2 Other data Sources:

- Household surveys
- Population census
- Sample or sentinel registration systems

5. Calculate:

Divide the number of deaths among adolescents [10-19 years old] in a given period by the total number of adolescents during that period and multiply by 100 000

6. Possible Limitations:

- NA

Data Collection Sheet

The following table contains detailed information and support from the literature on the measurement of the indicator.

Indicator	Adolescent mortality rate (per 100 000 population)
Dimension	D1
Definition	Number of deaths among adolescents (10-19 years old) per 100 000 adolescent population[12].
Rationale	NA
Type of indicator	Impact
Unit of measure	Deaths per 1000 population
Method of measurement	▪ Civil or sample registration: Mortality by age and sex are used to calculate age specific rates.<br>▪ Census: Mortality by age and sex tabulated from questions on recent deaths that occurred in the household during a given period preceding the census (usually 12 months).<br>▪ Census or surveys: Direct or indirect methods provide adult mortality rates based on information on survival of parents or siblings[12].
Formula	(Number of deaths among adolescents [10-19 years old] in a given period/Total number of adolescents during that period) x 100
Target	To be determined
Frequency	▪ Data collection continuous<br>▪ Data dissemination every 2-3 years
Possible Limitations	▪ NA
Denominator inclusion criteria	▪ Total number of adolescents during that period
Denominator exclusion criteria	▪ NA
Numerator inclusion criteria	▪ Number of deaths among adolescents [10-19 years old] in a given period
Numerator exclusion criteria	▪ NA
Data sources	▪ Civil registration with complete coverage<br>▪ Household surveys<br>▪ Population census<br>▪ Sample or sentinel registration systems

References:

Indicator Metadata Registry Details. (2020). Retrieved 17 December 2020, from https://www.who.int/data/gho/indicator-metadata-registry/imr-details/4751

World Health Organization, Data Portal, MATERNAL, NEWBORN, CHILD AND ADOLESCENT HEALTH AND AGEING, https://www.who.int/data/maternal-newborn-child-adolescent-ageing/indicator-explorer-new/mca/all-cause-mortality-rate-for-adolescents-aged-10-19-years

World Health Organization, Global Health Observatory, Adolescent mortality rate (per 100 000 population)https://www.who.int/data/gho/data/indicators/indicator-details/GHO/adolescent-mortality-rate-(per-100-000-population)

For the above indicator, can you provide the below information:

- Can the data elements (numerator and denominator) be collected in the context of Jordan?

[x] Yes Comments:

[ ] No Comments:

- Are the data sources listed above applicable (i.e. information can be extracted from the suggested data source) in the context of Jordan? If no, please provide the source.

[ ] Yes Comments: Hospital records

[ ] No Comments:

- Can the data for the above indicator be segregated for nationals and refugees?

[x] Yes Comments: Based on social security number

[ ] No Comments:

- Is the data for Jordan reported in the same way as indicated above? *If you answer "No", please indicate in the comments section how it is being reported. If the indicator is not being reported in Jordan, please insert "Not reported" in the comments section*

[ ] Yes Comments:

[x] No Comments: General mortality rate is reported; could be segregated based on age

- Is the data for this indicator available for the period extending from January 2019 to December 2021? (if yes, please provide the data)

[ ] Yes Comments:

[ ] No Comments:

Adolescent mortality rate from road traffic injuries

Definition:

The estimated proportion of adolescents aged 10–14 and 15–19, years who have died in a specified year due to road traffic accidents.

Formula:

$$\frac{\text{Deaths from motor vehicle accidents for people aged 10–14 and 15–19 years}}{\text{Adolescents aged 10–14 and 15–19 years}} \text{ X } 100{,}000$$

Method of measurement:

It should be noted that some countries have stopped reporting mortality data on motor-vehicle traffic injuries. The indicator on transport accidents is a proxy that allows a more comprehensive and up-to-date comparison to be made at the European level, although it should be understood that it includes deaths occurred in other types of transport accident (for example, railway and aviation). It is defined as including ICD-9 BTL code B47; ICD-9 codes 800–848; ICD-10 codes V01–V99; ex-USSR 175 list 160–162; ICD-10 Mortality Condensed list 1: 1096; and EUROSTAT list of 65 causes: 60. The injury rate includes individuals who sustained one or more serious or slight injuries, but also those who died immediately or within 30 days as a result of the accident. It should be noted that practices of data collection and reporting differ among countries.

Mortality rates show the average of the last three years available, as reported in the WHO health for all mortality database, November 2007 version. Data from 1980 are available in the database.

1. Collect Data on Denominator

Adolescents aged 10–14 and 15–19 years

1.1 Include denominator

1.2 Exclude denominator

NA

2. Collect Data on Numerator

Deaths from motor vehicle accidents for people aged 10–14 and 15–19 years

2.5 Include numerator
People aged 10 -19 years

2.4 Exclude numerator

3. Stratification Options
Type of accident (car accident, pedestrians and cyclists, motorcyclists and moped drivers, other), age and gender.

4. Preferred Data Sources
Death registry, hospital records

5. Other Data Sources

Household surveys, Population census, Sample or sentinel, Registration systems

Data collection sheet

Indicator	Adolescent mortality rate from road traffic injuries
Alternative Indicator	Mortality from road traffic injuries in children and young people
Definition	The estimated proportion of adolescents aged 10–14 and 15–19, years who have died in a specified year due to road traffic accidents.
Rationale	This is estimated to be the No.1 cause of death globally, for 10–19 year olds in 2012.<br><br>.
Type of indicator	Outcome
Unit of measure	Per 100,000 population
Formula	(Deaths from motor vehicle accidents for people aged 10–14 and 15–19 years/All deaths for measurement year) x 100,000
Target	Not specified
Frequency of collection	Annual
Frequency of Dissemination	Annual
Denominator inclusion criteria	Adolescents aged 10–14 and 15–19 years
Denominator exclusion criteria	
Numerator inclusion criteria	Deaths from motor vehicle accidents for people aged 0 – 24 years
Numerator exclusion criteria	
Preferred Data sources	Death registry, hospital records
Other Data Sources	

Limitations/comments	It should be noted that mortality rates and (in particular) injury rates for some countries may be biased owing to underreporting, especially in the central Asian republics, the Caucasus countries and some countries in the Balkans. Since health data recording and handling systems and practices vary between countries, so do the availability and accuracy of the data. Some countries are not able to ensure exact coding of underlying causes and complete registration of all deaths and injuries. In certain cases, under registration of deaths may be as high as 20% and this must be borne in mind when making comparisons between countries. This problem can be further aggravated by a lack of sufficiently accurate population estimates used as the denominator when calculating indicators. The problems are caused by a lack of surveillance through severe socioeconomic difficulties and armed conflicts in some countries.

References:

European Environment and Health Information System, Mortality from road traffic injuries in children and young people, December 2009, https://www.euro.who.int/__data/assets/pdf_file/0010/96976/2.1.-Mortality-from-road-traffic-injuries-EDITED_layouted_V2.pdf

WHO, Proposed health indicators for adolescents (ages 10–19 years), https://apps.who.int/adolescent/second-decade/images/Section_7/l7_1/1575_section%207.1.pdf

For the above indicator, can you provide the below information:

- Can the data elements (numerator and denominator) be collected in the context of Jordan?

[x] Yes   Comments:

[ ] No   Comments:

- Are the data sources listed above applicable (i.e. information can be extracted from the suggested data source) in the context of Jordan? If no, please provide the source.

[ ] Yes   Comments: Hospital records

[ ] No   Comments:

- Can the data for the above indicator be segregated for nationals and refugees?

[x] Yes   Comments: Based on social security number

[ ] No   Comments:

- Is the data for Jordan reported in the same way as indicated above? *If you answer "No", please indicate in the comments section how it is being reported. If the indicator is not being reported in Jordan, please insert "Not reported" in the comments section*

[ ] Yes   Comments:

[x] No   Comments: General mortality rate is reported; could be segregated for traffic accidents

- Is the data for this indicator available for the period extending from January 2019 to December 2021? (if yes, please provide the data)

[ ] Yes   Comments:

[ ] No   Comments:

Adolescent mortality rate from homicide

Definition:

Number of adolescent deaths due to homicide per 100 000 adolescent population

Formula:

(Number of deaths due to homicide among adolescents aged 10–14 and 15–19 years) /(Mid-year adolescent population (10–14 and 15–19 years) in a specified year) x 100

Method of measurement:

(Number of deaths due to homicide among adolescents aged 10–14 and 15–19 years) /(Mid-year adolescent population (10–14 and 15–19 years) in a specified year) x 100

1. Collect Data on Denominator

12.2 Include
13 Adolescent population

13.1 Exclude

2. Collect Data on Numerator

2.1. Include
Deaths due to homicide among adolescents

2.2. Exclude

3. Stratification Options
Sex and Age

4. Preferred Data Sources
WHO Global Mortality Database

United Nations Population Division (denominator)

5. Other Data Sources

Data collection sheet

Indicator	ADOLESCENT MORTALITY RATE FROM HOMICIDE
Definition	Number of adolescent deaths due to homicide per 100 000 adolescent population
Rationale	
Type of indicator	
Unit of measure	
Formula	Number of deaths due to homicide among adolescents aged 10–14 and 15–19 yearsx100,000 /Mid-year adolescent population (10–14 and 15–19 years) in a specified year
Target	
Frequency of collection	
Frequency of Dissemination	
Denominator inclusion criteria	Adolescents
Denominator exclusion criteria	
Numerator inclusion criteria	Deaths due to homicide aged 10-19
Numerator exclusion criteria	
Preferred Data sources	WHO Global Mortality Database<br><br>United Nations Population Division (denominator)
Other Data Sources	
Limitations/comments	

References:

9789241509626_eng.pdf (who.int)

For the above indicator, can you provide the below information:

- Can the data elements (numerator and denominator) be collected in the context of Jordan?

☐ Yes Comments:

☐ No Comments:

- Are the data sources listed above applicable (i.e. information can be extracted from the suggested data source) in the context of Jordan? If no, please provide the source.

☐ Yes Comments:

☐ No Comments:

- Can the data for the above indicator be segregated for nationals and refugees?

☐ Yes Comments:

☐ No Comments:

- Is the data for Jordan reported in the same way as indicated above? *If you answer "No", please indicate in the comments section how it is being reported. If the indicator is not being reported in Jordan, please insert "Not reported" in the comments section*

☐ Yes Comments:

☐ No Comments:

- Is the data for this indicator available for the period extending from January 2019 to December 2021? (if yes, please provide the data)

☐ Yes Comments:

☐ No Comments:

Adolescent maternal mortality ratio

Definition:

Number of maternal deaths among adolescents per 100 000 live births to adolescents

Formula:

(Number of maternal deaths from any cause related to or aggravated by pregnancy or its management (excluding accidental or incidental causes) during pregnancy and childbirth or within 42 days of termination of pregnancy, irrespective of the duration and site of the pregnancy, among adolescents aged 15–19 years in a specified period) x 100,000/ Number of live births to adolescents in the specified period

Method of measurement:

(Number of maternal deaths from any cause related to or aggravated by pregnancy or its management (excluding accidental or incidental causes) during pregnancy and childbirth or within 42 days of termination of pregnancy, irrespective of the duration and site of the pregnancy, among adolescents aged 15–19 years in a specified period) x 100,000/ Number of live births to adolescents in the specified period

1. Collect Data on Denominator

13.2 Include
Live births to adolescents

13.3 Exclude

2. Collect Data on Numerator

2.1. Include

14 Adolescent maternal deaths from any cause related to pregnancy and childbirth or within 42 days of termination of pregnancy

2.2. Exclude
Accidental or incidental causes

3. Stratification Options
This indicator should be disaggregated by age 15–19 years.

4. Preferred Data Sources
Civil registration and vital statistics systems

DHS

5. Other Data Sources

Data collection sheet

Indicator	ADOLESCENT MATERNAL MORTALITY RATIO
Definition	Number of maternal deaths among adolescents per 100 000 live births to adolescents
Rationale	
Type of indicator	
Unit of measure	
Formula	(Number of maternal deaths from any cause related to or aggravated by pregnancy or its management (excluding accidental or incidental causes) during pregnancy and childbirth or within 42 days of termination of pregnancy, irrespective of the duration and site of the pregnancy, among adolescents aged 15–19 years in a specified period) x 100,000/ Number of live births to adolescents in the specified period
Target	
Frequency of collection	
Frequency of Dissemination	
Denominator inclusion criteria	**Live births to adolescents**
Denominator exclusion criteria	
Numerator inclusion criteria	Adolescent maternal deaths from any cause related to pregnancy and childbirth or within 42 days of termination of pregnancy
Numerator exclusion criteria	**Accidental or incidental causes**
Preferred Data sources	Civil registration and vital statistics systems ,DHS ,United Nations Maternal Mortality Estimation Inter-agency Group
Other Data Sources	
Limitations/comments	

References:

9789241509626_eng.pdf (who.int)

For the above indicator, can you provide the below information:

- Can the data elements (numerator and denominator) be collected in the context of Jordan?

☐ Yes Comments:

☐ No Comments:

- Are the data sources listed above applicable (i.e. information can be extracted from the suggested data source) in the context of Jordan? If no, please provide the source.

☐ Yes Comments:

☐ No Comments:

- Can the data for the above indicator be segregated for nationals and refugees?

☐ Yes Comments:

☐ No Comments:

- Is the data for Jordan reported in the same way as indicated above? *If you answer "No", please indicate in the comments section how it is being reported. If the indicator is not being reported in Jordan, please insert "Not reported" in the comments section*

☐ Yes Comments:

☐ No Comments:

- Is the data for this indicator available for the period extending from January 2019 to December 2021? (if yes, please provide the data)

☐ Yes Comments:

☐ No Comments:

Prevalence of HIV infection among adolescents

Definition:

Percentage of adolescents living with HIV in the general population

Formula:

(Number of adolescents aged 10–14 and 15–19 years living with HIV) /(Number of adolescents aged 10–14 and 15–19 years in the population)x 100

Method of measurement:

1. Collect Data on Denominator
2.6 Include denominator
Total number of adolescents

2.7 Exclude denominator
NA

2. Collect Data on Numerator

2.1 Include numerator
Number of adolescents living with HIV

2.5 Exclude numerator

3. Stratification Options
Gender , age

4. Preferred Data Sources
National HIV registry

5. Other Data Sources
HIV prevalence survey

Data collection sheet

Indicator	Prevalence of HIV infection among adolescents
Alternative Indicator	Adolescents living with HIV
Definition	Percentage of adolescents living with HIV in the general population
Rationale	
Type of indicator	Outcome
Unit of measure	Percentage
Formula	(Number of adolescents aged 10–14 and 15–19 years living with HIV) / Number of adolescents aged 10–14 and 15–19 years in the population x 100
Target	Not specified
Frequency of collection	Annual
Frequency of Dissemination	Annual
Denominator inclusion criteria	Number of adolescents living with HIV
Denominator exclusion criteria	
Numerator inclusion criteria	Total number of adolescents
Numerator exclusion criteria	
Preferred Data sources	HIV national registry
Other Data Sources	HIV prevalence survey
Limitations/comments	

References:

WHO, Proposed health indicators for adolescents (ages 10–19 years), https://apps.who.int/adolescent/second-decade/images/Section_7/l7_1/1575_section%207.1.pdf

9789241509626_eng.pdf (who.int)

For the above indicator, can you provide the below information:

- Can the data elements (numerator and denominator) be collected in the context of Jordan?

[x] Yes Comments:

[ ] No Comments:

- Are the data sources listed above applicable (i.e. information can be extracted from the suggested data source) in the context of Jordan? If no, please provide the source.

[ ] Yes Comments: Hospital records

[ ] No Comments:

- Can the data for the above indicator be segregated for nationals and refugees?

[x] Yes Comments: Based on social security number

[ ] No Comments:

- Is the data for Jordan reported in the same way as indicated above? *If you answer "No", please indicate in the comments section how it is being reported. If the indicator is not being reported in Jordan, please insert "Not reported" in the comments section*

[ ] Yes Comments:

[x] No Comments: Total cases

- Is the data for this indicator available for the period extending from January 2019 to December 2021? (if yes, please provide the data)

[ ] Yes Comments:

[ ] No Comments:

Prevalence of depression among adolescents

Definition:

Proportion of adolescents who report experiencing symptoms of depression in a specified period

Formula:

Alternative 1:

Number of adolescents (> 15 years) who screen positive on the six depression questions in the WMH-CIDI screening tool / Total number of respondents aged 15–19 years in the survey x 100%

Alternative 2:

Number of adolescents aged 13–17 years in the GSHS who report having felt lonely and/or having felt so worried about something that they could not sleep (two separate items) during the past 12 months/ Total number of respondents aged 13–17 years in the survey x 100%

Method of measurement:

Alternative 1:

Number of adolescents (> 15 years) who screen positive on the six depression questions in the WMH-CIDI screening tool / Total number of respondents aged 15–19 years in the survey x 100%

Alternative 2:

Number of adolescents aged 13–17 years in the GSHS who report having felt lonely and/or having felt so worried about something that they could not sleep (two separate items) during the past 12 months/ Total number of respondents aged 13–17 years in the survey x 100%

1. Collect Data on Denominator

14.1 Include

15

16 Alternative 1: Total number of respondents aged 15-19

17 Alternative 2 : Total number of respondents aged 13-17

17.1 Exclude

2. Collect Data on Numerator

2.1. Include
Alternative 1: Information on the number of adolescents (aged 10–19 years, depending on coverage in the sample) in the World Mental Health Composite International Diagnostic Interview (WMH-CIDI) survey who screened positive with the tool .

Alternative 2: Information on the number of adolescents in the GSHS who reported "having felt lonely" or "having felt so worried they could not sleep" in the past 12 months

2.2. Exclude

3. Stratification Options
sex

4. Preferred Data Sources
- WMH-CIDI survey; data on adolescents are currently available only for subsamples in six countries and were collected for research purposes

- GSHS ("How often have you felt 'lonely' and/or 'worried' during the past 12 months?")

- HBSC ("How often have you felt 'low' and/or 'irritable and in a bad temper'?")

5. Other Data Sources

Prevalence of anaemia among adolescents (10-19 years), by age category (10-14, 15-19 years) and sex

Definition:

Percentage of adolescents (10-19 years) who have a haemoglobin level below 11 g/dl for males and 12 g/dl for females, at sea level, in a country within a given year, by age group (10-14, 15-19 years) and sex

Formula:

Number of female adolescents (10-19 years) who have a haemoglobin level less than 11 g/dl and male adolescents less than 12 g/dl at sea level in a country in a given year*100/ Total population of female and male adolescents (10-19 years) in the same country in the same year

Method of measurement:

1. Collect Data on Denominator

17.2 Include

Total population of female and male adolescents (10-19 years) in the same country in the same year
17.3 Exclude

2. Collect Data on Numerator
2.1. Include
Number of female adolescents (10-19 years) who have a haemoglobin level less than 11 g/dl and male adolescents less than 12 g/dl
2.2. Exclude

3. Stratification Options
Age group (10-14, 15-19 years); Sex

4. Preferred Data Sources
National surveillance system; Population-based survey; School-based survey
5. Other Data Sources

Data collection sheet

Indicator	Prevalence of anaemia among adolescents (10-19 years), by age category (10-14, 15-19 years) and sex
Definition	Percentage of adolescents (10-19 years) who have a haemoglobin level below 11 g/dl for males and 12 g/dl for females, at sea level, in a country within a given year, by age group (10-14, 15-19 years) and sex
Rationale	
Type of indicator	Outcome
Unit of measure	
Formula	Number of female adolescents (10-19 years) who have a haemoglobin level less than 11 g/dl and male adolescents less than 12 g/dl at sea level in a country in a given year*100/ Total population of female and male adolescents (10-19 years) in the same country in the same year
Target	
Frequency of collection	
Frequency of Dissemination	
Denominator inclusion criteria	Total population of female and male adolescents (10-19 years) in the same country in the same year
Denominator exclusion criteria	
Numerator inclusion criteria	Number of female adolescents (10-19 years) who have a haemoglobin level less than 11 g/dl and male adolescents less than 12 g/dl at sea level in a country in a given year
Numerator exclusion criteria	
Preferred Data sources	National surveillance system; Population-based survey; School-based survey
Other Data Sources	
Limitations/comments	

References:

gama-list-of-indicators-draft-2-v20201020.pdf (who.int)

For the above indicator, can you provide the below information:

- Can the data elements (numerator and denominator) be collected in the context of Jordan?

☐ Yes Comments:

☐ No Comments:

- Are the data sources listed above applicable (i.e. information can be extracted from the suggested data source) in the context of Jordan? If no, please provide the source.

☐ Yes Comments:

☐ No Comments:

- Can the data for the above indicator be segregated for nationals and refugees?

☐ Yes Comments:

☐ No Comments:

- Is the data for Jordan reported in the same way as indicated above? *If you answer "No", please indicate in the comments section how it is being reported. If the indicator is not being reported in Jordan, please insert "Not reported" in the comments section*

☐ Yes Comments:

☐ No Comments:

- Is the data for this indicator available for the period extending from January 2019 to December 2021? (if yes, please provide the data)

☐ Yes Comments:

☐ No Comments:

Data collection sheet

Indicator	Prevalence of depression among adolescents
Definition	Proportion of adolescents who report experiencing symptoms of depression in a specified period
Rationale	
Type of indicator	
Unit of measure	
Formula	Alternative 1:<br><br>Number of adolescents (> 15 years) who screen positive on the six depression questions in the WMH-CIDI screening tool / Total number of respondents aged 15–19 years in the survey x 100%<br><br>Alternative 2:<br><br>Number of adolescents aged 13–17 years in the GSHS who report having felt lonely and/or having felt so worried about something that they could not sleep (two separate items) during the past 12 months / Total number of respondents aged 13–17 years in the survey x 100%
Target	
Frequency of collection	
Frequency of Dissemination	
Denominator inclusion criteria	Alternative 1: Adolescents aged 10-19<br><br>Alternative 2: Adolescents aged 13-17
Denominator exclusion criteria	
Numerator inclusion criteria	Alternative 1: Information on the number of adolescents (aged 10–19 years, depending on coverage in the sample) in the World Mental Health Composite International Diagnostic Interview (WMH-CIDI) survey who screened positive with the tool .<br><br>Alternative 2: Information on the number of adolescents in the GSHS who reported “having felt lonely” or “having felt so worried they could not sleep” in the past 12 months

Numerator exclusion criteria	
Preferred Data sources	WMH-CIDI survey; data on adolescents are currently available only for subsamples in six countries and were collected for research purposes<br><br>GSHS (“How often have you felt ‘lonely’ and/or ‘worried’ during the past 12 months?”)<br><br>HBSC (“How often have you felt ‘low’ and/or ‘irritable and in a bad temper’?”
Other Data Sources	
Limitations/comments	Measure: Poor data availability, and the GSHS and HBSC measures do not comply with commonly used clinical measures of depression.<br><br>Data availability: The WMH-CIDI survey does not usually cover adolescents (only adults > 18 years)

References:

9789241509626_eng.pdf (who.int)

For the above indicator, can you provide the below information:

- Can the data elements (numerator and denominator) be collected in the context of Jordan?

☐ Yes Comments:

☐ No Comments:

- Are the data sources listed above applicable (i.e. information can be extracted from the suggested data source) in the context of Jordan? If no, please provide the source.

☐ Yes Comments:

☐ No Comments:

- Can the data for the above indicator be segregated for nationals and refugees?

☐ Yes Comments:

☐ No Comments:

- Is the data for Jordan reported in the same way as indicated above? *If you answer "No", please indicate in the comments section how it is being reported. If the indicator is not being reported in Jordan, please insert "Not reported" in the comments section*

☐ Yes Comments:

☐ No Comments:

- Is the data for this indicator available for the period extending from January 2019 to December 2021? (if yes, please provide the data)

☐ Yes Comments:

☐ No Comments:

Prevalence of anaemia among adolescents (10-19 years), by age category (10-14, 15-19 years) and sex

Definition:

Percentage of adolescents (10-19 years) who have a haemoglobin level below 11 g/dl for males and 12 g/dl for females, at sea level, in a country within a given year, by age group (10-14, 15-19 years) and sex

Formula:

Number of female adolescents (10-19 years) who have a haemoglobin level less than 11 g/dl and male adolescents less than 12 g/dl at sea level in a country in a given year*100/ Total population of female and male adolescents (10-19 years) in the same country in the same year

Method of measurement:

1. Collect Data on Denominator

17.4 Include

Total population of female and male adolescents (10-19 years) in the same country in the same year
17.5 Exclude

2. Collect Data on Numerator
2.1. Include
Number of female adolescents (10-19 years) who have a haemoglobin level less than 11 g/dl and male adolescents less than 12 g/dl
2.2. Exclude

3. Stratification Options
Age group (10-14, 15-19 years); Sex

4. Preferred Data Sources
National surveillance system; Population-based survey; School-based survey
5. Other Data Sources

Data collection sheet

Indicator	Prevalence of anaemia among adolescents (10-19 years), by age category (10-14, 15-19 years) and sex
Definition	Percentage of adolescents (10-19 years) who have a haemoglobin level below 11 g/dl for males and 12 g/dl for females, at sea level, in a country within a given year, by age group (10-14, 15-19 years) and sex
Rationale	
Type of indicator	Outcome
Unit of measure	
Formula	Number of female adolescents (10-19 years) who have a haemoglobin level less than 11 g/dl and male adolescents less than 12 g/dl at sea level in a country in a given year*100/ Total population of female and male adolescents (10-19 years) in the same country in the same year
Target	
Frequency of collection	
Frequency of Dissemination	
Denominator inclusion criteria	Total population of female and male adolescents (10-19 years) in the same country in the same year
Denominator exclusion criteria	
Numerator inclusion criteria	Number of female adolescents (10-19 years) who have a haemoglobin level less than 11 g/dl and male adolescents less than 12 g/dl at sea level in a country in a given year
Numerator exclusion criteria	
Preferred Data sources	National surveillance system; Population-based survey; School-based survey
Other Data Sources	
Limitations/comments	

References:

gama-list-of-indicators-draft-2-v20201020.pdf (who.int)

For the above indicator, can you provide the below information:

- Can the data elements (numerator and denominator) be collected in the context of Jordan?

☐ Yes Comments:

☐ No Comments:

- Are the data sources listed above applicable (i.e. information can be extracted from the suggested data source) in the context of Jordan? If no, please provide the source.

☐ Yes Comments:

☐ No Comments:

- Can the data for the above indicator be segregated for nationals and refugees?

☐ Yes Comments:

☐ No Comments:

- Is the data for Jordan reported in the same way as indicated above? *If you answer "No", please indicate in the comments section how it is being reported. If the indicator is not being reported in Jordan, please insert "Not reported" in the comments section*

☐ Yes Comments:

☐ No Comments:

- Is the data for this indicator available for the period extending from January 2019 to December 2021? (if yes, please provide the data)

☐ Yes Comments:

☐ No Comments:

Percentage of schools that have comprehensive health-related school policies

Definition:

Formula:

Method of measurement:

FRESH Checklist 2 can be used to collect information on school-level health-related policies. It must be adapted to each context to reflect the national school health policy (if present) and priorities. The checklist is organized into five sections, with each section corresponding to one of the five Sub-Indicators listed in the Purpose

1. Collect Data on Denominator

17.6 Include

17.7 Exclude

2. Collect Data on Numerator

2.1. Include

2.2. Exclude

3. Stratification Options

4. Preferred Data Sources

5. Other Data Sources

Data collection sheet

Indicator	Prescence of health education materials
Definition	
Rationale	Most schools around the world have school policies, defined as a set of rules and principles that guide schoolrelated activities and operations. School leadership, management committees, staff, parents and students are all expected to agree, abide by and act upon these policies to ensure the school operates effectively and achieves its goal(s). Since children's health and well-being (physical and socio-emotional) are an integral part of quality education, health-related policies are necessary to protect and promote children's health and well-being at school. School health-related policies should reflect both the national school health policy (if present) and priorities, and the local health priorities which may differ between schools. For example, a school located near a busy road may include a policy which focuses on protecting schoolchildren from traffic accidents, whereas a remote rural school may focus on addressing short-term hunger as children walk long distances to school. Core Indicator 2 assesses the extent to which schools have health-related policies, whether these policies address both national and local health priorities and whether they address all aspects of the three other FRESH pillars. The extent to which school health-related policies address local health priorities will depend in part on the level of participation from different stakeholders, particularly children (girls, boys and minority groups), but also parents and community leaders when developing the policy
Type of indicator	
Unit of measure	
Formula	
Target	
Frequency of collection	
Frequency of Dissemination	
Denominator inclusion criteria	
Denominator exclusion criteria	

Numerator inclusion criteria	
Numerator exclusion criteria	
Preferred Data sources	
Other Data Sources	
Limitations/comments	

References:

Helminth_control_in_SHC.indd (who.int)

Layout 1 (unesco.org)

For the above indicator, can you provide the below information:

- Can the data elements (numerator and denominator) be collected in the context of Jordan?

☐ Yes Comments:

☐ No Comments:

- Are the data sources listed above applicable (i.e. information can be extracted from the suggested data source) in the context of Jordan? If no, please provide the source.

☐ Yes Comments:

☐ No Comments:

- Can the data for the above indicator be segregated for nationals and refugees?

☐ Yes Comments:

☐ No Comments:

- Is the data for Jordan reported in the same way as indicated above? *If you answer "No", please indicate in the comments section how it is being reported. If the indicator is not being reported in Jordan, please insert "Not reported" in the comments section*

☐ Yes Comments:

☐ No Comments:

- Is the data for this indicator available for the period extending from January 2019 to December 2021? (if yes, please provide the data)

☐ Yes Comments:

☐ No Comments:

The proportion of adolescents aged 13–15 years considered underweight

Definition:

The proportion of adolescents aged 13–15 years considered underweight.

The standard measurement to be considered underweight is: > 1 SD below weight and height for age and sex, using WHO growth reference for adolescents

Formula:

Number of adolescents aged 13–15 years old considered underweight*100/ Total adolescent respondents aged 13–15 years

Method of measurement:

1. Collect Data on Denominator

17.8 Include

18 Total adolescent respondents aged 13–15 years

18.1 Exclude

2. Collect Data on Numerator

2.1. Include

Number of adolescents aged 13–15 years old considered underweight

2.2. Exclude

3. Stratification Options

Sex

4. Preferred Data Sources

5. Other Data Sources

Data collection sheet

Indicator	**The proportion of adolescents aged 13–15 years considered underweight**<br><br>**Prevalence of Underweight**
Definition	The proportion of adolescents aged 13–15 years considered underweight.<br><br>The standard measurement to be considered underweight is: > 1 SD below weight and height for age and sex, using WHO growth reference for adolescents
Rationale	Being underweight in early adolescence is associated with diarrhoea, malaria, pneumonia and maternal mortality. This risk factor commonly becomes established in childhood, but is associated with adverse consequences during the adolescent years
Type of indicator	
Unit of measure	
Formula	Number of adolescents aged 13–15 years old considered underweight*100/ Total adolescent respondents aged 13–15 years
Target	
Frequency of collection	
Frequency of Dissemination	
Denominator inclusion criteria	Total adolescent respondents aged 13–15 years
Denominator exclusion criteria	
Numerator inclusion criteria	Number of adolescents aged 13–15 years old considered underweight
Numerator exclusion criteria	
Preferred Data sources	
Other Data Sources	
Limitations/comments	

References:

1575_section 7.1.pdf (who.int)

For the above indicator, can you provide the below information:

- Can the data elements (numerator and denominator) be collected in the context of Jordan?

☐ Yes Comments:

☐ No Comments:

- Are the data sources listed above applicable (i.e. information can be extracted from the suggested data source) in the context of Jordan? If no, please provide the source.

☐ Yes Comments:

☐ No Comments:

- Can the data for the above indicator be segregated for nationals and refugees?

☐ Yes Comments:

☐ No Comments:

- Is the data for Jordan reported in the same way as indicated above? *If you answer "No", please indicate in the comments section how it is being reported. If the indicator is not being reported in Jordan, please insert "Not reported" in the comments section*

☐ Yes Comments:

☐ No Comments:

- Is the data for this indicator available for the period extending from January 2019 to December 2021? (if yes, please provide the data)

☐ Yes Comments:

☐ No Comments:

Dimension 2

Percentage of adolescents (10–19 years) who have accumulated at least 60 minutes of moderate-vigorous physical activity daily, and by age and sex (%)

Definition:

The percentage of adolescents (10–19 years) who have accumulated at least 60 minutes of moderate-vigorous physical activity daily

Formula:

Number of adolescents 10–19 years, and by sex, who reported during the survey having accumulated at least 60 minutes of moderate to vigorous physical activity daily in a locality in a specific period of time x 100/ Total population of adolescents 10–19 years, and by sex, in the same locality and period of time interviewed during the survey

Note: as adolescents are subdivided into 3 age categories, this indicator is further sub-divided into 6 sub-indicators. Each sub-indicator will be calculated for each age category 10–12, 13–15 and 16–19 years and by sex

Method of measurement:

1. Collect Data on Denominator
Total population of adolescents 10–19 years

18.2 **Include**
19

19.1 **Exclude**

2. Collect Data on Numerator
Number of adolescents 10–19 years, and by sex, who reported during the survey having accumulated at least 60 minutes of moderate to vigorous physical activity

2.1. Include

2.2. Exclude

3. Stratification Options
Sex and age

4. Preferred Data Sources
1. Adolescent health structure in Ministry of Health

2. Global school health survey

3. Stepwise survey

4. School health programme/Ministry of Health

5. Household survey

6. Special studies

5. Other Data Sources

Data collection sheet

Indicator	Percentage of adolescents (10–19 years) who have accumulated at least 60 minutes of moderate-vigorous physical activity daily, and by age and sex (%)
Definition	The percentage of adolescents (10–19 years) who have accumulated at least 60 minutes of moderate-vigorous physical activity daily
Rationale	Physical activity is a key determinant of energy expenditure, and thus is fundamental to energy balance and weight control. Physical activity reduces the risk for cardiovascular diseases and diabetes and has substantial benefits for many conditions, not only those associated with obesity. The beneficial effects of physical activity on the metabolic syndrome are mediated by mechanisms beyond controlling excess body weight. For example, physical activity reduces blood pressure, improves the level of high density lipoprotein cholesterol and improves control of blood glucose in overweight people, even without significant weight loss, and reduces the risk for colon cancer and breast cancer among women
Type of indicator	
Unit of measure	Percentage
Formula	Number of adolescents 10–19 years, and by sex, who reported during the survey having accumulated at least 60 minutes of moderate to vigorous physical activity daily in a locality in a specific period of time x 100/ Total population of adolescents 10–19 years, and by sex, in the same locality and period of time interviewed during the survey
Target	
Frequency of collection	
Frequency of Dissemination	
Denominator inclusion criteria	Total population of adolescents 10–19 years
Denominator exclusion criteria	

Numerator inclusion criteria	Number of adolescents 10–19 years, and by sex, who reported during the survey having accumulated at least 60 minutes of moderate to vigorous physical activity daily
Numerator exclusion criteria	
Preferred Data sources	
Other Data Sources	
Limitations/comments	

References:

Microsoft Word - Adolescent Health Indicators regional guide-Final.docx (who.int)

For the above indicator, can you provide the below information:

- Can the data elements (numerator and denominator) be collected in the context of Jordan?

☐ Yes Comments:

☐ No Comments:

- Are the data sources listed above applicable (i.e. information can be extracted from the suggested data source) in the context of Jordan? If no, please provide the source.

☐ Yes Comments:

☐ No Comments:

- Can the data for the above indicator be segregated for nationals and refugees?

☐ Yes Comments:

☐ No Comments:

- Is the data for Jordan reported in the same way as indicated above? *If you answer "No", please indicate in the comments section how it is being reported. If the indicator is not being reported in Jordan, please insert "Not reported" in the comments section*

☐ Yes Comments:

☐ No Comments:

- Is the data for this indicator available for the period extending from January 2019 to December 2021? (if yes, please provide the data)

☐ Yes Comments:

☐ No Comments:

Prevalence of Vitamin D Deficiency Among Healthy Adolescents

Definition:

By using a broader definition, 25-hydroxyvitamin D level of ≤20 ng/mL or ≤50 nmol/L) is defined as vitamin D insufficient

Formula:

Total number of surveyed adolescents with 25-hydroxyvitamin D level of ≤20 ng/mL or ≤50 nmol/L *100 / Total number of surveyed adolescents

Method of measurement:

1. Collect Data on Denominator
Total number of surveyed adolescents

19.2 Include
20

20.1 Exclude

2. Collect Data on Numerator
Total number of surveyed adolescents with 25-hydroxyvitamin D level of ≤20 ng/mL or ≤50 nmol/L

2.1. Include

2.2. Exclude

3. Stratification Options
Age, sex

4. Preferred Data Sources
National Survey

5. Other Data Sources

Data collection sheet

Indicator	Prevalence of Vitamin D Deficiency Among Healthy Adolescents
Definition	By using a broader definition, 25-hydroxyvitamin D level of ≤20 ng/mL or ≤50 nmol/L) is defined as vitamin D insufficient
Rationale	
Type of indicator	
Unit of measure	
Formula	Total number of surveyed adolescents with 25-hydroxyvitamin D level of ≤20 ng/mL or ≤50 nmol/L *100 / Total number of surveyed adolescents
Target	
Frequency of collection	
Frequency of Dissemination	
Denominator inclusion criteria	Total number of surveyed adolescents
Denominator exclusion criteria	
Numerator inclusion criteria	Total number of surveyed adolescents with 25-hydroxyvitamin D level of ≤20 ng/mL or ≤50 nmol/L
Numerator exclusion criteria	
Preferred Data sources	National Survey
Other Data Sources	
Limitations/comments	

References:

Vitamin D and adolescent health (nih.gov)

gordon2004.pdf (vitaminedelft.org)

For the above indicator, can you provide the below information:

- Can the data elements (numerator and denominator) be collected in the context of Jordan?

☐ Yes Comments:

☐ No Comments:

- Are the data sources listed above applicable (i.e. information can be extracted from the suggested data source) in the context of Jordan? If no, please provide the source.

☐ Yes Comments:

☐ No Comments:

- Can the data for the above indicator be segregated for nationals and refugees?

☐ Yes Comments:

☐ No Comments:

- Is the data for Jordan reported in the same way as indicated above? *If you answer "No", please indicate in the comments section how it is being reported. If the indicator is not being reported in Jordan, please insert "Not reported" in the comments section*

☐ Yes Comments:

☐ No Comments:

- Is the data for this indicator available for the period extending from January 2019 to December 2021? (if yes, please provide the data)

☐ Yes Comments:

☐ No Comments:

Current illicit drug use among adolescents (10-19 years old)

Definition:

Proportion of adolescents who report use illicit drugs (including marijuana (cannabis) , cocaine, inhalants, hallucinogens, heroin, or any non-medical use of analgesics, tranquilizers, stimulants, or sedatives) in the past 30 days

Formula:

Number of adolescents who report use of illicit drugs one or more times in the past 30 days (in the survey) /Total number of adolescent respondents in the survey x 100%

Method of measurement:

Number of adolescents who report use of illicit drugs one or more times in the past 30 days (in the survey) /Total number of adolescent respondents in the survey x 100%

1. Collect Data on Denominator

20.2 Include

21 Total number of adolescent respondents in the survey

21.1 Exclude

2. Collect Data on Numerator

2.1. Include

Number of adolescents who report use of illicit drugs one or more times in the past 30 days

2.2. Exclude

3. Stratification Options

Sex ,age and type of illicit drug

4. Preferred Data Sources

Population Survey

5. Other Data Sources

HBSC and GSHS

Data collection sheet

Indicator	CURRENT CANNABIS USE AMONG ADOLESCENTS
Definition	Proportion of adolescents who report use of cannabis in the past 30 days
Rationale	
Type of indicator	
Unit of measure	
Formula	Number of adolescents who report use of illicit drugs one or more times in the past 30 days (in the survey) /Total number of adolescent respondents in the survey x 100%
Target	
Frequency of collection	
Frequency of Dissemination	
Denominator inclusion criteria	Total number of adolescent respondents in the survey
Denominator exclusion criteria	
Numerator inclusion criteria	Number of adolescents who report use of illicit drugs one or more times in the past 30 days (in the survey)
Numerator exclusion criteria	
Preferred Data sources	Population Survey
Other Data Sources	HBSC and GSHS
Limitations/comments	Specific ages may vary by survey population

References:

9789241509626_eng.pdf (who.int)

Hawaii Health Matters :: Indicators :: Adolescent Use of Alcohol or Illicit Drugs :: State : Hawaii

Use and Abuse of Alcohol and Illicit Drugs in US Adolescents (nih.gov)

For the above indicator, can you provide the below information:

- Can the data elements (numerator and denominator) be collected in the context of Jordan?

☐ Yes Comments:

☐ No Comments:

- Are the data sources listed above applicable (i.e. information can be extracted from the suggested data source) in the context of Jordan? If no, please provide the source.

☐ Yes Comments:

☐ No Comments:

- Can the data for the above indicator be segregated for nationals and refugees?

☐ Yes Comments:

☐ No Comments:

- Is the data for Jordan reported in the same way as indicated above? *If you answer "No", please indicate in the comments section how it is being reported. If the indicator is not being reported in Jordan, please insert "Not reported" in the comments section*

☐ Yes Comments:

☐ No Comments:

- Is the data for this indicator available for the period extending from January 2019 to December 2021? (if yes, please provide the data)

☐ Yes Comments:

☐ No Comments:

Dimension 3:

SDG 3.7.2Adolescent birthrate (per 1000 women aged 10-14; 15-19 years)

Formula

$$\frac{\text{Number of live births to women15 to 19 years of age}}{\text{Estimate of exposure to childbearing by women 15 to 19 years of age}} \times 1,000$$

Data collection & dissemination:

- Data dissemination based on type of resource used:
  - Civil registration: Annual
  - Surveys: every 3–5 years
  - Census data: 5–10 years

Method of measurement:

The adolescent birth rate is generally computed as a ratio. The numerator is the number of live births to women 15 to 19 years of age, and the denominator an estimate of exposure to childbearing by women 15 to 19 years of age.
The computation is the same for the age group 10-14 years

Collect Data on Denominator
Denominator: Estimate of exposure to childbearing by women 15 to 19 years of age

1.1 Include

- If source is civil registration: the estimated or enumerated population of women aged 15 to 19 during a given year
- If source is survey data: person-years lived between the ages of 15 and 19 by the interviewed women during the same reference period. The reference period corresponds to the five years preceding the survey, or if no retrospective birth histories are available the estimate is based on the date of last birth or the number of births in the 12 months preceding the survey
- If source is census data: the adolescent birth rate is generally computed based on the date of last birth or the number of births in the 12 months preceding the enumeration
- Whenever data are available, adolescent fertility at ages 10-14 years can also be computed.

1.2 Exclude

- NA

2. Collect Data on Numerator
Numerator:

- Number of live births to women15 to 19 years of age

2.1 Include

- If source is civil registration: registered number of live-births born to women 15 to 19 years of age during a given year
- If source is survey data: births to women that were 15 to 19 years of age at the time of the birth during a reference period before the interview. The reference period corresponds to the five years preceding the survey, or if no retrospective birth histories are available the estimate is based on the date of last birth or the number of births in the 12 months preceding the survey
- If source is census data: the adolescent birth rate is generally computed based on the date of last birth or the number of births in the 12 months preceding the enumeration
- Whenever data are available, adolescent fertility at ages 10-14 years can also be computed.

2.2 Exclude

- NA

3. This indicator will be stratified based on:

- geographical area: rural or urban residence
- women's level of education
- poverty status
- other characteristics that are relevant in the national context to help identify population sub-groups

Disaggregation based on the type of resource used:

- Civil registration: maternal age, sex, place of birth, place of residence (e.g., urban, rural), subnational administrative units (e.g., districts, provinces, regions) and type of reporting source (e.g., health facility, community).
- Surveys: Socioeconomic status (e.g., education level, household wealth quintile), age of woman at the time of interview, place of residence (e.g. urban, rural), subnational administrative units (e.g., districts, provinces, regions), total number of living children at the time of interview, total number of antenatal care (ANC) visits, and timing of first ANC.
- Census data: Age, education level, marital status, number of living children, place of residence, socioeconomic status.

4. Data Sources

4.1 Preferred data Sources:

- Civil registration with complete coverage

4.2 Other data Sources:

- Population census
- Household surveys
- Routinely collected administrative data
- Population-based household surveys
- Nationally representative census data

5. Calculate:

Divide the number of births that occurred to women aged 15–19 years at the time of the birth in the specific period before the survey by the number of women-years of exposure of women aged 15–19 years during that period and multiplied by 1000

6. Possible Limitations:

- The adolescent birth rate is commonly reported as the age-specific fertility rate for ages 15 to 19 in the context of calculation of total fertility estimates. A related measure is the proportion of adolescent fertility measured as the percentage of total fertility contributed by women aged 15-19.
- Adolescent birth rates are subject to limitations which depend on the completeness of birth registration; the treatment of infants born alive but dead before registration or within the first 24 hours of life; the precision of the reported age of the mother; and the inclusion of births from previous periods
- The number of live births may also include births to women below age 15
- Calculated for different age groups for both the number of live births and the number of women
- age misreporting
- birth omissions
- misreporting the date of birth of the child
- sampling variability

Data Collection Sheet

The following table contains detailed information and support from the literature on the measurement of the indicator.

Indicator	Adolescent Birth rate (per 1000 women aged 10-14; 15-19 years)
Definition	The annual number of births to women aged 15–19 years per 1000 women in that age group. It is also referred to as the age-specific fertility rate for women aged 15–19 years.
Rationale	The adolescent birth rate, technically known as the age-specific fertility rate provides a basic measure of reproductive health focusing on a vulnerable group of adolescent women. There is substantial agreement in the literature that women who become pregnant and give birth very early in their reproductive lives are subject to higher risks of complications or even death during pregnancy and birth and their children are also more vulnerable. Therefore, preventing births very early in a woman's life is an important measure to improve maternal health and reduce infant mortality. Furthermore, women having children at an early age experience a curtailment of their opportunities for socio-economic improvement, particularly because young mothers are unlikely to keep on studying and, if they need to work, may find it especially difficult to combine family and work responsibilities. The adolescent birth rate provides also indirect evidence on access to reproductive health since the youth, and in particular unmarried adolescent women, often experience difficulties in access to reproductive health care[14]
Type of indicator	Impact
Unit of measure	Births per 1000 women in the respective age group
Method of measurement	The adolescent birth rate is generally computed as a ratio. The numerator is the number of live births to women 15 to 19 years of age, and the denominator an estimate of exposure to childbearing by women 15 to 19 years of age. The numerator and the denominator are calculated differently for civil registration, survey and census data. (a) In the case of civil registration the numerator is the registered number of live-births born to women 15 to 19 years of age during a given year, and the denominator is the estimated or enumerated population of women aged 15 to 19. (b) In the case of survey data, the adolescent birth rate is generally computed based on retrospective birth histories. The numerator refers to births to women that were 15 to 19 years of age at the time of the birth during a reference period before the interview, and the denominator to person-years lived between the ages of 15 and 19 by the interviewed women during the same reference period. Whenever possible, the reference period corresponds to the five years preceding the survey. The reported observation year corresponds to the middle of the reference period. For some surveys, no retrospective birth histories are available and the estimate is based on the date of last birth or the number of births in the 12 months preceding the survey. (c) In the case of census data, the adolescent birth rate is generally computed based on the date of last birth or the number of births in the 12 months preceding the enumeration. The census provides both the numerator and the denominator for the rates. In some cases, the rates based on censuses are adjusted for under registration based on indirect methods of estimation. For some countries with no other reliable data, the own-children method of indirect estimation provides estimates of the adolescent birth rate for a number of years before the census.

Formula	(Number of live births to women 15 to 19 years of age/Estimate of exposure to childbearing by women 15 to 19 years of age) x 1,000
Target	Available data should cover at least 50% of total number of women aged 15-19 years in the regional or global groupings.
Frequency	▪ Data dissemination based on type of resource used:<br>- Civil registration: Annual<br>- Surveys: every 3–5 years<br>- Census data: 5–10 years
Possible Limitations	▪ The adolescent birth rate is commonly reported as the age-specific fertility rate for ages 15 to 19 in the context of calculation of total fertility estimates. A related measure is the proportion of adolescent fertility measured as the percentage of total fertility contributed by women aged 15-19.<br>▪ Adolescent birth rates are subject to limitations which depend on the completeness of birth registration; the treatment of infants born alive but dead before registration or within the first 24 hours of life; the precision of the reported age of the mother; and the inclusion of births from previous periods<br>▪ The number of live births may also include births to women below age 15<br>▪ Calculated for different age groups for both the number of live births and the number of women<br>▪ age misreporting<br>▪ birth omissions<br>▪ misreporting the date of birth of the child<br>▪ sampling variability
Denominator inclusion criteria	▪ If source is civil registration: the estimated or enumerated population of women aged 15 to 19 during a given year<br>▪ If source is survey data: person-years lived between the ages of 15 and 19 by the interviewed women during the same reference period. The reference period corresponds to the five years preceding the survey, or if no retrospective birth histories are available the estimate is based on the date of last birth or the number of births in the 12 months preceding the survey<br>▪ If source is census data: the adolescent birth rate is generally computed based on the date of last birth or the number of births in the 12 months preceding the enumeration
Denominator exclusion criteria	▪ NA
Numerator inclusion criteria	▪ If source is civil registration: registered number of live-births born to women 15 to 19 years of age during a given year<br>▪ If source is survey data: births to women that were 15 to 19 years of age at the time of the birth during a reference period before the interview. The reference period corresponds to the five years

	preceding the survey, or if no retrospective birth histories are available the estimate is based on the date of last birth or the number of births in the 12 months preceding the survey<br>▪ If source is census data: the adolescent birth rate is generally computed based on the date of last birth or the number of births in the 12 months preceding the enumeration<br>▪
Numerator exclusion criteria	▪ NA
Data sources	▪ Civil registration with complete coverage<br>▪ Population census<br>▪ Household surveys<br>▪ Routinely collected administrative data<br>▪ Population-based household surveys<br>▪ Nationally representative census data

References:

Indicator Metadata Registry Details. (2020). Retrieved 17 December 2020, from https://www.who.int/data/gho/indicator-metadata-registry/imr-details/3

Metadata-03-07-02.pdf (un.org)

For the above indicator, can you provide the below information: NA

- Can the data elements (numerator and denominator) be collected in the context of Jordan?

☐ Yes Comments:

☐ No Comments:

- Are the data sources listed above applicable (i.e. information can be extracted from the suggested data source) in the context of Jordan? If no, please provide the source.

☐ Yes Comments:

☐ No Comments:

- Can the data for the above indicator be segregated for nationals and refugees?

☐ Yes Comments:

☐ No Comments:

- Is the data for Jordan reported in the same way as indicated above? *If you answer "No", please indicate in the comments section how it is being reported. If the indicator is not being reported in Jordan, please insert "Not reported" in the comments section*

☐ Yes Comments:

☐ No Comments:

- Is the data for this indicator available for the period extending from January 2019 to December 2021? (if yes, please provide the data)

☐ Yes Comments:

☐ No Comments:

Dimension 4:

Dental visits among children and adolescents aged 1-17 years

Definition:

Dental visits among children and adolescents aged 1-17 years

Formula:

Children and adolescents aged 1-17 years with parent-reported dental visit for any kind of dental care, including check-ups, dental cleanings, x-rays, or filling cavities in the previous year/ Children and adolescents aged 1-17 years

Method of measurement:

1. Collect Data on Denominator
Children and adolescents aged 1-17 years

21.2 Include
22

22.1 Exclude
unknowns and refusals

2. Collect Data on Numerator
Children and adolescents aged 1-17 years with parent-reported dental visit for any kind of dental care, including check-ups, dental cleanings, x-rays, or filling cavities

2.1. Include

2.2. Exclude

3. Stratification Options
Age, sex

4. Preferred Data Sources

5. Other Data Sources

Data collection sheet

Indicator	Dental visits among children and adolescents aged 1-17 years
Definition	Dental visits among children and adolescents aged 1-17 years
Rationale	Access to dental care is important to obtain prevention, education, and early identification and treatment of oral diseases.2 The American Academy of Pediatric Dentistry, the American Academy of Pediatrics, the American Dental Association, and the American Association of Public Health Dentistry recommend establishing a dental home and the first dental visit by age 1 year. Referring a child for an oral health examination by a dentist who provides care for infants and young children 6 months after the first tooth erupts or by 12 months of age establishes the child's dental home and provides an opportunity to implement preventive dental health habits that meet each child's unique needs and keep the child free from dental or oral disease. Private and public funds are spent each year for emergency department visits due to oral health conditions and for providing restorations for the children that could have potentially been avoided with routine and optimal preventive and early dental care
Type of indicator	
Unit of measure	
Formula	Children and adolescents aged 1-17 years with parent-reported dental visit for any kind of dental care, including check-ups, dental cleanings, x-rays, or filling cavities in the previous year/ Children and adolescents aged 1-17 years
Target	
Frequency of collection	
Frequency of Dissemination	
Denominator inclusion criteria	Children and adolescents aged 1-17 years
Denominator exclusion criteria	unknowns and refusals
Numerator inclusion criteria	Children and adolescents aged 1-17 years with parent-reported dental visit for any kind of dental care, including check-ups, dental cleanings, x-rays, or filling cavities in the previous year

Numerator exclusion criteria	
Preferred Data sources	
Other Data Sources	
Limitations/comments	Indicator does not validate types of dental care children actually received

References:

Indicator Definitions - Oral Health | CDI | DPH | CDC

For the above indicator, can you provide the below information:

- Can the data elements (numerator and denominator) be collected in the context of Jordan?

☐ Yes Comments:

☐ No Comments:

- Are the data sources listed above applicable (i.e. information can be extracted from the suggested data source) in the context of Jordan? If no, please provide the source.

☐ Yes Comments:

☐ No Comments:

- Can the data for the above indicator be segregated for nationals and refugees?

☐ Yes Comments:

☐ No Comments:

- Is the data for Jordan reported in the same way as indicated above? *If you answer "No", please indicate in the comments section how it is being reported. If the indicator is not being reported in Jordan, please insert "Not reported" in the comments section*

☐ Yes Comments:

☐ No Comments:

- Is the data for this indicator available for the period extending from January 2019 to December 2021? (if yes, please provide the data)

☐ Yes Comments:

☐ No Comments:
